# Phosphodiesterase 4D contributes to angiotensin II-induced abdominal aortic aneurysm through smooth muscle cell apoptosis

**DOI:** 10.1038/s12276-022-00815-y

**Published:** 2022-08-23

**Authors:** Ran Gao, Wenjun Guo, Tianfei Fan, Junling Pang, Yangfeng Hou, Xiaohang Feng, Bolun Li, Weipeng Ge, Tianhui Fan, Tiantian Zhang, Jiakai Lu, He Jing, Mu Jin, Chen Yan, Jing Wang

**Affiliations:** 1 State Key Laboratory of Medical Molecular Biology, Institute of Basic Medical Sciences, Chinese Academy of Medical Sciences, Department of Pathophysiology, Peking Union Medical College, Beijing, China; 2 Department of Anesthesiology, Beijing Anzhen Hospital, Capital Medical University, Beijing Institute of Heart, Lung and Blood Vessel Diseases, Beijing, China; 3 Department of Anesthesiology, Beijing Friendship Hospital, Capital Medical University, Beijing, China; 4 Aab Cardiovascular Research Institute, University of Rochester, School of Medicine and Dentistry, Rochester, NY 14642 USA

**Keywords:** Aneurysm, Apoptosis

## Abstract

Abdominal aortic aneurysm (AAA) is a permanent expansion of the abdominal aorta that has a high mortality but limited treatment options. Phosphodiesterase (PDE) 4 family members are cAMP-specific hydrolyzing enzymes and have four isoforms (PDE4A-PDE4D). Several pan-PDE4 inhibitors are used clinically. However, the regulation and function of PDE4 in AAA remain largely unknown. Herein, we showed that PDE4D expression is upregulated in human and angiotensin II-induced mouse AAA tissues using RT-PCR, western blotting, and immunohistochemical staining. Furthermore, smooth muscle cell (SMC)-specific *Pde4d* knockout mice showed significantly reduced vascular destabilization and AAA development in an experimental AAA model. The PDE4 inhibitor rolipram also suppressed vascular pathogenesis and AAA formation in mice. In addition, PDE4D deficiency inhibited caspase 3 cleavage and SMC apoptosis in vivo and in vitro, as shown by bulk RNA-seq, western blotting, flow cytometry and TUNEL staining. Mechanistic studies revealed that PDE4D promotes apoptosis by suppressing the activation of cAMP-activated protein kinase A (PKA) instead of the exchange protein directly activated by cAMP (Epac). Additionally, the phosphorylation of BCL2-antagonist of cell death (Bad) was reversed by PDE4D siRNA in vitro, which indicates that PDE4D regulates SMC apoptosis via the cAMP-PKA-pBad axis. Overall, these findings indicate that PDE4D upregulation in SMCs plays a causative role in AAA development and suggest that pharmacological inhibition of PDE4 may represent a potential therapeutic strategy.

## Aortic aneurysm: Targeting a key enzyme

An enzyme that promotes dangerous expansion of the abdominal aorta could be targeted by new therapies. Abdominal aortic aneurysm (AAA) is an irreversible and often fatal dilation of the abdominal aorta, which can currently only be treated through invasive surgery. Now, Jing Wang at Peking Union Medical College in Beijing, China, and co-workers have shown that the enzyme phosphodiesterase 4D (PDE4D) is strongly expressed in human and mouse AAA tissues. Far fewer mice in which PDE4D expression had been knocked out developed AAA. The researchers revealed the molecular mechanisms through which PDE4D promotes the death of smooth muscle cells; these cells would usually maintain healthy aortic function by contracting and expanding in response to blood flow. Drugs that inhibit PDE4D could therefore form part of an effective therapeutic strategy.

## Introduction

Abdominal aortic aneurysm (AAA) is a chronic vessel wall degenerative disease characterized by the irreversible progressive dilatation of the abdominal aorta over 3.0 cm (1.5-fold of normal abdominal aorta)^1^. AAA is a multifactorial disease that predominantly affects elderly males 65 years or older^2^. AAA rupture is a life-threatening medical emergency, with mortality rates > 81%^3^. At present, there are no effective clinical medicines to prevent, delay, or reverse the growth or rupture of AAA, except surgical repair^4^. Therefore, understanding the molecular mechanisms and identifying regulators underlying the pathogenesis of AAA are essential for developing potential therapeutic strategies. Smooth muscle cells (SMCs) are the major cell type in the medial layer of large arteries. SMCs regulate vascular contractility in response to pulsatile blood flow and pressure in aortas. SMCs are the major contributors to elastin fibers that maintain the elasticity of aortas. Increasing evidence has indicated that the loss of vascular SMCs contributes to aortic wall weakening, dilation, and aneurysm formation^5,6^.

The second messenger cyclic nucleotides 3′,5′-cyclic adenosine monophosphate (3′,5′-cAMP) and 3′,5′-cyclic guanosine monophosphate (3′,5′-cGMP) are important in regulating smooth muscle contractile function and vessel wall structure integrity. Abnormal cyclic nucleotide homeostasis contributes to a variety of cardiovascular diseases^7–9^. Cyclic nucleotide phosphodiesterases (PDEs) are a superfamily of enzymes responsible for hydrolyzing cyclic nucleotides and thus play crucial roles in regulating the duration, magnitude, and compartmentalization of cyclic nucleotide responses^10^. There are 11 families within the PDE superfamily (PDE1 to PDE11), and each family contains multiple subtypes. PDE dysregulation has been associated with numerous diseases^11^. Over the past decades, PDEs have been proven to be ideal and feasible drug targets. Several family-specific PDE inhibitors are currently used or are currently under clinical trials for the treatment of a variety of diseases^12–14^.

A few reports have suggested potential links between cAMP signaling and AAA. For example, ablation of SMC-specific *Gsα*, a G-protein responsible for cAMP production, exaggerated AAA formation in mice^15^. Cilostazol, an inhibitor of cAMP-hydrolyzing PDE3 and adenosine uptake^16^, suppressed AAA in rats and mice^17^. The mechanistic action of cilostazol in AAA remains to be clarified: cAMP versus adenosine. Recently, we reported that PDE1C, a Ca^2+^/calmodulin-stimulated PDE, contributed to AAA by promoting SMC senescence by suppressing cAMP-mediated activation of SIRT1^18^. These previous studies showed a protective effect of cAMP on AAA prevention. However, the roles of different cAMP signaling pathways regulated by distinct cAMP-PDEs in AAA remain unclear. PDE4 family members are cAMP-specific hydrolyzing enzymes encoded by four isoforms: PDE4A, 4B, 4C, and 4D. Several PDE4 family-selective inhibitors—such as rolipram, roflumilast, apremilast, and crisaborole—have been used to clinically treat inflammatory diseases, including inflammatory airway diseases, psoriatic arthritis, and atopic dermatitis^19^. Understanding the roles of PDE4 isozymes and the effects of PDE4 inhibitors in AAA is critical for developing potential therapeutic strategies.

In this study, we aimed to explore the regulation, function, and mechanistic action of the PDE4 isozyme in AAA pathogenesis and development. Because our pilot study showed that PDE4D is most prominently upregulated in SMCs of human and mouse AAA tissues, we focused on SMC PDE4D in the current study. Furthermore, PDE4D deficiency improved vascular pathogenesis and AAA development in SMC-specific *Pde4d* knockout mice. The PDE4 inhibitor rolipram protected against AAA in mice. In addition, we demonstrated that PDE4D promotes SMC apoptosis via the cAMP-PKA-pBad axis.

## Materials and methods

### Animal models

All animal procedures were performed in accordance with Peking Union Medical College guidelines (Animal Care and Use Committee of Peking Union Medical College accreditation number: ACUC-A01-2017-007). *Pde4d*-floxed mice *(Pde4d*^*flox/flox*^) were generated in and obtained from the Shanghai Model Organisms Center (Shanghai, China) through homologous recombination using CRISPR/Cas9 technology. *Pde4d*-floxed mice carry floxed exon 10 of the *Pde4d* gene. *Pde4d*^*flox/flox*^ mice were crossed with *Apoe*^*−/−*^ mice. For generation of SMC-specific knockout mice on the *Apoe*^*−/−*^ background (*Apoe*^*−/−*^*Pde4d*^*SMC−/-*^), *Apoe*^*−/−*^ mice carrying the floxed *Pde4d* allele were crossed with *Tagln*-Cre mice (Shanghai Model Organisms Center, China). For genotyping, a One Step Mouse Genotyping Kit (Vazyme Biotech Co., Ltd., PD101-01, Nanjing, China) was used to test tail biopsies of mice by PCR. Primers targeting the wild-type allele, the floxed allele, and cre recombinase are listed in Supplementary Table 2. After genotyping analysis, *Apoe*^*−/−*^*Pde4d*^*flox/flox*^ and *Apoe*^*−/−*^*Pde4d*^*SMC−/−*^ littermates were used for study. Only male mice were used, as AAA primarily affects elderly males in humans^2^.

For induction of AAA, eight-week-old male mice were infused with angiotensin II (Ang II, 1000 ng kg^−1^ min^−1^; Sigma, Cat#: A9525-50MG, US) subcutaneously implanted with osmotic pumps (Alzet MODEL 2004; DURECT, US) for twenty-eight days and fed a high-fat diet (HFD; Research Diets, Cat#: D12108C, US), as described previously^20^. For a model with the important features of human AAA, the main method used in animal models is subcutaneous infusion of Ang IIto induce AAA in mice^20–22^. Based on previous experience and literature reports^20,23^, four groups of mice were randomly treated as follows: *Apoe*^*−/−*^*Pde4d*^*flox/flox*^ mice treated with saline (*n* = 6), *Apoe*^*−/−*^*Pde4d*^*SMC−/−*^ mice treated with saline (*n* = 6), *Apoe*^*−/−*^*Pde4d*^*flox/flox*^ mice treated with Ang II (*n* = 17), and *Apoe*^*−/−*^*Pde4d*^*SMC−/−*^ mice treated with Ang II (*n* = 16).

For determination of the effect of rolipram on AAA, *Apoe*^*−/−*^ male mice (C57BL/6, N11, The Jackson Laboratory, US) at eight weeks old were treated as mentioned above. A total of 0.375 mg mL^−1^ rolipram (PDE4 inhibitor, 8 mL kg^−1^ d^−1^, dissolved in ethyl alcohol; Sigma-Aldrich, R6520) was administered orally via gavage daily for twenty-eight days. The mice were randomly divided into four groups: Ang II (−) mice treated with 7% ethyl alcohol (3 mg kg^−1^ d^−1^; *n* = 5), Ang II (−) mice treated with rolipram (*n* = 6), Ang II (+) mice treated with 7% ethyl alcohol (3 mg kg^−1^ d^−1^; *n* = 23), and Ang II (+) mice treated with rolipram (*n* = 16).

AAA was defined as 50% dilation in the diameter of the external abdominal aorta compared with the normal abdominal aorta of the control group. The aortas were excised, and the maximal external diameter of the abdominal aorta was measured by two different investigators using a stereoscope at the time of necropsy. The animals were raised in a specific pathogen-free (SPF) facility.

### Human aortic tissues

Human specimens were obtained under protocols approved by the Ethics Committee of Beijing Anzhen Hospital, Capital Medical University. Human samples from the anterior region of the aneurysmal aortic wall were obtained from nine AAA patients who had undergone open aortic aneurysm surgery repair (Project Number: 2017-051X). The patients were diagnosed with AAA by repeated ultrasonography or CT angiography. Specimens from patients with collagen vascular disease or severe chronic kidney disease (estimated glomerular filtration rate < 30 mL [min·1.73 m^2^]^−1^) were excluded. Samples were snap-frozen in liquid nitrogen directly after surgery. Corresponding adjacent human aortic non-AAA tissue specimens used for controls were collected from the bodies of six deceased donors with no detectable vascular disease (Volunteer Corpse Donation Reception Station). Samples in the freezers were snap-frozen in liquid nitrogen after dissection. Informed consent was obtained from all study participants. The clinical information associated with the samples is shown in Supplementary Table 1.

### RNA sequencing analysis

The filtered and trimmed reads of each sample were aligned to the rat reference genome (Rnor_6.0) using HISAT2 software with default parameters^24,25^. The read counts of genes were calculated using the R packages Genomic Features and Genomic Alignments^26^. The FPKM (fragments per kilobase million) of genes was determined using the DESeq2 package^27^. Differentially expressed genes were identified using a paired *t* test with *p* value < 0.1. Pathway enrichment analysis was performed using the MetaCore online tool (https://portal.genego.com/). An FDR cutoff of < 0.05 was set to select for significant pathways. The relationship between pathways and genes was visualized using the R package GOplot (v1.0.2)^28^.

### Histological, immunohistochemical, and immunofluorescent analysis

Human aortic tissue samples were vertically embedded in optimal cutting temperature compound (O.C.T, Thermo Fisher, US) and stored at −80 °C. Mouse AAA aortas were dissected at the maximal suprarenal outer aortic diameter and embedded vertically in O.C.T compound for histological evaluation. Corresponding adjacent normal aortic vascular tissues were collected from non-Ang II-infused control mice. At least ten-fifteen serial frozen sections, six μm thick, were sectioned from the AAA portion, covering the maximal dilated aorta and control tissues using a freezing microtome (Leica CM1860, Germany). Immunohistochemical staining was performed as previously described^29,30^. Tissue sections were incubated with rabbit anti-PDE4D (1:100, Abcam, ab14613, Britain) antibody overnight at 4 °C, followed by incubation with anti-rabbit IgG-peroxidase conjugate (Beijing Zsbio Biotechnology, China) for thirty mins at room temperature and developed by adding the substrate 3-amino-9-ethylcarbazole (AEC). Images were taken with a microscope (Nikon, digital sight DS—Fi2, Germany). For quantitative analysis, the positive staining intensity was semiquantified with Image-Pro Plus Software (Media Cybernetics, Bethesda, MD). For each animal, a total of five fields were randomly selected, quantified, and averaged. For negative controls, sections were incubated with goat anti-rabbit secondary antibody using the primary antibody omitted (Supplementary Fig. 1a–c). In addition, mouse aortic sections were stained for elastin staining (Modified Verhoeff Van Gieson Elastic Stain Kit, Sigma, Cat#: HT25A, US). Elastin staining is indicated by the darkest color, and elastin degradation was rated according to the key provided by the manufacturer^31^. Mouse heart tissues were stained with hematoxylin and eosin (H&E) (Solarbio, Cat#: G1120, China).

Immunofluorescence analysis of PDE4D (1:50, Abcam, ab14613, Britain), α-SMA (1:250, Invitrogen, 14-9760-82, Britain), and terminal deoxynucleotidyl transferase (TdT)-mediated dUTP nick end labeling (TUNEL; Beyotime Biotechnology, China, Cat#: C1088) was performed. The sections were coincubated overnight at 4 °C with primary antibodies against PDE4D and α-SMA. The antibodies were detected using fluorescein-labeled secondary antibodies. The nuclei were stained with 4’,6-diamidino-2-phenylindole (DAPI) (Abcam, Cat#: ab228549, Britain). Images were photographed using a fluorescence microscope (Nikon, digital sight DS—Fi2, Japan). For negative controls, adjacent sections were incubated with fluorescein-labeled secondary antibodies using the primary antibody omitted (Supplementary Fig. 1e, f). Apoptotic cells were examined by TUNEL staining and quantified as the ratio of apoptotic cells to total cells in 5 fields per randomly selected mouse using Image-Pro Plus Software. For negative controls, adjacent sections were incubated with dUTP and without TdT (Supplementary Fig. 15).

### Measurement of blood pressure by tail-cuff plethysmography

Systolic and diastolic blood pressure were measured via the noninvasive tail-cuff method (CODA, Kent Scientific, US) before osmotic pump implantation and later sacrifice as described^32^. Briefly, mice were warmed to ~37 °C in restraint tubes on the heating pad with their tails restrained in the occlusion tail cuff. Mice were trained for ~3–15 min until a stable blood pressure was recorded. The average of at least five successful measurements was considered the blood pressure of the mice on that recording day.

### Cell culture and small interfering RNA (siRNA)

Rat aortic smooth muscle cells (SMCs) were purchased from ScienCell (San Diego, California, US, Cat# R6110) and cultured in smooth muscle cell medium (SMCM, ScienCell, US, Cat#: 1101) containing 5% fetal bovine serum (FBS), 100 U mL^−1^ penicillin, and 100 g mL^−1^ streptomycin. SMCs have a stable phenotype, are not prone to phenotypic transition, and can be passaged for a long time. SMCs between passages five and twelve were used in all experiments. Cells were maintained at 37 °C in a humidified incubator with an atmosphere of 5% CO_2_. For in vitro experiments, cells were cultured in 6-well plates (6 × 10^5^ cells/well; NEST, Cat#: 703001) and stimulated with 100 nM Ang II (Sigma, Cat#: A9525-50MG) or 300 μM H_2_O_2_ (Sigma-Aldrich, H1009, US) for 24 h. For inhibition of PDE4D, 500 nM rolipram (PDE4 inhibitor, Sigma-Aldrich, R6520, US) was added 0.5 h before Ang II or H_2_O_2_ treatment. For knockdown of PDE4D, PDE4D siRNA (RiboBio, siB180730051733) and control scrambled siRNA (RiboBio, siN0000001-1-5) were purchased from RiboBio. siRNA transfections were performed according to the manufacturer’s protocol. Briefly, SMCs at 80% confluence were transfected with 200 nM PDE4D siRNA duplexes in 5 μL of Oligofectamine for 48 h (Invitrogen, Carlsbad, CA, US, Cat#: 12252011). siRNA efficiency was determined by RT-PCR and western blot.

### Real-time polymerase chain reaction (RT-PCR)

Total RNA was extracted from aortic tissues or SMCs using TRIzol reagent (Invitrogen, Carlsbad, CA, Cat#: 15596018) according to the manufacturer’s protocol. Equal amounts of RNA (1000 ng) were reverse transcribed into cDNA (TianGen, KR116-02), and quantitative real-time PCR was performed in triplicate using a single-color RT-PCR detection system (Bio-Rad, Hercules, CA, US). *Pde4a*, *Pde4b*, *Pde4c*, *Pde4d*, *Il-1α*, *Il-1β*, *Il-6*, *Mrc-1*, *Arg-1*, *Cd163*, *Mmp2*, *Mmp9*, *Sm22α*, *Cnn*, *Opn*, *Col1a1*, *Col3a1*, *Fn*, *Tnf-α*, and *Ccl2* levels were normalized to the level of the housekeeping gene glyceraldehyde-3-phosphate dehydrogenase (*Gapdh*). The mRNA levels were recorded as the expression fold change compared with the control group or a single control mouse and were calculated using the 2^-ΔΔCt^ method. The RT-PCR primers are shown in Supplementary Table 2.

### Western blot analysis

Protein was extracted from aortic tissues or SMCs in RIPA lysis buffer with protease inhibitor cocktail (Roche, NJ, US) for thirty mins and centrifuged at 12,000 rpm for 10 min. The protein concentration was determined using a BCA Protein Assay Kit (Pierce, Holmdel, NJ, US, Cat#: 23225). Equal amounts of protein (25–30 μg) were separated by sodium dodecyl sulfate-polyacrylamide gel electrophoresis (SDS-PAGE; 8%, 10%, or 12% gel) and transferred onto a polyvinylidene difluoride (PVDF) membrane. The PVDF membranes were blocked with 5% (m/v) fat-free milk powder/Tris buffered saline-Tween (TBST) for 1 h and incubated overnight at 4 °C with primary antibodies against PDE4D (1:1000, Abcam, Britain, Cat#: ab171750), cleaved caspase-3 (1:1000, Cell Signaling Technology, US, Cat#: 9662S), caspase-3 (1:1000, Cell Signaling Technology, US, Cat#: 9662S), phospho-Bad (pBad, 1:1000, Thermo Fisher, US, Cat#: PAS-105022) and Bad (1:1000, Cell Signaling Technology, US, Cat#: 9292S). Immunoblotting of the housekeeping protein GAPDH (1:8000, Proteintech, US, Cat#: 60004-1-Ig) was performed to ensure equal protein loading. After three washes with TBST, the membranes were incubated with horseradish peroxidase (HRP)-labeled rabbit (1:5000, Invitrogen, Britain, Cat#: A24531) or mouse (1:5000, Invitrogen, Britain, Prod#: 04-6020) secondary antibodies for 1 h at room temperature. Immunoreactive bands were visualized with SuperSignal™ West Pico PLUS Chemiluminescent Substrate (Pierce, Cat#: 34577). Protein expression was measured by analyzing the intensities of the protein bands with Image-Pro Plus 6.0 software.

### Flow cytometry

SMC apoptosis was investigated via flow cytometry. Briefly, the digested SMC suspension was passed through a 40 μm Cell Strainer (Biologix Group, Ltd., Jiangsu, China) and stained according to the protocol of the Annexin V-FITC/PI Apoptosis Detection Kit (Dojindo, Japan, AD10). All groups of cells in the mixture were used as the gating control samples stained with only Annexin V-FITC or PI or without any dye. The data were acquired and analyzed by BD Accuri C6 flow cytometry (BD Biosciences, US). The gating strategy was to divide positive and negative events in negative, Annexin V-FITC, and PI gating control samples (Supplementary Fig. 12a–d).

### Measurement of cAMP

A cAMP Direct Fluorometric Immunoassays Kit (Abcam, ab138880) was used to measure the cAMP level according to the manufacturer’s instructions. For in vitro cAMP quantification, cells plated in sterile 96-well plates (1 × 10^5^ cells/well) were lysed with 100 μL/well of Cell Lysis Buffer. For in vivo cAMP quantification, aortic tissues were lysed in 20 μL/mg of Cell Lysis Buffer. Briefly, all samples and standards (75 μL each) mixed with 25 μL of 1x HRP-cAMP were incubated in plates at room temperature for 2 h on a plate shaker. After four washes, the plate was incubated with 100 μL/well of AbRed Working Solution for 45 min. The fluorescence was measured at Ex/Em = 540/590 nm using a Biotek Synergy™ H1 microplate reader. The standard curve was determined by regression analysis using a logistic curve-fit and prepared for every experiment independently.

### Cell proliferation assay

SMC proliferation was assayed using BeyoClick™ EdU-488 Cell Proliferation Assay Kit (Beyotime, Beijing, China) following the manufacturer’s instructions. Images were taken by a fluorescence microscope (Nikon Eclipse Ti2, Japan), and the signals were counted in three random visual fields for each sample.

### Statistical analysis

Data were statistically analyzed using GraphPad Prism 9 (GraphPad Software, LLC, San Diego, CA). Data are expressed as the mean ± SEM. The Shapiro-Wilk test was used to test for normal distribution. The Brown-Forsythe test or F test was used to test for equality of variances. Normally distributed datasets with equal variance were analyzed with the parametric unpaired Student’s *t* test for 2 independent groups and the parametric one-way ANOVA or two-way ANOVA followed by the Holm-Sidak’s post hoc test for ≥3 groups. Normally distributed datasets without equal variance were analyzed with a parametric Welch’s t test for 2 independent groups and the parametric Welch ANOVA with Dunnett’s T3 post hoc test for ≥3 groups. Where a normal distribution could not be confirmed, the nonparametric Mann-Whitney test was used for 2 independent groups. All tests were two sided. A *p* value < 0.05 was considered statistically significant. Detailed statistical analyses and plotting methods are listed in Supplementary Table 3.

## Results

### PDE4D expression is upregulated in human and mouse AAA tissues

**Fig. 1:**
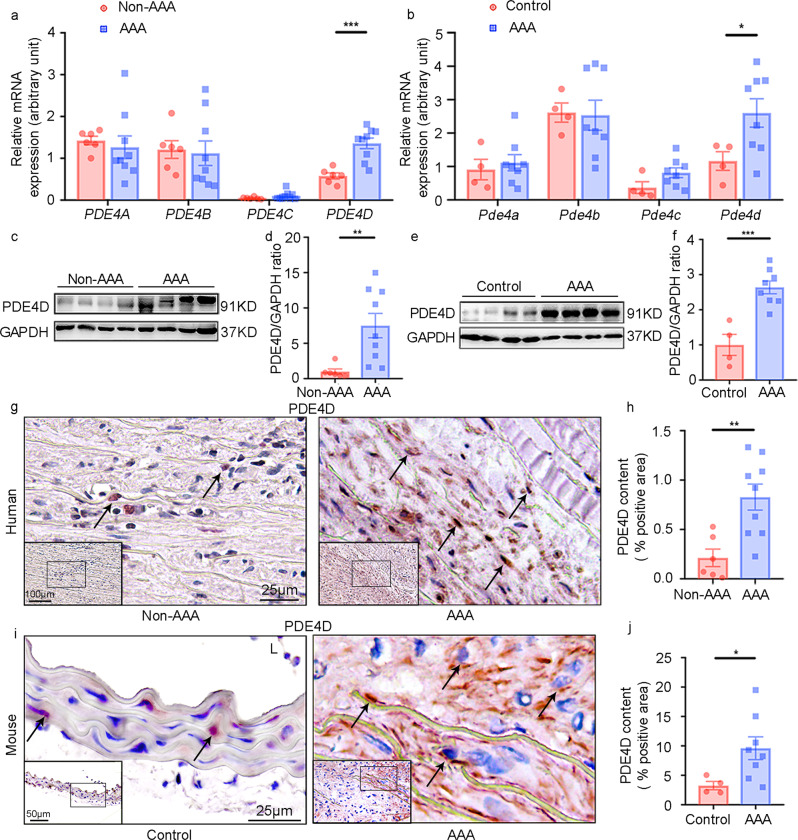
Phosphodiesterase (PDE) 4D expression is upregulated in human and mouse AAA tissues. **a** RT-PCR quantification of *PDE4A*, *PDE4B*, *PDE4C,* and *PDE4D* mRNA levels (fold change versus one of the non-AAA subjects) in human tissues. ***p < 0.001, Welch’s *t* test for PDE4A, Mann–Whitney test for PDE4B/PDE4C, unpaired Student’s *t* test for PDE4D, mean ± SEM, non-AAA (*n* = 6), AAA (*n* = 9). Human AAA tissues were collected from AAA patients during surgery, and non-AAA tissues were collected from corresponding tissues of deceased donors with no detectable vascular disease. **b** RT-PCR quantification of *Pde4a*, *Pde4b*, *Pde4c*, and *Pde4d* mRNA (fold change versus one of the control subjects) in mouse AAA tissues from the *Apoe*^*−/−*^ mice treated with 1000 ng kg^−1^ min^−1^ angiotensin II (Ang II) and a high-fat diet (HFD) and control mouse abdominal aortas from the *Apoe*^*−/−*^ mice treated with saline. *p < 0.05, unpaired Student’s *t* test, mean ± SEM, controls (*n* = 4), AAA (*n* = 8). Mouse AAA aortas were dissected at the maximal suprarenal outer aortic diameter, and controls were collected from the corresponding suprarenal abdominal aortas of control mice. (**c**, **d**). Representative western blots and quantification of PDE4D protein levels in human AAA and non-AAA tissues normalized to glyceraldehyde-3-phosphate dehydrogenase (GAPDH) protein (fold change versus non-AAA subjects). **p < 0.01, Mann-Whitney test, mean ± SEM, non-AAA (*n* = 6), AAA (*n* = 9). (**e**, **f**) Representative western blots and quantification of PDE4D protein levels in mouse AAA and control tissues normalized to GAPDH protein (fold change versus control subjects). ***p < 0.001, unpaired Student’s *t* test, mean ± SEM, controls (*n* = 4), AAA (*n* = 8). (**g**, **h**) Representative images of immunohistochemistry staining of PDE4D (**g**) and quantification of PDE4D staining intensity per medial area (**h**) in human tissues. A total of five random images per human in each group were selected for statistical analysis of the ratio of positive areas to vascular areas. L lumen. **p < 0.01, unpaired Student’s *t* test, mean ± SEM, non-AAA (*n* = 6), AAA (*n* = 9). (**i**, **j**) Representative images of immunohistochemistry analysis of PDE4D (**i**) and quantification of PDE4D staining intensity per medial area (**j**) in mouse tissues. A total of five random images per mouse in each group were selected for statistical analysis of the ratio of positive areas to vascular areas. L lumen. *p < 0.05, Welch’s *t* test, mean ± SEM, controls (*n* = 4), AAA (*n* = 8).

To explore PDE4 expression during AAA formation, we assessed the mRNA levels of individual PDE4 family isoforms (PDE4A-D) in both human and mouse AAA tissues. Human AAA tissues were collected from AAA patients via surgery, and control non-AAA tissues were corresponding tissues collected from the bodies of deceased donors with no detectable vascular disease (Supplementary Table 1). Mouse AAA was induced by a chronic infusion of Ang II (1000 ng kg^−1^ min^−1^) for twenty-eight days via osmotic minipumps in *Apoe*^*−/*−^ male mice fed a high-fat diet (HFD): a well-established murine AAA model^20^. Control mice received vehicle infusion. Mouse AAA tissues and corresponding normal aortic tissues were dissected from the suprarenal aortic regions of the mice with AAA and the control mice, respectively. Among the four PDE4 isozymes, only *PDE4D* expression levels were significantly increased in human AAA compared to non-AAA tissues (Fig. 1a). Similar observations were obtained for mouse AAA tissues (Fig. 1b). Henceforth, we focused on PDE4D in this study. Consistently, we found that the PDE4D protein levels were significantly higher in human (Fig. 1c, d) and mouse AAA tissues (Fig. 1e, f). Immunohistochemical staining further confirmed the PDE4D protein increase in human (Fig. 1g, h) and mouse AAA lesions (Fig. 1i, j). PDE4D antibody specificity was supported by negative controls performed in mouse and human tissues (Supplementary Fig. 1a–g). These results demonstrated an upregulation of PDE4D expression in AAA.

**Fig. 2:**
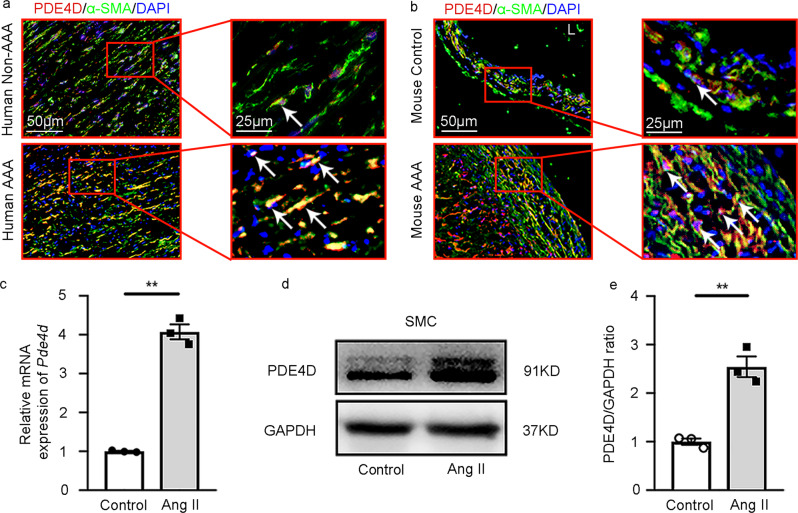
PDE4D is primarily expressed in smooth muscle cells of AAA tissues. **a** Immunofluorescence staining of PDE4D and α smooth muscle actin (α-SMA, smooth muscle cell marker) in human non-AAA sections and AAA tissues (6 μm). We obtained similar results from at least three different sets of human tissues in separate experiments. PDE4D: red, α-SMA: green. **b** Immunofluorescence staining of PDE4D and α-SMA in mouse control sections and AAA tissues (6 μm). We obtained similar results from at least three different sets of mouse samples in separate experiments. PDE4D: red, α-SMA: green. L: lumen. **c** RT-PCR analysis of *Pde4d* expression in rat aortic SMCs treated with Ang II (100 nM, 24 h). **p < 0.0001, Welch’s *t* test, mean ± SEM, *n* = 3 separate experiments. **d** Representative immunoblot analysis of PDE4D protein expression in SMCs treated with Ang II (100 nM, 24 h). **e** Quantification of PDE4D protein expression by immunoblotting in (**d**) normalized to GAPDH protein (fold change versus control). **p < 0.01, unpaired Student’s *t* test, mean ± SEM, *n* = 3 separate experiments.

PDE4D expression was largely observed in the medial areas where SMCs resided (Fig. 1g–j), suggesting the expression PDE4D in SMCs. To further confirm the expression of PDE4D in SMCs, we performed double immunofluorescence staining of PDE4D and α-smooth muscle actin (α-SMA), a marker for differentiated contractile and dedifferentiated synthetic SMCs (or myofibroblasts). We observed prominent PDE4D staining in α-SMA-positive cells in human (Fig. 2a and Supplementary Fig. 2a) and mouse AAA lesions (Fig. 2b and Supplementary Fig. 2b). Consistently, in cultured rat aorta SMCs, Ang II also increased PDE4D mRNA and protein levels (Fig. 2c–e). Therefore, in this study, we focused on the role of SMC PDE4D in SMC pathogenesis and AAA development.

### SMC-specific *Pde4d* deficiency decreases Ang II-induced AAA formation

To explore the role of SMC PDE4D in AAA development, we used SMC-specific *Pde4d* knockout mice on an *Apoe*^*−/−*^ background (*Apoe*^*−/−*^*Pde4d*^*SMC−/−*^). We generated *Pde4d*-floxed mice (*Pde4d*^*flox/flox*^) carrying floxed exon 10 of the *Pde4d* gene through homologous recombination via CRISPR/Cas9 technology. We then crossed *Pde4d*^*flox/flox*^ mice with *Apoe*^*−/−*^ mice and subsequently crossed *Apoe*^*−/−*^*Pde4d*^*flox/flox*^ mice with *Tagln*-Cre mice (Supplementary Fig. 3a). The resultant *Apoe*^*−/−*^*Pde4d*^*flox/flox*^ and *Apoe*^*−/−*^*Pde4d*^*SMC−/−*^ littermates served as experimental mouse groups. RT-PCR (Supplementary Fig. 3b), immunoblotting (Supplementary Fig. 3c), and immunostaining (Supplementary Fig. 1d–g) confirmed PDE4D depletion in mouse aortic SMCs. We also observed no significant alterations in other PDE4 isozymes between *Apoe*^*−/−*^*Pde4d*^*flox/flox*^ and *Apoe*^*−/−*^*Pde4d*^*SMC−/*−^ aortic tissues (Supplementary Fig. 3b).

**Fig. 3:**
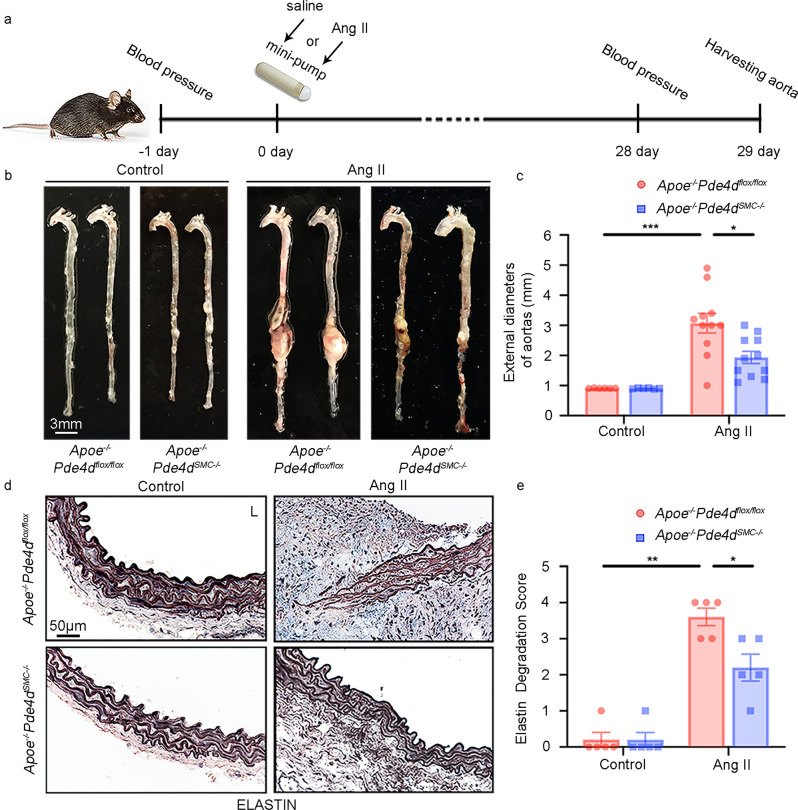
Effect of SMC-specific *Pde4d* deficiency on Ang II-induced AAA in mice. AAAs were induced by Ang II infusion (1000 ng kg^−1^ min^−1^) and administered an HFD for 28 days, and controls were infused with saline. Mouse AAA aortas were dissected at the maximal suprarenal outer aortic diameter, and controls were collected from corresponding suprarenal abdominal aortas of the control mice. **a** Diagrammatic drawing of the mouse model of Ang II-induced AAA. *Apoe*^*−/−*^*Pde4d*^*flox/flox*^ (*n* = 6) and *Apoe*^*−/−*^*Pde4d*^*SMC−/−*^ (*n* = 6) with saline infusion and *Apoe*^*−/−*^*Pde4d*^*flox/flox*^ (*n* = 11) and *Apoe*^*−/−*^*Pde4d*^*SMC−/−*^ (*n* = 11) with Ang II infusion. **b** Representative images of the entire aortas in the indicated groups. **c** Quantification of the maximal external diameter (mm) of the abdominal aorta measured by two different investigators using a stereoscope. *p < 0.05, ***p < 0.001, Welch ANOVA with Dunnett’s T3 post hoc test, mean ± SEM, *Apoe*^*−/−*^*Pde4d*^*flox/flox*^ (*n* = 6) and *Apoe*^*−/−*^*Pde4d*^*SMC−/−*^ (*n* = 6) with saline infusion, *Apoe*^*−/−*^*Pde4d*^*flox/flox*^ (*n* = 11) and *Apoe*^*−/−*^*Pde4d*^*SMC−/−*^ (*n* = 11) with Ang II infusion. **d** Representative images of elastin staining of the mouse arterial wall. L: lumen. **e** Quantification of the elastin degradation score from the four indicated groups in (**d**). Elastin staining is indicated by the darkest color. *p < 0.05, **p < 0.01, Mann–Whitney test, mean ± SEM, *Apoe*^*−/−*^*Pde4d*^*flox/flox*^ (*n* = 5) and *Apoe*^*−/−*^*Pde4d*^*SMC−/−*^ (*n* = 5) with saline infusion, *Apoe*^*−/−*^*Pde4d*^*flox/flox*^ (*n* = 5) and *Apoe*^*−/−*^*Pde4d*^*SMC−/−*^ (*n* = 5) with Ang II infusion.

AAA was induced by Ang II infusion (1000 ng kg^−1^ min^−1^) for twenty-eight days along with an HFD, while the controls were infused with saline (Fig. 3a). The aortas were excised, and the maximal external diameter of the abdominal aorta was measured by two different investigators via stereoscopy at the time of necropsy. AAA was defined as a 50% dilation in the diameter of the external abdominal aorta compared with the normal mouse abdominal aorta. Under Ang II infusion, AAA development was significantly attenuated in the *Apoe*^*−/−*^*Pde4d*^*SMC−/−*^ mice compared to the *Apoe*^*−/−*^*Pde4d*^*flox/flox*^ mice, as illustrated by the aortic morphology (Fig. 3b). The average external diameter of the abdominal aortas was significantly smaller in the *Apoe*^*−/−*^*Pde4d*^*SMC−/−*^ Ang II group than in the *Apoe*^*−/−*^*Pde4d*^*flox/flox*^ Ang II group (1.936 ± 0.203 mm vs. 3.073 ± 0.327 mm; Fig. 3c). Supplementary Fig. 4a includes images of all AAA samples shown in Fig. 3b, c. In addition, the fragmentation and degradation of the aortic elastic lamina—a characteristic feature of AAA—was evaluated. Verhoeff Van Gieson staining for elastin and semiquantitative analysis revealed more profound elastin degradation in the *Apoe*^*−/−*^*Pde4d*^*flox/flox*^ Ang II mice than in the *Apoe*^*−/−*^*Pde4d*^*SMC−/−*^ Ang II mice (Fig. 3d, e). *Mmp2* and *Mmp9* mRNA levels were both upregulated in the *Apoe*^*−/−*^*Pde4d*^*flox/flox*^ Ang II group, but only *Mmp2* expression was rescued in *Apoe*^*−/−*^*Pde4d*^*SMC−/−*^ mouse aortas (Supplementary Fig. 5a, b). In addition, aortic cAMP levels were decreased in the Ang II-induced AAA mice compared with the saline-treated mice, and SMC-specific knockout of *Pde4d* reversed the cAMP levels in AAA tissues (Supplementary Fig. 6a). These results suggest that PDE4D deficiency in SMCs enhances the stability of the aortic wall and decreases Ang II-induced AAA formation.

Additionally, Ang II infusion increased systolic and diastolic blood pressure (BP) in the *Apoe*^*−/−*^*Pde4d*^*flox/flox*^ mice, both of which were significantly lower in the *Apoe*^*−/−*^*Pde4d*^*SMC−/−*^ Ang II mice (Supplementary Fig. 4b, c). Based on a previous report by Daugherty et al., AAA occurred in the *Apoe*^*−/−*^ mice infused with Ang II (1000 ng kg^−1^ min^−1^) for twenty-eight days, accompanied by increased BP. Hydralazine administration (an antihypertensive drug) lowered systolic BP in the Ang II-infused *Apoe*^*−/−*^ mice, while hydralazine did not prevent AAA formation^33^. Therefore, it is generally believed that Ang II infusion-induced AAA formation is independent of BP elevation. Thus, the protective effect of *Pde4d*^*SMC−/−*^ against AAA development is unlikely to result from a BP reduction.

### The PDE4 inhibitor rolipram attenuates Ang II-induced AAA formation

**Fig. 4:**
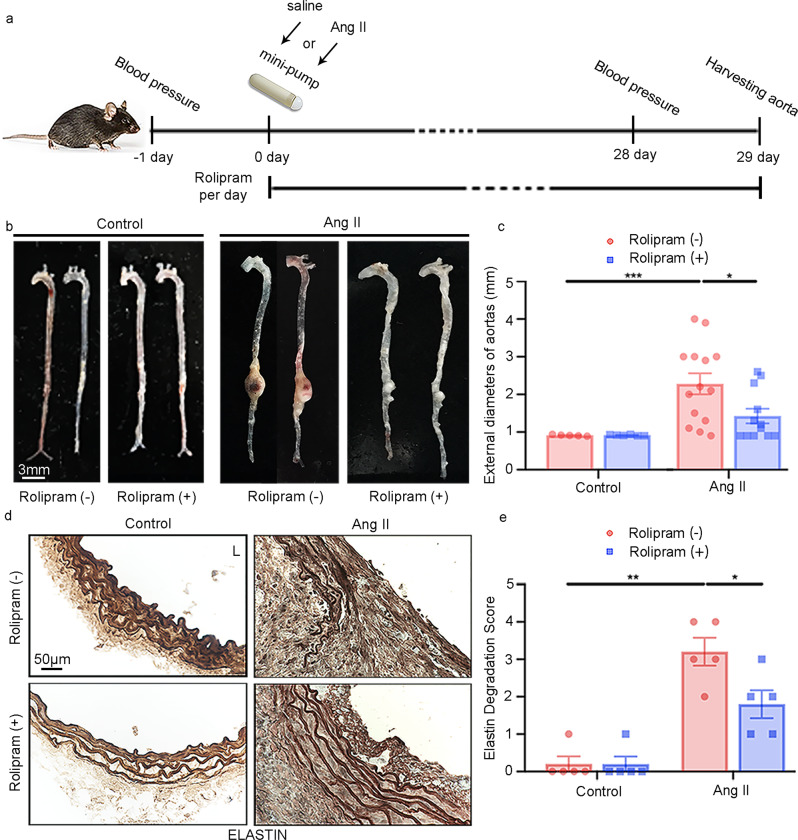
Effect of rolipram on Ang II-induced AAA in mice. AAAs were induced by Ang II infusion (1000 ng kg^−1^ min^−1^) and an HFD for 28 days, and controls were infused with saline. Rolipram (3 mg kg^−1^ d^−1^) was orally administered daily for 28 days. Mouse AAA aortas were dissected at the maximal suprarenal outer aortic diameter, and controls were collected from corresponding suprarenal abdominal aortas of the control mice. **a** Schematic diagram of the mouse model of Ang II-induced AAA treated with vehicle or rolipram. Vehicle (*n* = 5) and rolipram (*n* = 6) with saline infusion, vehicle (*n* = 14) and rolipram (*n* = 12) with Ang II infusion. **b** Representative images of entire aortas of the *Apoe*^*−/−*^ mice treated with vehicle or rolipram. **c** Quantification of the maximal external diameter (mm) of the abdominal aorta measured by two different investigators using a stereoscope. *p < 0.05, ***p < 0.001, Welch’s *t* test between the rolipram (−) control and rolipram (−) Ang II groups, Mann–Whitney test between the rolipram (−) Ang II and rolipram (+) Ang II groups, mean ± SEM, vehicle (*n* = 5) and rolipram (*n* = 6) with saline infusion, vehicle (*n* = 14) and rolipram (*n* = 12) with Ang II infusion. **d** Representative images of elastin staining of the mouse arterial wall. L: lumen. **e** Quantification of the elastin degradation score from the four indicated groups in (**d**). Elastin staining is indicated by the darkest color. *p < 0.05, **p < 0.01, Mann–Whitney test between the rolipram (−) control and rolipram (−) Ang II groups, unpaired Student’s *t* test between the rolipram (−) Ang II and rolipram (+) Ang II groups, mean ± SEM, vehicle (*n* = 5) and rolipram (*n* = 5) with saline infusion, vehicle (*n* = 5) and rolipram (*n* = 5) with Ang II infusion.

Next, we sought to determine the pharmacological effect of a PDE4 inhibitor on AAA. We selected the pan-PDE4 inhibitor rolipram, which was used clinically for neuroinflammation in the early 1990s^19^. Male C57BL/6J *Apoe*^*−/−*^ mice at the age of eight weeks were infused with Ang II or saline and fed an HFD for four weeks. Rolipram (3 mg kg^−1^ d^−1^) or vehicle (7% ethyl alcohol) was given daily via gavage for four weeks (Fig. 4a). Rolipram (+) treatment significantly reduced the AAA size compared with rolipram (−) vehicle treatment (Fig. 4b). The average external diameter of the abdominal aorta was smaller in the rolipram (+) Ang II group (1.425 ± 0.192 mm) than in the rolipram (−) Ang II group (2.279 ± 0.279 mm; Fig. 4c). Supplementary Fig. 7a includes images of all AAA samples shown in Fig. 4b, c. Moreover, elastin fragmentation in the aortic wall was significantly reduced in the rolipram (+) Ang II mice compared with the rolipram (−) Ang II mice (Fig. 4d, e). Consistently, rolipram also attenuated *Mmp2*, but not *Mmp9*, in aortic tissues induced by Ang II (Supplementary Fig. 5c, d). As expected, cAMP levels in the AAA mice were lower than those in the saline-treated mice, and rolipram reversed cAMP levels in AAA tissues (Supplementary Fig. 6b). These results demonstrate a significant pharmacological impact of rolipram in preventing AAA development in mice. Similar to the results in *Pde4d*^*SMC−/−*^ mice, rolipram also reduced systolic and diastolic BP in AAA mice (Supplementary Fig. 7b, c).

### PDE4D promotes SMC apoptosis in vitro and in vivo

**Fig. 5:**
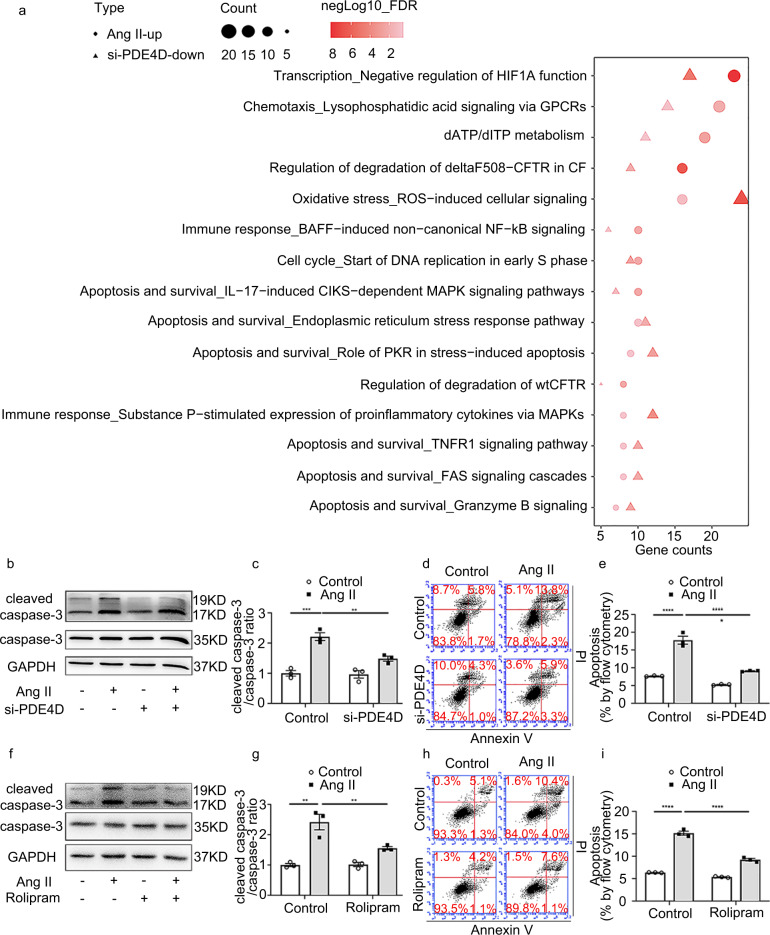
PDE4D regulates SMC apoptosis induced by Ang II. **a** The top 15 MetaCore pathways (based on their minimum FDR) enriched for both the upregulated genes in Ang II (100 nM, 24 h)-stimulated rat aortic SMCs and the downregulated genes in PDE4D siRNA (200 nM, 48 h)-treated SMCs. **b** Representative immunoblot analysis of caspase-3 and cleaved caspase-3 expression in the SMCs treated with Ang II (100 nM, 24 h) and/or PDE4D siRNA (200 nM, 48 h) as indicated. **c** Quantification of cleaved caspase-3 protein expression by immunoblotting in (**b**) normalized to caspase-3 protein (fold change versus control). **p < 0.01, ***p < 0.001, two-way ANOVA with Holm-Sidak’s post hoc test, mean ± SEM, *n* = 3 separate experiments. **d** Flow cytometric analysis of annexin V/propidium iodide (PI)-stained SMCs treated with or without Ang II (100 nM, 24 h)/PDE4D siRNA (200 nM, 48 h) as indicated. **e** Quantification of the total (early and late) apoptosis rates of annexin V/PI-stained SMCs treated with or without Ang II (100 nM, 24 h)/PDE4D siRNA (200 nM, 48 h) as indicated. ****p < 0.0001, two-way ANOVA with Holm–Sidak’s post hoc test, mean ± SEM, *n* = 3 separate experiments. **f** Immunoblot analysis of caspase-3 and cleaved caspase-3 expression in the SMCs treated with or without Ang II (100 nM, 24 h)/rolipram (500 nM, 24.5 h). **g** Quantification of cleaved caspase-3 protein expression by immunoblotting in (**f**) normalized to caspase-3 protein (fold change versus control). **p < 0.01, two-way ANOVA with Holm–Sidak’s post hoc test, mean ± SEM, *n* = 3 separate experiments. **h** Flow cytometric analysis of annexin V/PI-stained SMCs treated with or without Ang II (100 nM, 24 h)/rolipram (500 nM, 24.5 h) as indicated. **i** Quantification of the total (early and late) apoptosis rates of annexin V/PI-stained SMCs treated with or without Ang II (100 nM, 24 h)/rolipram (500 nM, 24.5 h) as indicated. ****p < 0.0001, two-way ANOVA with Holm-Sidak’s post hoc test, mean ± SEM, *n* = 3 separate experiments.

To determine the underlying mechanism by which PDE4D participates in AAA formation, we performed bulk RNA-seq to identify PDE4D-regulated genes in rat aortic SMCs treated with PDE4D-specific or scramble control siRNA. RNA-seq revealed 1,848 genes upregulated in SMCs stimulated with Ang II (100 nM, 24 h; Supplementary Fig. 8a, c, d) and 1,551 genes downregulated in SMCs by PDE4D siRNA (Supplementary Fig. 8b, c) (for the full list, refer to Supplementary Dataset 1). Among these genes, 235 were upregulated by Ang II, and the upregulation was reversed by si-PDE4D (Supplementary Fig. 8c). We next performed MetaCore pathway enrichment analysis of these 235 genes (for the full list, refer to Supplementary Dataset 1). We focused on the top 15 pathways shared by the two groups (control vs. Ang II and si-scramble vs. si-PDE4D) based on their minimum false discovery rate (FDR). Among these pathways, apoptosis-related pathways were predominant, which is consistent with the important role of SMC apoptosis in AAA pathogenesis (Fig. 5a).

We next determined the role of PDE4D in regulating SMC apoptosis using PDE4D siRNA to knockdown PDE4D expression or rolipram to inhibit PDE4 activity in SMCs (Supplementary Fig. 9a–c). We found that Ang II increased caspase-3 cleavage, a key event in apoptosis^34^. PDE4D siRNA substantially attenuated Ang II-induced caspase-3 cleavage (Fig. 5b, c). Similarly, PDE4D siRNA also reduced caspase-3 cleavage induced by H_2_O_2_ as a reactive oxidative stress (ROS) mediator known to be important in AAA (Supplementary Fig. 10a, b). We also assessed SMC apoptosis via Annexin V/propidium iodide (PI) staining and flow cytometry (Fig. 5d and Supplementary Fig. 10c). We found that SMC apoptosis induced by Ang II or H_2_O_2_ was significantly suppressed by PDE4D siRNA (Fig. 5e and Supplementary Fig. 10d). As with PDE4D siRNA, rolipram also reduced cleaved caspase-3 levels in SMCs treated with Ang II (Fig. 5f, g) or H_2_O_2_ (Supplementary Fig. 11a, b) and decreased SMC apoptosis induced by Ang II (Fig. 5h, i) or H_2_O_2_ (Supplementary Fig. 11c, d). The gating strategy of flow cytometry was to divide positive and negative events in negative, Annexin V-FITC, and PI gating control samples (Supplementary Fig. 12a–d). These results support a proapoptotic function of PDE4D in SMCs in vitro.

**Fig. 6:**
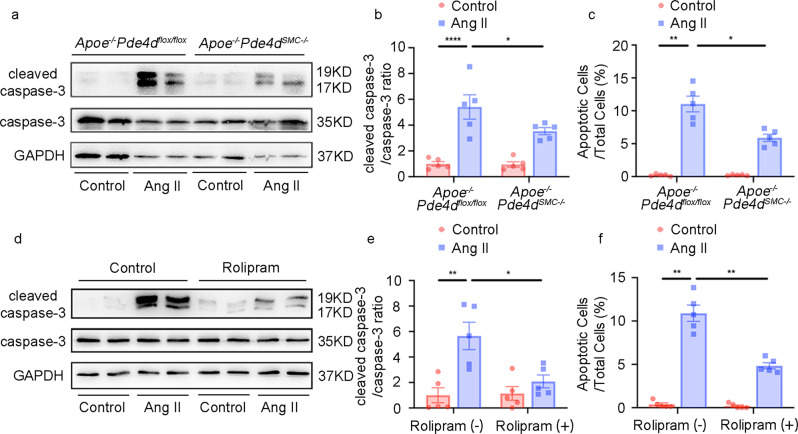
PDE4D knockout and rolipram alleviates apoptosis in vivo. **a** Representative immunoblot analysis of caspase-3 and cleaved caspase-3 expression in the *Apoe*^*−/−*^*Pde4d*^*flox/flox*^ and *Apoe*^*−/−*^*Pde4d*^*SMC−/−*^ mice with or without 1000 ng kg^−1^ min^−1^ Ang II treatment and a high-fat diet (HFD) for 28 days. **b** Quantification of cleaved caspase-3 protein expression by immunoblotting in (**a**) normalized to caspase-3 protein (fold change versus control). *p < 0.05, ****p < 0.0001, two-way ANOVA with Holm-Sidak’s post hoc test, mean ± SEM, *Apoe*^*−/−*^*Pde4d*^*flox/flox*^ (*n* = 5) and *Apoe*^*−/−*^*Pde4d*^*SMC−/−*^ (*n* = 5) with saline infusion, *Apoe*^*−/−*^*Pde4d*^*flox/flox*^ (*n* = 5) and *Apoe*^*−/−*^*Pde4d*^*SMC−/−*^ (*n* = 5) with Ang II infusion. **c** Quantification of cell apoptosis rates with TUNEL staining in Supplementary Fig. 13. A total of five random images per mouse were selected for TUNEL staining statistical analysis. Apoptotic cells examined by TUNEL staining were quantified as the ratio of apoptotic cells to total cells in five fields per mouse. *p < 0.05, **p < 0.01, Welch ANOVA with Dunnett’s T3 post hoc test, mean ± SEM, *Apoe*^*−/−*^*Pde4d*^*flox/flox*^ (*n* = 5) and *Apoe*^*−/−*^*Pde4d*^*SMC−/−*^ (*n* = 5) with saline infusion, *Apoe*^*−/−*^*Pde4d*^*flox/flox*^ (*n* = 5) and *Apoe*^*−/−*^*Pde4d*^*SMC−/−*^ (*n* = 5) with Ang II infusion. (d) Representative immunoblot analysis of caspase-3 and cleaved caspase-3 expression in the *Apoe*^*−/−*^ mice with or without Ang II/rolipram treatment. **e** Quantification of cleaved caspase-3 protein expression by immunoblotting in (**d**) normalized to caspase-3 protein (fold change versus control). *p < 0.05, **p < 0.01, two-way ANOVA with Holm-Sidak’s post hoc test, mean ± SEM, vehicle (*n* = 5) and rolipram (*n* = 5) with saline infusion, vehicle (*n* = 5) and rolipram (*n* = 5) with Ang II infusion. **f** Quantification of cell apoptosis rates with TUNEL staining in Supplementary Fig. 14. A total of 5 random images per mouse were selected for TUNEL staining statistical analysis. Apoptotic cells examined by TUNEL staining were quantified as the ratio of apoptotic cells to total cells in five fields per mouse. **p < 0.01, Mann–Whitney test, mean ± SEM, vehicle (*n* = 5) and rolipram (*n* = 5) with saline infusion, vehicle (*n* = 5) and rolipram (*n* = 5) with Ang II infusion.

Consistent with the in vitro findings, cleaved caspase-3 in Ang II-induced AAA tissues was significantly reduced in the *Apoe*^*−/−*^*Pde4d*^*SMC−/−*^ mice (Fig. 6a, b). Apoptotic cell numbers detected by TUNEL staining were also decreased in the *Apoe*^*−/−*^*Pde4d*^*SMC−/−*^ Ang II mouse tissues compared to the *Apoe*^*−/−*^*Pde4d*^*flox/flox*^ Ang II group (Fig. 6c and Supplementary Fig. 13). Accordingly, rolipram decreased cleaved caspase-3 levels and TUNEL-positive cells in AAA tissues (Fig. 6d–f and Supplementary Fig. 14). Supplementary Fig. 15 shows the negative staining of TUNEL. These results suggest that PDE4D promotes SMC apoptosis in AAA.

### PDE4D antagonizes PKA-mediated phosphorylation of Bad and induces cleaved caspase-3 and apoptosis in SMCs

**Fig. 7:**
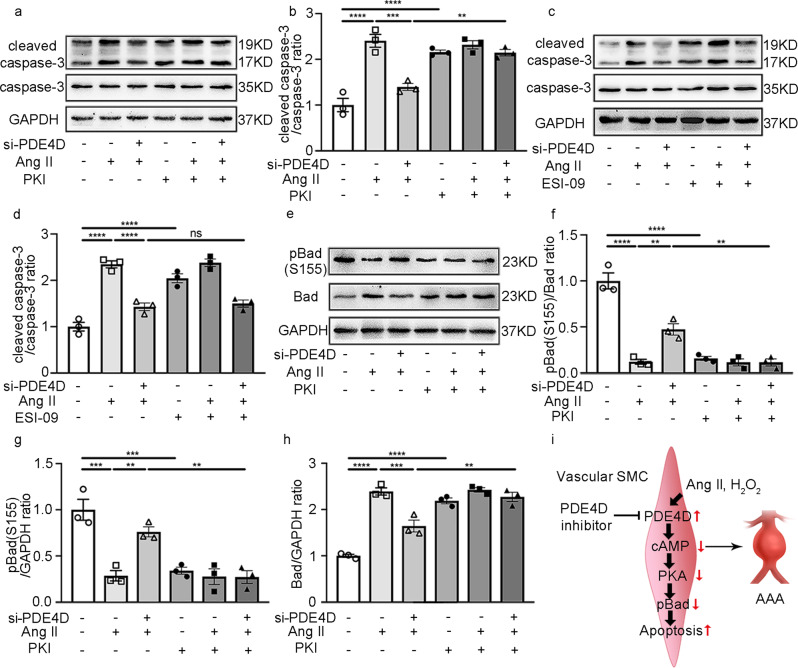
PDE4D promotes apoptosis through Bad phosphorylation regulated by PKA. **a** Representative immunoblot analysis of caspase-3 and cleaved caspase-3 expression in the SMCs treated with PKI (PKA inhibitor, 10 μM, 60 min) and/or Ang II (100 nM, 24 h) and/or PDE4D siRNA (200 nM, 48 h) as indicated. **b** Quantification of cleaved caspase-3 protein expression by immunoblotting in (**a**) normalized to caspase-3 protein (fold change versus control). **p < 0.01, ***p < 0.001, ****p < 0.0001, one-way ANOVA with Holm-Sidak’s post hoc test, mean ± SEM, *n* = 3 separate experiments. **c** Immunoblot analysis of caspase-3 and cleaved caspase-3 expression in the SMCs treated with ESI-09 (Epac inhibitor, 100 μM, 60 min) and/or Ang II (100 nM, 24 h) and/or PDE4D siRNA (200 nM, 48 h) as indicated. **d** Quantification of cleaved caspase-3 protein expression by immunoblotting in (c) normalized to caspase-3 protein (fold change versus control). ****p < 0.0001, ns: no significant difference, one-way ANOVA with Holm–Sidak’s post hoc test, mean ± SEM, *n* = 3 separate experiments. **e** Representative immunoblot analysis of phospho-Bad (pBad) and Bad expression in the SMCs treated with PKI (PKA inhibitor, 10 μM, 60 min) and/or Ang II (100 nM, 24 h) and/or PDE4D siRNA (200 nM, 48 h) as indicated. **f** Quantification of pBad expression by immunoblotting in (**e**) normalized to Bad protein (fold change versus control). **p < 0.01, ****p < 0.0001, one-way ANOVA with Holm-Sidak’s post hoc test, mean ± SEM, *n* = 3 separate experiments. **g** Quantification of pBad expression by immunoblotting in (**e**) normalized to GAPDH protein (fold change versus control). **p < 0.01, ***p < 0.001, one-way ANOVA with Holm–Sidak’s post hoc test, mean ± SEM, *n* = 3 separate experiments. **h** Quantification of Bad expression by immunoblotting in (**e**) normalized to GAPDH protein (fold change versus control). **p < 0.01, ***p < 0.001, ****p < 0.0001, one-way ANOVA with Holm-Sidak’s post hoc test, mean ± SEM, *n* = 3 separate experiments. **i** PDE4D expression was significantly increased in Ang II-induced AAA, aggravating SMC apoptosis, which promoted pBad via the cAMP-PKA axis.

PDE4 family members are cAMP-hydrolyzing enzymes. We examined the levels of cAMP in vitro using a cAMP kit. As expected, Ang II reduced SMC cAMP and PDE4D siRNA, and rolipram upregulated cAMP levels in vitro (Supplementary Fig. 6c, d). cAMP activates cAMP-dependent protein kinase A (PKA) or the exchange protein directly activated by cAMP (Epac)^35^. To identify whether PDE4D regulates apoptosis by relying on the PKA and/or Epac pathways, we used PKA- or Epac-selective inhibitors, including PKI (PKA inhibitor) and ESI-09 (Epac inhibitor). We found that PDE4D siRNA attenuated the apoptosis marker cleaved caspase-3 in SMCs induced by Ang II; however, the inhibitory effect of PDE4D siRNA on cleaved caspase-3 was blocked by PKI (Fig. 7a, b) but not ESI-09 (Fig. 7c, d). These data suggest that PDE4D regulates SMC apoptosis in a cAMP-PKA signaling-dependent manner.

Based on our transcriptome sequencing results, the BCL2 antagonist of cell death (Bad) was highlighted in several apoptosis-related pathways (Supplementary Fig. 16). Bad is an important protein involved in apoptosis. Bad can be phosphorylated by PKA at Ser155^36^. This Bad phosphorylation (pBad) causes Bad inactivation and thus inhibits apoptosis^37^. We therefore investigated the role of PDE4D in Bad phosphorylation. We found that Ang II reduced Bad phosphorylation, which was reversed by PDE4D siRNA (Fig. 7e–h). Interestingly, the effect of PDE4D siRNA on increasing Bad phosphorylation was largely blocked in the presence of PKI (Fig. 7e–h), suggesting that PDE4D negatively regulates the PKA phosphorylation of Bad.

## Discussion

In this study, we reported the role and regulatory mechanism of PDE4D in SMC apoptosis and AAA formation (Fig. 7i). We showed that PDE4D played an essential role in SMC apoptosis, at least partially by attenuating PKA-mediated phosphorylation and inactivation of the proapoptotic molecule Bad. We demonstrated PDE4D upregulation in SMCs from human and mouse AAA lesions through multiple approaches, including RT-PCR, western blotting, and immunostaining. To perform in vivo evaluation of the role of SMC PDE4D in AAA formation, we generated SMC-specific *Pde4d* knockout mice on an *Apoe*^*−/−*^ genetic background and tested them in a well-established AAA mouse model. We demonstrated a causative role of SMC PDE4D in aortic wall degeneration and AAA development. Moreover, we identified a protective effect of the PDE4 inhibitor rolipram against vascular degeneration and AAA development. Rolipram is a pan-PDE4 inhibitor that targets all four PDE4 members^38^. While possessing a history of clinical use, it has been shown to induce nausea and emesis in humans^39^, likely due to its blood-brain barrier penetration and inhibition of PDE4D in the brain chemoreceptor trigger zone of vomiting^19,40,41^. Thus, developing potential PDE4 inhibitors that are peripherally restricted will be beneficial for treating peripheral diseases.

In addition to PDE4 in SMCs, a number of different cell types in AAA tissues may also express PDE4, including endothelial cells, adventitial fibroblasts, and macrophages. However, these different cell types may primarily express distinct PDE4 isozyme(s), among 4A, 4B, 4C, and 4D. It has been shown previously that PDE4B is highly expressed in inflammatory cells and contributes significantly to inflammation^42^. The role of PDE4B in inflammation leads to the development of the PDE4 inhibitor roflumilast for the treatment of COPD. Previous studies have also shown that PDE4D is the dominant PDE4 isozyme expressed in rodent or human arterial VSMCs^43,44^. Because we found that PDE4D is most significantly upregulated in AAA tissues, particularly in SMCs, we thus focused on the role of PDE4D in SMC and AAA development in the current study using SMC-specific PDE4D deficiency animal models. Although our data demonstrated a critical role of SMC-derived PDE4D in AAA, we cannot exclude the possible contributions of PDE4D in other cell types to AAA. We also cannot exclude the possible contribution of PDE4B in immune cells to AAA, particularly in the rolipram treatment model, given an important role of inflammation in Ang II-induced AAA. We indeed found that the pan-PDE4 inhibitor rolipram elicited more profound effects on suppressing AAA than SMC-specific PDE4D deficiency (aortic diameters, 1.936 ± 0.203 mm vs. 1.425 ± 0.192 mm). These results suggest that inhibiting other PDE4 isozymes and/or PDE4D in other cell types may contribute to the protective effects of rolipram. It will be of great future interest to elucidate the contributions of distinct PDE4 isozymes in differing cell types to AAA development, given that AAA is a multifactorial disease involving numerous cell types.

In our current study, we focused on the contribution of SMCs in AAA using SMC (Tagln-Cre)-specific *Pde4d* KO mice. Although Tagln (also called smooth muscle 22α) is expressed on SMCs in cardiac, smooth, and skeletal muscle, Tagln mediates Cre expression at a high level in vascular SMCs. To exclude the effect of Tagln in cardiac myocytes, we indicated that the structure of heart tissues did not change between the *Apoe*^*−/−*^*Pde4d*^*flox/flox*^ and *Apoe*^*−/−*^*Pde4d*^*SMC−/−*^ mice (Supplementary Fig. 3d). Furthermore, comparing the *Pde4d*^*SMC−/−*^ mice and the mice treated with the pan-PDE4 inhibitor rolipram, we found that rolipram elicited more profound effects on suppressing AAA than that in the *Pde4d*^*SMC−/−*^ mice (aortic diameters, 1.425 ± 0.192 mm vs. 1.936 ± 0.203 mm). These results suggest that inhibiting other PDE4 isozymes and/or PDE4D in other cell types may contribute to the protective effects of rolipram. It will be of great future interest to elucidate the contributions of distinct PDE4 isozymes in differing cell types to AAA development, given that AAA is a multifactorial disease involving numerous cell types.

The roles of cAMP signaling in vascular SMC apoptosis are controversial. Some studies have shown the antiapoptotic effect of cAMP in vascular SMCs. For example, it has been shown that rat aortic SMC apoptosis is attenuated by the activation of cAMP/PKA signaling through the beta-adrenergic receptor (β-AR) agonist adenosine or the PDE4 inhibitor Ro-201724^45^. Some other studies reported a proapoptotic effect of cAMP in vascular SMCs. For example, human vascular SMC apoptosis was promoted by the nonselective elevation of intracellular cAMP levels through 8-bromo-cAMP and forskolin or increasing PDE3-regulated cAMP through cilostazol^46^. In the current study, we provided solid evidence and demonstrated that rat aortic SMC apoptosis is attenuated by stimulating cAMP/PKA signaling through the PDE4 inhibitor rolipram or specific depletion of PDE4D isozymes. These lines of experimental evidence from ours and others suggest that SMC viability may be differentially, even oppositely, regulated by different cAMP/PKA signalosomes that are coupled to distinct G-protein coupled receptors (GPCRs) and/or PDEs. Although we demonstrated the roles of PDE4D in SMC apoptosis in this study, there are eleven different PDE4D variants reported to date, and the expression of different PDE4D variants in SMCs has been reported previously. The contractile/quiescent vascular SMCs primarily express PDE4D3, while the synthetic/activated SMCs express PDE4D1 and PDE4D2^47^. PDE4D8 is enriched in the pseudopodia of SMCs and regulates SMC migration^48^. These findings suggest that different PDE4D variants may be involved in distinct SMC functions. The specific PDE4D variant(s) in SMC apoptosis remain to be elucidated in the future.

We also found that Ang II-induced elevation of systolic and diastolic BP, measured by tail cuff, was significantly reduced in the SMC-specific *Pde4d* knockout mice and in the mice treated with rolipram. This raised the question of whether BP reduction contributes to the effect of PDE4D deficiency/inhibition in AAA. Daugherty et al. found that both NE and Ang II raised arterial pressure. However, Ang II but not NE induced AAA. Moreover, blocking this BP elevation using the antihypertensive drug hydroxyzine did not prevent Ang II-induced AAA occurrence and development^33^. Although previous experimental evidence indicates that Ang II induces AAA formation in mice independent of its effect on BP elevation^33^, BP may affect the outcomes of AAA progression and rupture^49^. Thus, we do not exclude the potential impact of BP reduction by PDE4D deficiency or inhibition in AAA progression or rupture.

In addition to apoptosis, we also determined whether PDE4D was associated with other phenotypes of SMCs, including proliferation, dedifferentiation, deposition of extracellular matrix (ECM), and inflammation, in AAA development. We found that Ang II significantly increased the proliferation of SMCs; however, PDE4D siRNA could not suppress the proliferation induced by Ang II (Supplementary Fig. 17a, b). SMCs can dedifferentiate into macrophage-like SMCs or osteoblast-like SMCs by changing from the contractile type to the synthetic type^50^. Our results showed that Ang II-treated SMCs expressed higher levels of the SMC contractile marker proteins smooth muscle 22α (SM22α) and SM-calponin (CNN) as well as lower levels of the synthetic marker osteopontin (OPN), but PDE4D siRNA did not reverse the dedifferentiation phenotype (Supplementary Fig. 18a–c). In addition, we found that *Mmp2* and *Mmp9* expression was upregulated by Ang II, but PDE4D deficiency only rescued *Mmp2* expression in vitro (Supplementary Fig. 19a, b). Moreover, expression of the ECM genes Collagen 1A1 (*Col1a1*), Collagen 3A1 (*Col3a1*) and fibronectin 1 (*Fn*)^51^ was elevated by Ang II stimulation, but only *Col1a1* expression was reversed by PDE4D siRNA (Supplementary Fig. 20a–c). Regarding inflammation, Sadhan et al. reported that Ang II promoted CCL2, IL-6 and TNF-α expression in SMCs^52^. Therefore, we found that Ang II stimulation increased all three cytokines; however, suppression of PDE4D did not rescue the upregulation of *Ccl2*, *Il-6,* and *Tnf-α* expression (Supplementary Fig. 21a–c). These findings indicate that PDE4D in SMCs may affect ECM deposition by regulating Col1a1 and MMP2 expression in AAA progression.

In conclusion, our study shows that PDE4D in SMCs exacerbates Ang II-induced AAA. Furthermore, the inhibition of PDE4 alleviates vascular pathogenesis and AAA formation. We verified the mechanism by which PDE4D influences SMC apoptosis via in vitro and in vivo experimental models and identified those results using a variety of molecular biological methods. This study illustrates that PDE4D in SMCs plays a pivotal role in AAA and that PDE4 inhibitors may be potential targets for AAA treatment.

## Supplementary information

SUPPLEMENTAL MATERIAL
