# Supplementary material for: Phosphodiesterase 4D contributes to angiotensin II-induced abdominal aortic aneurysm through smooth muscle cell apoptosis
Source: Exp Mol Med. 2022 Aug 23;54(8):1201–13. doi: 10.1038/s12276-022-00815-y (PMC9440214; doi:10.1038/s12276-022-00815-y)
Supplement: Supplementary file 1 — SUPPLEMENTAL MATERIAL [file 12276_2022_815_MOESM1_ESM.pdf]

Supplementary Information

Phosphodiesterase 4D contributes to angiotensin II-induced abdominal aortic aneurysm through smooth muscle cell apoptosis

Running title: PDE4D aggravates abdominal aortic aneurysm

Ran Gao[1,*], Wenjun Guo[1,*], Tianfei Fan[1,*], Junling Pang[1], Yangfeng Hou[1], Xiaohang Feng[1], Bolun Li[1], Weipeng Ge[1], Tianhui Fan[1], Tiantian Zhang[1], Jiakai Lu[2], He Jing[2], Mu Jin[3], Chen Yan[4], Jing Wang[1,†]
[1]State Key Laboratory of Medical Molecular Biology, Institute of Basic Medical Sciences, Chinese Academy of Medical Sciences, Department of Pathophysiology, Peking Union Medical College, Beijing, China
[2]Department of Anesthesiology, Beijing Anzhen Hospital, Capital Medical University, Beijing Institute of Heart, Lung and Blood Vessel Diseases, Beijing, China
[3]Department of Anesthesiology, Beijing Friendship Hospital, Capital Medical University, Beijing, China
[4]Aab Cardiovascular Research Institute, University of Rochester, School of Medicine and Dentistry, Rochester, NY 14642, USA

* Ran Gao, Wenjun Guo and Tianfei Fan contributed equally to the manuscript.

† Corresponding authors:
Dr. Jing Wang, State Key Laboratory of Medical Molecular Biology, Institute of Basic Medical Sciences, Chinese Academy of Medical Sciences, Department of Pathophysiology, Peking Union Medical College, Beijing, China. E-mail: wangjing@ibms.pumc.edu.cn.

Supplementary Figures

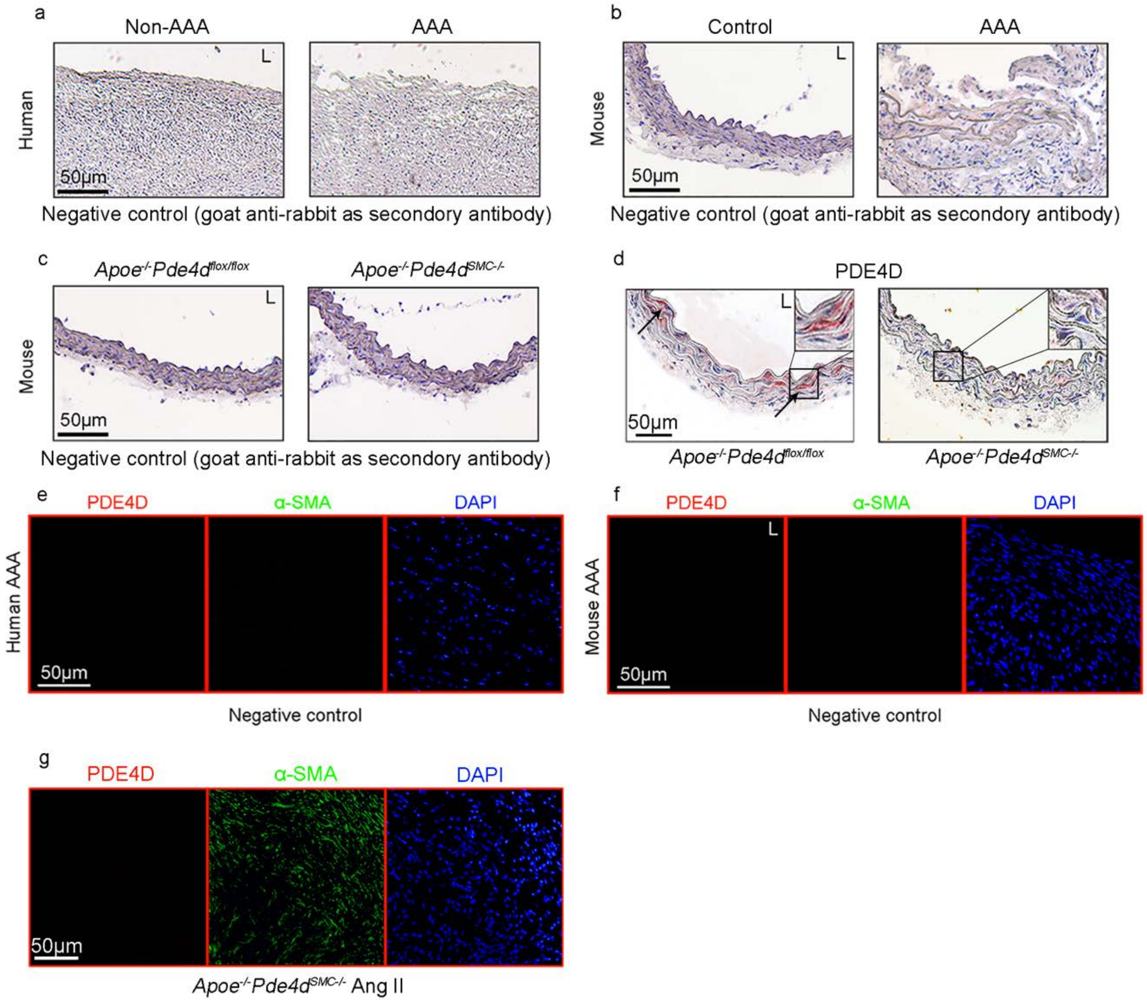

Supplementary Fig. 1 PDE4D antibody specificity and negative control of immunohistochemical staining. (a) Representative images of immunohistochemical analysis for negative control (only with goat anti-rabbit antibody as secondary antibody) of PDE4D in human Non-AAA and AAA tissues. (b) Representative images of immunohistochemical analysis for negative control (only with goat anti-rabbit antibody as secondary antibody) of PDE4D in mouse control (*Apoe*$^{-/-}$ mice treated with saline) and AAA tissues (*Apoe*$^{-/-}$ mice treated with 1000 ng kg^{-1} min^{-1} Ang II and HFD). (c) Representative images of immunohistochemical analysis for negative control (only with goat anti-rabbit antibody as secondary antibody) of PDE4D in mouse *Apoe*$^{-/-}$*Pde4d*$^{flox/flox}$ and *Apoe*$^{-/-}$*Pde4d*$^{SMC-/-}$ normal aortic vascular tissues. (d) Representative images of immunohistochemistry analysis of PDE4D in the aortas from *Apoe*$^{-/-}$*Pde4d*$^{flox/flox}$ and *Apoe*$^{-/-}$*Pde4d*$^{SMC-/-}$ with saline infusion. (e) Representative images of immunofluorescence analysis for negative control (only with secondary antibody) of PDE4D and α-SMA in human AAA tissues. (f) Representative images of immunofluorescence analysis for negative control (only with secondary antibody) of PDE4D and α-SMA in mouse AAA tissues (*Apoe*$^{-/-}$ mice treated with 1000 ng kg^{-1} min^{-1} Ang II and HFD). (g) Representative images of immunofluorescence analysis of PDE4D in *Apoe*$^{-/-}$*Pde4d*$^{SMC-/-}$ Ang II mice treated with 1000 ng kg^{-1} min^{-1} Ang II and HFD. L: lumen.

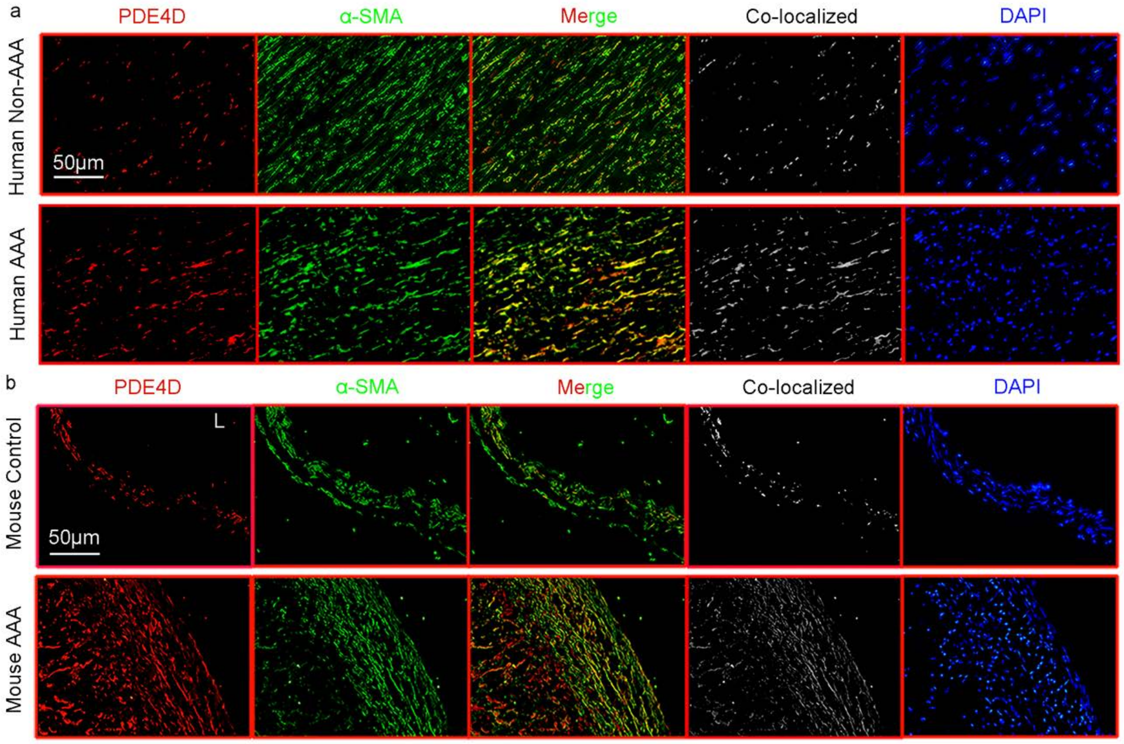

Supplementary Fig. 2 Immunofluorescence staining of PDE4D, α smooth muscle actin (α-SMA, smooth muscle cell marker) and 4',6-diamidino-2-phenylindole (DAPI, nucleus marker). (a) Immunofluorescence staining of PDE4D, α-SMA and DAPI in human Non-AAA sections and AAA tissues (6μm). We obtained similar results from at least 3 different sets of human tissues in separate experiments. PDE4D: red, α-SMA: green, DAPI: blue. (b) Immunofluorescence staining of PDE4D, α-SMA and DAPI in mouse control sections and AAA tissues (6μm). We obtained similar results from at least 3 different sets of mouse samples in separate experiments. PDE4D: red, α-SMA: green, DAPI: blue. L: lumen.

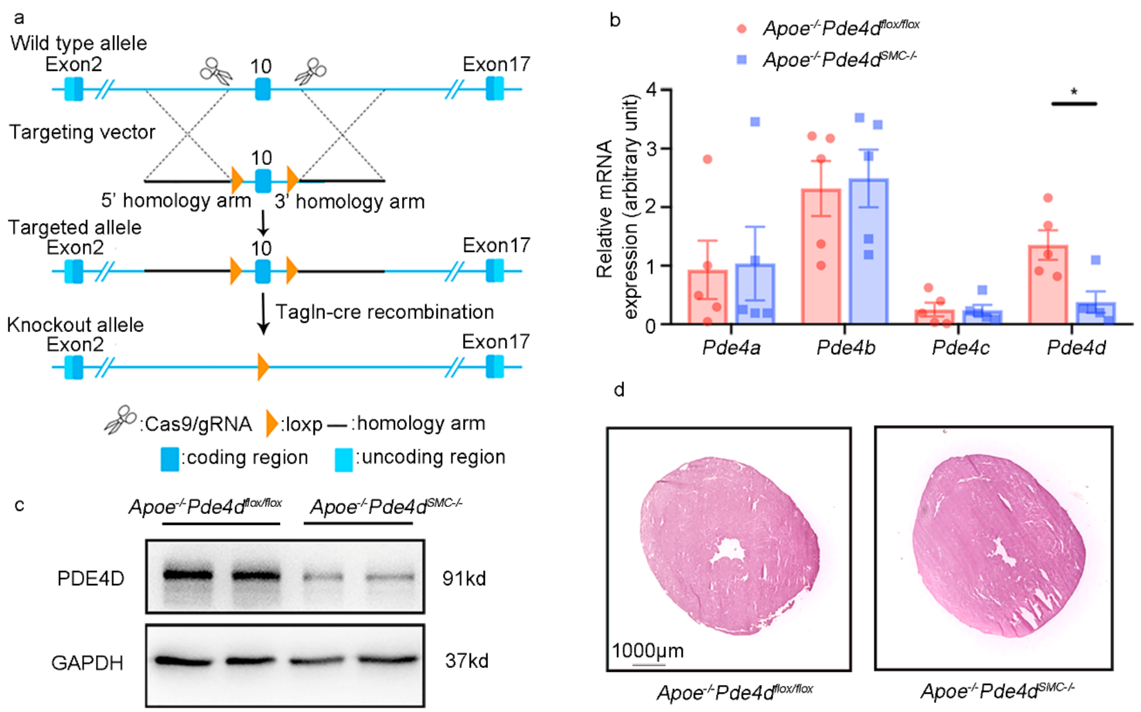

Supplementary Fig. 3 The identification of smooth muscle cell (SMC)-specific depletion phosphodiesterase (PDE) 4D (*Pde4d*$^{SMC-/-}$) mice. (a) Generation of smooth muscle-specific depletion *Pde4d* mice via using Cre-LoxP recombination system. Exon10 is deleted on *Tagln*-Cre–mediated recombination. (b) The mRNA levels of *Pde4a*, *Pde4b*, *Pde4c* and *Pde4d* in mouse aortas. *p<0.05, Mann-Whitney test, mean ± SEM, *Apoe*$^{-/-}$*Pde4d*$^{flox/flox}$ (n=5) and *Apoe*$^{-/-}$*Pde4d*$^{SMC-/-}$ mice (n=5) with saline infusion. (c) Representative immunoblot analys of PDE4D expression in mouse aortas. *Apoe*$^{-/-}$*Pde4d*$^{flox/flox}$ (n=2) and *Apoe*$^{-/-}$*Pde4d*$^{SMC-/-}$ mice (n=2) with saline infusion. (d) Representative images of heart tissues by H and E staining in *Apoe*$^{-/-}$*Pde4d*$^{flox/flox}$ and *Apoe*$^{-/-}$*Pde4d*$^{SMC-/-}$ with saline infusion.

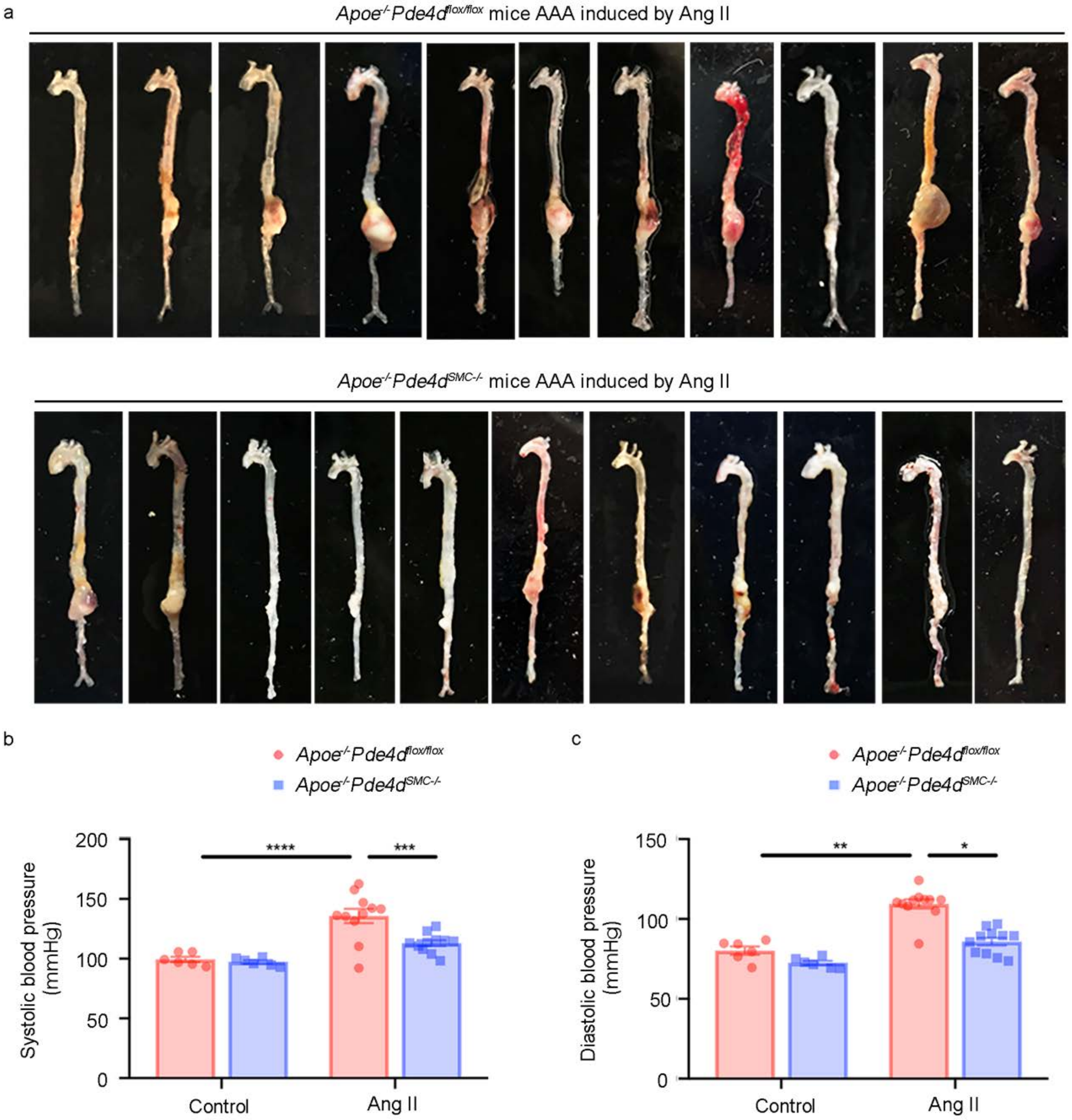

Supplementary Fig. 4 The all AAA sample images and blood pressure of *Apoe*$^{-/-}$*Pde4d*$^{flox/flox}$ and *Apoe*$^{-/-}$*Pde4d*$^{SMC-/-}$ mice. (a) Images of all AAA samples shown in Fig. 3b-c. (b) Systolic blood pressure in *Apoe*$^{-/-}$*Pde4d*$^{flox/flox}$ and *Apoe*$^{-/-}$*Pde4d*$^{SMC-/-}$ mice. Each dot represents an individual mouse. ***$p<0.001$, ****$p<0.0001$, two-way ANOVA with Holm-Sidak's post-hoc test, mean ± SEM, *Apoe*$^{-/-}$*Pde4d*$^{flox/flox}$ (n=6) and *Apoe*$^{-/-}$*Pde4d*$^{SMC-/-}$ (n=6) with saline infusion, *Apoe*$^{-/-}$*Pde4d*$^{flox/flox}$ (n=11) and *Apoe*$^{-/-}$*Pde4d*$^{SMC-/-}$ (n=11) with Ang II infusion. (c) Diastolic blood pressure in *Apoe*$^{-/-}$*Pde4d*$^{flox/flox}$ and *Apoe*$^{-/-}$*Pde4d*$^{SMC-/-}$ mice. *$p<0.05$, **$p<0.01$, ANOVA Kruskal-Wallis test with post Dunn's multiple comparisons test, mean ± SEM, *Apoe*$^{-/-}$*Pde4d*$^{flox/flox}$ (n=6) and *Apoe*$^{-/-}$*Pde4d*$^{SMC-/-}$ (n=6) with saline infusion, *Apoe*$^{-/-}$*Pde4d*$^{flox/flox}$ (n=11) and *Apoe*$^{-/-}$*Pde4d*$^{SMC-/-}$ (n=11) with Ang II infusion.

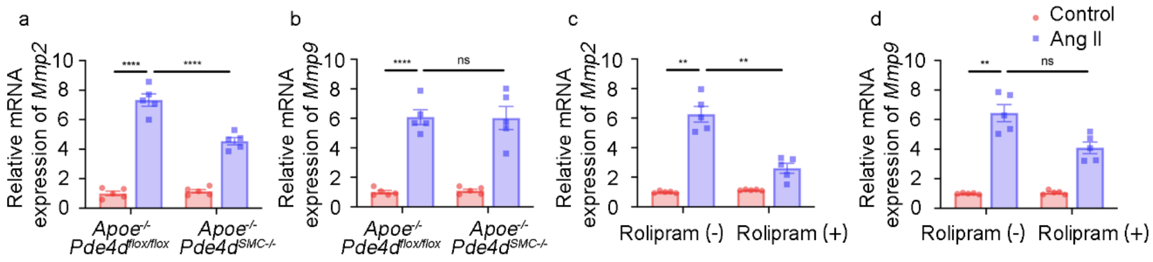

Supplementary Fig. 5 PDE4D knockout and rolipram regulate AAA induced matrix metallopeptidases *in vivo*. (a) RT-PCR quantification of *Mmp2* mRNA in aorta of *Apoe*$^{-/-}$*Pde4d*$^{flox/flox}$ and *Apoe*$^{-/-}$*Pde4d*$^{SMC-/-}$ mice with or without 1000 ng kg^{-1} min^{-1} Ang II treatment and high fat diet (HFD) for 28 days (fold change versus one of control subjects). ****p<0.0001, two-way ANOVA with Holm-Sidak's post-hoc test, mean ± SEM, *Apoe*$^{-/-}$*Pde4d*$^{flox/flox}$ (n=5) and *Apoe*$^{-/-}$*Pde4d*$^{SMC-/-}$ (n=5) with saline infusion, *Apoe*$^{-/-}$*Pde4d*$^{flox/flox}$ (n=5) and *Apoe*$^{-/-}$*Pde4d*$^{SMC-/-}$ (n=5) with Ang II infusion. (b) RT-PCR quantification of *Mmp9* mRNA in aorta of *Apoe*$^{-/-}$*Pde4d*$^{flox/flox}$ and *Apoe*$^{-/-}$*Pde4d*$^{SMC-/-}$ mice with or without 1000 ng kg^{-1} min^{-1} Ang II treatment and high fat diet (HFD) for 28 days (fold change versus one of control subjects). ****p<0.0001, ns: no significant difference, two-way ANOVA with Holm-Sidak's post-hoc test, mean ± SEM, *Apoe*$^{-/-}$*Pde4d*$^{flox/flox}$ (n=5) and *Apoe*$^{-/-}$*Pde4d*$^{SMC-/-}$ (n=5) with saline infusion, *Apoe*$^{-/-}$*Pde4d*$^{flox/flox}$ (n=5) and *Apoe*$^{-/-}$*Pde4d*$^{SMC-/-}$ (n=5) with Ang II infusion. (c) RT-PCR quantification of *Mmp2* mRNA in aorta of *Apoe*$^{-/-}$ mice with or without Ang II and high fat diet (HFD) / rolipram treatment. (fold change versus one of control subjects). **p<0.01, Welch ANOVA with Dunnett's T3 post-hoc test, mean ± SEM, Vehicle (n=5) and Rolipram (n=5) with saline infusion, Vehicle (n=5) and Rolipram (n=5) with Ang II infusion. (d) RT-PCR quantification of *Mmp9* mRNA in aorta of *Apoe*$^{-/-}$ mice with or without Ang II and high fat diet (HFD) / rolipram treatment. (fold change versus one of control subjects). **p<0.01, ns: no significant difference, Welch ANOVA with Dunnett's T3 post-hoc test, mean ± SEM, Vehicle (n=5) and Rolipram (n=5) with saline infusion, Vehicle (n=5) and Rolipram (n=5) with Ang II infusion.

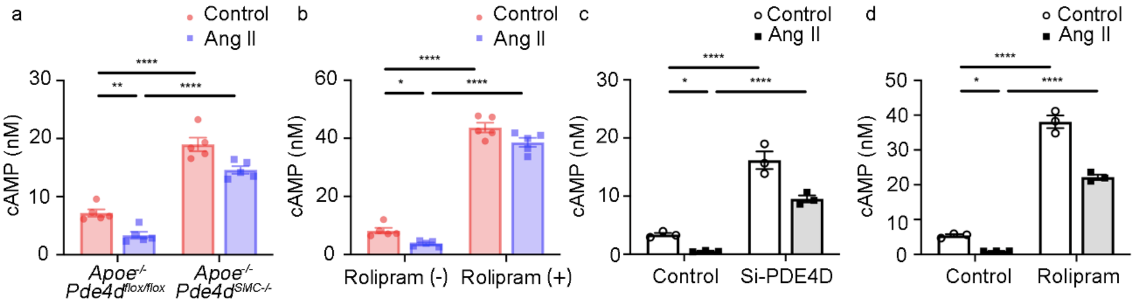

Supplementary Fig. 6 PDE4D suppression and rolipram activate cAMP production *in vivo* and *in vitro*. (a) cAMP concentration (nM) in aorta of *Apoe*$^{-/-}$*Pde4d*$^{flox/flox}$ and *Apoe*$^{-/-}$*Pde4d*$^{SMC-/-}$ mice with or without 1000 ng kg^{-1} min^{-1} Ang II treatment and high fat diet (HFD) for 28 days. **$p<0.01$, ****$p<0.0001$, two-way ANOVA with Holm-Sidak's post-hoc test, mean ± SEM, *Apoe*$^{-/-}$*Pde4d*$^{flox/flox}$ (n=5) and *Apoe*$^{-/-}$*Pde4d*$^{SMC-/-}$ (n=5) with saline infusion, *Apoe*$^{-/-}$*Pde4d*$^{flox/flox}$ (n=5) and *Apoe*$^{-/-}$*Pde4d*$^{SMC-/-}$ (n=5) with Ang II infusion. (b) cAMP concentration (nM) in aorta of *Apoe*$^{-/-}$ mice with or without Ang II and high fat diet (HFD) / rolipram treatment. *$p<0.05$, ****$p<0.0001$, two-way ANOVA with Holm-Sidak's post-hoc test, mean ± SEM, Vehicle (n=5) and Rolipram (n=5) with saline infusion, Vehicle (n=5) and Rolipram (n=5) with Ang II infusion. (c) cAMP concentration (nM) in SMCs treated with Ang II (100 nM, 24h) and / or PDE4D siRNA (200 nM, 48h) as indicated. *$p<0.05$, ****$p<0.0001$, two-way ANOVA with Holm-Sidak's post-hoc test, mean ± SEM, n=3 separate experiments. (d) cAMP concentration (nM) in SMCs treated with or without Ang II (100 nM, 24h) / rolipram (500 nM, 24.5h) as indicated. *$p<0.05$, ****$p<0.0001$, two-way ANOVA with Holm-Sidak's post-hoc test, mean ± SEM, n=3 separate experiments.

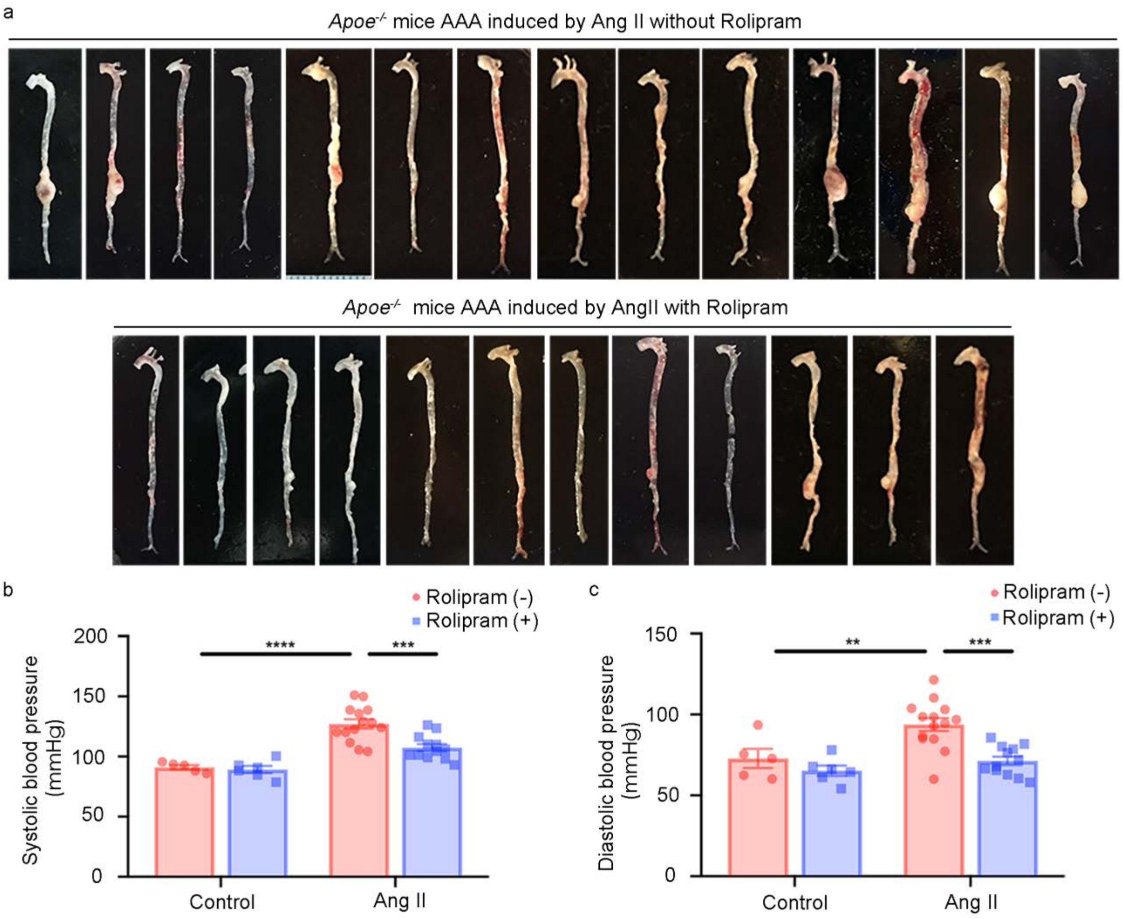

Supplementary Fig. 7 The all AAA sample images and blood pressure of *Apoe*$^{-/-}$ mice with or without rolipram treatment. (a) Images of all AAA samples shown in Fig. 4b-c. (b) Systolic blood pressure in *Apoe*$^{-/-}$ mice with or without Ang II/rolipram treatment. ***p<0.001, ****p<0.0001, two-way ANOVA with Holm-Sidak's post-hoc test, mean ± SEM, Vehicle (n=5) and Rolipram (n=6) with saline infusion, Vehicle (n=14) and Rolipram (n=12) with Ang II infusion. (c) Diastolic blood pressure in *Apoe*$^{-/-}$ mice with or without Ang II/rolipram treatment. **p<0.01, ***p<0.001, two-way ANOVA with Holm-Sidak's post-hoc test, mean ± SEM, Vehicle (n=5) and Rolipram (n=6) with saline infusion, Vehicle (n=14) and Rolipram (n=12) with Ang II infusion.

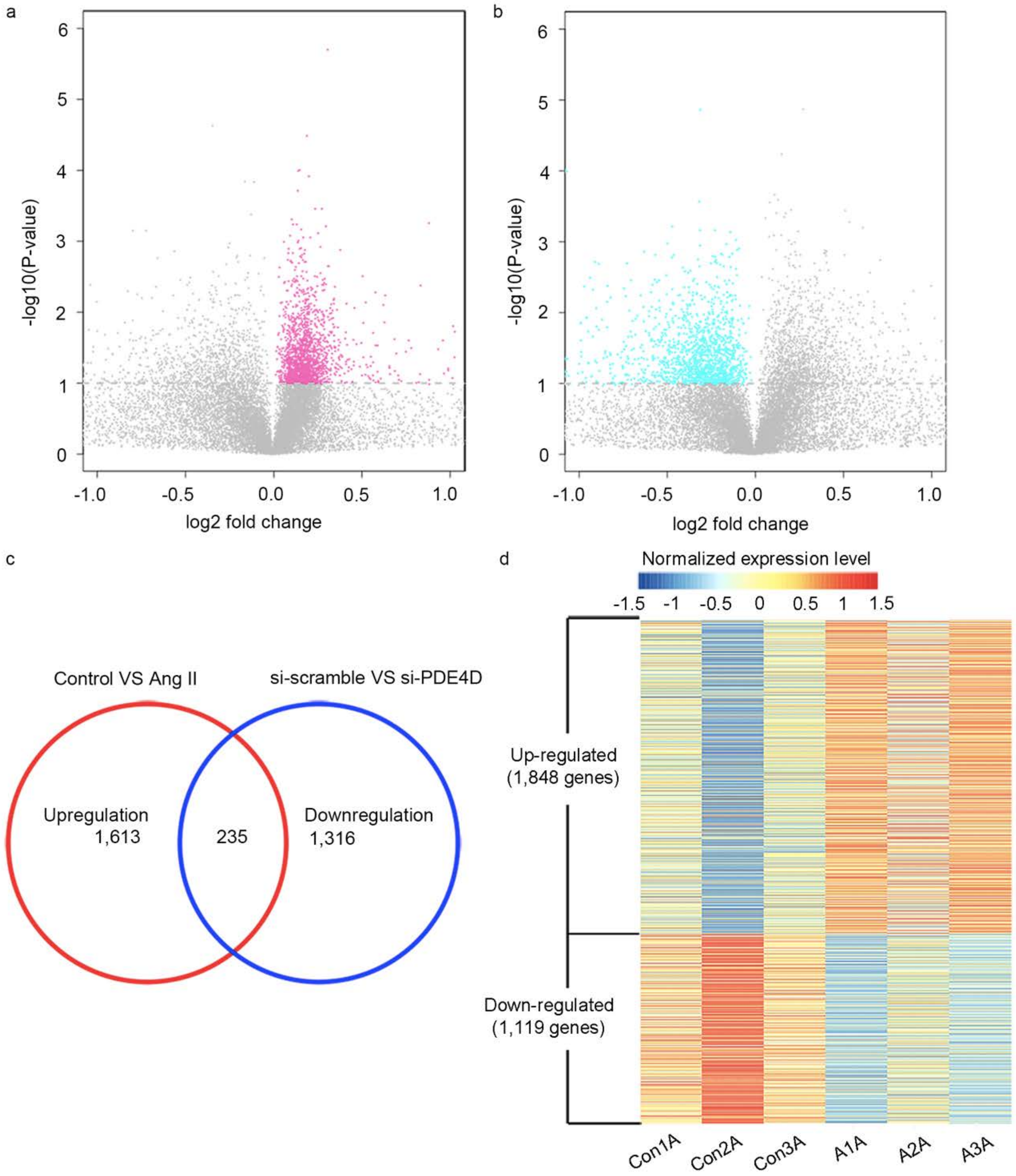

Supplementary Fig. 8 Significantly altered genes and pathways in SMCs treated with Ang II (100 nM, 24h) or PDE4D siRNA (200 nM, 48h) through RNA sequencing. (a) Volcano plot of the differentially expressed genes in SMC treated with Ang II. Pink dots represent genes with increased expression after Ang II treatment. (b) Volcano plot of the differentially expressed genes in SMC treated with PDE4D siRNA. Blue dots represent genes with decreased expression after the treatment of PDE4D siRNA. (c) Venn diagram of differentially expressed genes between the up-regulated genes in Ang II stimulated SMCs and the down-regulated genes in PDE4D siRNA treated SMCs. (d) Heatmap of genes differentially expressed in SMCs with or without Ang II (100 nM, 24h) stimulation. There are 3 replicates per group, with each column represents an individual replicate. Each row represents an individual gene. The color bar represents normalized expression levels (row z-score) of genes, with upregulated genes shown in red and downregulated genes in blue.

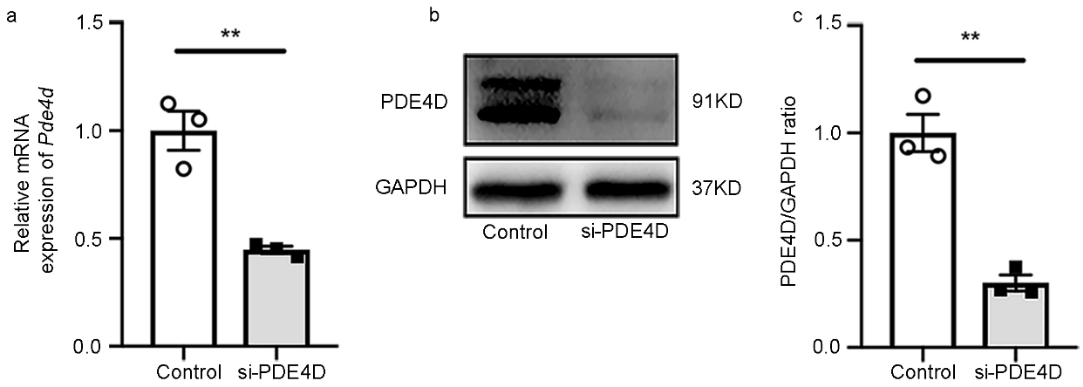

Supplementary Fig. 9 The mRNA and protein expression of PDE4D after transfection with PDE4D siRNA in SMCs. (a) SMCs were transfected with control siRNA or PDE4D siRNA for 48h (200 nM), and *Pde4d* expression was then detected by RT-PCR. $^{**}p<0.01$, unpaired Student's t-test, mean ± SEM, n=3 separate experiments. (b) Immunoblot analysis of PDE4D expression in SMCs treated with PDE4D siRNA (200 nM, 48h). (c) Quantification of PDE4D protein expression by immunoblotting in (b) normalized to GAPDH protein (fold change versus controls). $^{**}p<0.01$, unpaired Student's t-test, mean ± SEM, n=3 separate experiments.

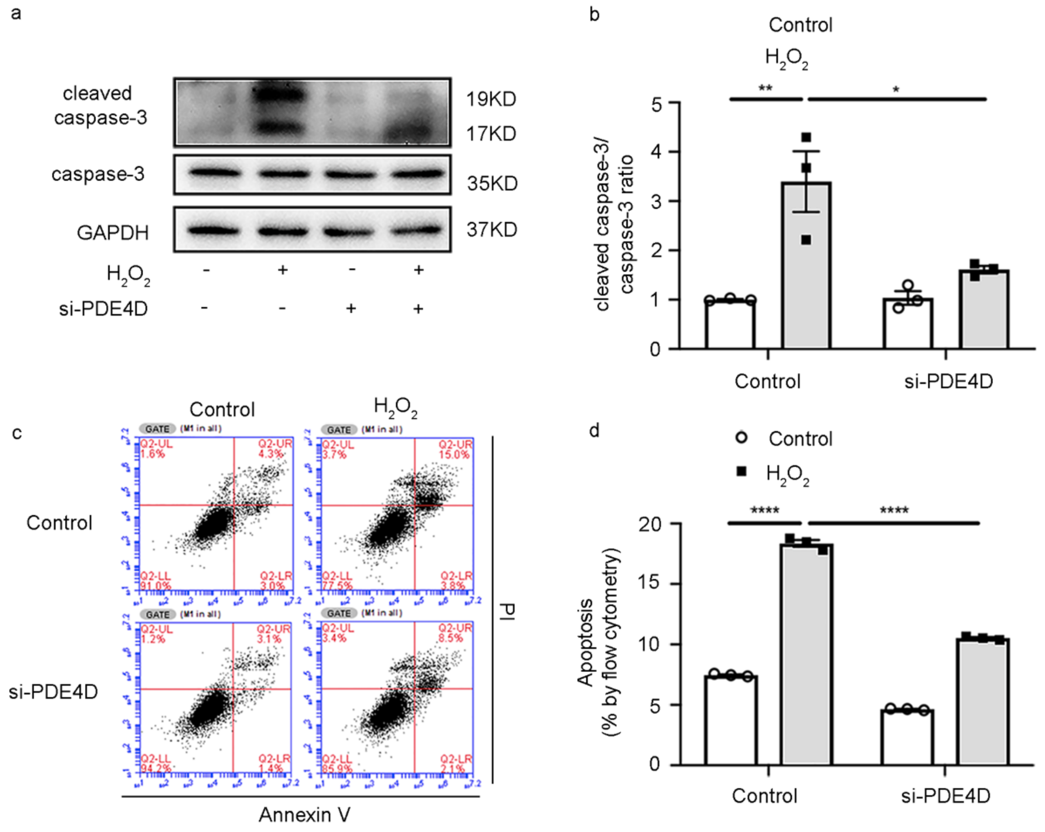

Supplementary Fig. 10 PDE4D siRNA inhibits H_2O_2-induced smooth muscle cell (SMC) apoptosis *in vitro*. (a) Immunoblot analysis of caspase-3 and cleaved caspase-3 expression in SMCs treated with or without H_2O_2 (300 µM, 24h) / PDE4D siRNA (200 nM, 48h) as indicated. (b) Quantification of cleaved caspase-3 protein expression by immunoblotting in (a) normalized to caspase-3 protein (fold change versus control). **p*<0.05, ***p*<0.01, two-way ANOVA with Holm-Sidak's post-hoc test, mean ± SEM, n=3 separate experiments. (c) Flow cytometric analysis of annexin V/ propidium iodide (PI) stained SMCs treated with or without H_2O_2 (300 µM, 24h) / PDE4D siRNA (200 nM, 48h) as indicated. (d) Quantification of total (early and late) apoptosis rates of annexin V/PI stained SMCs treated with or without H_2O_2 (300 µM, 24h) / PDE4D siRNA (200 nM, 48h) as indicated. *****p*<0.0001, two-way ANOVA with Holm-Sidak's post-hoc test, mean ± SEM, n=3 separate experiments.

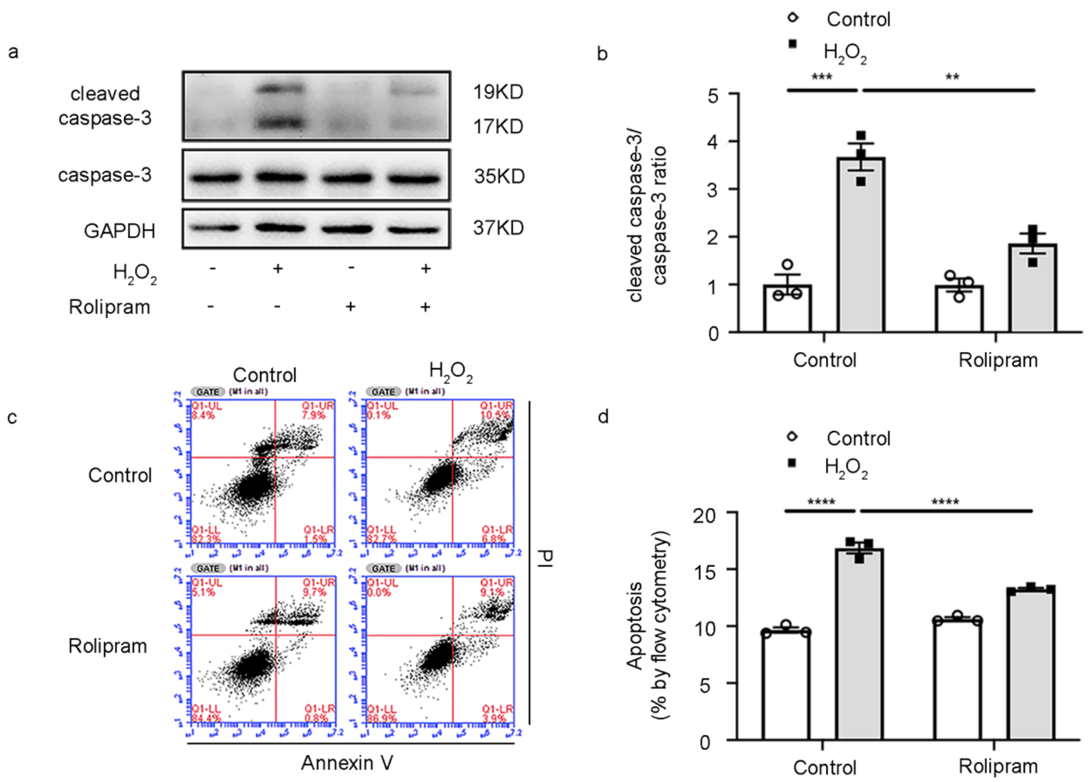

Supplementary Fig. 11 Rolipram inhibits H_2O_2-induced SMC apoptosis *in vitro*. (a) Immunoblot analysis of caspase-3 and cleaved caspase-3 expression in SMCs treated with or without H_2O_2 (300 µM, 24h) / rolipram (500 nM, 24.5h). (b) Quantification of cleaved caspase-3 protein expression by immunoblotting in (a) normalized to caspase-3 protein (fold change versus control). $^{**}p<0.01$, $^{***}p<0.001$, two-way ANOVA with Holm-Sidak's post-hoc test, mean ± SEM, n=3 separate experiments. (c) Flow cytometric analysis of annexin V/ propidium iodide (PI) stained SMCs treated with or without H_2O_2 (300 µM, 24h) / rolipram (500 nM, 24.5h) as indicated. (d) Quantification of total (early and late) apoptosis rates of annexin V/PI stained SMCs treated with or without H_2O_2 (300 µM, 24h) / rolipram (500 nM, 24.5h) as indicated. $^{****}p<0.0001$, two-way ANOVA with Holm-Sidak's post-hoc test, mean ± SEM, n=3 separate experiments.

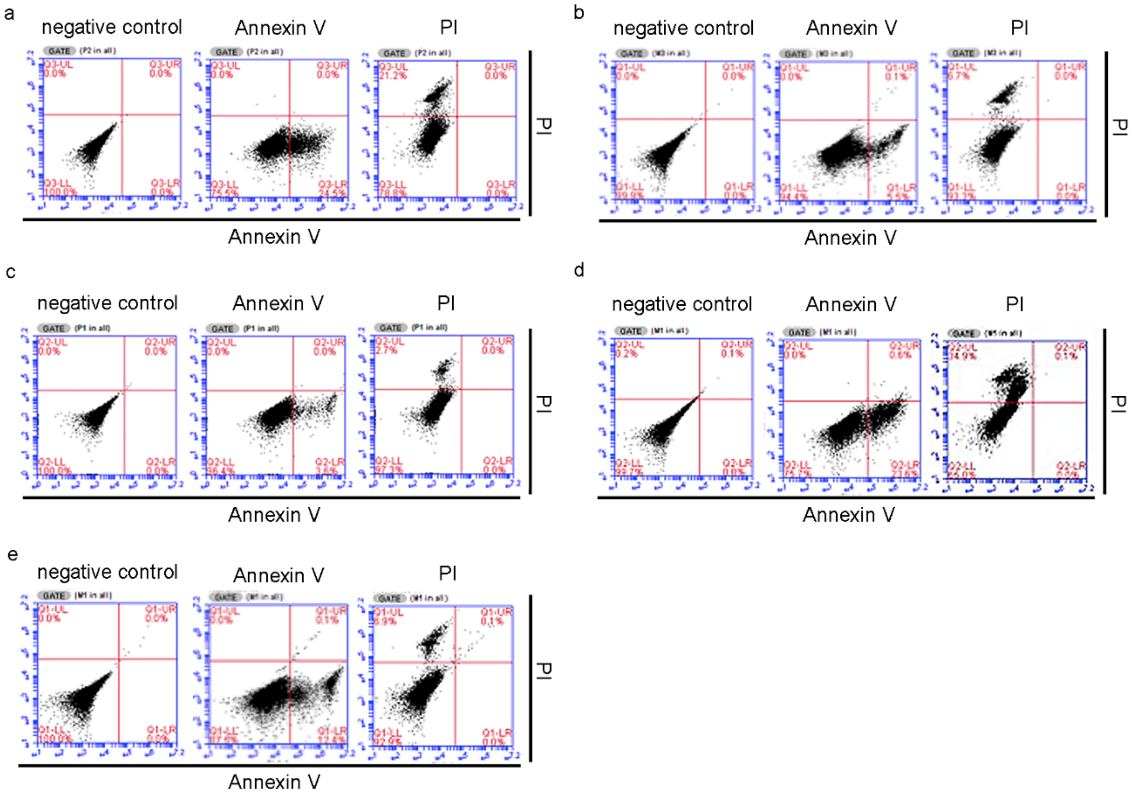

Supplementary Fig. 12 Negative control, Annexin V positive cells, PI positive cells of flow cytometric. (a) Negative control, PI positive cells, Annexin V positive cells of flow cytometric analysis of annexin V/PI stained SMCs treated with or without Ang II (100 nM, 24h) / PDE4D siRNA (200 nM, 48h) in Fig. 5d. (b) Negative control, PI positive cells, Annexin V positive cells of flow cytometric analysis of annexin V/PI stained SMCs treated with or without Ang II (100 nM, 24h) / rolipram (500 nM, 24.5h) in Fig. 5h. (c) Negative control, PI positive cells, Annexin V positive cells of flow cytometric analysis of annexin V/PI stained SMCs treated with or without HIF1an siRNA (100 nM, 48h) / PDE4D siRNA (200 nM, 48h) in Fig. 7h. (d) Negative control, PI positive cells, Annexin V positive cells of flow cytometric analysis of annexin V/PI stained SMCs treated with or without H_2O_2 (300 μM, 24h) / PDE4D siRNA (200 nM, 48h) in Supplementary Fig. 10c. (e) Negative control, PI positive cells, Annexin V positive cells of flow cytometric analysis of annexin V/PI stained SMCs treated with or without H_2O_2 (300 μM, 24h) / rolipram (500 nM, 24.5h) in Supplementary Fig. 11c.

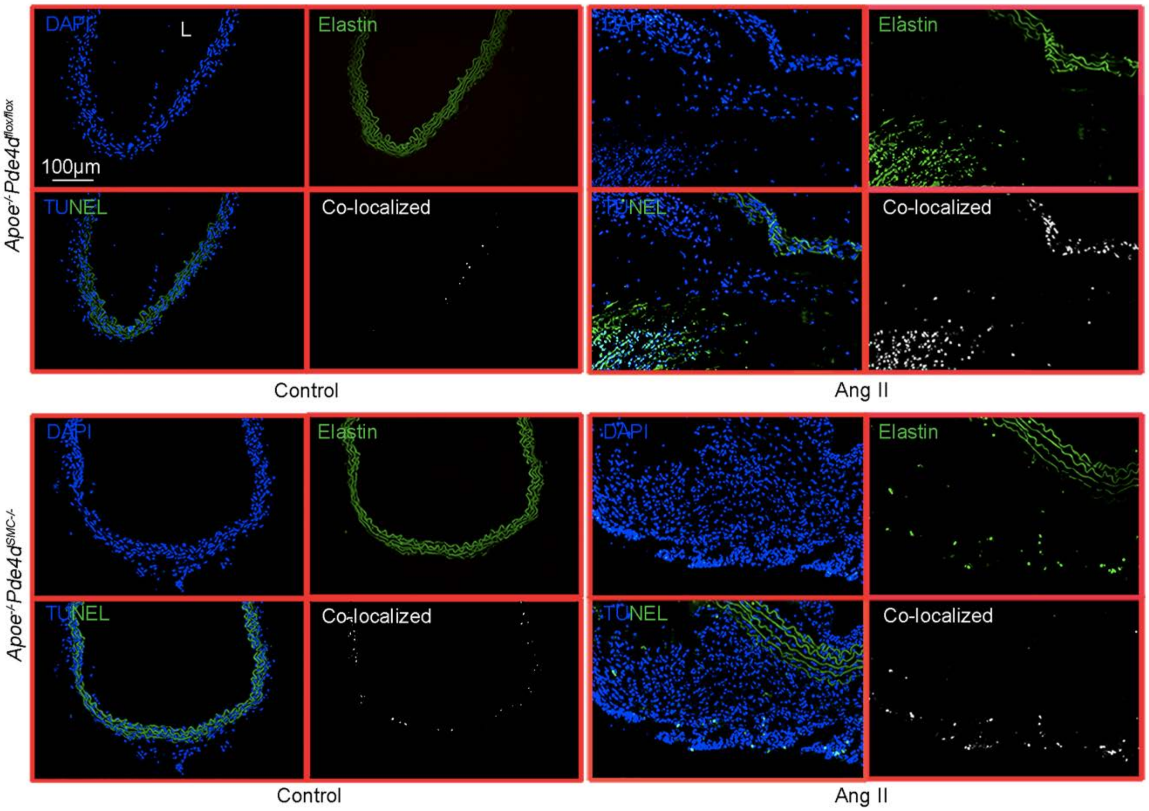

Supplementary Fig. 13 Representative images of terminal deoxynucleotidyl transferase (TdT)-mediated dUTP nick-end labeling (TUNEL) staining of apoptosis cells in abdominal aorta tissue in *Apoe*$^{-/-}$*Pde4d*$^{flox/flox}$ and *Apoe*$^{-/-}$*Pde4d*$^{SMC-/-}$ mice with or without Ang II treatment.

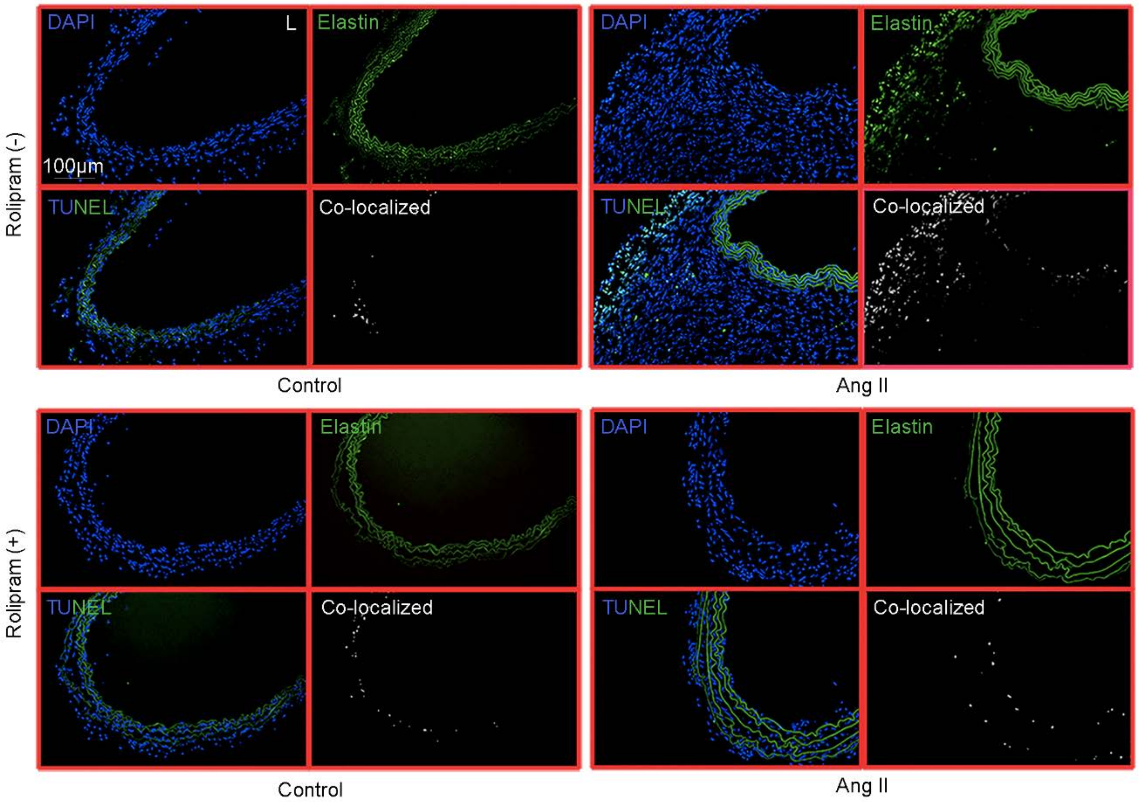

Supplementary Fig. 14 Representative images of TUNEL staining of apoptosis cells in abdominal aorta tissue in *Apoe*$^{-/-}$ mice treated with or without Ang II / rolipram.

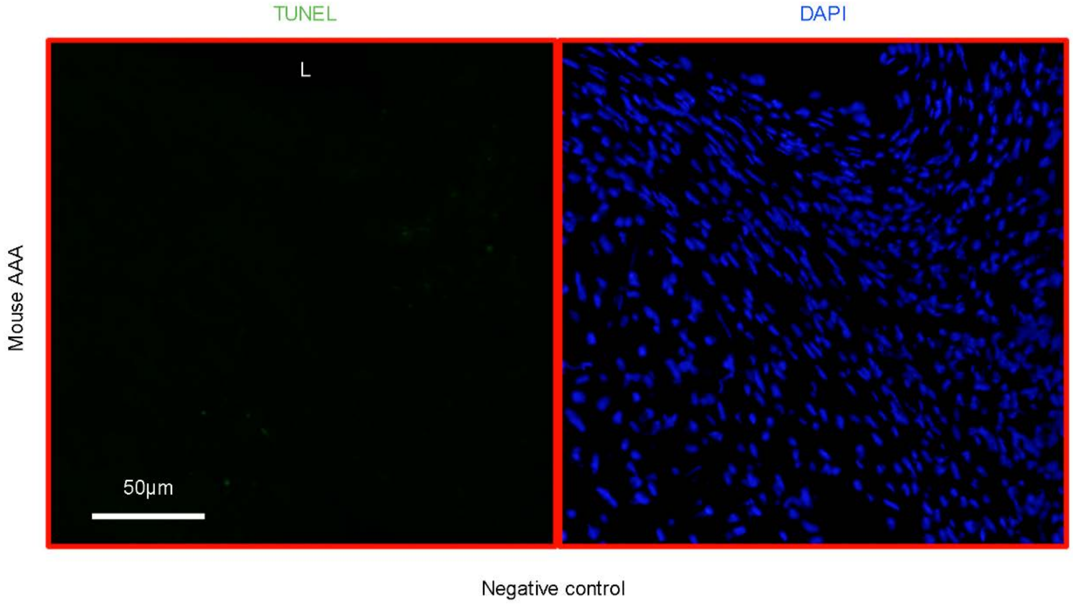

Supplementary Fig. 15 Representative images of negative control of TUNEL staining in mouse AAA tissues (*Apoe*$^{-/-}$ mice treated with 1000 ng kg^{-1} min^{-1} Ang II and HFD). L: lumen.

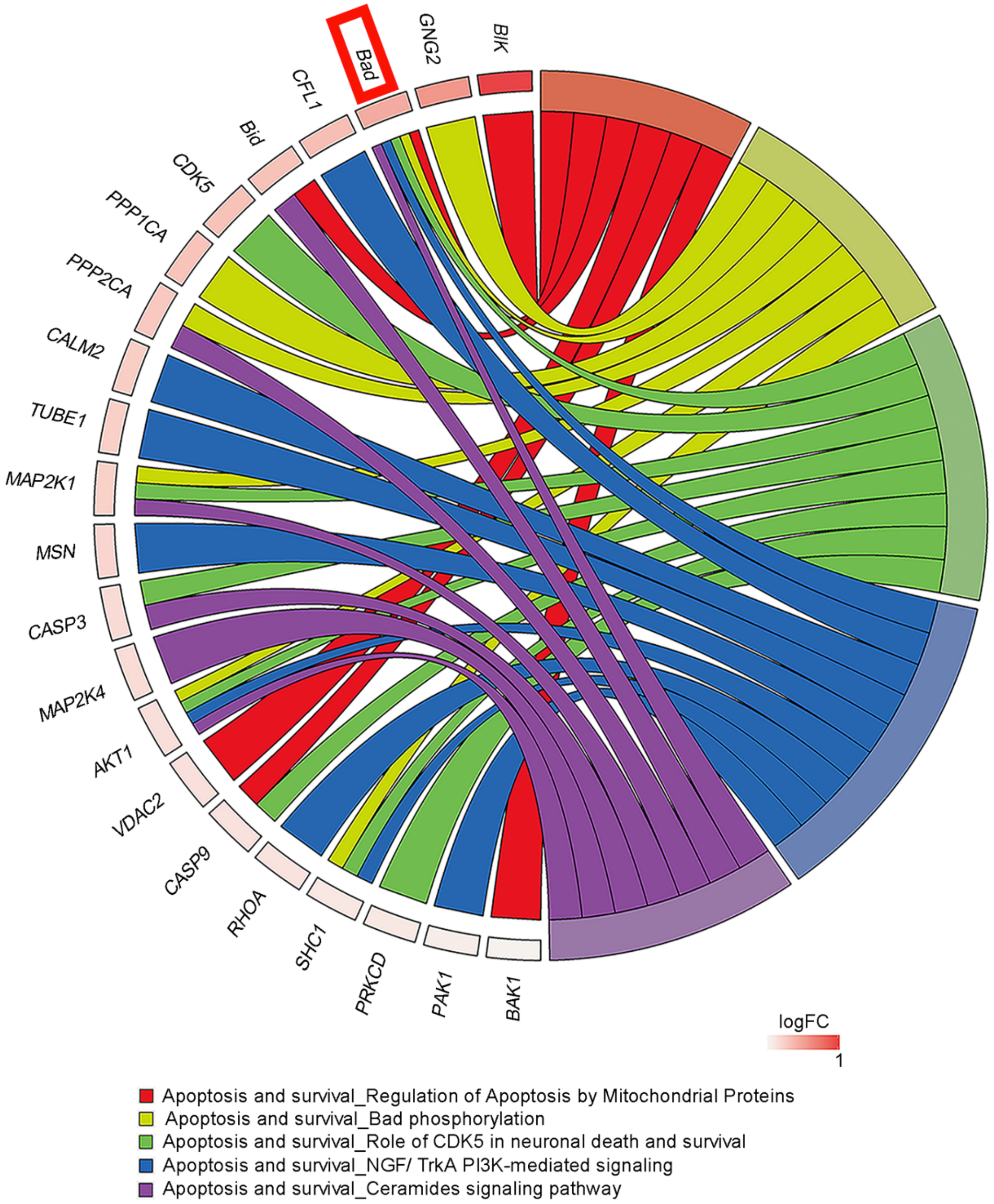

Supplementary Fig. 16 The relationship between selected apoptosis-related pathways and the differentially expressed genes (DEGs). The right half of the circle displayed the five apoptosis-related pathways we focused on, and the left half of the circle displayed the DEGs that are involved in these pathways. Each link between them represented that the gene was assigned to the corresponding pathway. The genes were ordered based on their "logFC" during the differential analysis.

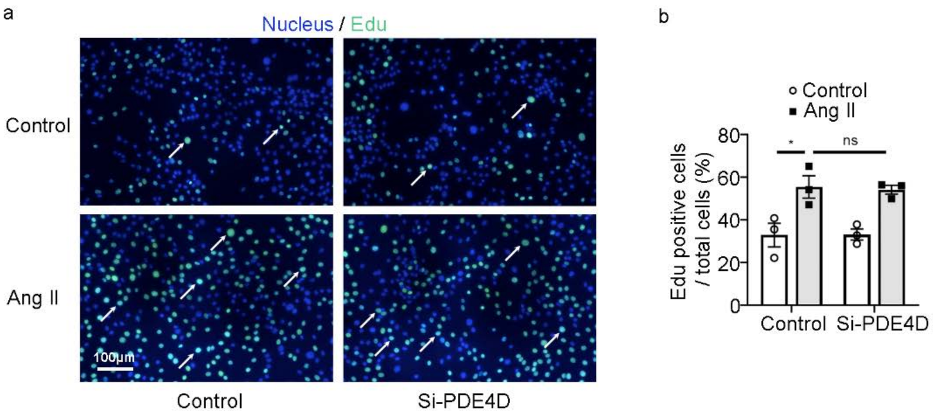

Supplementary Fig. 17 PDE4D suppression does not affect Ang II induced proliferation in SMCs. (a) Representative images of EdU staining in SMCs treated with Ang II (100 nM, 24h) and / or PDE4D siRNA (200 nM, 48h) as indicated. (b) Quantification of SMC proliferation rates by EdU staining in (a). EdU positive cell rate was mean value of three random view in each separate experiment. *p<0.05, ns: no significant difference, two-way ANOVA with Holm-Sidak's post-hoc test, mean ± SEM, n=3 separate experiments.

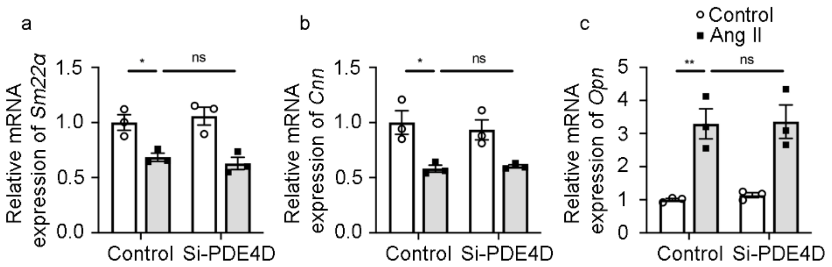

Supplementary Fig. 18 PDE4D suppression do not regulate Ang II induced dedifferentiation in SMCs. (a) RT-PCR analysis of *Sm22α* expression in SMCs treated with Ang II (100 nM, 24h) and / or PDE4D siRNA (200 nM, 48h) as indicated (fold change versus control). *p<0.05, ns: no significant difference, two-way ANOVA with Holm-Sidak's post-hoc test, mean ± SEM, n=3 separate experiments. (b) RT-PCR analysis of *Cnn* expression in SMCs treated with Ang II (100 nM, 24h) and / or PDE4D siRNA (200 nM, 48h) as indicated (fold change versus control). *p<0.05, ns: no significant difference, two-way ANOVA with Holm-Sidak's post-hoc test, mean ± SEM, n=3 separate experiments. (c) RT-PCR analysis of *Opn* expression in SMCs treated with Ang II (100 nM, 24h) and / or PDE4D siRNA (200 nM, 48h) as indicated (fold change versus control). **p<0.01, ns: no significant difference, two-way ANOVA with Holm-Sidak's post-hoc test, mean ± SEM, n=3 separate experiments.

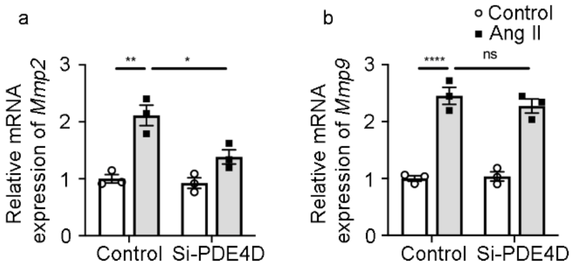

Supplementary Fig. 19 PDE4D suppression regulate Ang II induced matrix metallopeptidases in SMCs. (a) RT-PCR analysis of *Mmp2* expression in SMCs treated with Ang II (100 nM, 24h) and / or PDE4D siRNA (200 nM, 48h) as indicated (fold change versus control). *$p<0.05$, **$p<0.01$, two-way ANOVA with Holm-Sidak's post-hoc test, mean ± SEM, n=3 separate experiments. (b) RT-PCR analysis of *Mmp9* expression in SMCs treated with Ang II (100 nM, 24h) and / or PDE4D siRNA (200 nM, 48h) as indicated (fold change versus control). ****$p<0.0001$, ns: no significant difference, two-way ANOVA with Holm-Sidak's post-hoc test, mean ± SEM, n=3 separate experiments.

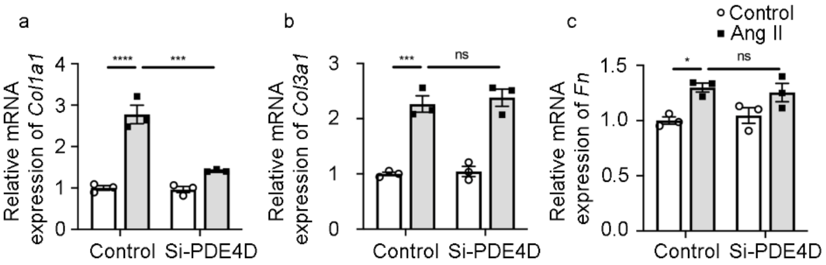

Supplementary Fig. 20 PDE4D suppression and rolipram regulate Ang II induced deposition of extracellular matrix in SMCs. (a) RT-PCR analysis of *Col1a1* expression in SMCs treated with Ang II (100 nM, 24h) and / or PDE4D siRNA (200 nM, 48h) as indicated (fold change versus control). ***$p<0.001$, ****$p<0.0001$, two-way ANOVA with Holm-Sidak's post-hoc test, mean ± SEM, n=3 separate experiments. (b) RT-PCR analysis of *Col3a1* expression in SMCs treated with Ang II (100 nM, 24h) and / or PDE4D siRNA (200 nM, 48h) as indicated (fold change versus control). ***$p<0.001$, ns: no significant difference, two-way ANOVA with Holm-Sidak's post-hoc test, mean ± SEM, n=3 separate experiments. (c) RT-PCR analysis of *Fn* expression in SMCs treated with Ang II (100 nM, 24h) and / or PDE4D siRNA (200 nM, 48h) as indicated (fold change versus control). *$p<0.05$, ns: no significant difference, two-way ANOVA with Holm-Sidak's post-hoc test, mean ± SEM, n=3 separate experiments.

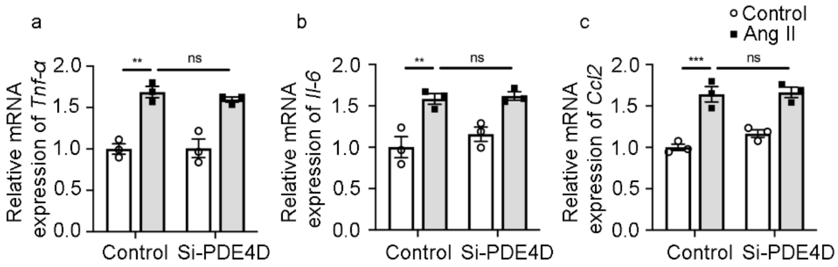

Supplementary Fig. 21 PDE4D suppression and rolipram do not regulate Ang II induced inflammation in SMCs. (a) RT-PCR analysis of *Tnf-α* expression in SMCs treated with Ang II (100 nM, 24h) and / or PDE4D siRNA (200 nM, 48h) as indicated (fold change versus control). **p<0.01, ns: no significant difference, two-way ANOVA with Holm-Sidak's post-hoc test, mean ± SEM, n=3 separate experiments. (b) RT-PCR analysis of *Il-6* expression in SMCs treated with Ang II (100 nM, 24h) and / or PDE4D siRNA (200 nM, 48h) as indicated (fold change versus control). **p<0.01, ns: no significant difference, two-way ANOVA with Holm-Sidak's post-hoc test, mean ± SEM, n=3 separate experiments. (c) RT-PCR analysis of *Ccl2* expression in SMCs treated with Ang II (100 nM, 24h) and / or PDE4D siRNA (200 nM, 48h) as indicated (fold change versus control). ***p<0.001, ns: no significant difference, two-way ANOVA with Holm-Sidak's post-hoc test, mean ± SEM, n=3 separate experiments.

Supplementary Tables

Supplementary Table 1. Clinical information of human samples

	Control (n=6)	AAA patients (n=9)	*p*
Sex: Male[F]	83.33% (5/6)	100% (9/9)	0.400
Age(yrs)[T]			
Mean	72.17± 22.46	48.56± 15.46	0.054
Median	83.5	49	
Range	42-92	15-65	
Hypertension[F]	0% (0/6)	33.33% (3/9)	0.229
Diabetes[F]	16.67% (1/6)	11.11% (1/9)	1.000
Smoking[F]	0% (0/6)	44.44% (4/9)	0.103
Alcohol[F]	0% (0/6)	33.33% (3/9)	0.229

F Fisher's exact test; T t-test $*p<0.05$

Supplementary Table 2. Primer sequences

	Forward-Primer	Reverse-Primer
h*PDE4A*	CTGCGACATCTTCCAGAACCTC	GCTGGTCACTTTCTTGGTCTCC
h*PDE4B*	TAGTCAGCCTCCTGTCTCCAGA	GAAGCCATCTCACTGACAGACC
h*PDE4C*	AGGTCACTACCACGCCAATGTG	CAGCCAGGATTTCCAAGTCTGTG
h*PDE4D*	GGACACTTTGGAGGACAATCGTG	CCTTTTCCGTGTCTGACTCACC
m*Pde4a*	AAAGCTGGTACACACCGGAAG	CAGGCCCCATTTGCTCAAGT
m*Pde4b*	CAACGCCAGACACTCAGGAA	AGAACACCGGAGCTTGTCAC
m*Pde4c*	GGTGTGATCCTGAGACGGTT	TTTCGAGGTCAAAGCTGCTC
m*Pde4d*	CTCTCGGAGCAAAAGTGCCT	GATTCGCTTCGCAAGTCTGC
m*Mmp2*	CAAGGATGGACTCCTGGCACAT	TACTCGCCATCAGCGTTCCCAT
m*Mmp9*	GTATGGTCGTGGCTCTAAGC	AAAACCCTCTTGGTCTGCGG
r*Pde4d*	CCATGTGCAACCAACCATCC	GGTGAGCTCCCGATTAAGCA
r*Mmp2*	CAGACAAAGAGTTGGCAGTG	GTTGTAGTTGGCCACATCTG
r*Mmp9*	AGGCAGAGGATTACCTGTAC	ATGATGGTGCCACTTGAGGT
r*Sm22α*	CCATCGTGGGACGTCCCAG	CCTTGATGTCACGGACGATCTCAC
r*Cnn*	GGCACCAGCTGGAGAACATAGG	GGCAGCCCATACACCGTCATG
r*Opn*	CAGTCGATGTCCCTGACGG	GTTGCTGTCCTGATCAGAGG
r*Col1a1*	GACTGTCCCAACCCCCAAAA	CTTGGGTCCCTCGACTCCTA
r*Col3a1*	GGACCAGGCAATGATGGGAA	CAGGGAAACCCATGACACCA
r*Fn*	GGATCCCCTCCCAGAGAAGT	GGGTGTGGAAGGGTAACCAG
r*Tnf-α*	AAATGGGCTCCCTCTCATCAGTTC	TCTGCTTGGTGGTTTGCTACGAC
r*Il-6*	GGATACCACCCACCACAGACCAG	CGATGAGTTTTCTGACAGTGCATCATC
r*Ccl2*	GCAGCTCAGCAGAGGTAGTTGG	CGGTATAGGGTCTGAGAAGATTACCG
Pde4d flox	TATTGCCCAGGAAACAGTAACA	GACATCGGCCTTTTCTTCTTCTCC
Pde4d Ko	TTGCCCAGGAAACAGTAACAGAAG	AAACAAGCCACCAAACAGCAACAA
Tagln-Cre	TCGATGCAACGAGTGATGAG	TCCATGAGTGAACGAACCTG

Supplementary Table 3. Statistics of each figure

	Figures	Shapiro-Wilk normality test	Brown-Forsythe test	Test	Plot
**Figure 1**					
	Figure 1a human *PDE4A* mRNA, non-AAA vs AAA	Accept normality	Reject constancy of varaince (F test to compare variances)	Parametric: Welch's t-test	Mean with SE
	Figure 1a human *PDE4B* mRNA, non-AAA vs AAA	Reject normality	Accept constancy of varaince (F test to compare variances)	Non-parametric: Mann-Whitney test	Mean with SE
	Figure 1a human *PDE4C* mRNA, non-AAA vs AAA	Reject normality	Reject constancy of varaince (F test to compare variances)	Non-parametric: Mann-Whitney test	Mean with SE
	Figure 1a human *PDE4D* mRNA, non-AAA vs AAA	Accept normality	Accept constancy of varaince (F test to compare variances)	Parametric: unpaired Student's t-test	Mean with SE
	Figure 1b mouse *Pde4a* mRNA, control vs AAA	Accept normality	Accept constancy of varaince (F test to compare variances)	Parametric: unpaired Student's t-test	Mean with SE
	Figure 1b mousen *Pde4b* mRNA, control vs AAA	Accept normality	Accept constancy of varaince (F test to compare variances)	Parametric: unpaired Student's t-test	Mean with SE
	Figure 1b mouse *Pde4c* mRNA, control vs AAA	Accept normality	Accept constancy of varaince (F test to compare variances)	Parametric: unpaired Student's t-test	Mean with SE
	Figure 1b mouse *Pde4d* mRNA, control vs AAA	Accept normality	Accept constancy of varaince (F test to compare variances)	Parametric: unpaired Student's t-test	Mean with SE
	Figure 1d human PDE4D protein, non-AAA vs AAA	Reject normality	Reject constancy of varaince (F test to compare variances)	Non-parametric: Mann-Whitney test	Mean with SE
	Figure 1f mouse PDE4D protein, control vs AAA	Accept normality	Accept constancy of varaince (F test to compare variances)	Parametric: unpaired Student's t-test	Mean with SE
	Figure 1h human PDE4D staining, non-AAA vs AAA	Accept normality	Accept constancy of varaince (F test to compare variances)	Parametric: unpaired Student's t-test	Mean with SE
	Figure 1j mouse PDE4D staining, control vs AAA	Accept normality	Reject constancy of varaince (F test to compare variances)	Parametric: Welch's t-test	Mean with SE
**Figure 2**					
	Figure 2c SMC treated with Ang II-*Pde4d* mRNA	Accept normality	Reject constancy of varaince (F test to compare variances)	Parametric: Welch's t-test	Mean with SE
	Figure 2e SMC treated with Ang II-PDE4D protein	Accept normality	Accept constancy of varaince (F test to compare variances)	Parametric: unpaired Student's t-test	Mean with SE
**Figure 3**					
	Figure 3c *Apoe* $^{-/-}$ *Pde4d* $^{SMC-/-}$ AAA model max diameter	Accept normality	Reject constancy of varaince	Parametric: Welch ANOVA with Dunnett's T3 post-hoc test	Mean with SE
	Figure 3e *Apoe* $^{-/-}$ *Pde4d* $^{SMC-/-}$ AAA model elastin degradation	Reject normality	Accept constancy of varaince	Non-parametric: Mann-Whitney test between *Apoe* $^{-/-}$ *Pde4d* $^{flox/flox}$ and Ang II *Apoe* $^{-/-}$ *Pde4d* $^{flox/flox}$; between Ang II *Apoe* $^{-/-}$ *Pde4d* $^{flox/flox}$ and Ang II *Apoe* $^{-/-}$ *Pde4d* $^{SMC-/-}$	Mean with SE
**Figure 4**					
	Figure 4c rolipram AAA model max diameter	Reject normality	Reject constancy of varaince	Parametric: Welch's t-test between ctrl and Ang II. Non-parametric: Mann-Whitney test between AngII and Ang II+rolipram	Mean with SE
	Figure 4e rolipram AAA model elastin degradation	Reject normality	Accept constancy of varaince	Non-parametric: Mann-Whitney test between ctrl and Ang II. Parametric: unpaired Student's t-test between AngII and Ang II+rolipram	Mean with SE
**Figure 5**					
	Figure 5c SMC treated with Ang II, siPDE4D-cleaved caspase-3 protein	Accept normality	Accept constancy of varaince	Parametric: two-way ANOVA with Holm-Sidak's post-hoc test	Mean with SE
	Figure 5e SMC treated with Ang II, siPDE4D-annexin V/PI flow	Accept normality	Accept constancy of varaince	Parametric: two-way ANOVA with Holm-Sidak's post-hoc test	Mean with SE
	Figure 5g SMC treated with Ang II, rolipram-cleaved caspase-3 protein	Accept normality	Accept constancy of varaince	Parametric: two-way ANOVA with Holm-Sidak's post-hoc test	Mean with SE
	Figure 5i SMC treated with Ang II, rolipram-annexin V/PI flow	Accept normality	Accept constancy of varaince	Parametric: two-way ANOVA with Holm-Sidak's post-hoc test	Mean with SE
**Figure 6**					
	Figure 6b *Apoe* $^{-/-}$ *Pde4d* $^{SMC-/-}$ AAA model-cleaved caspase-3 protein	Accept normality	Accept constancy of varaince	Parametric: two-way ANOVA with Holm-Sidak's post-hoc test	Mean with SE
	Figure 6c *Apoe* $^{-/-}$ *Pde4d* $^{SMC-/-}$ AAA model-TUNEL staining	Accept normality	Reject constancy of varaince	Parametric: Welch ANOVA with Dunnett's T3 post-hoc test	Mean with SE
	Figure 6e rolipram AAA model-cleaved caspase-3 protein	Accept normality	Accept constancy of varaince	Parametric: two-way ANOVA with Holm-Sidak's post-hoc test	Mean with SE
	Figure 6f rolipram AAA model-TUNEL staining	Reject normality	Reject constancy of varaince	Non-parametric: Mann-Whitney test between ctrl and Ang II. Non-parametric: Mann-Whitney test between AngII and Ang II+rolipram	Mean with SE
**Figure 7**					
	Figure 7b SMC treated with Ang II, siPDE4D, PKI-cleaved caspase3 protein	Accept normality	Accept constancy of varaince	Parametric: one-way ANOVA with Holm-Sidak's post-hoc test	Mean with SE
	Figure 7d SMC treated with Ang II, siPDE4D, ESI-09-cleaved caspase3 protein	Accept normality	Accept constancy of varaince	Parametric: one-way ANOVA with Holm-Sidak's post-hoc test	Mean with SE
	Figure 7f SMC treated with Ang II, siPDE4D, PKI-pBad protein	Accept normality	Accept constancy of varaince	Parametric: one-way ANOVA with Holm-Sidak's post-hoc test	Mean with SE
	Figure 7g SMC treated with Ang II, siPDE4D, PKI-pBad protein	Accept normality	Accept constancy of varaince	Parametric: one-way ANOVA with Holm-Sidak's post-hoc test	Mean with SE
	Figure 7h SMC treated with Ang II, siPDE4D, PKI-Bad protein	Accept normality	Accept constancy of varaince	Parametric: one-way ANOVA with Holm-Sidak's post-hoc test	Mean with SE

Supplementary Table 3. Statistics of each figure (continued)

	Supplemental Figures	Shapiro-Wilk normality test	Brown-Forsythe test	Test	Plot
**Supplemental Figure 3**					
	Supplemental Figure 3b mouse *Pde4a* mRNA, *Apoe* $^{-/-}$ *Pde4d* $^{flox/flox}$ vs *Apoe* $^{-/-}$ *Pde4d* $^{SMC-/-}$	Reject normality	Accept constancy of varaince (F test to compare variances)	Non-parametric: Mann-Whitney test	Mean with SE
	Supplemental Figure 3b mouse *Pde4b* mRNA, *Apoe* $^{-/-}$ *Pde4d* $^{flox/flox}$ vs *Apoe* $^{-/-}$ *Pde4d* $^{SMC-/-}$	Accept normality	Accept constancy of varaince (F test to compare variances)	Parametric: unpaired Student's t-test	Mean with SE
	Supplemental Figure 3b mouse *Pde4c* mRNA, *Apoe* $^{-/-}$ *Pde4d* $^{flox/flox}$ vs *Apoe* $^{-/-}$ *Pde4d* $^{SMC-/-}$	Accept normality	Accept constancy of varaince (F test to compare variances)	Parametric: unpaired Student's t-test	Mean with SE
	Supplemental Figure 3b mouse *Pde4d* mRNA, *Apoe* $^{-/-}$ *Pde4d* $^{flox/flox}$ vs *Apoe* $^{-/-}$ *Pde4d* $^{SMC-/-}$	Reject normality	Accept constancy of varaince (F test to compare variances)	Non-parametric: Mann-Whitney test	Mean with SE
**Supplemental Figure 4**					
	Supplemental Figure 4b Systolic blood pressure in *Apoe* $^{-/-}$ *Pde4d* $^{flox/flox}$ and *Apoe* $^{-/-}$ *Pde4d* $^{SMC-/-}$ mice	Accept normality	Accept constancy of varaince	Parametric: two-way ANOVA with Holm-Sidak's post-hoc test	Mean with SE
	Supplemental Figure 4c Diastolic blood pressure in *Apoe* $^{-/-}$ *Pde4d* $^{flox/flox}$ and *Apoe* $^{-/-}$ *Pde4d* $^{SMC-/-}$ mice	Reject normality	Accept constancy of varaince	Non-parametric: ANOVA Kruskal-Wallis test with post Dunn's multiple comparisons test	Mean with SE
**Supplemental Figure 5**					
	Supplemental Figure 5a *Apoe* $^{-/-}$ *Pde4d* $^{SMC-/-}$ AAA model-*Mmp2* mRNA	Accept normality	Accept constancy of varaince	Parametric: two-way ANOVA with Holm-Sidak's post-hoc test	Mean with SE
	Supplemental Figure 5b *Apoe* $^{-/-}$ *Pde4d* $^{SMC-/-}$ AAA model-*Mmp9* mRNA	Accept normality	Accept constancy of varaince	Parametric: two-way ANOVA with Holm-Sidak's post-hoc test	Mean with SE
	Supplemental Figure 5c rolipram AAA model-*Mmp2* mRNA	Accept normality	Reject constancy of varaince	Parametric: Welch ANOVA with Dunnett's T3 post-hoc test	Mean with SE
	Supplemental Figure 5d rolipram AAA model-*Mmp9* mRNA	Accept normality	Reject constancy of varaince	Parametric: Welch ANOVA with Dunnett's T3 post-hoc test	Mean with SE
**Supplemental Figure 6**					
	Supplemental Figure 6a *Apoe* $^{-/-}$ *Pde4d* $^{SMC-/-}$ AAA mode-cAMP	Accept normality	Accept constancy of varaince	Parametric: two-way ANOVA with Holm-Sidak's post-hoc test	Mean with SE
	Supplemental Figure 6b rolipram AAA model-cAMP	Accept normality	Accept constancy of varaince	Parametric: two-way ANOVA with Holm-Sidak's post-hoc test	Mean with SE
	Supplemental Figure 6c SMC treated with Ang II, siPDE4D-cAMP	Accept normality	Accept constancy of varaince	Parametric: two-way ANOVA with Holm-Sidak's post-hoc test	Mean with SE
	Supplemental Figure 6d SMC treated with Ang II, rolipram-cAMP	Accept normality	Accept constancy of varaince	Parametric: two-way ANOVA with Holm-Sidak's post-hoc test	Mean with SE
**Supplemental Figure 7**					
	Supplemental Figure 7b Systolic blood pressure in Apoe-/- mice with or without Ang II/rolipram	Accept normality	Accept constancy of varaince	Parametric: two-way ANOVA with Holm-Sidak's post-hoc test	Mean with SE
	Supplemental Figure 7c Diastolic blood pressure in Apoe-/- mice with or without Ang II/rolipram	Accept normality	Accept constancy of varaince	Parametric: two-way ANOVA with Holm-Sidak's post-hoc test	Mean with SE
**Supplemental Figure 9**					
	Supplemental Figure 9a SMC treated with siPDE4D-Pde4d mRNA	Accept normality	Accept constancy of varaince (F test to compare variances)	Parametric: unpaired Student's t-test	Mean with SE
	Supplemental Figure 9c SMC treated with siPDE4D-PDE4D protein	Accept normality	Accept constancy of varaince (F test to compare variances)	Parametric: unpaired Student's t-test	Mean with SE
**Supplemental Figure 10**					
	Supplemental Figure 10b SMC treated with H2O2, siPDE4D-cleaved caspase-3 protein	Accept normality	Accept constancy of varaince	Parametric: two-way ANOVA with Holm-Sidak's post-hoc test	Mean with SE
	Supplemental Figure 10d SMC treated with H2O2, siPDE4D-annexin V/PI flow	Accept normality	Accept constancy of varaince	Parametric: two-way ANOVA with Holm-Sidak's post-hoc test	Mean with SE
**Supplemental Figure 11**					
	Supplemental Figure 11b SMC treated with H2O2, rolipram-cleaved caspase-3 protein	Accept normality	Accept constancy of varaince	Parametric: two-way ANOVA with Holm-Sidak's post-hoc test	Mean with SE
	Supplemental Figure 11d SMC treated with H2O2, rolipram-annexin V/PI flow	Accept normality	Accept constancy of varaince	Parametric: two-way ANOVA with Holm-Sidak's post-hoc test	Mean with SE
**Supplemental Figure 17**					
	Supplemental Figure 17b SMC treated with Ang II, siPDE4D-EdU staining	Accept normality	Accept constancy of varaince	Parametric: two-way ANOVA with Holm-Sidak's post-hoc test	Mean with SE
**Supplemental Figure 18**					
	Supplemental Figure 18 SMC treated with Ang II, siPDE4D-*Sm22 α* mRNA	Accept normality	Accept constancy of varaince	Parametric: two-way ANOVA with Holm-Sidak's post-hoc test	Mean with SE
	Supplemental Figure 18 SMC treated with Ang II, siPDE4D-*Cnn* mRNA	Accept normality	Accept constancy of varaince	Parametric: two-way ANOVA with Holm-Sidak's post-hoc test	Mean with SE
	Supplemental Figure 18 SMC treated with Ang II, siPDE4D-*Opn* mRNA	Accept normality	Accept constancy of varaince	Parametric: two-way ANOVA with Holm-Sidak's post-hoc test	Mean with SE
**Supplemental Figure 19**					
	Supplemental Figure 19a SMC treated with Ang II, siPDE4D-*Mmp2* mRNA	Accept normality	Accept constancy of varaince	Parametric: two-way ANOVA with Holm-Sidak's post-hoc test	Mean with SE
	Supplemental Figure 19b SMC treated with Ang II, siPDE4D-*Mmp9* mRNA	Accept normality	Accept constancy of varaince	Parametric: two-way ANOVA with Holm-Sidak's post-hoc test	Mean with SE
**Supplemental Figure 20**					
	Supplemental Figure 20a SMC treated with Ang II, siPDE4D-*Col1a1* mRNA	Accept normality	Accept constancy of varaince	Parametric: two-way ANOVA with Holm-Sidak's post-hoc test	Mean with SE
	Supplemental Figure 20b SMC treated with Ang II, siPDE4D-*Col3a1* mRNA	Accept normality	Accept constancy of varaince	Parametric: two-way ANOVA with Holm-Sidak's post-hoc test	Mean with SE
	Supplemental Figure 20c SMC treated with Ang II, siPDE4D-*Fn* mRNA	Accept normality	Accept constancy of varaince	Parametric: two-way ANOVA with Holm-Sidak's post-hoc test	Mean with SE
**Supplemental Figure 21**					
	Supplemental Figure 21a SMC treated with Ang II, siPDE4D-*Tnf-α* mRNA	Accept normality	Accept constancy of varaince	Parametric: two-way ANOVA with Holm-Sidak's post-hoc test	Mean with SE
	Supplemental Figure 21b SMC treated with Ang II, siPDE4D-*Il-6* mRNA	Accept normality	Accept constancy of varaince	Parametric: two-way ANOVA with Holm-Sidak's post-hoc test	Mean with SE
	Supplemental Figure 21c SMC treated with Ang II, siPDE4D-*Ccl2* mRNA	Accept normality	Accept constancy of varaince	Parametric: two-way ANOVA with Holm-Sidak's post-hoc test	Mean with SE

Supplementary Dataset 1. RNA sequencing data. List of genes upregulated in rat aortic smooth muscle cells (SMCs) with Ang II (100nM, 24h) stimulation (Supplementary Table 4) and list of genes downregulated in SMCs after PDE4D suppression (Supplementary Table 5). List of MetaCore pathways (based on their minimum FDR) enriched for both the up-regulated genes in Ang II (100 nM, 24h) stimulated SMCs (Supplementary Table 6) and the down-regulated genes in PDE4D siRNA (200 nM) treated SMCs Supplementary Table 7).

Supplementary Table 4. The 1848 genes up-regulated under AngII.

Symbol	Gene id	A1A	A2A	A3A	con1A	con2A	con3A
Gtf2h2	ENSRNOG00000018230	12.58204	8.702019	11.29785	10.46781	6.577485	9.178095
Ldha	ENSRNOG00000013009	937.6022	394.1554	1469.684	818.3751	277.1435	1350.952
Gpr62	ENSRNOG00000022567	0.174323	0.097022	0.096954	0.074483	0	0
Srsf5	ENSRNOG00000005513	150.3875	141.4785	160.7884	134.9432	126.5325	145.4476
Exosc10	ENSRNOG00000010719	30.39819	19.60395	25.13462	27.96985	17.24051	22.69231
Tsr2	ENSRNOG00000002556	25.09823	18.70887	19.2965	22.25717	15.97165	16.48866
Tldc2	ENSRNOG00000006395	0.177958	0.11005	0.219946	0.063363	0	0.107338
Tmem87a	ENSRNOG00000008455	16.93313	14.72039	14.4749	15.43866	13.29584	13.01729
Fam96a	ENSRNOG00000017119	37.42143	26.50822	36.10881	32.36692	21.45005	30.76575
Wdr73	ENSRNOG00000010664	11.76358	10.12928	11.82456	9.78205	8.078354	9.902044
Mettl16	ENSRNOG00000002764	10.00159	8.747772	9.726619	9.321418	8.068791	9.000802
Stk26	ENSRNOG00000007879	0.033656	0.020813	0.010399	0.023967	0.0111	0
Axl	ENSRNOG00000020716	280.9993	216.2972	276.8463	260.6302	195.4885	257.6567
Nfe2l2	ENSRNOG00000001548	156.6745	147.0991	139.724	143.6589	133.6746	125.596
Slc18b1	ENSRNOG00000016371	7.707378	11.85556	8.864634	5.898861	10.14275	7.000275
RGD1304810	ENSRNOG00000011253	0.015074	0.013983	0.013973	0	0	0
Lrrc1	ENSRNOG00000005970	4.924686	4.767819	4.823156	4.381434	4.271193	4.306673
Sec22a	ENSRNOG00000043069	15.15745	13.08023	15.15734	12.87905	10.72482	12.65391
Cldn12	ENSRNOG00000039862	6.626955	7.018244	6.343011	6.156434	6.588653	5.907805
Svbp	ENSRNOG00000007404	28.11799	29.54117	33.3427	23.70932	25.56274	29.13047
Cr1l	ENSRNOG00000008193	47.87516	59.09034	63.39977	41.51639	52.23897	57.18674
Nmd3	ENSRNOG00000009310	33.67873	29.24819	35.2949	30.5634	25.86308	32.23062
Ip6k1	ENSRNOG00000019932	44.78013	63.74343	49.37313	42.43129	61.30201	47.18496
Tigd5	ENSRNOG00000008686	3.352602	3.579844	2.94198	3.040012	3.287534	2.615329
Pold2	ENSRNOG00000014098	17.66496	6.690335	12.05474	15.47944	4.742631	9.918632
Sdhb	ENSRNOG00000007967	88.12555	66.71187	79.69983	79.97255	57.77299	71.72319
Stk11	ENSRNOG00000014287	51.42993	45.65206	49.24693	48.6221	42.49868	46.14964
Ddx23	ENSRNOG00000060154	64.81726	41.61905	51.83169	59.21532	36.08238	46.83404
Pmf1	ENSRNOG00000019620	52.34522	18.39146	56.35487	47.41438	12.94741	51.49394
Nabp1	ENSRNOG00000015416	4.813148	3.012156	3.987929	3.838356	2.152713	3.05721
Kdm4d	ENSRNOG00000024726	0.193552	0.164579	0.134561	0.103374	0.063833	0.043779
Parp3	ENSRNOG00000012865	98.18061	76.78785	70.16209	91.99008	70.02144	64.20963
Poldip3	ENSRNOG00000022877	54.96015	48.8388	51.36084	51.67438	45.94997	48.16888
Acot9	ENSRNOG00000003782	44.28577	44.1936	54.8707	38.3039	38.52486	49.63896
Gstk1	ENSRNOG00000016484	6.127267	4.262779	4.223385	5.580025	3.769465	3.660198
AABR07049994.1	ENSRNOG00000047185	87.46388	80.1181	94.91417	79.09055	70.49664	85.78352
Eipr1	ENSRNOG00000009285	22.41028	17.66978	22.51927	20.34739	15.30841	20.21083
Mpz	ENSRNOG00000003171	0.020144	0.037372	0.018673	0	0.019931	0
Tufm	ENSRNOG00000018604	53.39393	44.14874	49.34619	50.66855	41.03525	46.5767
Arfgap2	ENSRNOG00000014429	51.07228	46.21406	57.92259	44.81751	40.40802	52.53401
Zfp637	ENSRNOG00000023065	29.23087	31.32923	28.89189	27.01295	29.38335	26.93001
Oser1	ENSRNOG00000008297	36.69207	31.62931	28.62251	34.64076	29.24379	26.3647
Anks1a	ENSRNOG00000000498	3.406357	3.484316	3.353371	2.954588	3.095884	2.938534
Rbck1	ENSRNOG00000006695	57.3464	59.21256	57.23268	50.82111	51.73902	50.62704
Ctnna1	ENSRNOG00000005796	142.7676	143.2354	167.2711	130.4762	132.4874	156.4377
Mut	ENSRNOG00000050843	9.22322	10.48054	8.426236	8.535252	9.748306	7.79903
Txndc15	ENSRNOG00000000133	51.52207	59.28813	57.80491	45.51494	53.90624	52.62247
Zscan29	ENSRNOG00000012419	3.04252	2.836035	2.847806	2.441421	2.320078	2.255869
Ptcd1	ENSRNOG00000000987	7.983751	6.706924	6.330507	6.710803	5.205894	4.936431
Cdc25a	ENSRNOG00000020737	10.38944	6.723722	8.286782	9.572679	6.008858	7.581421
Lsp1	ENSRNOG00000020300	23.82576	16.56868	28.91141	23.14234	15.9862	28.24603
Ccdc191	ENSRNOG00000057815	2.319378	1.754601	1.909923	2.164807	1.570685	1.741909
Ifi27	ENSRNOG00000009263	1794.736	1001.76	1245.358	1628.991	857.0245	1104.569

Hars	ENSRNOG00000028105	62.91966	46.48942	54.53082	55.61567	40.35092	47.49425
Akr1b1	ENSRNOG00000009513	223.4707	191.6221	187.7159	191.7621	153.6558	153.6625
Rimkla	ENSRNOG00000008625	0.131406	0.243786	0.121808	0	0.097508	0
Eif2b1	ENSRNOG00000001039	13.71705	12.39675	13.95532	11.54274	10.24042	12.13107
St3gal4	ENSRNOG00000009850	64.41165	60.3578	55.4301	59.14089	55.24777	49.33328
Dnpep	ENSRNOG00000019772	124.2291	122.1937	109.9354	112.1203	107.9556	97.85348
AABR07013288.4	ENSRNOG00000053387	5.17555	1.567636	2.545625	4.174501	0.627014	1.720117
Thap7	ENSRNOG00000037967	13.39561	10.53841	10.04202	11.7731	8.593292	8.130856
Ifngr1	ENSRNOG00000012074	96.88631	77.29374	68.05098	94.68194	75.41927	66.18683
Tfg	ENSRNOG00000001633	34.70578	34.25687	40.83759	31.587	31.06089	38.19427
Haus8	ENSRNOG00000052038	21.64327	8.030593	15.90748	18.93279	4.890905	13.30158
Cript	ENSRNOG00000015215	33.63668	35.34242	33.88159	29.92753	31.66895	29.50603
Gtf2b	ENSRNOG00000011135	47.68395	34.24699	43.26745	40.31078	25.94003	36.47824
Cog7	ENSRNOG00000060008	13.26754	11.61443	12.47151	12.24921	10.36945	11.37638
LOC498122	ENSRNOG00000048861	6.61392	6.428443	6.301812	5.995248	5.891956	5.793174
Adss	ENSRNOG00000004481	83.97166	60.97811	82.88312	77.54839	55.22111	77.65866
Ap3s2	ENSRNOG00000043141	49.80266	46.11298	49.11712	43.21929	39.55129	43.65909
Lrrn4	ENSRNOG00000032989	1.564548	0.881624	4.635448	1.405691	0.752271	4.484664
Snx17	ENSRNOG00000026884	60.02096	58.63746	59.34101	55.0922	52.557	53.72583
Ggh	ENSRNOG00000007351	8.769793	7.130941	8.077789	8.004024	6.507534	7.407121
Rpl6	ENSRNOG00000025936	559.5116	560.9447	570.6795	517.93	509.5887	524.772
Slamf8	ENSRNOG00000008736	3.478425	1.751198	2.866095	3.334482	1.632509	2.743608
Pus1	ENSRNOG00000037500	18.47991	16.36116	17.83249	15.64793	13.01521	14.33921
Dync1i2	ENSRNOG00000009781	35.89863	36.26317	42.23422	34.40763	34.4509	40.42718
Isoc2b	ENSRNOG00000016829	24.0257	15.28023	18.73684	20.8229	11.35157	14.87102
Cers5	ENSRNOG00000052990	57.54707	52.83017	58.10264	53.73556	48.17685	54.17657
Nhp2	ENSRNOG00000004247	129.3359	65.32104	139.017	111.414	45.01583	116.6077
Nod1	ENSRNOG00000010629	28.84065	21.9599	32.76106	26.38371	18.88527	29.94658
Pex16	ENSRNOG00000006539	20.05648	20.51209	18.93811	16.57423	16.59359	15.8022
AABR07011031.1	ENSRNOG00000059120	8.384761	6.988727	11.71482	4.802702	2.524799	7.805937
Med29	ENSRNOG00000019702	58.78064	45.83921	59.68674	50.55722	37.47166	49.6583
Acox3	ENSRNOG00000008474	8.178592	12.35329	9.808623	7.545574	11.58629	9.023488
Bola1	ENSRNOG00000021185	26.62667	26.8002	23.28211	21.55996	20.51749	17.94248
LOC100912489	ENSRNOG00000058444	1623.519	375.4371	889.9091	1495.605	250.5285	736.5025
Zbtb8os	ENSRNOG00000008226	32.52415	22.68607	28.84518	29.05383	18.51829	25.4636
Bcl7b	ENSRNOG00000032705	19.0184	18.91252	20.75046	16.96635	17.18532	19.09761
Ppox	ENSRNOG00000003567	12.08717	11.55893	12.80218	10.55968	9.719158	10.87941
Plin2	ENSRNOG00000007060	57.57536	47.52735	45.95958	51.96926	40.42625	39.28734
RGD1311739	ENSRNOG00000021245	9.524055	9.457401	8.667054	7.889	7.528534	7.131937
LOC100911576	ENSRNOG00000050800	96.33952	71.5003	115.6623	84.65933	56.61045	102.1119
Stard5	ENSRNOG00000025052	27.42775	18.66149	26.4379	25.26003	15.91727	24.08021
Cox6b2	ENSRNOG00000038616	0.479351	0.518758	0.592451	0	0.079044	0.216845
Cep44	ENSRNOG00000010566	12.62055	9.945319	13.7488	11.06722	8.728728	12.35357
Snrpd2	ENSRNOG00000015844	95.18648	78.88509	94.4909	80.95208	60.78463	78.7324
Nutf2	ENSRNOG00000018945	53.31316	36.27693	53.5735	47.4773	31.52114	48.81648
Sgce	ENSRNOG00000046905	41.86463	43.9307	38.51812	39.8952	41.59636	36.00502
Nln	ENSRNOG00000011561	24.19236	24.24963	25.09476	22.95708	22.72717	23.54035
Rab5c	ENSRNOG00000018568	110.4446	82.83256	95.97318	100.8823	71.03196	83.88898
Ints14	ENSRNOG00000012469	11.67202	10.93457	14.47202	9.815047	9.17299	12.2528
Ddx49	ENSRNOG00000022368	69.378	51.55471	58.32723	63.57535	45.07842	50.888
RGD1561590	ENSRNOG00000020771	121.0399	77.71522	123.533	110.2033	69.29054	113.8956
Ccm2	ENSRNOG00000060825	31.88563	26.56536	34.40403	26.96119	21.21825	30.24886

Ncoa4	ENSRNOG00000019768	69.821	59.92167	61.16808	63.34169	54.79124	54.77029
Thnsl1	ENSRNOG00000033076	0.970735	1.278917	0.938955	0.586082	0.904754	0.636424
Alg6	ENSRNOG00000009045	9.214817	8.227775	10.0948	8.237621	7.459374	9.273284
Eif3k	ENSRNOG00000020495	164.6578	147.2872	152.2061	146.102	124.1102	133.3253
Ddx21	ENSRNOG00000043099	69.68024	52.73941	66.36285	61.20752	44.67403	59.76956
Bdh1	ENSRNOG00000001736	6.609713	10.64492	11.63809	5.53473	9.3657	10.63795
LOC100909548	ENSRNOG00000014992	31.54392	24.60813	32.05381	28.77772	22.18263	29.92728
Ccng1	ENSRNOG00000003256	168.6639	236.0093	180.3943	152.8949	221.4126	161.6281
Tomm22	ENSRNOG00000014058	78.37929	70.43961	82.58946	65.4206	59.51311	72.52925
Uxt	ENSRNOG00000009893	45.27354	32.11079	55.09375	39.49787	25.4747	47.56125
LOC103691744	ENSRNOG00000057089	1.552361	1.210421	1.689232	0.961272	0.756813	1.139874
Hsd17b4	ENSRNOG00000015840	35.66395	43.61368	35.91501	31.88888	40.64435	32.83948
Cmc2	ENSRNOG00000011279	45.84125	34.83092	53.37	41.78027	29.72049	49.25986
Sumo2	ENSRNOG00000003670	337.8106	222.1846	336.5852	298.918	175.7159	300.6252
Pdcd5	ENSRNOG00000013250	51.49434	34.83092	56.47451	44.68771	28.10328	47.98525
Brms1l	ENSRNOG00000051077	0.36693	0.267431	0.194359	0.307957	0.216092	0.126467
Eif2s1	ENSRNOG00000009432	126.6363	75.47963	121.431	114.7346	59.67839	107.468
Poll	ENSRNOG00000016748	15.88681	17.66588	14.45343	13.92894	15.75056	12.00503
LOC691716	ENSRNOG00000054854	22.9793	21.22509	21.30087	20.15898	18.96229	18.3132
Minpp1	ENSRNOG00000011287	36.34757	34.25896	36.47723	32.44189	29.15736	32.27013
Snrpa	ENSRNOG00000001501	37.7787	15.11875	41.09905	31.70192	7.332784	34.89209
Prelid3b	ENSRNOG00000046644	58.23949	40.04371	54.39999	52.66019	32.60155	47.72302
Tmlhe	ENSRNOG00000000729	9.261452	8.964525	11.7577	7.903527	7.943059	10.49681
AABR07053687.1	ENSRNOG00000015756	16.53024	13.09074	13.27019	14.15388	10.57555	11.37103
Lsm4	ENSRNOG00000019572	65.12366	37.50728	82.79444	58.77672	29.15772	74.69435
Psmf1	ENSRNOG00000009640	35.02597	20.15864	24.09295	33.09577	18.11897	22.55821
Gpd1	ENSRNOG00000056457	0.143866	0.24264	0.157605	0.027941	0.142339	0.070998
Nit1	ENSRNOG00000003881	92.93482	89.63727	69.71386	83.35481	78.65922	61.52409
Cers2	ENSRNOG00000021138	45.79911	39.37352	40.31315	43.45272	37.48096	37.77359
Utp4	ENSRNOG00000020333	24.54562	18.23963	20.2743	22.7598	15.83953	18.0755
Pnp	ENSRNOG00000009982	285.1357	282.354	321.7384	252.7894	240.0576	287.8833
Ccdc175	ENSRNOG00000004774	1.917758	2.088308	2.0482	1.232069	1.429927	1.533909
LOC100912034	ENSRNOG00000047914	28.60476	25.97288	31.36192	27.50863	25.09483	30.18024
Usp47	ENSRNOG00000026754	35.67595	33.92566	39.99745	34.39318	32.64043	39.02106
Fam131a	ENSRNOG00000045653	4.91715	5.20728	5.07836	4.130833	4.618113	4.31708
Manbal	ENSRNOG00000031453	58.52535	59.49879	62.38177	52.03437	50.97489	55.58536
Gsto2	ENSRNOG00000012801	0.409784	0.880276	0.539791	0.230379	0.746853	0.390262
Mrpl46	ENSRNOG00000018547	13.5917	10.89154	11.56553	10.97108	8.730276	8.627209
Cxcl16	ENSRNOG00000026647	136.5424	117.2402	76.2273	120.9858	100.9712	64.11902
Ap1s2	ENSRNOG00000038686	4.404441	6.454291	5.020019	3.386661	5.47679	3.7368
Ssh3	ENSRNOG00000018878	26.17528	29.27666	22.90176	24.61237	27.43387	21.5428
Ift88	ENSRNOG00000009278	2.867083	4.011645	2.869835	2.534964	3.718636	2.62667
Oit3	ENSRNOG00000046365	0.131406	0.060946	0.15226	0.087728	0.016251	0.118888
Nsg1	ENSRNOG00000005700	8.011109	10.27762	27.48912	6.105184	7.694324	25.02889
AABR07018321.1	ENSRNOG00000016777	34.36737	34.22499	31.38837	31.48853	31.43834	27.71928
Txndc17	ENSRNOG00000014072	39.60447	37.32408	44.13711	35.15524	31.28472	39.14565
Trap1	ENSRNOG00000005418	50.65355	35.20494	49.67911	45.58255	29.13871	42.7124
LOC103689947	ENSRNOG00000053812	43.99094	48.06355	27.39867	40.92679	44.42925	24.74861
Nol3	ENSRNOG00000015588	42.23659	55.30616	43.74954	36.55911	48.14365	38.39276
Smg9	ENSRNOG00000019596	16.73165	18.17862	16.53781	14.94956	16.20652	14.1175

Mccc2	ENSRNOG00000017752	19.86504	20.18343	15.64021	18.79943	19.21929	14.86767
Vasp	ENSRNOG00000016367	53.8454	56.61264	48.85474	49.99348	51.31582	43.95754
Toe1	ENSRNOG00000017561	10.52815	8.975592	10.25063	9.142357	7.45635	9.14837
LOC100912481	ENSRNOG00000046950	83.22396	52.2067	86.23137	74.3525	41.90656	78.77863
LOC100361025	ENSRNOG00000004090	7.566106	4.00222	7.158377	6.111538	2.691176	6.109926
Spcs1	ENSRNOG00000018075	102.1457	83.14092	106.6049	91.5274	71.0781	97.92249
Rai14	ENSRNOG00000028872	20.53419	22.05367	20.67283	19.39965	21.12764	19.38497
Tcaf2	ENSRNOG00000030222	0.906447	0.77737	0.753045	0.703347	0.575323	0.487419
Plaur	ENSRNOG00000037931	42.82434	27.21909	37.21751	37.29164	23.23272	32.58028
Ecsit	ENSRNOG00000014128	15.01461	11.25919	12.56122	13.181	9.737055	10.4337
Tef	ENSRNOG00000019383	24.88544	20.42332	19.00438	23.89394	19.67742	17.97766
Acad9	ENSRNOG00000014178	23.23052	18.33691	22.05992	20.7215	15.13543	19.69521
C1d	ENSRNOG00000005982	9.095109	8.74798	10.45805	7.688084	7.418673	8.657968
Lrrc41	ENSRNOG00000013642	45.5854	36.48223	42.39724	41.45372	31.60255	36.61832
Cacybp	ENSRNOG00000002572	53.82837	41.23968	54.88479	46.67301	31.67543	47.54896
Timm50	ENSRNOG00000037638	25.46261	18.51026	23.13681	23.18294	15.43978	20.76306
Mrps28	ENSRNOG00000032630	14.67885	11.78594	16.1285	11.6559	9.051024	12.36095
Cdca7	ENSRNOG00000001514	16.61509	4.140843	12.26444	14.54599	2.120614	10.76033
Dpm2	ENSRNOG00000049110	59.22986	40.56475	48.87451	50.06241	33.77328	41.77677
Phkg1	ENSRNOG00000000920	1.003595	1.203415	0.884912	0.810548	0.944507	0.686533
P2ry6	ENSRNOG00000019270	17.09134	16.17907	34.77828	14.18868	12.29813	30.76114
Dnaja1	ENSRNOG00000007029	112.5308	96.05544	111.77	101.9667	85.79252	97.9922
Atrip	ENSRNOG00000020670	6.965104	6.539843	7.034941	5.803464	5.333295	5.4674
Gorasp1	ENSRNOG00000018047	20.78021	23.43044	19.22047	17.17119	19.50034	16.42678
Pef1	ENSRNOG00000013972	57.79886	55.00382	60.84926	50.23182	44.55862	52.55599
Esm1	ENSRNOG00000010797	132.1218	97.39192	100.0147	114.9928	85.0036	86.4551
Aldh16a1	ENSRNOG00000020623	29.64305	29.87037	34.89568	25.15035	23.72348	28.71636
Uri1	ENSRNOG00000014463	19.29673	15.12282	18.01635	18.4928	14.38495	16.99666
Slc35b4	ENSRNOG00000008851	20.10154	24.63744	24.30306	17.26395	20.90363	21.53519
AABR07045058.1	ENSRNOG00000033891	21.2364	16.40052	21.73679	17.58423	12.44914	18.9435
Comtd1	ENSRNOG00000013968	21.93078	32.4012	17.75012	18.59475	28.5712	13.05609
Aifm2	ENSRNOG00000059445	19.34616	16.457	20.82685	17.17785	13.46383	18.48786
Phf5a	ENSRNOG00000024170	59.01195	38.14245	49.31921	51.08966	30.25668	43.58419
Arl6ip5	ENSRNOG00000006818	118.18	97.76214	104.7278	106.3762	83.00559	94.17608
Dph3	ENSRNOG00000019727	7.794749	7.155271	8.782151	6.891038	6.222326	7.555226
Cdc6	ENSRNOG00000027787	21.26143	5.241767	12.92977	18.174	2.338661	10.74862
Pno1	ENSRNOG00000005524	20.47458	13.02994	17.18471	18.66277	10.82843	14.59485
AABR07036019.1	ENSRNOG00000051442	2.008178	2.03854	1.767839	1.537986	1.674464	1.428369
RGD735065	ENSRNOG00000000524	14.41845	11.38874	14.80715	13.2919	10.20194	13.97
B3galt6	ENSRNOG00000019979	8.890907	10.13796	8.632408	8.258299	9.49192	7.773731
Stip1	ENSRNOG00000021164	103.9974	59.15122	96.16938	97.20862	49.40447	87.72005
Rangrf	ENSRNOG00000004980	22.25135	15.97978	13.05764	20.46185	13.44879	10.63411
Tmem129	ENSRNOG00000017329	24.2899	18.6796	24.74503	22.19528	16.35452	21.80205
Lrrc8e	ENSRNOG00000028460	7.818041	8.112105	9.302644	6.838748	6.755783	8.263537
Pla2g5	ENSRNOG00000016838	2.715748	4.288804	1.872437	2.109741	3.863841	1.299599
B4galt1	ENSRNOG00000059461	38.09001	31.01487	37.13101	36.64262	28.96158	35.15861
Rnf167	ENSRNOG00000003879	20.50155	17.40609	17.93687	17.82145	13.54928	14.43686
Loxl3	ENSRNOG00000061373	111.3324	59.82684	103.9624	101.3763	48.72097	89.91298
St6galnac4	ENSRNOG00000048870	24.48857	21.94101	22.77029	20.99406	19.45805	20.02615
Mdp1	ENSRNOG00000019840	12.82021	7.866824	14.23486	11.34483	6.038199	12.10096
Acp6	ENSRNOG00000017494	17.0226	16.86642	15.05503	16.36227	16.02208	14.45858
Wdr18	ENSRNOG00000012379	30.98644	19.64644	25.52593	28.77735	16.47244	22.95746

Mapkapk5	ENSRNOG00000001345	25.84165	20.93014	25.74972	23.39695	18.04879	23.76522
Eapp	ENSRNOG00000004509	38.56487	26.44232	30.82777	35.59416	22.30333	26.62054
Gnpda2	ENSRNOG00000002177	5.789516	5.830726	4.734147	4.682335	4.725656	3.947224
Epha2	ENSRNOG00000009222	26.12031	20.7045	25.35993	24.35449	19.29211	24.12703
Plekhf2	ENSRNOG00000026662	15.26463	14.59942	16.11526	14.82811	14.12531	15.78861
Atp6v1h	ENSRNOG00000030862	38.59871	40.06814	36.73556	35.27664	37.32965	34.44711
RGD1565784	ENSRNOG00000029415	11.22986	9.992144	11.60217	9.456607	7.421492	9.509093
Atp1b3	ENSRNOG00000011501	149.2672	174.4036	158.7545	137.3371	159.0855	141.2897
Usp39	ENSRNOG00000010930	17.70422	12.75971	16.86695	16.5473	11.06218	15.38962
Thap3	ENSRNOG00000026840	20.52979	19.97399	18.88501	17.37613	16.68366	14.4907
Cd82	ENSRNOG00000000047	112.3155	115.3269	126.2265	100.2948	106.3411	113.0847
Triobp	ENSRNOG00000059015	10.63681	8.70901	10.17217	10.25291	8.163408	9.628572
LOC100363116	ENSRNOG00000052273	10.45069	8.435612	10.43516	8.852194	7.147139	8.542844
LOC108348189	ENSRNOG00000004484	25.69335	23.44637	24.23565	22.17139	20.53603	21.83036
Rab21	ENSRNOG00000003923	31.75093	31.3079	29.15017	29.42593	29.40985	27.55813
Mpzl3	ENSRNOG00000026753	4.119714	3.860378	4.304659	3.751126	3.505557	4.049743
Slc24a4	ENSRNOG00000006729	0.21663	0.334913	0.312366	0.167121	0.273867	0.239548
B3galnt1	ENSRNOG00000012019	43.73441	32.52484	43.3791	40.30025	27.46941	39.19052
Rtel1	ENSRNOG00000027513	19.92736	20.33725	22.46812	18.18228	18.67282	21.2705
Slc25a3	ENSRNOG00000008289	347.0821	337.217	373.9524	323.7471	302.7561	342.8011
Scfd2	ENSRNOG00000026828	7.332938	7.279438	6.454486	6.493029	6.634605	5.499587
Ube2t	ENSRNOG00000005038	34.37426	13.64919	37.74951	27.84808	9.061124	31.1551
LOC100359574	ENSRNOG00000025154	120.1185	78.66207	105.229	104.0555	60.09375	92.69966
Ankrd46	ENSRNOG00000025504	35.28513	33.49306	31.41759	32.94353	30.28131	27.97899
Cmtm6	ENSRNOG00000010951	46.00751	43.31921	57.53666	40.46651	38.08679	53.75644
Nit2	ENSRNOG00000027797	73.71029	94.56526	73.74378	61.29506	85.28739	64.97041
Hspe1	ENSRNOG00000051624	349.6149	278.5169	362.405	326.5808	250.1753	328.2718
Zfp512	ENSRNOG00000039815	10.16233	9.052924	9.264448	9.575962	8.548516	8.525989
RGD1310852	ENSRNOG00000006370	3.747144	3.091454	2.977242	3.227907	2.366226	2.445007
AABR07053500.2	ENSRNOG00000060434	1.072833	0.884595	0.662981	0.636652	0.235877	0.107849
AABR07071891.4	ENSRNOG00000049295	8.58682	4.995131	6.070905	7.617611	4.031849	5.398702
Tut1	ENSRNOG00000020047	14.61917	12.11616	12.75066	13.1693	10.19084	11.40305
RGD1560212	ENSRNOG00000057774	51.73989	43.69013	52.5543	47.51973	37.39575	46.93356
Galnt12	ENSRNOG00000008099	11.43738	9.32828	12.12227	10.7649	8.390913	11.44125
Hmgb1	ENSRNOG00000058908	18.85065	14.12997	27.17447	15.3321	9.919095	24.35116
Soat1	ENSRNOG00000004111	71.48188	47.71407	68.77498	67.21143	42.42249	62.41021
LOC103689999	ENSRNOG00000037984	40.14082	40.79448	44.86298	33.60662	32.34031	39.0618
Plaa	ENSRNOG00000007753	36.4341	32.12874	38.28201	33.79642	29.6212	36.49439
Stx1a	ENSRNOG00000029165	1.201155	1.404863	1.387743	0.873957	1.171183	1.149735
Polr1c	ENSRNOG00000019079	42.31684	42.86479	48.0649	38.93381	38.37873	44.97312
AABR07055527.1	ENSRNOG00000039997	1.564548	2.293972	2.152016	1.347751	1.997345	1.826469
Med8	ENSRNOG00000028477	32.68166	31.60583	36.79393	28.34868	25.28305	30.51218
AABR07043200.1	ENSRNOG00000058276	0.715222	0.442297	0.441988	0.381991	0.117938	0.215697
Psma4	ENSRNOG00000013493	35.44547	26.54421	36.53394	31.80815	21.1366	31.37377
Stx5	ENSRNOG00000018847	40.48465	44.31245	43.53752	36.22899	40.18033	37.67908
Txndc12	ENSRNOG00000008090	97.28062	77.00213	92.91993	77.76412	60.15822	80.02317
Dpp8	ENSRNOG00000019105	47.78799	45.37554	56.39479	44.75938	41.50778	51.81554
Opa1	ENSRNOG00000001717	32.92932	31.96325	38.23029	30.21475	30.0023	35.28177

Ddx39a	ENSRNOG00000004373	55.8333	34.95108	72.39208	50.30929	26.8788	66.28704
Rpp40	ENSRNOG00000016226	8.648346	9.012373	8.879215	7.893181	8.502399	8.146423
Akt1	ENSRNOG00000028629	139.0468	126.705	173.7159	129.5333	114.4017	159.2962
Arpc5	ENSRNOG00000028062	73.62293	73.88318	84.27146	66.16773	62.56187	74.50598
Cops7a	ENSRNOG00000016778	46.53632	43.68955	43.41441	39.75211	39.2213	37.6321
Sdcbp	ENSRNOG00000009683	138.2616	123.5518	173.7603	125.5443	108.309	163.715
Higd1a	ENSRNOG00000019428	65.46462	25.48325	107.6399	55.42629	16.28386	101.003
Lamc2	ENSRNOG00000002667	1.143829	0.597589	0.572796	0.884759	0.260158	0.344957
Dcaf4	ENSRNOG00000008399	10.06013	7.954465	8.499467	9.021068	7.015093	7.120249
Psmb2	ENSRNOG00000011463	157.3326	101.8922	146.1165	142.5142	80.6891	130.7396
Syngr4	ENSRNOG00000021093	0.494377	0.349401	0.305512	0.402348	0.209627	0.191693
Psmg2	ENSRNOG00000017729	25.09582	16.58197	21.39242	23.20331	13.78721	19.28236
Sumo4	ENSRNOG00000032840	31.35392	21.34007	30.63954	27.39844	16.07119	24.57354
Nudcd2	ENSRNOG00000060914	37.88419	30.94601	45.56571	32.41446	23.72525	40.70616
Casp3	ENSRNOG00000010475	37.90022	29.13903	35.0612	34.22313	26.73032	32.06655
Fam35a	ENSRNOG00000059057	5.754573	8.511934	5.241574	4.687301	7.540035	4.543044
Psmb10	ENSRNOG00000019494	118.3024	49.79842	85.51361	105.7896	41.25359	76.30128
Dnajb6	ENSRNOG00000010353	40.01767	33.66467	39.83776	37.505	29.82364	36.28893
Map3k6	ENSRNOG00000008936	4.802898	4.663923	5.952164	4.520242	4.477937	5.684237
Phpt1	ENSRNOG00000016723	85.74215	66.09646	91.70285	79.46014	56.39872	83.35256
Slc47a1	ENSRNOG00000057404	8.455284	3.20858	5.272636	6.979058	2.072373	4.294351
Nop56	ENSRNOG00000007128	73.04944	36.29286	53.79854	66.75022	26.87864	46.69857
Mtif2	ENSRNOG00000004161	7.441661	8.167613	8.451508	5.719029	6.870895	7.298138
LOC103693210	ENSRNOG00000013238	18.77457	20.69896	19.71941	17.20019	18.40363	17.33153
Gys1	ENSRNOG00000020812	38.58681	19.6378	47.82007	35.3277	16.67802	45.70754
Gstcd	ENSRNOG00000011669	5.412991	3.340052	5.061829	4.797125	2.469367	4.113498
Ctbs	ENSRNOG00000015573	32.90197	37.22339	31.38187	27.52534	33.44934	27.60251
Mcrs1	ENSRNOG00000054838	30.33046	25.8041	30.37022	26.41276	22.66917	27.82014
Ndufb6	ENSRNOG00000024539	59.99506	45.35218	65.07547	54.39273	36.6781	57.35867
RGD1563365	ENSRNOG00000008696	17.90611	18.80694	20.4331	16.56312	16.87244	19.05764
Map2k1	ENSRNOG00000010176	107.3074	71.17395	127.3808	96.79012	56.54159	117.3435
Fam173b	ENSRNOG00000022347	17.27588	15.69666	13.79126	14.93175	14.04606	12.1541
Kcnn4	ENSRNOG00000019440	45.57085	51.43376	24.12254	39.88888	45.9879	20.40515
AABR07037489.1	ENSRNOG00000060137	1.742441	2.00557	2.266314	1.373998	1.769077	1.939625
Gcdh	ENSRNOG00000003307	24.63224	29.05363	20.7911	21.86211	24.98502	17.86491
Ss18	ENSRNOG00000016800	37.30763	33.67539	38.29424	34.78653	31.22714	36.64042
Wdr33	ENSRNOG00000011382	14.80391	15.0331	16.32253	14.0847	14.46616	15.85625
Gsdmd	ENSRNOG00000007728	95.73202	70.75197	83.07847	88.38809	60.55797	76.15447
Wwp2	ENSRNOG00000012937	28.95229	22.54094	39.24456	26.55174	21.00335	37.25513
Pou5f1	ENSRNOG00000046487	0.212592	0.172552	0.197065	0.113543	0.052584	0.120213
Nxt1	ENSRNOG00000004700	43.76327	27.88109	35.1742	37.04909	20.18865	30.26407
Tpd52l2	ENSRNOG00000015122	59.69142	54.81379	64.55763	54.90601	48.75174	60.64195
Pycr3	ENSRNOG00000054724	39.6736	28.27786	35.09124	33.54725	22.13335	31.03129
Prss46	ENSRNOG00000020981	0.384664	1.921327	0.713136	0.126427	1.75653	0.481877
Btg2	ENSRNOG00000003300	11.50511	13.86711	9.903963	10.37951	12.68805	8.247768
Dusp14	ENSRNOG00000030091	8.178035	8.117962	13.05822	6.791374	6.142431	11.70759
Slc25a39	ENSRNOG00000020994	104.9037	87.57143	107.9845	97.03893	81.6285	98.5785
Pkp4	ENSRNOG00000005504	5.722504	4.6457	7.377469	5.431435	4.383031	7.192962
AABR07053879.1	ENSRNOG00000027574	2.342022	1.068433	2.028601	1.927531	0.607781	1.736826
Akr7a2	ENSRNOG00000017780	78.54695	75.2004	64.65091	74.44505	68.72481	58.81705
Aass	ENSRNOG00000039494	22.37286	14.75398	20.12545	20.58095	11.96942	17.45647
Zfp410	ENSRNOG00000010985	19.61216	18.29812	18.34869	16.78616	16.10048	16.53766
RGD1309594	ENSRNOG00000001215	18.1018	14.91918	15.22055	13.60923	11.33655	12.36395

Micu1	ENSRNOG00000043436	39.46361	37.89019	35.26882	35.17705	32.51872	31.86178
Rsl24d1	ENSRNOG00000052787	55.46296	38.88102	42.32288	52.58151	35.20051	39.97673
Uqcrh	ENSRNOG00000012550	207.0351	222.7396	248.8634	193.1394	202.7323	226.8134
Ctnnb1	ENSRNOG00000054172	251.3902	265.0367	295.4426	227.9022	243.5893	280.6427
Cdc23	ENSRNOG00000024241	30.01178	24.55085	31.63214	25.77126	20.07359	28.78773
Plin3	ENSRNOG00000048834	122.3412	141.1221	136.7682	110.6425	123.92	124.8747
Twsg1	ENSRNOG00000013340	35.44639	54.96319	35.83331	33.23035	53.56189	33.74301
RGD1308430	ENSRNOG00000029146	35.69616	29.22035	34.93616	31.14614	22.69162	27.646
LOC103689927	ENSRNOG00000047396	21.02266	20.92861	17.70364	19.16754	18.21462	15.85008
Mrpl13	ENSRNOG00000004401	52.89999	36.65757	53.1847	47.9014	28.64892	45.91729
Hspb2	ENSRNOG00000010402	54.92161	60.96996	52.62188	50.84864	54.40636	46.96719
Gpx1	ENSRNOG00000048812	185.9823	51.46836	127.0657	170.5898	40.21458	108.9216
Pgm1	ENSRNOG00000009889	43.59324	32.41719	52.08765	36.52035	27.81407	46.96524
Ppme1	ENSRNOG00000017227	25.43354	19.23136	28.49105	23.611	17.17224	25.67448
Klhdc10	ENSRNOG00000010267	21.36029	21.39911	21.96771	20.18834	19.8179	20.07534
Ppie	ENSRNOG00000014762	36.25539	21.62978	32.51955	33.93843	18.75391	30.73297
Sdf2	ENSRNOG00000012121	66.85652	71.67662	78.54911	64.54952	67.94263	75.32056
Polr3a	ENSRNOG00000010008	8.85211	8.425969	7.818209	8.308844	7.575137	7.188801
Emc7	ENSRNOG00000005884	93.56023	77.47666	92.58352	82.60848	64.50075	84.58839
Ufm1	ENSRNOG00000038176	27.62054	23.7105	29.35627	23.6555	20.51731	26.90073
Lonp2	ENSRNOG00000015162	23.01912	24.05624	22.88998	21.35074	22.96354	21.70853
Irak1	ENSRNOG00000060869	31.05072	38.28504	32.51281	29.49799	35.76013	30.35339
Ptgs2	ENSRNOG00000002525	5.212059	3.807267	3.072334	4.815288	3.321721	2.439357
Irf1	ENSRNOG00000008144	56.51633	38.21702	42.32215	50.57229	34.39199	38.03838
Hmgcs2	ENSRNOG00000019120	0.223951	1.211811	0.276791	0.159479	1.163265	0.236388
Tnpo1	ENSRNOG00000014999	36.52891	35.72682	41.24579	34.53903	34.50821	39.62548
Cep85	ENSRNOG00000016249	13.12901	10.36671	13.27782	11.18053	9.14903	11.80381
Ppil2	ENSRNOG00000026900	40.06511	31.00658	35.29648	36.65371	25.64563	29.91706
Msx1	ENSRNOG00000006876	7.948359	7.99879	9.517561	6.103062	5.944932	8.259413
Zcchc4	ENSRNOG00000029064	4.437212	4.454298	4.169458	3.765804	3.352726	3.230876
Gpr108	ENSRNOG00000046128	36.09472	37.92407	34.77364	32.49269	33.07931	28.86925
Vdac2	ENSRNOG00000013505	102.2216	99.17547	103.4013	96.32574	89.53692	95.6646
Narf	ENSRNOG00000036664	10.56031	11.70218	13.69079	9.918164	10.7177	13.00424
Arfip2	ENSRNOG00000018440	21.22987	28.07678	24.62355	18.56231	26.3028	22.8078
Gucy2e	ENSRNOG00000015058	0.113566	0.076615	0.105271	0.077196	0.020429	0.046704
Tiprl	ENSRNOG00000003048	15.87953	13.42734	16.462	13.33891	11.02975	14.89465
Kcnj8	ENSRNOG00000013463	8.51431	10.84762	17.6443	6.221903	7.644763	13.86049
Alg12	ENSRNOG00000004591	18.36963	12.90793	13.39369	16.33854	9.852196	11.32411
Cenpb	ENSRNOG00000057284	87.49191	79.97957	91.92404	83.20809	74.30122	84.87248
Psenen	ENSRNOG00000020941	87.44999	94.2137	77.92212	82.4799	86.66148	69.79651
Gtf2f1	ENSRNOG00000047134	59.28009	45.13245	51.95698	54.90815	38.59691	44.77003
Atp5po	ENSRNOG00000001991	202.4369	166.8752	208.247	183.6342	136.6853	178.6539
Smim3	ENSRNOG00000019536	23.46546	27.04976	49.90776	19.42967	20.70475	43.41906
Mtrr	ENSRNOG00000017826	12.67794	10.97931	13.07609	11.17168	9.754558	11.08947
Mrpl53	ENSRNOG00000053109	70.50895	53.69766	66.7125	64.2859	46.85104	56.998
Sfrp4	ENSRNOG00000054957	1.881138	1.502183	2.562536	1.10953	0.801113	2.094133
Nup205	ENSRNOG00000010852	27.04382	15.18471	24.76682	24.64486	11.23463	21.64304
Ak2	ENSRNOG00000000122	63.6887	36.46394	65.02726	56.72396	26.67788	58.81143
Trappc10	ENSRNOG00000023210	11.45644	13.10183	11.47914	11.24099	12.74748	11.15255
Hax1	ENSRNOG00000045647	36.06871	35.92328	38.4244	32.59759	30.1599	33.63747
Ifrd2	ENSRNOG00000016150	15.10796	6.570863	8.683836	13.57474	4.387418	6.130996
Eif3l	ENSRNOG00000011020	112.358	117.4781	115.4445	103.4628	104.5268	107.0354
RGD1308706	ENSRNOG00000017090	12.84372	10.93111	17.87038	11.64902	9.230124	16.78742
Ide	ENSRNOG00000016833	22.56454	21.76424	21.87084	21.20897	20.55494	19.95923
Lsm1	ENSRNOG00000015375	29.24263	25.43573	23.70049	27.47602	23.6774	22.60165

Noa1	ENSRNOG00000002055	9.879759	9.95169	11.27911	8.967077	8.500563	9.802344
Mark3	ENSRNOG00000010330	21.51136	22.61816	27.26894	20.46356	21.3499	25.56585
Cops3	ENSRNOG00000053943	42.26895	30.28025	39.50486	36.65694	25.97092	36.0969
Pdf	ENSRNOG00000022303	25.03276	31.02754	27.05507	22.59016	29.35752	24.25897
RGD1309104	ENSRNOG00000028236	26.49201	28.53037	27.35993	23.74244	24.29078	24.47885
Epb41l4b	ENSRNOG00000056550	0.010292	0.019093	0.01431	0	0.010182	0
Cks1b	ENSRNOG00000042561	49.99534	22.35255	45.49787	44.60727	15.62409	41.48645
Pex14	ENSRNOG00000013498	21.72444	20.51784	22.33251	18.65292	15.73483	19.06592
Nip7	ENSRNOG00000020391	34.42536	27.43896	32.82686	28.29425	19.58449	28.11801
Ube3d	ENSRNOG00000010802	3.65343	3.821987	4.214411	3.252086	3.433921	3.617593
Trim27	ENSRNOG00000055917	53.06054	42.3941	43.83912	45.43891	36.23839	39.30452
Psmb3	ENSRNOG00000052730	216.6367	157.9227	217.0339	191.9296	118.9811	190.0636
Kcnk5	ENSRNOG00000047005	0.205957	0.140772	0.21101	0.023158	0.032174	0.049036
P4ha1	ENSRNOG00000050655	337.4466	181.6594	487.4718	310.1502	141.5962	461.7577
Lonp1	ENSRNOG00000046502	51.43788	35.44385	44.27672	50.73694	34.27159	43.19256
LOC691083	ENSRNOG00000013437	3.593413	2.52805	4.023335	2.954269	1.777637	3.579879
Angptl4	ENSRNOG00000007545	22.49369	2.472109	7.724541	21.32918	1.672564	6.36981
Usp4	ENSRNOG00000054863	26.02589	22.57717	23.01173	23.77558	21.08769	20.49453
Rpl4	ENSRNOG00000009378	1146.183	1110.195	1125.729	1040.696	931.6848	981.0089
Lta4h	ENSRNOG00000004494	30.83531	26.3949	43.27111	27.09695	23.29503	38.1789
Gemin6	ENSRNOG00000027332	15.77202	7.651277	13.78825	13.38197	4.199931	11.61554
Sdc1	ENSRNOG00000059947	69.96849	49.48102	90.59683	61.04632	34.31616	77.80292
Ccz1b	ENSRNOG00000001032	12.06563	12.27871	14.02677	10.99617	10.5337	12.27759
Bcs1l	ENSRNOG00000016754	12.91108	7.815306	10.2161	11.52923	6.330698	9.332602
Gusb	ENSRNOG00000000913	33.32641	40.80356	33.81368	31.64013	37.93016	31.37706
Snrpd1	ENSRNOG00000013714	55.91754	37.92886	68.81818	48.25621	25.00522	56.91997
C5ar2	ENSRNOG00000049028	3.802072	4.018952	6.474997	2.990864	3.324309	5.999821
Mok	ENSRNOG00000007850	4.43512	2.003737	2.257951	3.878354	1.614255	1.912762
Grina	ENSRNOG00000029941	168.6217	186.256	124.2473	158.4681	170.5408	107.0138
Pja1	ENSRNOG00000047339	19.90818	21.34989	19.52849	17.83683	18.02082	17.21664
Coro1a	ENSRNOG00000019430	1.174145	0.914883	0.957778	0.476593	0.209103	0.531149
Siae	ENSRNOG00000031266	13.18561	11.31501	13.87231	11.92839	9.59903	11.7234
Slc25a44	ENSRNOG00000025269	14.24042	24.85507	13.30589	13.11819	23.26422	11.37908
Nmi	ENSRNOG00000027502	41.36351	20.81584	32.5565	39.10368	19.21245	29.80421
Cct5	ENSRNOG00000011632	279.7239	243.1374	300.5855	255.3963	201.9638	269.1878
Tpi1	ENSRNOG00000015290	115.4818	47.15681	180.0346	104.7446	30.71872	161.6038
Rnd1	ENSRNOG00000059857	34.53868	11.71133	36.0612	28.16857	6.464991	32.36766
Sms	ENSRNOG00000007688	35.74011	27.53772	32.11917	33.49441	25.95265	29.37693
LOC100910506	ENSRNOG00000060946	3.700064	3.621932	4.265716	3.416072	3.366458	3.852271
Birc2	ENSRNOG00000010602	21.34786	15.76704	19.00774	20.44896	14.55655	18.29118
Lrit1	ENSRNOG00000012790	0.092531	0.128749	0.085773	0.029652	0.027465	0.016743
Vav1	ENSRNOG00000050430	0.074149	0.027513	0.027493	0.031682	0	0
Pla1a	ENSRNOG00000057153	45.32843	21.87011	23.02306	39.45914	14.89894	19.01113
Cyp2j10	ENSRNOG00000042224	1.773824	1.940516	1.152771	1.513744	1.783611	0.968146
LOC100302465	ENSRNOG00000027542	0.691922	0.252149	0.62993	0.620311	0.134471	0.54776
Phb2	ENSRNOG00000012999	140.6274	113.5768	132.3011	126.9323	90.05285	114.1693
Psmb7	ENSRNOG00000011732	117.0311	81.51174	121.3303	105.5228	62.03451	106.9848
Adk	ENSRNOG00000012325	26.13091	18.59617	25.24601	23.35363	14.46392	22.67644
Sumo1	ENSRNOG00000016133	113.9933	90.85376	120.201	105.3402	75.8658	106.544
Mcm2	ENSRNOG00000016316	82.80157	34.19005	53.43191	74.92927	25.45062	48.40092
Dcaf13	ENSRNOG00000004301	39.07921	28.45572	37.75938	37.32259	26.67796	35.01622
Jazf1	ENSRNOG00000027026	1.751263	2.150057	2.530515	1.320463	1.885559	2.23686
Alyref	ENSRNOG00000036687	56.64718	31.15214	56.08152	48.91202	19.47972	48.75728
Tmem88	ENSRNOG00000009870	1.437747	1.044705	1.865825	1.075035	0.782368	1.40919

Wdr74	ENSRNOG00000042878	25.18568	18.3623	22.51686	21.48787	13.52582	19.71515
Esd	ENSRNOG00000009512	87.93608	58.98001	76.09937	78.27593	50.72434	62.36151
Mtcp1	ENSRNOG00000024071	7.267577	9.238307	8.857574	6.612963	8.122534	8.036463
Rnps1	ENSRNOG00000008703	110.4835	67.82661	103.5217	95.49565	58.27466	93.80611
Nrn1	ENSRNOG00000050767	2.865306	2.708509	2.908975	2.448518	2.429909	2.41954
Ccdc12	ENSRNOG00000020946	17.3659	13.45253	20.89248	15.14573	10.6471	17.09063
LOC100360413	ENSRNOG00000056855	1476.182	1677.179	1567.915	1349.038	1461.64	1354.777
Ssb	ENSRNOG00000007998	57.35403	41.45729	58.76784	49.20696	30.98904	52.76067
Tnfaip2	ENSRNOG00000010165	52.07632	41.35311	46.48509	49.28861	38.47148	42.06949
LOC687399	ENSRNOG00000045541	0.427266	0.442963	0.419356	0.295314	0.298398	0.204653
Ubtd1	ENSRNOG00000013813	21.99481	26.8277	24.06794	19.21081	23.48463	22.17179
Mcu	ENSRNOG00000045920	16.288	13.24934	15.72369	13.34223	11.29012	13.89935
Nubp1	ENSRNOG00000002574	33.40096	26.71037	41.19854	31.10322	22.83936	38.45647
Ccdc84	ENSRNOG00000012137	8.644949	12.9352	10.20852	7.467752	11.23071	9.181708
Pop1	ENSRNOG00000005243	6.053209	4.474151	7.167506	5.392046	3.311917	6.134563
Cd302	ENSRNOG00000006623	38.57321	57.31269	33.51206	36.72796	55.87943	32.4562
Arpin	ENSRNOG00000014589	21.34724	15.01081	23.47438	19.69791	12.2119	20.66412
Paics	ENSRNOG00000002101	63.9712	61.4152	55.15168	57.4066	52.16974	49.71306
Fam213b	ENSRNOG00000013468	7.792411	8.538936	10.35861	6.082673	6.015264	7.321286
Naif1	ENSRNOG00000050204	3.48547	3.428059	3.18506	2.983755	2.690301	2.739703
Neil1	ENSRNOG00000018577	6.38857	7.840987	6.445652	4.619041	5.955277	5.367155
Psmb8	ENSRNOG00000000456	73.84423	24.1591	40.36613	70.10348	17.87401	36.01132
Wdr54	ENSRNOG00000060514	16.43873	10.71971	17.41954	13.24462	8.792426	15.25417
P4ha2	ENSRNOG00000033663	91.20181	84.91468	123.3542	83.54954	71.39883	112.8406
Lars	ENSRNOG00000018304	49.0563	47.17571	49.58824	44.91341	40.28101	44.903
AABR07026654.1	ENSRNOG00000014745	12.15421	14.43459	17.03625	11.13375	12.75401	15.24506
Calm2	ENSRNOG00000016770	310.4896	244.7874	369.8193	280.8355	195.3414	336.1488
Cd44	ENSRNOG00000006094	103.0924	69.96867	89.17583	96.63324	62.93068	85.1916
Cluap1	ENSRNOG00000007117	21.57038	14.9627	18.30813	19.76095	12.82737	15.24553
Psmd4	ENSRNOG00000021042	120.4836	87.92077	100.6265	105.1863	73.16341	91.82689
Cdc7	ENSRNOG00000002105	8.367139	4.059826	7.445756	7.328806	2.470764	6.463708
Aip	ENSRNOG00000022289	28.61389	25.02811	28.59049	25.7643	23.41235	26.41233
Gnal	ENSRNOG00000010440	0.109409	0.081191	0.101418	0.046747	0.043299	0.059392
Noc4l	ENSRNOG00000037478	7.676619	6.109295	6.600553	6.646774	5.479503	5.920005
Dpy30	ENSRNOG00000027126	47.0915	32.35248	47.05736	43.67657	26.24617	42.08769
Agtrap	ENSRNOG00000008619	18.33518	23.54936	14.9846	17.25582	22.35906	13.20219
Pigq	ENSRNOG00000020140	21.78901	14.96732	18.16849	20.52215	14.04595	17.43882
Cox7a2l	ENSRNOG00000004526	141.197	169.2754	144.1582	129.4207	148.9032	129.4799
Me2	ENSRNOG00000015582	17.80806	11.95772	20.58378	15.96361	9.789687	17.45618
Slc51b	ENSRNOG00000028889	2.956626	0.731358	1.735758	2.000189	0.195016	0.980828
AABR07063279.1	ENSRNOG00000051056	20.28183	17.90603	18.77591	16.66503	15.77593	16.26843
Nat9	ENSRNOG00000003264	17.83299	19.66518	20.07458	15.94	17.8131	17.11523
Urod	ENSRNOG00000018211	56.99624	58.48475	51.95003	51.39347	50.76326	47.51054
RGD1308147	ENSRNOG00000015523	11.8256	11.00481	12.05451	10.84468	9.819047	10.37231
Nme6	ENSRNOG00000020721	17.20811	12.37245	13.34606	15.40385	10.64376	12.31757
Slc4a5	ENSRNOG00000010378	0.060207	0.055849	0.027905	0.010719	0.009928	0
Gch1	ENSRNOG00000011039	2.93907	1.222137	2.442563	2.652286	0.726969	1.94848
Ube2l6	ENSRNOG00000030467	57.24313	22.59436	45.79936	49.70221	17.27016	41.4226
Emc6	ENSRNOG00000019352	25.14625	17.82152	20.51421	19.35314	13.13746	17.2942
Ncbp3	ENSRNOG00000018550	11.87753	12.09407	12.82282	10.54255	10.6163	10.60455
Ergic2	ENSRNOG00000001852	39.79381	33.37542	43.5334	34.14973	28.95721	40.38033
Dok1	ENSRNOG00000007412	48.24022	24.69206	39.6609	43.59074	21.80018	36.68539
Rhod	ENSRNOG00000019220	13.99881	16.45444	12.32048	12.389	14.97525	9.876206

Pgs1	ENSRNOG00000002949	26.55712	30.91282	36.85668	22.94991	25.71586	33.82187
LOC100911515	ENSRNOG00000050669	391.2944	163.2993	613.1073	344.1012	100.1015	529.2179
Zfp276	ENSRNOG00000016631	7.838792	8.453439	8.117859	6.078089	7.433054	6.341321
Elac2	ENSRNOG00000003424	16.36263	13.35245	16.52684	14.90864	10.97082	13.91946
Nup54	ENSRNOG00000002247	21.94472	15.9046	19.98834	19.80841	12.43594	16.14512
Gon7	ENSRNOG00000046085	4.386987	3.145759	4.5615	4.038652	2.741321	3.969095
Psmc2	ENSRNOG00000012026	161.6693	104.697	131.7433	145.6468	80.03717	116.7453
Nup43	ENSRNOG00000049505	19.12074	8.613285	14.57193	16.98762	6.688721	11.34867
Fam72a	ENSRNOG00000042747	7.053724	3.394538	6.931807	5.268562	2.151377	5.901971
Psmb9	ENSRNOG00000000459	97.18312	25.20658	45.01949	86.94693	18.62419	38.77761
Bad	ENSRNOG00000021147	41.22529	43.32117	45.27282	35.46105	32.80833	36.21225
LOC108349606	ENSRNOG00000031244	462.5754	468.6379	472.5734	429.011	408.5692	415.6707
Plekhj1	ENSRNOG00000019247	24.21351	22.41844	23.03559	20.03022	19.49844	20.63046
Kif20a	ENSRNOG00000024428	78.5177	31.71867	173.842	70.01577	16.16516	160.976
Amd1	ENSRNOG00000000585	59.34423	41.18179	53.2968	49.40879	34.83016	47.22902
LOC100294508	ENSRNOG00000012204	25.11193	20.16913	20.85619	23.54493	18.29248	19.83111
Mgarp	ENSRNOG00000011923	0.965194	0.346576	2.770668	0.565385	0.06161	2.253553
Dcaf11	ENSRNOG00000018825	32.23757	35.00166	35.08683	27.91905	32.48077	32.16427
Psma7	ENSRNOG00000056853	208.0679	171.2457	230.9613	189.0607	140.3423	196.2804
Ncaph2	ENSRNOG00000009598	58.09026	31.70604	49.03378	53.83566	25.60709	45.53926
Cdk5	ENSRNOG00000008017	19.85704	24.26312	23.22628	17.68514	20.64703	19.28202
Fkbp14	ENSRNOG00000009886	31.70568	43.1561	38.82366	26.52036	35.35255	34.22528
Cldn25	ENSRNOG00000057454	0.164689	0.152767	0.254434	0	0	0
Tas1r2	ENSRNOG00000061876	7.129142	7.044038	6.450969	6.601238	6.756903	6.02516
Zfp28	ENSRNOG00000042777	2.653805	4.651376	3.207473	2.349222	4.134293	2.865295
Tmed10	ENSRNOG00000007901	224.6817	208.9855	252.5503	205.8474	174.9776	227.3187
Cdc34	ENSRNOG00000060530	39.6651	27.97784	32.18024	35.93342	21.11233	26.15252
Ube2k	ENSRNOG00000027088	20.18333	13.89453	26.78933	18.54973	13.00383	25.51854
Ccdc14	ENSRNOG00000021469	6.032511	2.145178	3.885412	5.720578	1.640768	3.572049
Cuedc2	ENSRNOG00000019574	33.60053	25.91586	41.78164	30.10832	19.75469	35.56758
Zc3hc1	ENSRNOG00000010154	29.19117	21.08187	24.99811	25.37601	17.67913	22.92759
Arfgap3	ENSRNOG00000046472	49.14872	65.79327	53.01344	43.14278	55.59281	46.31974
Nans	ENSRNOG00000008945	110.7223	73.25037	101.4794	96.47325	52.35188	89.52569
Tmem68	ENSRNOG00000008872	9.175861	11.02195	10.25643	7.829095	9.594451	9.475012
Nap1l5	ENSRNOG00000007808	0.140022	0.259771	0.346118	0.074784	0.207803	0.253367
Trmt6	ENSRNOG00000021270	10.4339	8.734614	10.71062	9.576639	7.583057	9.148635
Ccdc148	ENSRNOG00000057740	0.042017	0.038975	0.090879	0	0	0.025343
Dguok	ENSRNOG00000011617	22.95703	22.70332	19.90175	20.31927	21.24206	17.32252
Rpl35al1	ENSRNOG00000032348	52.79638	38.84175	49.6784	46.30773	35.34937	43.85518
Ddx18	ENSRNOG00000025430	26.25643	22.81522	24.35484	22.91146	20.96208	21.96813
Srbd1	ENSRNOG00000014720	15.16704	12.30407	13.34556	14.52103	11.25182	12.69733
Ces2g	ENSRNOG00000013808	0.098788	0.018327	0.009157	0.084418	0.009774	0
Farsa	ENSRNOG00000003149	53.21382	42.7956	50.76356	47.13317	34.64351	46.32589
Borcs7	ENSRNOG00000020076	10.28048	9.487526	10.74716	9.391206	8.975753	9.808239
Ndufs2	ENSRNOG00000038372	120.8956	118.993	126.4038	111.7413	102.2509	114.1149
Ugdh	ENSRNOG00000002643	151.4596	101.669	200.2838	140.8227	81.73454	183.3245
AABR07029651.1	ENSRNOG00000002423	39.45164	30.09391	39.4938	34.54727	27.36761	36.03331
Slc12a2	ENSRNOG00000015971	12.03731	17.76099	13.65399	10.85282	16.36113	12.90746
Prdm5	ENSRNOG00000023679	10.23979	11.31121	11.03158	9.748076	10.49845	10.11302
Ciapin1	ENSRNOG00000016234	38.46201	31.08626	35.03217	31.52819	24.04453	31.14588
Eif4a3	ENSRNOG00000045791	128.2929	96.24943	111.4737	110.9416	79.1308	101.8943
Atg4c	ENSRNOG00000008767	8.152117	10.54094	9.922912	6.538245	9.151868	9.064252

Chmp7	ENSRNOG00000016939	16.3334	17.63623	16.59014	14.96281	16.44261	15.86031
Mtg2	ENSRNOG00000059919	17.49963	18.23568	19.23318	15.81518	15.44212	16.07453
Hnrnpk	ENSRNOG00000019113	254.1842	225.3213	293.2096	235.7554	191.5518	260.6216
LOC100361259	ENSRNOG00000055911	34.82046	38.68268	43.32945	30.92136	31.63377	38.37316
Ube3c	ENSRNOG00000010702	31.47533	29.10411	32.47771	29.28319	25.5203	30.28515
Lgals8	ENSRNOG00000018046	45.25115	47.83854	51.08946	39.8685	41.53263	47.74598
Trappc5	ENSRNOG00000001003	27.52146	29.20929	31.33166	23.49149	21.60422	25.04116
Rfc3	ENSRNOG00000001088	45.35983	15.29555	32.47357	42.48841	11.79203	27.3506
Tgm1	ENSRNOG00000020136	0.822812	0.237226	1.041001	0.641365	0.132013	0.845033
Rbm3	ENSRNOG00000005387	226.8183	245.1913	277.498	190.6719	187.4639	243.0213
Atp5mf	ENSRNOG00000027049	298.4216	256.9507	410.3681	266.8832	198.1661	355.7769
Taldo1	ENSRNOG00000018367	161.3976	124.5691	151.2128	149.4443	107.3674	128.8037
Mcfd2	ENSRNOG00000015059	123.069	119.1122	124.5073	106.8803	98.31462	113.449
Serpind1	ENSRNOG00000001865	0.08131	0.181018	0.180891	0.017371	0.128716	0.14713
Arf1	ENSRNOG00000060229	343.4679	301.9456	396.3753	324.8968	266.7637	367.4843
Esam	ENSRNOG00000033217	0.297272	0.275752	0.258336	0.119077	0.073529	0.151287
Nedd8	ENSRNOG00000019895	186.9357	133.1796	208.8391	170.6162	102.9185	186.546
Epn2	ENSRNOG00000060550	41.46258	38.82866	51.75978	37.85491	36.58208	47.4985
Zfand4	ENSRNOG00000011791	2.344174	2.292369	3.992474	2.126881	1.886172	3.615707
Epor	ENSRNOG00000012619	0.488479	0.394015	0.748104	0.29492	0.273166	0.518808
Mrpl32	ENSRNOG00000015989	28.553	23.26596	28.73351	24.75481	18.52693	26.23123
LOC108348064	ENSRNOG00000027295	22.55382	24.78652	23.57088	19.9002	20.02732	20.32285
Timm8a1	ENSRNOG00000011226	43.83386	29.8129	43.50815	35.35035	24.63589	33.68442
Gnb4	ENSRNOG00000011070	9.158328	9.369866	9.912618	8.430383	8.981173	9.236376
LOC100912489	ENSRNOG00000051891	941.3764	399.5508	847.9627	844.2185	249.3377	761.4373
Ddx51	ENSRNOG00000037480	9.604633	7.027627	8.110009	9.00648	5.898222	7.241435
Msn	ENSRNOG00000030118	176.6133	125.0437	215.1874	165.1379	104.4943	193.9347
Gtf2e1	ENSRNOG00000026008	12.22075	7.803383	9.44213	11.14527	5.755183	7.788027
Akr1b8	ENSRNOG00000009734	24.63783	9.58132	15.24897	21.95289	8.175538	12.93202
Vps52	ENSRNOG00000000470	18.87959	17.41185	18.92113	17.64067	15.30048	16.5674
Ttc8	ENSRNOG00000004542	9.371349	8.885136	9.144837	8.069479	7.805412	8.464241
Dnal4	ENSRNOG00000015583	20.89163	20.31999	20.7758	18.06397	16.10445	18.41983
Ptma	ENSRNOG00000025731	134.5593	100.5368	189.7092	120.9555	89.67555	169.9503
Acy1	ENSRNOG00000011189	22.84875	19.97915	20.80441	19.46918	17.30238	19.03398
Aktip	ENSRNOG00000011956	15.46919	22.1577	17.70966	15.15859	21.64939	17.40483
Rhoj	ENSRNOG00000021919	19.47564	18.97622	22.62814	17.79296	18.00653	21.55301
Tmco4	ENSRNOG00000017401	5.598559	4.895045	4.64498	4.417088	4.266781	3.771359
LOC100359583	ENSRNOG00000018584	872.1789	748.356	1219.095	776.5444	564.7914	1063.357
Ripk3	ENSRNOG00000020465	84.15735	47.30532	74.18553	67.55631	33.12379	65.53501
Prim1	ENSRNOG00000031993	54.11218	21.3874	37.99058	44.75829	13.64051	33.12088
Fbxo18	ENSRNOG00000018549	48.71099	51.73014	53.96179	45.27971	47.41481	51.70899
Exosc2	ENSRNOG00000009245	18.16761	8.496594	11.64906	15.30981	4.940676	9.797307
LOC108348072	ENSRNOG00000047854	1.753015	1.405282	4.152709	1.410174	0.749436	3.563667
LOC108351137	ENSRNOG00000030963	696.5584	423.2427	934.1855	635.3336	322.5361	816.2801
Shroom1	ENSRNOG00000007431	9.994752	12.64661	11.26191	8.643201	11.25058	10.5155
Hnrnpr	ENSRNOG00000011910	36.97849	26.9172	37.98037	34.34871	24.54594	36.60543
Atp5if1	ENSRNOG00000013300	214.1827	145.7239	213.7538	203.4933	127.4762	193.2545
Rnh1	ENSRNOG00000016416	303.1597	256.8896	302.499	270.6558	205.7069	273.0482
Tmem120a	ENSRNOG00000001441	47.69104	43.99223	42.41695	45.34516	39.51582	38.99716
Rabepk	ENSRNOG00000018591	16.32779	12.40007	14.47137	12.59623	10.46263	11.23052

Olr1158	ENSRNOG00000026232	0.318889	0.221853	0.258647	0.042579	0.078876	0.036064
Eif2b4	ENSRNOG00000005301	18.51082	16.28112	17.55885	17.41645	14.16278	15.8147
Ndel1	ENSRNOG00000004139	49.3237	56.19212	51.0759	45.09951	53.65678	46.16751
Abat	ENSRNOG00000002636	0.014234	0.026407	0.026389	0	0	0
Slc8b1	ENSRNOG00000001383	33.58993	40.51626	22.46425	31.25948	39.18588	20.98253
Porcn	ENSRNOG00000004819	3.967306	5.594507	3.974704	2.86799	4.361331	3.335596
Sfi1	ENSRNOG00000018412	7.426088	5.913854	6.100112	7.291021	5.823102	5.925321
Rgs16	ENSRNOG00000027024	0.091509	0.042442	0.028275	0.065165	0.01509	0.013799
Vps4b	ENSRNOG00000002705	41.78041	37.34881	38.37105	38.30401	32.4245	35.74196
Pak1	ENSRNOG00000029784	49.47848	31.10123	43.76997	47.94156	29.73145	41.35804
Wdr13	ENSRNOG00000039587	23.42789	25.41529	28.2271	18.87485	22.90913	25.19296
Mroh7	ENSRNOG00000058870	0.054131	0.050213	0.016726	0.028911	0.017852	0
Cbx8	ENSRNOG00000048113	7.465589	10.43902	9.816567	6.172896	8.535339	8.780769
Rpl12	ENSRNOG00000016220	581.2428	686.276	649.6227	523.2657	618.1202	545.4702
LOC100362830	ENSRNOG00000008551	546.1341	593.7868	554.5249	517.5917	539.7765	502.3744
Tmem35b	ENSRNOG00000028083	18.92011	11.57655	12.20926	16.44005	8.063698	10.33654
Smarcd2	ENSRNOG00000010557	46.99841	38.92947	44.78214	45.24925	35.59245	42.32357
Timm10	ENSRNOG00000007883	13.08531	9.994098	9.822295	10.3691	7.071281	8.300055
Slc39a11	ENSRNOG00000031213	11.41475	7.685848	10.72223	10.55304	6.203363	9.807864
Sppl2a	ENSRNOG00000011652	26.32487	32.90794	28.72575	23.18272	31.23629	26.51498
Ppp1r35	ENSRNOG00000024505	16.65744	12.00555	16.32944	15.19024	9.169086	13.73349
Ncstn	ENSRNOG00000005355	68.76439	75.91045	58.59238	61.79262	67.16131	54.11146
Nat1	ENSRNOG00000014055	10.0366	8.724938	9.217537	9.333678	7.378728	7.939183
Ubl5	ENSRNOG00000020442	168.444	148.2759	165.7632	154.6321	124.29	150.931
Gna13	ENSRNOG00000036745	16.82889	18.28857	15.12538	15.97884	17.77529	14.6257
Psmc1	ENSRNOG00000003951	116.4163	86.21407	109.3365	110.6851	75.33701	98.67548
Mrps9	ENSRNOG00000016201	18.31927	19.57686	22.04579	15.96352	18.25732	19.48199
Psmd12	ENSRNOG00000003117	51.134	36.60078	50.03438	49.44338	33.28075	47.1435
Dhfr	ENSRNOG00000013521	7.164826	3.043984	7.02824	5.690686	2.286191	5.676571
Znhit3	ENSRNOG00000027818	20.25678	13.51989	14.65538	17.85557	9.450593	12.21811
RGD1304567	ENSRNOG00000025034	36.79953	26.32863	28.83698	30.90072	21.64309	25.83376
Zbed4	ENSRNOG00000004588	3.120211	4.001962	3.610444	2.695195	3.382442	3.279554
Slc46a3	ENSRNOG00000000937	1.946762	1.64692	1.443655	1.64695	1.494653	1.183606
Dnajb4	ENSRNOG00000013011	22.99788	20.34821	21.37598	21.49712	17.85701	19.91724
Gsr	ENSRNOG00000014915	56.54082	44.04211	52.39353	50.61525	37.43078	49.01989
Bnip3	ENSRNOG00000017243	55.83637	36.20865	94.99728	46.85174	27.50706	80.1016
Nae1	ENSRNOG00000033133	15.66131	15.25199	18.43043	13.43732	13.86622	17.16757
Alad	ENSRNOG00000015206	12.1905	11.56712	13.16804	10.74121	8.804023	11.20978
Clptm1	ENSRNOG00000018255	88.74003	74.77736	77.52877	84.29755	66.04888	70.72268
RGD1309534	ENSRNOG00000010887	10.0473	10.78933	10.32029	9.668716	10.5974	10.01835
Rrp9	ENSRNOG00000012927	27.41347	14.45275	18.35372	24.4985	12.31357	16.84754
P2rx2	ENSRNOG00000037456	0.288285	0.250702	0.200421	0.211708	0.124786	0.048904
Mrps10	ENSRNOG00000022609	6.375119	4.159338	5.937749	5.636772	3.349892	4.636352
Mthfsd	ENSRNOG00000046443	8.641447	6.266384	7.628827	6.959562	5.394472	6.01884
Dse	ENSRNOG00000000824	35.86337	32.98142	54.34355	33.87515	29.10752	50.7417
Tp53i3	ENSRNOG00000005177	20.10971	19.72802	16.84469	17.29056	15.85155	14.8375
Rpia	ENSRNOG00000005576	11.87399	11.89864	12.67787	11.00121	11.41992	12.11154
Kars	ENSRNOG00000019456	141.4141	76.0849	115.1118	127.424	68.54305	105.7868
Plod1	ENSRNOG00000007763	76.38431	85.37059	84.63804	72.62881	79.02535	77.19515
Gstp1	ENSRNOG00000018237	498.4417	435.2732	327.2447	457.577	382.3759	300.4054
Kntc1	ENSRNOG00000033658	22.3649	7.053274	17.13196	20.59621	4.289035	15.60451
Aig1	ENSRNOG00000010753	34.95773	34.78186	43.75327	32.28497	29.51119	38.97247
LOC303566	ENSRNOG00000020908	2.499614	0.687955	1.845993	2.244581	0.298944	1.341994
Ppa2	ENSRNOG00000012091	5.114981	6.27525	6.729698	4.607632	5.503794	6.312496
Serbp1	ENSRNOG00000005890	199.7455	145.5385	204.9228	178.7016	134.9492	186.5763

Stx6	ENSRNOG00000003402	26.96989	24.38462	24.38242	24.2472	22.98287	22.41098
Mcm4	ENSRNOG00000001833	76.85261	26.21446	50.76603	66.88446	16.29998	45.55079
Mmp23	ENSRNOG00000017477	30.15219	35.97138	24.4748	27.79431	31.26793	20.53292
Fam109b	ENSRNOG00000042522	4.588405	6.312778	5.566962	4.211751	5.608687	4.836866
Rhoc	ENSRNOG00000012630	212.8535	154.8296	219.4333	185.5254	113.0767	196.8522
Defb29	ENSRNOG00000023195	0.537953	0.399208	0.897589	0.114926	0	0.681393
Tgds	ENSRNOG00000009661	13.99419	13.96455	15.65221	12.37451	11.99576	14.66669
Hdgf	ENSRNOG00000042261	145.3852	107.1896	192.7454	133.2959	98.38632	175.7336
Rps12	ENSRNOG00000016411	192.448	178.1316	142.0465	166.3455	159.9597	128.4441
LOC100363782	ENSRNOG00000050510	80.91262	69.51097	80.88229	71.1192	53.26194	71.54971
Cacul1	ENSRNOG00000009954	34.59184	33.03881	34.69647	31.28944	29.71835	32.96653
Gtf2e2	ENSRNOG00000014422	23.45262	19.30165	25.1162	21.69354	16.09448	23.06957
Ankrd24	ENSRNOG00000006759	4.225092	4.049873	3.75581	3.691514	3.78366	3.312938
Trmt112	ENSRNOG00000021132	57.57098	52.06369	62.06757	48.87415	43.45062	57.55101
Npm1	ENSRNOG00000004616	332.5079	374.7094	374.8595	303.9331	318.0398	331.903
Actr10	ENSRNOG00000007504	73.85284	62.70548	74.30468	63.99899	56.76152	68.66388
Cd320	ENSRNOG00000006901	23.92894	29.46204	22.23453	21.65397	28.17634	20.84191
Crat	ENSRNOG00000018145	31.63283	39.0295	42.20782	27.77756	32.20278	38.03673
Jtb	ENSRNOG00000016379	23.5143	28.28485	26.19032	18.50754	25.7918	22.15586
Trmt44	ENSRNOG00000008898	2.837094	3.33383	2.816316	2.142844	2.770323	2.471062
Anapc10	ENSRNOG00000018296	17.80138	14.25585	16.89582	16.56773	11.79512	14.65652
Elob	ENSRNOG00000004814	405.6455	252.8642	424.3559	374.6009	199.7555	393.2483
Taf8	ENSRNOG00000015249	5.224299	4.101696	4.5154	4.337504	3.660276	3.731945
Commd8	ENSRNOG00000002320	31.60748	38.22844	36.9945	25.03918	28.58772	31.95951
Fnta	ENSRNOG00000014462	66.23562	58.16887	65.5778	58.20821	49.23229	61.11786
Cstf1	ENSRNOG00000004775	18.24941	14.79151	16.27476	15.6433	12.10406	14.90203
Ofd1	ENSRNOG00000004574	7.431602	6.962261	8.525246	6.78647	5.867537	7.89053
LOC108351137	ENSRNOG00000018630	704.4775	408.0988	902.6074	652.993	304.4348	811.4705
LOC498453	ENSRNOG00000022323	31.43427	25.33292	33.59612	28.93363	22.15172	28.84333
Bzw1	ENSRNOG00000013977	143.4348	121.7024	148.0208	129.2879	107.4796	140.6878
Tsc22d1	ENSRNOG00000001030	30.1005	34.19247	46.85057	27.87212	29.75075	43.53438
Eif3g	ENSRNOG00000020619	148.1935	129.7814	141.1773	138.9311	111.3846	123.9099
Thap1	ENSRNOG00000056956	9.64253	11.15431	10.93619	9.330505	10.55032	10.33196
Ptrhd1	ENSRNOG00000004059	7.255676	5.057327	8.70163	6.173992	3.366267	7.788414
Dazap1	ENSRNOG00000031387	44.25871	31.50399	58.89291	40.73454	27.42241	52.42642
Nudt22	ENSRNOG00000021158	20.10809	18.94541	18.2165	16.79332	15.17273	16.35116
Romo1	ENSRNOG00000045555	97.86943	91.04319	101.5765	89.25258	73.65752	86.02397
Clip2	ENSRNOG00000021611	23.87389	28.0765	26.18269	23.58887	27.52526	25.80492
Clns1a	ENSRNOG00000012788	52.77669	44.83426	57.83423	44.52244	35.14525	53.06265
RGD1561916	ENSRNOG00000013320	0.129035	0.059847	0.179415	0.034458	0	0.058372
Cystm1	ENSRNOG00000018775	56.09968	83.46119	104.4196	43.07655	58.96218	88.45776
Plpp2	ENSRNOG00000000177	21.66656	29.50892	26.43999	20.47804	28.61289	25.8485
Fam192a	ENSRNOG00000017841	38.62323	20.76028	24.28126	34.51299	18.71627	21.18064
Wdr24	ENSRNOG00000019713	11.23183	10.78766	9.305469	10.00529	8.792401	8.201498
Eif2d	ENSRNOG00000004910	26.18927	22.90916	27.00834	24.06942	18.95364	24.47087
Rtca	ENSRNOG00000014575	48.88428	40.36964	52.10854	45.50187	34.509	45.2159
Mcm10	ENSRNOG00000017981	13.22373	3.598435	7.005477	11.56426	1.952193	6.157188
Exosc7	ENSRNOG00000060337	21.40518	14.47388	17.53688	19.6696	11.05248	15.12779
Cyb561d2	ENSRNOG00000021887	19.83391	19.98456	21.69788	17.53237	15.78459	19.09933
Spata7	ENSRNOG00000003955	5.516966	6.057551	5.931546	5.031171	5.421278	4.991435
Rpl5	ENSRNOG00000023529	889.4476	1050.597	1044.847	813.8124	905.5504	894.6256
Ypel5	ENSRNOG00000026742	28.57502	28.61641	27.45976	26.19522	23.82351	23.89156
Prkcd	ENSRNOG00000016346	27.9011	28.72023	30.01045	26.87131	26.79026	27.93776

LOC102553386	ENSRNOG00000053557	104.6566	82.75941	113.9482	93.60161	63.78545	103.0049
Fam71e1	ENSRNOG00000031739	2.539556	4.935782	2.522212	2.260575	4.367141	2.078821
Fahd1	ENSRNOG00000014727	6.210352	10.19028	8.465798	5.853302	9.500642	8.003208
Inhbe	ENSRNOG00000007601	0.469019	0.166349	0.332465	0.368379	0.054593	0.149768
AABR07042936.2	ENSRNOG00000057497	81.43188	64.34627	86.80663	77.47974	56.84491	78.85944
Ptpa	ENSRNOG00000018457	102.3065	93.79194	146.5112	96.72975	83.6836	135.0992
Mmp16	ENSRNOG00000005708	20.96956	28.12962	13.51173	19.41715	27.16717	11.53941
Slx1b	ENSRNOG00000019369	5.957847	8.346684	6.727215	5.211689	6.852789	5.656508
Arpc3	ENSRNOG00000008673	131.2555	124.4954	157.4011	117.1256	96.29504	137.3581
Gps2	ENSRNOG00000016360	14.99195	13.26402	12.89511	13.64596	10.50087	10.45495
Cnn2	ENSRNOG00000043044	388.5791	449.4296	434.1428	355.5449	382.3481	384.2678
Rchy1	ENSRNOG00000002546	20.84067	20.66523	20.58831	19.50279	18.90859	19.72914
Bmp4	ENSRNOG00000009694	116.4041	132.4188	120.4606	101.2294	121.5181	112.9159
Tubgcp2	ENSRNOG00000018137	39.14359	23.59037	33.5256	35.837	17.48419	29.75283
Mrps18b	ENSRNOG00000000804	17.0401	12.7143	12.64266	15.14709	9.777357	11.09956
Galk1	ENSRNOG00000006359	43.41975	31.22681	47.68809	35.35292	21.38124	42.92726
Got2	ENSRNOG00000011782	113.9532	92.24727	126.9744	103.9978	72.25305	107.6366
Hsf2	ENSRNOG00000000808	14.09015	10.56561	12.14467	13.25411	9.225613	11.42645
Glrx3	ENSRNOG00000016227	138.2391	93.6559	125.6959	125.6711	80.0941	119.0523
Ptpn4	ENSRNOG00000002625	3.136792	3.601721	3.013053	2.819397	3.440913	2.796208
Snapc5	ENSRNOG00000010156	22.62375	16.87623	17.67997	21.61526	14.89133	15.66422
Fhl2	ENSRNOG00000016866	69.36506	100.5533	76.20693	62.8665	88.5522	68.84117
Pcbp4	ENSRNOG00000012406	41.72746	43.909	40.29776	37.64935	40.77013	38.32387
Kctd18	ENSRNOG00000027091	5.719056	6.73182	6.310642	5.595168	6.489851	6.149831
LOC102555453	ENSRNOG00000028993	166.2568	192.4793	179.4143	135.7104	145.0217	154.6491
Ascc1	ENSRNOG00000056647	30.82317	26.5747	30.86881	27.98181	23.16597	29.23678
Khdrbs3	ENSRNOG00000009539	19.47904	12.21883	17.3929	17.43872	11.19607	15.33712
Nubpl	ENSRNOG00000027444	3.973455	3.298801	4.088385	3.69258	3.164681	3.846601
Gchfr	ENSRNOG00000012290	16.42403	18.76785	16.54827	14.42909	16.01421	12.54445
Rogdi	ENSRNOG00000003125	14.07172	12.34686	10.47155	12.85378	10.9338	9.798426
Prmt3	ENSRNOG00000014829	15.73097	13.47557	16.09088	13.96876	10.82714	14.75228
Lrrc45	ENSRNOG00000045926	17.59964	16.60596	18.2845	15.67492	15.23262	15.50853
Zfp207	ENSRNOG00000000236	53.46998	45.14087	62.87052	50.49157	40.31372	56.627
Rpl7	ENSRNOG00000006992	172.8456	195.4413	207.7782	159.0346	166.5539	185.6969
Dynll1	ENSRNOG00000011222	282.5976	248.9449	344.1682	247.8518	182.5052	302.4683
G6pd	ENSRNOG00000056728	75.49272	87.82367	93.05626	70.86495	78.7031	83.54621
Slc46a1	ENSRNOG00000010291	10.95664	15.42097	8.690176	8.782106	12.32376	7.170112
AABR07070043.1	ENSRNOG00000050630	466.1798	274.2971	591.4759	423.6667	232.0894	516.9239
Bcam	ENSRNOG00000029399	0.263014	0.243974	0.344193	0.165262	0.091843	0.265956
Pbdc1	ENSRNOG00000002662	22.40502	25.20912	23.90945	19.25432	18.58671	18.74838
Pdcd2l	ENSRNOG00000021119	8.250465	10.05272	7.824526	8.114885	9.806252	7.538455
U2af1l4	ENSRNOG00000024497	23.5308	16.60274	22.62424	21.89286	14.42682	19.36422
Arfip1	ENSRNOG00000010533	40.37435	46.91208	45.76482	35.46462	40.38196	42.63762
Vhl	ENSRNOG00000010258	34.16751	23.12811	29.75493	30.52289	21.33554	26.1588
Commd2	ENSRNOG00000043386	9.836552	9.592393	9.834084	8.301328	8.765173	8.913812
Grn	ENSRNOG00000021031	329.4676	490.5118	224.2524	313.0396	458.8697	204.3431
Trpv2	ENSRNOG00000003104	1.825119	3.199266	1.642052	1.304476	2.788287	1.396289
Atg12	ENSRNOG00000000157	36.58335	25.13907	29.57062	32.106	23.00558	25.44614
Txnl4a	ENSRNOG00000052004	24.14987	17.19867	24.50418	22.85606	14.79858	21.75544
Pex13	ENSRNOG00000054896	11.03263	10.38386	11.41153	9.540808	9.127771	10.71271
Eef1d	ENSRNOG00000021638	146.0824	130.3785	166.0773	127.5655	96.12743	145.8266
Clca4l	ENSRNOG00000036877	0.117138	0.205243	0.229228	0.013903	0.051509	0.153082

Iqcc	ENSRNOG00000048553	3.597345	3.640286	4.133791	3.175697	3.08853	3.873329
Nudt21	ENSRNOG00000042983	34.08046	32.42335	45.68918	32.80237	30.02288	42.97016
Tesk2	ENSRNOG00000017282	1.71242	1.89924	2.381015	1.431519	1.767907	2.14432
Dcdc2	ENSRNOG00000017511	0.016595	0.005131	0.010255	0.005909	0	0
Gpr85	ENSRNOG00000024636	0.027671	0.025668	0.02565	0	0	0.012517
Rgs17	ENSRNOG00000018690	5.179193	7.360021	3.670455	4.068802	6.271213	3.132972
AABR07051445.1	ENSRNOG00000048676	0.124335	0.076889	0.038418	0.04427	0	0
AABR07044711.1	ENSRNOG00000047276	345.0937	202.7045	469.6195	315.0514	139.4133	410.6654
Mbnl1	ENSRNOG00000014076	155.8462	201.6359	168.2635	145.9871	196.5233	162.1529
Dhps	ENSRNOG00000004219	32.67426	24.53469	30.42674	28.43951	20.5293	28.40828
Bak1	ENSRNOG00000000485	15.12345	12.68767	13.89856	14.86767	12.17572	13.36423
Dnase1l1	ENSRNOG00000055641	30.80534	30.23184	35.48174	26.05332	27.68859	30.03935
Gng2	ENSRNOG00000048980	1.508964	0.659292	1.317661	1.226398	0.486831	0.949721
Ost4	ENSRNOG00000008605	249.5076	204.5416	281.2127	231.2495	196.0655	266.0384
Ppm1m	ENSRNOG00000046535	20.33654	17.30069	19.02317	19.2616	15.23971	16.72031
Med28	ENSRNOG00000003592	25.30838	23.04276	26.49228	23.04015	18.85095	24.05221
Pip4k2c	ENSRNOG00000005138	19.78256	25.43696	19.01518	19.07856	23.95835	17.96213
Lsm7	ENSRNOG00000019552	67.09602	45.67989	69.32774	58.76634	35.32353	52.90771
Rpp21	ENSRNOG00000000786	21.33977	19.01546	28.55447	18.49552	14.48392	26.24952
Dut	ENSRNOG00000007221	105.0492	53.96182	121.8228	91.2116	36.63352	113.8109
Eif4h	ENSRNOG00000001454	191.9691	145.771	207.0761	182.8883	127.585	187.8283
Ccdc32	ENSRNOG00000010472	17.75715	17.4302	15.81177	14.62062	15.94865	13.54866
Nt5c3b	ENSRNOG00000016475	18.84151	13.11592	16.42607	17.42706	10.13198	14.32166
Tube1	ENSRNOG00000000598	5.432304	2.544107	5.035524	5.024247	1.822136	4.639128
Cxcl1	ENSRNOG00000002802	560.6826	336.1597	402.9438	469.224	237.467	357.0028
Cebpg	ENSRNOG00000021144	26.10968	30.24388	28.35861	24.14525	27.98527	27.32102
Il33	ENSRNOG00000016456	0.153512	0.01424	0.11384	0.131183	0	0.083334
Tubb4b	ENSRNOG00000010170	456.0386	226.8133	573.4094	395.9521	129.3617	523.8791
Ccdc169	ENSRNOG00000038095	0.033068	0.015337	0.030653	0	0	0
Chmp4c	ENSRNOG00000010238	22.35012	24.51956	19.9957	20.7478	23.74743	18.91859
Tmem109	ENSRNOG00000028017	81.8333	55.85847	88.58168	75.1249	42.16299	74.25378
Krtcap2	ENSRNOG00000020542	48.10804	43.31055	57.70696	42.4266	34.46019	53.3402
Pfdn5	ENSRNOG00000012985	151.6565	159.8539	148.5908	143.7189	142.4203	134.3571
Copg2	ENSRNOG00000011014	22.76228	21.50106	26.28139	21.19809	18.80989	22.85623
LOC100365043	ENSRNOG00000059382	1036.297	146.6372	301.3825	983.9878	66.11053	261.9703
Csf3r	ENSRNOG00000008759	0.01833	0.008502	0.025487	0	0	0.008292
Coasy	ENSRNOG00000019918	15.41569	15.41556	15.02736	14.35149	14.59292	13.36122
Crcp	ENSRNOG00000000901	17.40142	13.16586	11.82746	16.1215	12.52781	10.44456
Set	ENSRNOG00000034241	42.54195	28.17094	37.2927	37.77303	25.18893	34.90562
Phospho2	ENSRNOG00000007979	8.262954	11.35647	10.15742	6.691197	10.20582	9.429579
Qpct	ENSRNOG00000005705	2.165351	1.848823	2.782697	1.73473	0.900768	1.981348
AABR07066818.1	ENSRNOG00000022934	446.2712	425.9829	519.3588	398.0314	351.3566	482.81
2-Sep	ENSRNOG00000017952	159.0338	153.7538	196.3716	150.079	135.3884	177.1543
Ap2s1	ENSRNOG00000015865	126.2194	110.9019	137.5105	112.9467	88.13605	125.5078
Pdxk	ENSRNOG00000049937	19.67705	12.48863	18.84325	18.80606	11.32962	18.31132
Gabarapl1	ENSRNOG00000053362	69.66413	123.4163	76.81795	64.94527	116.1893	66.69536
Exoc3	ENSRNOG00000039776	23.87587	25.24201	23.49094	22.6752	23.99248	21.27669
Psma3	ENSRNOG00000007851	54.3033	43.84448	65.85758	50.9731	36.60781	60.48008
Prdx5	ENSRNOG00000021125	412.2583	286.1599	470.6132	378.6275	212.5233	404.6286
Nipa2	ENSRNOG00000012690	29.10715	28.89992	30.1332	26.92636	24.81037	27.78366
Nr1h2	ENSRNOG00000019812	43.2672	51.33068	41.47087	39.51219	48.15205	39.76918
Abcf2	ENSRNOG00000010609	54.88741	37.44799	46.23038	47.49252	29.29671	42.50021

Ndufv3	ENSRNOG00000001182	38.55879	37.15	34.49456	34.48557	32.29867	32.29763
Mtfr1l	ENSRNOG00000016937	53.89791	62.91121	65.11578	49.34154	53.97124	55.12663
Mcpt1l1	ENSRNOG00000053494	0.218309	1.316285	1.281635	0.038865	1.007962	1.119243
Mrpl28	ENSRNOG00000042720	96.97771	61.15661	83.95837	91.30769	48.76617	74.6162
Mettl26	ENSRNOG00000021615	18.05734	17.45459	16.08661	16.61446	15.27767	15.03943
Moap1	ENSRNOG00000033970	0.311525	0.288973	0.211765	0.221842	0.246575	0.15032
Zfp281	ENSRNOG00000058643	21.65871	18.7788	22.69891	18.45831	17.25502	20.5414
Npepo	ENSRNOG00000017505	20.81054	18.97448	20.48113	19.74802	18.42659	19.27323
Agpat2	ENSRNOG00000019466	20.69104	27.12218	26.03415	18.26103	23.22333	24.09058
AC130232.2	ENSRNOG00000060701	7.358116	8.72532	7.898854	5.293855	6.179259	6.74871
Homez	ENSRNOG00000014887	2.39085	2.990033	4.076261	1.618956	2.323233	3.727491
Tmem115	ENSRNOG00000021899	32.81476	37.12221	30.29074	31.36898	33.943	27.88865
Gga2	ENSRNOG00000018599	19.89075	26.67188	19.52833	19.0336	25.60469	19.0482
Wdr3	ENSRNOG00000019670	18.30274	16.10243	16.07081	16.12801	14.03733	15.07013
Nr1i2	ENSRNOG00000002906	0.016081	0.044751	0.044719	0	0.01591	0.029098
Tfap2a	ENSRNOG00000015522	0.022723	0.052694	0.094783	0	0.011241	0.071953
Nudt5	ENSRNOG00000017741	31.0358	21.5079	30.0332	28.27555	16.05545	26.7447
Acadvl	ENSRNOG00000018114	49.97933	41.48365	48.77388	43.73692	32.4087	44.53213
Rtraf	ENSRNOG00000000340	122.3942	118.7808	134.4569	113.3636	98.70996	118.9176
Cyp4f17	ENSRNOG00000062306	1.357347	3.365195	1.978813	0.975376	2.551586	1.161065
Ankzf1	ENSRNOG00000019052	26.30975	24.1308	20.30163	22.05547	21.22295	18.31141
Rbx1	ENSRNOG00000058914	256.0691	172.6633	256.5251	231.087	120.6543	222.8459
Usp20	ENSRNOG00000007710	13.42038	20.39665	13.73407	12.56736	18.98633	11.84731
Irf8	ENSRNOG00000017869	14.49057	7.862289	17.07996	12.91981	7.161877	15.77754
AABR07029054.1	ENSRNOG00000032439	37.1327	26.50412	34.91375	33.1153	22.22627	32.96759
Opa3	ENSRNOG00000025890	8.704164	9.98766	10.14841	8.06047	9.148897	8.824617
Xab2	ENSRNOG00000000988	19.46046	15.61902	18.41305	18.66105	14.65001	16.81581
Gart	ENSRNOG00000028292	50.03604	40.86349	38.01549	42.71998	31.44944	33.80221
Parp1	ENSRNOG00000003084	40.12908	25.51817	38.29971	37.68352	20.53513	32.87954
Plcd1	ENSRNOG00000032238	25.85351	33.43514	28.14954	25.10286	32.46787	26.60856
Npl	ENSRNOG00000002775	0.564872	0.413669	0.413379	0.412841	0.32356	0.336226
Sf3b3	ENSRNOG00000017724	77.65657	55.36925	76.10161	72.10907	51.42218	73.56899
Rpl3	ENSRNOG00000016896	418.9739	505.3761	461.1914	388.7123	439.6932	415.6619
Mccc1	ENSRNOG00000013293	5.377221	9.142232	4.913862	4.848884	8.681035	4.010525
Siva1	ENSRNOG00000028640	37.97415	19.5542	27.47883	31.07487	13.49784	24.417
Thap6	ENSRNOG00000058724	5.896537	4.76956	6.427838	4.887661	3.920416	4.737346
Clec2g	ENSRNOG00000059538	273.2526	203.3793	283.8642	252.8285	191.6588	273.2806
Fam229b	ENSRNOG00000024918	7.096342	5.286835	6.526223	6.56547	4.643826	5.459837
Ndufa11	ENSRNOG00000048320	241.4989	178.7424	257.52	207.3576	124.5221	231.3297
Ccl5	ENSRNOG00000010906	10.81691	0.31955	0.319326	10.22965	0	0
Tmem138	ENSRNOG00000020693	8.995396	5.592633	10.7974	7.522058	4.151706	10.12406
Map3k9	ENSRNOG00000007271	0.040517	0.159732	0.122062	0	0.070152	0.036657
Nat8b	ENSRNOG00000015851	6.21932	5.228243	7.67473	5.771386	4.307317	7.103942
Oaz1	ENSRNOG00000019459	760.577	753.4423	1083.193	691.4542	605.4294	931.3703
Mvp	ENSRNOG00000020182	83.17228	61.93532	54.25611	78.47139	52.51037	48.61907
Pkm	ENSRNOG00000011329	1219.487	712.6503	1618.764	1104.573	491.9852	1357.432
Prtfdc1	ENSRNOG00000024874	16.79237	15.00869	19.0913	14.21892	13.8398	16.59249
Jpt2	ENSRNOG00000024661	51.75034	33.09628	42.85812	46.79631	24.33358	38.28782
Psmd8	ENSRNOG00000037580	70.82179	81.14984	96.49129	60.12398	58.6241	82.03454
Otub1	ENSRNOG00000021175	68.71026	47.42538	58.40976	63.88443	45.16651	53.42535
Tf	ENSRNOG00000030625	16.70254	18.53886	19.68574	14.45692	13.71994	16.49283
Kif22	ENSRNOG00000020281	93.42661	28.73994	104.1684	84.83167	14.09705	96.78362
Rbm10	ENSRNOG00000008472	21.85981	18.79187	23.38774	20.26387	17.80526	21.18584
Rps27a	ENSRNOG00000004426	1046.114	1084.787	1186.23	944.9046	881.1241	1064.755
Syf2	ENSRNOG00000060597	49.16312	50.42572	40.82928	44.53349	47.22172	33.89884

Tmem161b	ENSRNOG00000032414	11.74535	11.20502	12.15012	11.3107	10.24757	11.20784
Eif3d	ENSRNOG00000005804	133.8209	98.03781	128.31	121.2801	74.12468	114.975
Stap2	ENSRNOG00000047306	5.774726	7.947827	6.971263	4.963424	6.457509	6.172367
Rpl30l1	ENSRNOG00000057677	0.3237	0.200178	0.200037	0.115256	0.106755	0
Ube2d3	ENSRNOG00000013741	160.8281	136.3976	159.9107	144.2878	114.3416	150.1801
Mesd	ENSRNOG00000012366	128.2741	107.6751	125.0903	119.3204	89.51954	114.2132
Cavin2	ENSRNOG00000025895	46.67445	44.01649	31.041	46.05813	43.488	29.98811
Coq3	ENSRNOG00000009974	15.59512	11.23664	14.29601	13.95365	8.682709	13.09431
Cox10	ENSRNOG00000024972	4.545492	3.853993	3.887513	3.937171	2.938029	3.464399
Klhdc9	ENSRNOG00000004022	0.990852	0.402116	0.707994	0.727652	0.265507	0.560291
Pccb	ENSRNOG00000015869	28.56922	24.43538	26.70066	26.66846	20.77855	24.65204
Maoa	ENSRNOG00000002848	44.6696	34.11189	40.48786	40.91778	31.63955	38.75095
Hadh	ENSRNOG00000010697	25.2591	24.03942	19.34395	21.3238	20.08686	17.54929
Snapc2	ENSRNOG00000001056	16.71543	16.81281	13.77971	15.80838	15.33961	13.07087
Fdxr	ENSRNOG00000058497	5.119411	3.632556	4.405171	4.793043	3.491067	4.133514
Tkt	ENSRNOG00000016064	186.2271	210.8469	293.2663	167.5041	169.2794	252.298
Tmsb4x	ENSRNOG00000047931	1810.703	1757.026	2275.374	1617.867	1625.058	2188.126
Qars	ENSRNOG00000060912	70.12157	85.96406	79.1663	67.36989	80.55336	72.81535
Timm23	ENSRNOG00000019811	62.02436	47.90834	63.60101	57.82997	42.68943	54.97339
Tax1bp3	ENSRNOG00000019357	64.56259	77.84487	71.92218	60.63962	68.85186	63.82991
Blmh	ENSRNOG00000003563	35.85813	26.10308	40.51383	33.5786	22.57922	38.87594
Tcp1	ENSRNOG00000014160	108.4733	75.21218	119.2987	93.17318	54.92831	110.4425
Xrcc1	ENSRNOG00000019915	18.43125	11.86732	17.15201	15.17118	9.815055	15.61288
Rps3a	ENSRNOG00000011893	373.0678	431.5867	437.5496	353.9442	388.3589	406.4942
Nsmce1	ENSRNOG00000015218	22.46283	14.83921	21.17225	20.81399	11.16717	18.63452
Edem2	ENSRNOG00000019299	18.19659	16.23224	19.23227	16.96758	14.00049	16.3911
Zfpl1	ENSRNOG00000050180	10.26222	10.17874	9.766102	9.949382	9.810646	9.136245
Shc1	ENSRNOG00000020657	77.20096	82.05296	72.16406	74.16267	75.33119	67.5996
Fam207a	ENSRNOG00000001225	17.71594	14.75003	17.36321	15.62361	13.70169	14.93375
Cnbp	ENSRNOG00000010239	351.7088	291.0142	375.4526	302.2591	223.3562	345.7646
Pnma1	ENSRNOG00000010553	0.714821	0.487555	1.500617	0.404235	0.353617	1.255416
Hsd11b1	ENSRNOG00000005861	42.84893	80.22182	30.23425	34.87589	70.3376	25.98327
Gltp	ENSRNOG00000001192	21.0175	17.89071	20.74882	19.04511	16.22101	19.90131
Rpl21	ENSRNOG00000032803	348.8546	346.5488	379.3534	319.8589	283.1896	337.6996
AC128960.1	ENSRNOG00000038045	59.44549	66.39898	78.17202	50.06587	44.80694	61.9776
RGD1310352	ENSRNOG00000006148	106.2438	106.4005	121.4762	98.99115	91.93619	113.18
Capn2	ENSRNOG00000034015	154.5267	151.7604	149.0278	147.2112	135.1364	137.1371
Pofut1	ENSRNOG00000010104	35.59238	33.50985	33.44345	32.71306	30.33451	27.79172
Nthl1	ENSRNOG00000012213	21.06183	16.08941	20.49521	19.4098	14.92939	17.96337
Lypla2	ENSRNOG00000010067	28.21683	29.57342	30.66235	26.09568	24.77513	27.29721
G6pc3	ENSRNOG00000020902	19.22128	18.3034	18.47987	18.26857	16.08765	16.76676
Armc1	ENSRNOG00000013253	28.9234	22.1575	29.71834	27.54477	20.32094	28.92422
Cnot10	ENSRNOG00000052305	27.0802	22.32475	21.8436	21.8289	16.89359	19.4502
Zbed5	ENSRNOG00000000918	25.14856	23.39808	28.39406	23.32972	20.53296	24.28351
Ap3s1	ENSRNOG00000003829	47.68888	52.5852	51.78994	40.9206	49.69797	46.21293
Tagln2	ENSRNOG00000008301	599.5125	379.9443	867.2647	555.682	278.2142	775.077
Tbl3	ENSRNOG00000013429	28.51982	23.48715	29.78192	24.70531	17.10084	26.70198
Hddc2	ENSRNOG00000021442	20.11662	15.56777	18.28063	18.83224	12.77489	16.48769
Rnf4	ENSRNOG00000013930	82.35165	78.91786	96.21013	77.26451	69.78129	91.5476
Zfp277	ENSRNOG00000045854	22.59595	22.50139	19.98038	19.45112	19.46297	18.59959
Tnfrsf12a	ENSRNOG00000003546	236.7585	183.5224	242.9951	199.4207	127.6546	217.8744
Vps36	ENSRNOG00000012654	24.15221	19.4533	29.65925	21.96928	18.50863	27.63147
AABR07026957.1	ENSRNOG00000019533	2.224539	3.231991	2.335341	2.061279	3.155548	2.158127
Acsl5	ENSRNOG00000016265	91.5844	90.79041	107.3	80.64324	74.24064	99.82571
Bag2	ENSRNOG00000012709	47.45594	46.71019	40.36123	39.95419	32.84745	33.11973

Dmap1	ENSRNOG00000019407	16.5658	15.47793	16.29052	14.54081	12.75568	15.11812
Plpp1	ENSRNOG00000009980	97.8623	88.20049	124.6229	90.78316	72.76273	114.7171
Mrps18c	ENSRNOG00000002178	46.59993	29.44913	38.42055	40.80316	21.2158	34.81218
Cpt1c	ENSRNOG00000026163	18.60952	15.78236	13.29788	17.3659	15.25488	12.25741
Wdr53	ENSRNOG00000001754	6.820099	6.350256	5.582403	6.377862	5.347243	4.889779
Wisp2	ENSRNOG00000010666	714.4531	748.855	517.9133	678.5652	670.9956	468.6298
Dynlrb1	ENSRNOG00000025715	133.0769	97.87593	129.6516	124.4722	79.21323	109.713
Cept1	ENSRNOG00000017723	27.11686	22.37722	24.80786	24.25463	21.15131	22.19418
Dusp14l1	ENSRNOG00000046368	9.874734	9.568292	17.78223	8.599596	8.338662	15.42127
Mapkap1	ENSRNOG00000017583	17.35752	17.34105	17.48753	15.17084	15.92471	16.49752
LOC100362339	ENSRNOG00000018471	552.9622	634.6655	668.7474	503.3404	518.0903	563.0382
Rad51	ENSRNOG00000037302	40.70279	11.52458	27.41797	37.92499	5.811747	24.09573
Tyk2	ENSRNOG00000032948	7.232127	7.934589	7.048028	6.987557	7.610842	6.523061
Hsd17b1	ENSRNOG00000019830	0.687036	2.132505	0.808315	0.592745	1.908511	0.621591
Phb	ENSRNOG00000046799	68.40466	54.90025	73.89631	62.98898	42.17096	64.31103
Xkr5	ENSRNOG00000028636	18.6798	5.67461	17.20359	17.28426	3.03479	15.80906
Fam45a	ENSRNOG00000010230	26.79591	21.75584	23.06067	22.38284	18.77658	21.11437
Zfp287	ENSRNOG00000003215	5.085293	4.519346	4.713858	4.257973	4.171126	3.992378
Nfyc	ENSRNOG00000010735	15.94578	14.82609	17.1696	11.70762	12.96854	14.27479
Mrpl14	ENSRNOG00000019734	44.34537	27.50224	40.59448	37.06912	20.17228	37.39261
Vdac3	ENSRNOG00000019277	220.051	168.131	219.543	200.2841	127.339	195.9431
Hmox2	ENSRNOG00000003773	20.58836	21.69325	22.00001	17.80793	20.50347	19.36038
Prkar1b	ENSRNOG00000028733	4.183749	4.657058	7.303876	3.426215	3.173497	5.488564
Atg16l1	ENSRNOG00000017913	16.22396	16.14553	16.36591	14.21111	14.66915	15.51111
Fn3krp	ENSRNOG00000036660	9.342585	7.657273	8.066273	8.515216	7.208987	7.643772
Atp5mg	ENSRNOG00000028884	338.4934	290.9148	356.2378	314.1456	232.4834	308.3148
Dctn2	ENSRNOG00000025481	142.9696	153.7151	161.1804	135.2842	135.3122	145.1286
Tmx4	ENSRNOG00000024852	26.94766	24.03249	24.38145	24.69933	21.24072	23.22037
Pagr1	ENSRNOG00000020217	5.075541	5.530893	4.659141	4.789927	5.314195	4.190811
Ngly1	ENSRNOG00000006143	22.19858	24.69854	22.94873	21.37186	23.05429	20.95722
Pnkp	ENSRNOG00000020318	21.52111	14.19442	22.13142	17.09979	12.17003	17.30565
Fam83c	ENSRNOG00000046772	0.048264	0.059693	0.089477	0.017185	0	0.014555
Tas1r1	ENSRNOG00000009708	0.502241	0.588484	0.637078	0.33883	0.496911	0.418526
Ezr	ENSRNOG00000018524	30.91385	21.45981	34.52373	28.01116	15.01619	30.45318
Skap2	ENSRNOG00000012228	30.07622	32.64863	32.77543	28.57893	32.01616	31.36388
Cetn3	ENSRNOG00000048563	10.43739	10.44927	13.42116	9.075524	7.304187	11.28572
Tax1bp1	ENSRNOG00000008393	102.2224	113.118	122.7353	96.54805	103.9536	118.5597
Zc2hc1a	ENSRNOG00000022356	49.29232	50.05187	40.19426	44.4874	47.62163	37.5973
Nosip	ENSRNOG00000020543	36.52992	27.31036	26.02437	33.13142	20.42023	22.19413
Lrrfip1	ENSRNOG00000019892	62.17137	74.81734	74.13175	57.54345	63.77453	66.04376
Nup107	ENSRNOG00000006541	31.30308	15.99792	24.3848	27.40008	10.43961	22.02275
Fscn3	ENSRNOG00000007942	0.334327	0.426422	0.5036	0.17856	0.351453	0.321385
Selp	ENSRNOG00000002794	3.39854	1.215032	4.7364	2.672256	0.782241	4.398679
Dvl1	ENSRNOG00000019423	21.20796	26.20275	25.10074	20.33278	24.08818	23.48746
Sf3b6	ENSRNOG00000046172	63.0866	62.63947	79.53103	56.17424	49.93361	62.83053
Aldh3a2	ENSRNOG00000002342	28.5873	38.70557	35.56452	27.37284	35.76211	32.95255
Nek6	ENSRNOG00000010897	106.1877	96.74434	131.0554	93.67844	70.35003	115.765
Clptm1l	ENSRNOG00000016923	55.78777	56.60024	61.42327	54.47337	53.47631	59.18707
Phldb1	ENSRNOG00000026985	49.95738	46.85683	48.85557	48.29212	42.83686	45.21056
Pgam5	ENSRNOG00000037443	30.91396	25.12652	31.31189	28.63207	21.98824	29.99865
Glce	ENSRNOG00000025372	11.66799	11.4357	10.8305	11.06164	11.1743	10.21832
Atg5	ENSRNOG00000000322	17.96081	20.05743	22.43207	16.41201	18.21108	19.19489
Pcsk6	ENSRNOG00000011526	0.715136	0.268705	0.562208	0.657525	0.170169	0.515973
LOC108348098	ENSRNOG00000020117	50.81055	48.79093	50.24846	44.66111	45.90754	43.22034
Mrps21	ENSRNOG00000024845	46.80705	46.03228	52.12983	41.0304	35.44384	46.87452

Hsd17b12	ENSRNOG00000009630	65.46347	52.05208	69.93075	56.07539	36.43472	62.75921
AABR07028749.1	ENSRNOG00000045917	0.077742	0.072114	0.144126	0	0	0
Fan1	ENSRNOG00000023985	2.941771	3.613833	3.289702	2.725408	3.275261	2.785796
Mst1	ENSRNOG00000019680	0.213694	0.325655	0.367874	0.130436	0.271834	0.331437
Emg1	ENSRNOG00000012828	29.51757	16.92005	30.97933	25.12301	14.79521	25.75801
Rps27	ENSRNOG00000016961	1747.572	1684.309	1554.59	1586.301	1613.553	1384.521
Arpc2	ENSRNOG00000014289	72.62869	67.92636	86.86022	69.00676	59.26142	80.70753
Arhgef25	ENSRNOG00000005034	78.20359	96.36443	96.0488	74.30851	92.57635	88.62835
Dmd	ENSRNOG00000046366	0.548673	0.658646	0.3989	0.491272	0.633325	0.362573
Birc5	ENSRNOG00000050819	39.14058	15.08143	63.51293	35.46872	6.468376	57.71014
Set	ENSRNOG00000025892	19.35731	14.39076	17.98185	16.66	12.32033	16.88071
Tmem9	ENSRNOG00000010204	71.36254	56.73352	62.48747	66.09809	46.48565	57.15496
Ech1	ENSRNOG00000020308	28.68959	36.6407	25.17099	26.37495	34.77727	21.18206
Crem	ENSRNOG00000014900	3.244491	4.129823	3.53737	2.923902	3.433086	3.124812
Anapc4	ENSRNOG00000004130	34.61716	32.9111	38.75766	30.20022	25.9006	35.66648
Faf1	ENSRNOG00000008523	48.78127	45.68485	47.25902	41.26312	40.55911	44.07918
Pex11b	ENSRNOG00000021216	15.97041	12.70858	12.37785	13.40772	11.57323	10.77643
LOC10091142	ENSRNOG00000051591	82.29243	58.3988	75.85965	71.04199	45.13934	70.49874
Coa3	ENSRNOG00000020487	52.71422	37.52875	44.19417	46.59973	32.42328	41.72534
Rad17	ENSRNOG00000018353	11.45636	12.45492	13.27762	10.66962	10.9314	12.49217
AABR07006873.1	ENSRNOG00000051256	7.250215	7.13652	6.705978	6.57775	6.311844	6.37339
Laptm4b	ENSRNOG00000006766	59.79371	60.77004	64.95331	56.52225	58.49459	59.76919
RGD1563300	ENSRNOG00000025630	0.231785	0.215006	0.214855	0.082529	0.152883	0.069902
Cd3eap	ENSRNOG00000016825	13.9889	11.33712	13.99086	12.46527	7.648655	10.4748
Tex2	ENSRNOG00000013659	11.80173	9.264583	13.45298	10.44301	8.687185	12.07414
Rpl21	ENSRNOG00000000957	813.5648	764.4052	827.5737	752.5223	668.1725	785.5132
Mocs2	ENSRNOG00000056325	35.72958	28.93443	33.57986	33.64718	23.96436	28.67016
Dmac1	ENSRNOG00000023946	13.21971	18.21433	15.80663	11.68314	15.33741	14.3759
AC114111.1	ENSRNOG00000052955	0.486073	0.676328	0.45057	0.129803	0	0.109943
Ndufs1	ENSRNOG00000011849	43.46837	34.14106	47.32787	41.16285	28.4561	42.22509
Cmtr1	ENSRNOG00000000532	64.45437	37.38843	53.69628	60.82472	32.55781	51.73058
Ppil1	ENSRNOG00000000523	45.55049	30.2186	40.36921	42.30681	22.36387	32.86059
Ino80c	ENSRNOG00000016532	12.24555	9.259532	12.92468	11.45309	7.722963	10.96093
Lmo1	ENSRNOG00000014629	10.30824	5.635294	8.866377	9.354177	4.763718	8.478462
Pycr2	ENSRNOG00000003267	37.31636	35.34917	41.28015	33.84279	27.08563	35.65724
Pusl1	ENSRNOG00000022354	10.91266	10.36207	8.799882	9.628536	9.702564	8.13868
Cox6c	ENSRNOG00000010807	273.0695	241.2507	321.9603	246.8318	181.5238	284.7457
Bscl2	ENSRNOG00000052393	37.84716	34.76949	39.93235	34.02549	25.29124	32.68413
Eva1a	ENSRNOG00000007118	4.831137	2.84089	7.417128	3.824298	1.792447	6.985692
Hsd3b7	ENSRNOG00000019080	90.13887	116.5817	63.40798	78.85507	103.3801	58.122
Eme2	ENSRNOG00000024867	21.89532	25.88324	25.92699	20.25062	23.41314	24.88284
Rab18	ENSRNOG00000018972	70.39386	69.24146	71.4934	65.21179	59.67583	66.83175
Commd1	ENSRNOG00000009281	31.03261	26.32126	31.63963	29.26238	25.57038	29.82237
Tomm20	ENSRNOG00000019980	203.4551	197.1501	201.0965	188.3489	168.6562	186.9518
Rab3gap1	ENSRNOG00000003953	43.62777	39.66399	39.14429	40.64155	34.84361	37.02765
Qpctl	ENSRNOG00000015413	10.80015	12.76812	12.30923	8.629187	9.742172	11.07316
Coro1b	ENSRNOG00000021828	200.6378	256.6556	209.5906	181.7897	222.1602	192.9749
Tspan12	ENSRNOG00000059016	6.849982	10.37517	9.531337	5.546997	7.626267	8.00451
Knop1	ENSRNOG00000048270	0.492771	0.396152	0.213163	0.421092	0.35753	0.178332
Mrpl22	ENSRNOG00000027039	52.72188	36.9191	45.78268	44.245	28.55341	42.28209
Hpf1	ENSRNOG00000011293	24.46299	12.32079	24.54639	21.13208	10.5907	20.20592
Serinc1	ENSRNOG00000029360	225.4961	249.0422	208.6526	205.9451	241.2674	191.3873
Idh2	ENSRNOG00000013949	125.2228	91.16915	133.8348	110.9453	68.50798	124.0674

Trmt2a	ENSRNOG00000001885	28.02082	19.27621	21.77587	24.33997	14.24934	19.75102
Bloc1s2	ENSRNOG00000012684	21.99625	21.74356	26.43099	19.93586	16.59687	22.58121
Dhx32	ENSRNOG00000018119	3.679675	4.56924	4.032064	3.440789	4.210557	3.882247
Snapin	ENSRNOG00000013356	52.74116	63.99753	55.91867	50.26125	59.173	49.67755
Mrpl41	ENSRNOG00000029875	48.734	36.01665	42.01468	42.44164	32.40798	39.1284
Usp5	ENSRNOG00000015409	31.64438	25.73467	34.84996	30.37275	22.96267	33.26213
Mfsd4b	ENSRNOG00000024543	4.055485	5.196961	3.820728	3.682762	4.395098	3.369238
Rab38	ENSRNOG00000016769	5.427228	2.746006	4.429046	3.938787	1.669979	3.829521
Mrps7	ENSRNOG00000003797	37.77512	28.263	34.98108	32.70678	23.95972	32.98416
Stoml2	ENSRNOG00000009535	60.06703	46.00157	58.6116	53.71638	37.63868	55.29034
Capg	ENSRNOG00000013668	169.9124	140.9755	154.3183	159.7569	115.5042	135.3139
Scarb2	ENSRNOG00000002225	87.39888	89.56057	77.88486	79.93776	82.64539	74.91007
Acat1	ENSRNOG00000007862	24.49455	23.77298	25.95363	22.80675	19.561	21.97478
Cops8	ENSRNOG00000019635	45.61055	40.97498	46.2339	42.57653	36.40433	44.33676
Mier3	ENSRNOG00000013121	10.01981	9.746628	9.001068	8.967304	8.772841	8.582333
RGD1311517	ENSRNOG00000020199	6.74728	3.596347	7.068524	6.063263	2.055681	5.329371
Camk1	ENSRNOG00000021781	49.57094	75.80846	50.98288	45.13394	64.80921	43.23195
Mrpl58	ENSRNOG00000032780	24.94207	24.56673	31.69579	22.71878	19.74187	26.01262
Hnrnpdl	ENSRNOG00000002270	43.21623	34.51759	55.41908	39.82843	31.22805	48.82778
Cox4i1	ENSRNOG00000017817	390.0283	391.3356	421.9636	368.3339	342.8427	366.5385
Ripk2	ENSRNOG00000009389	43.54551	22.15387	29.34332	39.41809	19.14927	27.70179
Klhl17	ENSRNOG00000020302	2.889803	3.15565	2.865223	2.696079	3.040089	2.780005
Atp5pb	ENSRNOG00000016000	27.19264	24.04228	28.26026	24.32207	19.32264	26.21616
Cct8	ENSRNOG00000001592	120.6309	93.19101	137.2241	112.6426	73.80018	124.3675
Ranbp3	ENSRNOG00000049785	49.37017	33.93621	41.81357	47.20122	30.04894	40.01996
Smyd2	ENSRNOG00000003583	58.00449	40.35963	54.65176	48.61472	34.98054	50.40845
Mcm3	ENSRNOG00000012543	62.61179	18.71033	43.2826	59.52063	10.75746	36.70679
Gpr132	ENSRNOG00000013914	0.109427	0.058003	0.115925	0	0.015467	0.028287
Slco1a2	ENSRNOG00000031249	0.083708	0.110926	0.354716	0.038321	0.070988	0.27048
LOC308990	ENSRNOG00000016978	0.034929	0.032401	0.145702	0	0	0.079005
Atad3a	ENSRNOG00000018118	20.57553	13.14291	18.18626	18.06269	12.15752	15.89028
Exoc3l4	ENSRNOG00000010111	0.683554	0.322409	0.515493	0.507396	0.252179	0.345908
Hint2	ENSRNOG00000015866	72.72744	74.47634	48.64598	63.99653	68.91248	44.98723
Mlf2	ENSRNOG00000016589	82.70931	77.33402	91.75709	78.00941	66.11807	84.62232
Atp6v1c2	ENSRNOG00000050553	1.944462	1.559957	1.924223	1.768279	1.221888	1.473956
Umps	ENSRNOG00000001797	63.00506	31.87669	52.64332	54.85853	23.29074	49.24576
M6pr	ENSRNOG00000059373	120.9836	105.7367	129.5165	111.8872	83.26613	107.0325
Fastkd1	ENSRNOG00000024335	3.886944	3.801518	3.818437	3.678074	3.270926	3.430679
Smpd4	ENSRNOG00000001875	14.30133	8.107022	10.73453	13.29993	6.353292	9.945519
RGD1564801	ENSRNOG00000048095	2.367964	2.534474	2.82215	2.056875	1.879414	2.471998
Bud13	ENSRNOG00000018665	9.343867	8.409685	8.629184	8.663704	6.942112	6.866745
Gmpr	ENSRNOG00000017250	3.644784	4.402511	2.96536	3.052994	3.450441	2.562158
Orai3	ENSRNOG00000039730	7.402546	9.989009	7.371524	6.417449	9.486673	6.078618
Nlrc5	ENSRNOG00000048222	5.325842	1.134933	1.249372	4.927145	0.783276	1.095117
Tmx1	ENSRNOG00000057934	87.38302	86.66963	97.50374	76.65917	68.41617	89.48941
RGD1309106	ENSRNOG00000026523	0.119267	0.129072	0.092129	0.042466	0	0.035969
Usp19	ENSRNOG00000049531	40.33706	41.43652	43.92533	39.23268	39.43607	41.12856
Timm13	ENSRNOG00000019682	112.974	100.5986	119.034	101.8421	72.36292	98.79404
Hopx	ENSRNOG00000024689	4.557989	4.026695	3.286165	4.134556	3.292732	2.225547
Ormdl2	ENSRNOG00000030120	52.66818	55.17638	60.00671	47.91857	48.84357	49.19243
Idh3a	ENSRNOG00000010277	21.44575	18.93046	21.87434	20.75562	17.38226	20.07957
Rps17	ENSRNOG00000019106	1272.962	1338.023	1416.582	1177.721	1102.869	1259.576
Hspbp1	ENSRNOG00000017795	51.55185	34.61322	37.39284	48.63223	31.965	31.84905
Sv2a	ENSRNOG00000021182	6.067661	7.431139	5.314719	5.27899	6.212069	4.813408
Adgrg5	ENSRNOG00000039891	0.298009	0.125653	0.276242	0.245979	0	0.196088
Eif2b2	ENSRNOG00000006467	49.87385	32.4954	61.14192	45.40491	23.89571	56.99081

Cdc5l	ENSRNOG00000019975	41.3891	39.29263	42.23339	38.12205	32.54951	38.74479
Map2k4	ENSRNOG00000003834	35.26121	33.70734	40.20216	33.10075	29.06136	37.68614
Tm9sf1	ENSRNOG00000019710	42.3111	40.56607	44.25711	39.7088	33.82945	39.1847
Ppp1r12c	ENSRNOG00000053828	45.02027	38.79278	44.2916	39.64043	34.23984	42.23775
Dimt1	ENSRNOG00000013596	16.37545	12.05885	17.92045	15.73372	10.37788	16.55709
Meis3	ENSRNOG00000021390	7.998522	10.12877	6.069465	7.177625	9.273487	5.734016
Nop16	ENSRNOG00000017284	35.14375	24.16721	29.99963	32.43428	17.15357	24.75853
Ddx59	ENSRNOG00000042451	11.78218	7.864522	11.6994	8.888226	6.036109	10.50169
LOC499240	ENSRNOG00000013713	0.09136	0.169494	0.056458	0	0.03013	0
Pcdh12	ENSRNOG00000019265	0.028174	0.039202	0.019587	0.007524	0.006969	0.006373
AABR07020879.1	ENSRNOG00000003952	0.111067	0.110952	0.110874	0.063882	0.092982	0.069568
Cct4	ENSRNOG00000009642	202.5696	179.8992	226.0774	183.7983	171.8084	204.8644
Metap1d	ENSRNOG00000061587	1.740766	2.24331	1.646108	1.222989	1.965029	1.405824
Lman1	ENSRNOG00000024470	89.71651	87.45343	91.26375	78.37725	70.71053	84.60061
Exoc1	ENSRNOG00000002144	16.05163	20.50258	16.90865	14.88959	19.91961	16.33419
Apbb1	ENSRNOG00000018020	33.54485	43.10574	35.57266	31.88936	38.81428	31.57427
Cep76	ENSRNOG00000021918	6.230278	3.894268	6.054658	5.855946	3.17045	5.707511
Tmem11	ENSRNOG00000005377	24.7207	19.36355	24.76798	20.91941	17.92442	21.65621
Sec61b	ENSRNOG00000006345	135.2304	149.3019	196.7621	117.4724	106.0974	169.4385
Aatf	ENSRNOG00000002778	16.37134	15.92604	16.84877	15.17822	13.33186	13.69147
Snx24	ENSRNOG00000017488	11.14982	13.09541	12.92723	9.363055	12.08371	12.1363
Fbln2	ENSRNOG00000007338	233.3119	222.5331	262.1111	218.1843	190.6846	245.5326
Nudt14	ENSRNOG00000014362	42.20325	36.84281	42.05264	37.0683	26.42119	36.83401
Fam92a	ENSRNOG00000016338	29.53304	28.47134	33.73104	28.54202	25.84541	31.523
Irf9	ENSRNOG00000019478	84.61482	34.52576	54.67107	78.26035	27.17713	51.89389
Rpl11	ENSRNOG00000026260	164.0541	167.7863	174.6572	150.6932	137.1722	157.0414
Hist1h1d	ENSRNOG00000048264	518.6653	120.1483	260.9177	493.1054	59.34011	224.0226
Wdr89	ENSRNOG00000021628	2.669286	2.392964	2.740017	2.315456	2.038321	2.026021
Atp5mc1	ENSRNOG00000007235	278.4393	210.9723	291.9777	254.5245	156.2842	260.6361
Ube2f	ENSRNOG00000019953	38.15478	41.94691	50.08896	33.99925	31.29173	42.67849
Pfkp	ENSRNOG00000017163	44.07435	41.2398	57.53131	40.62602	36.98549	49.84117
Tuba4a	ENSRNOG00000003597	28.18779	25.06479	30.86435	22.1874	15.41955	26.88693
Mreg	ENSRNOG00000015774	0.166147	0.106698	0.11847	0.109215	0.075869	0.092505
Aasdhppt	ENSRNOG00000005795	40.3837	38.1095	46.45195	36.89435	31.54166	43.40732
Mea1	ENSRNOG00000017144	98.33349	88.25067	89.17628	92.76997	74.20241	79.78074
Rdh5	ENSRNOG00000053850	11.23223	2.729176	4.580599	10.26167	2.36413	3.735484
Calm2	ENSRNOG00000030871	336.9822	202.2102	393.4885	301.3857	125.0228	352.2883
LOC103692128	ENSRNOG00000048146	0.046016	0.08537	0.127965	0	0.045528	0.041633
Cdc123	ENSRNOG00000017770	55.18717	45.98749	59.34445	50.83554	37.15061	54.95446
Txn1	ENSRNOG00000012081	860.4119	537.3913	1095.577	807.147	422.6914	953.3819
AABR07054368.1	ENSRNOG00000029996	978.0441	1147.81	1078.409	916.2626	982.7586	943.5929
Ube2i	ENSRNOG00000017907	123.0384	105.0039	126.2375	116.7956	102.5731	121.93
Ndc80	ENSRNOG00000013727	17.48714	6.850317	21.38971	16.163	3.956805	19.83619
Gosr1	ENSRNOG00000003971	17.27419	11.62391	12.15414	16.0643	11.17043	11.10649
Rrp8	ENSRNOG00000018766	6.597783	5.464429	5.910299	5.448554	4.484403	5.480215
Trappc2b	ENSRNOG00000049062	22.01034	23.22061	25.74232	20.47233	19.45482	23.38814
Knstrn	ENSRNOG00000009334	37.37946	15.51539	66.80425	31.43473	8.778	64.28203
Actr1b	ENSRNOG00000016789	26.03551	29.89532	33.83463	25.13096	27.46547	31.81802
LOC103692829	ENSRNOG00000061320	311.6513	357.3394	397.9116	285.5736	298.7119	327.8598
Zfp473	ENSRNOG00000026572	0.981983	0.498824	0.715203	0.811671	0.381684	0.433644
Klhl13	ENSRNOG00000014029	17.61071	20.89419	17.55583	14.79288	16.22577	15.61408
Dnase1l2	ENSRNOG00000042352	1.615017	1.414876	1.497055	0.91048	1.109636	1.095881
Ppp1r14c	ENSRNOG00000016368	21.89237	17.45318	22.60539	19.4319	14.05554	21.31548

LOC103693430	ENSRNOG00000020296	246.2969	175.5321	249.1518	214.5948	119.2178	224.5628
Zfyve21	ENSRNOG00000012185	36.61494	51.60343	37.37651	33.28472	45.97611	35.00839
Cdc16	ENSRNOG00000017536	16.74902	17.02549	19.70598	16.09465	15.53141	18.87063
MGC94199	ENSRNOG00000049287	7.258211	7.863887	9.347525	6.471203	6.357733	7.267274
Peg12	ENSRNOG00000024429	4.571075	4.601319	5.132759	3.881521	4.280024	4.774911
Timp1	ENSRNOG00000010208	558.5182	153.3972	332.7786	513.0165	105.578	314.703
Pde1a	ENSRNOG00000054212	9.590726	6.560002	11.76381	7.781736	5.865886	10.51775
Dnajc11	ENSRNOG00000008802	22.98063	17.3172	19.51152	21.75181	15.31399	18.69436
Ubash3b	ENSRNOG00000008187	0.674242	0.37526	1.085218	0.536883	0.278963	0.854022
Aldh1a1	ENSRNOG00000017619	0.034058	0.015796	0.063141	0.01819	0	0.030814
Dctn3	ENSRNOG00000014208	45.79739	38.54719	44.564	40.02515	32.8371	32.83517
Sirt3	ENSRNOG00000013828	11.64757	11.93771	11.22466	11.10757	10.53004	9.825689
Pole4	ENSRNOG00000006102	25.48957	20.52656	22.62491	21.04839	16.18471	20.93415
AABR07065438.1	ENSRNOG00000031889	385.7651	412.7015	404.6445	354.9996	338.3815	360.2086
Emc9	ENSRNOG00000019162	1.675136	1.291529	1.421703	1.475623	1.119254	1.348264
Aspa	ENSRNOG00000019659	3.656409	2.493454	2.367896	2.710794	1.998765	1.933507
Srsf3	ENSRNOG00000000520	57.9066	37.35928	65.83631	50.05671	23.70195	60.02633
Polr2f	ENSRNOG00000011214	55.03615	41.66377	55.1242	50.98392	31.87724	44.1586
AABR07036855.1	ENSRNOG00000056398	141.4486	135.4224	120.5753	119.1771	118.9752	112.2669
Arpp19	ENSRNOG00000023086	158.1716	105.5202	126.4945	145.7651	98.9587	120.8573
Mtmr4	ENSRNOG00000007496	21.83275	20.46193	19.49239	21.22627	19.10809	18.76658
Mlf1	ENSRNOG00000012827	1.148085	0.532487	1.252033	0.649247	0.133635	1.069273
Cpsf3	ENSRNOG00000052418	38.474	29.97986	37.16021	30.10424	25.15527	33.6224
Ttc30b	ENSRNOG00000059460	8.714677	7.880359	7.983272	7.62197	7.479309	7.02465
LOC108348079	ENSRNOG00000059412	8.060462	5.200022	9.68556	7.121838	4.660235	8.283004
Utp15	ENSRNOG00000016591	12.27348	11.9549	11.72137	11.72137	10.47511	10.6519
Rtcb	ENSRNOG00000004813	65.93118	64.42605	73.69483	61.29673	52.35939	65.59048
Cenpw	ENSRNOG00000042944	14.1425	5.177863	23.17536	11.79973	3.457489	19.09749
Rpl36	ENSRNOG00000033473	343.9644	311.0686	396.0063	317.8453	280.4988	338.1207
Il1r1	ENSRNOG00000014504	20.03381	13.87595	17.83933	19.42005	12.22369	16.63792
Kri1	ENSRNOG00000057447	17.85742	13.11164	16.30305	16.78894	11.01352	13.42297
Avpi1	ENSRNOG00000014828	77.34986	81.96833	74.9753	70.41285	63.84832	62.83625
Trim15	ENSRNOG00000031689	2.522346	1.929691	3.663844	2.138779	1.337834	2.705552
Steap1	ENSRNOG00000000017	6.864453	3.61915	12.99806	6.361046	2.583128	11.6249
Tspan7	ENSRNOG00000003229	12.56783	8.937822	9.851288	11.37562	6.173589	8.31853
Trim11	ENSRNOG00000002915	13.69097	15.41377	13.76978	11.46466	11.33869	11.99195
Mrpl23	ENSRNOG00000020354	86.50211	73.85444	86.88739	73.23616	54.28117	79.45748
Cct2	ENSRNOG00000021317	126.9357	113.3928	142.2489	114.8907	108.5238	134.9683
Ctdspl2	ENSRNOG00000022141	20.56263	13.7037	17.59636	19.14591	12.75281	17.05684
Phyhip	ENSRNOG00000049071	0.071636	0.0443	0.132807	0.025507	0.023625	0.075614
Pcid2	ENSRNOG00000025222	11.52349	10.87661	9.219341	8.709395	7.80475	8.09626
Uba3	ENSRNOG00000006221	52.36068	36.80976	43.37552	46.6673	32.29443	41.31599
Ube2n	ENSRNOG00000058053	49.39215	31.2471	50.04462	42.45572	23.70943	47.29166
Dtymk	ENSRNOG00000018904	84.00003	59.20255	83.46564	73.99429	39.87285	74.68053
Polr2c	ENSRNOG00000015720	53.46637	37.1969	50.87798	46.97208	26.51333	46.59144
Ptp4a2	ENSRNOG00000050044	109.8135	99.14137	119.837	102.9934	80.67657	106.6175
	ENSRNOG00000049104	1.459857	1.828947	1.886971	1.292237	1.473134	1.710193
Adprm	ENSRNOG00000003397	20.53513	23.43836	22.53415	18.98636	19.25812	19.57295
LOC100909441	ENSRNOG00000015544	436.6517	234.1455	642.0731	386.4405	122.7046	583.9607
Rexo4	ENSRNOG00000027867	33.80862	28.57351	35.50295	29.82994	27.1394	32.35252
Faap20	ENSRNOG00000036876	9.594577	9.943842	7.411483	8.761994	7.969216	6.296132
Ahsa1	ENSRNOG00000048981	63.65269	47.97702	57.92746	61.47556	44.9493	52.6803

Ints10	ENSRNOG00000055331	22.58309	17.69023	27.19668	21.18457	13.84425	24.31152
Dap3	ENSRNOG00000020373	39.63139	30.18573	40.96386	34.72319	27.97362	38.40958
Sgo1	ENSRNOG00000022499	5.739026	2.465489	5.67293	5.171862	1.767083	5.422092
Zdhhc2	ENSRNOG00000022686	9.695687	11.06158	16.35004	8.276105	7.344472	13.90222
Fcf1	ENSRNOG00000004723	78.06554	45.64051	55.66043	70.07559	40.78027	52.47161
LOC103694864	ENSRNOG00000047940	2.879244	3.355069	2.205736	2.516348	2.660341	1.894527
2-Mar	ENSRNOG00000007769	136.3947	141.6065	121.3525	125.5034	128.0441	116.4929
Mrpl16	ENSRNOG00000021005	22.51642	20.43171	22.41673	19.79272	19.3998	19.63239
AABR07024825.1	ENSRNOG00000046048	4.680039	3.937408	4.338204	4.127164	3.553536	3.397232
Tab2	ENSRNOG00000016054	26.0726	25.67771	26.5881	24.34253	22.31148	25.0596
Cdh16	ENSRNOG00000012222	0.424793	0.107466	0.536954	0.330003	0.063679	0.489145
AC129049.1	ENSRNOG00000031381	64.765	73.08192	65.5208	59.80084	62.44566	60.21967
Lypla1	ENSRNOG00000008320	79.99323	74.61655	55.23226	67.3764	66.36629	50.41752
Polr3h	ENSRNOG00000004471	51.82403	38.42915	55.05759	42.32997	30.82579	51.66039
Rad23b	ENSRNOG00000016137	136.8591	106.0329	143.4019	130.8814	89.40922	128.3412
Gucd1	ENSRNOG00000049952	18.31556	20.8944	24.59495	16.62242	16.14955	20.41986
Cog4	ENSRNOG00000017745	59.10639	45.68339	46.27899	52.72178	37.04343	43.16065
Trappc4	ENSRNOG00000011904	18.29034	15.32355	17.85213	17.26138	14.12654	15.54665
Rhoa	ENSRNOG00000050519	272.3735	261.4957	340.4183	260.7342	230.9248	320.6138
L3mbtl2	ENSRNOG00000024743	12.1527	9.579504	10.98468	11.66578	8.988395	9.871128
Adh4	ENSRNOG00000046357	12.80454	12.19288	14.72531	11.86224	9.576406	12.29341
Psmd5	ENSRNOG00000018809	56.75639	34.67352	60.67942	52.66919	27.46425	57.69
Fam107b	ENSRNOG00000014886	23.36232	19.98876	26.95376	20.29031	16.53893	25.72483
Jam3	ENSRNOG00000009149	51.24331	45.14501	46.03861	45.95163	42.22648	38.19854
Mrps23	ENSRNOG00000010363	26.4353	21.29154	24.36994	23.37112	19.20692	23.23455
Mus81	ENSRNOG00000020617	36.83549	26.68333	29.12417	29.65177	21.88844	26.43947
Mfap5	ENSRNOG00000015505	12.83849	19.43232	22.16996	11.02262	17.41435	21.45127
Mrpl57	ENSRNOG00000010855	12.44811	9.963753	11.22247	11.32777	7.745339	10.21228
Upk3b	ENSRNOG00000023686	4.389682	6.766982	4.742352	3.643211	4.93675	3.685811
Bin3	ENSRNOG00000018023	13.87776	10.88225	12.51644	11.37388	9.627821	11.3681
Irf3	ENSRNOG00000043388	37.54915	41.05417	37.19284	33.85938	35.11813	34.88985
Rfxank	ENSRNOG00000033318	5.521933	6.940572	6.679781	4.954648	5.599908	5.070868
Fam109a	ENSRNOG00000028206	7.350046	9.288244	7.405643	6.599504	7.166642	5.541584
Ube2m	ENSRNOG00000027514	73.18545	59.63663	72.36139	67.24345	50.77691	69.06955
Fsip1	ENSRNOG00000005888	4.699292	2.032851	6.387478	3.837148	1.184707	4.599141
Hars2	ENSRNOG00000016087	11.25258	7.679822	11.90615	10.54383	6.321181	9.96068
Avpr1a	ENSRNOG00000004400	10.01969	15.54559	18.8688	8.718442	13.08092	15.30542
B3gnt9	ENSRNOG00000053895	25.18252	31.73825	21.30475	22.93389	26.66983	18.69761
Katnb1	ENSRNOG00000014626	15.57212	11.44605	16.64354	14.36404	9.580472	15.94255
Rce1	ENSRNOG00000019468	17.60614	16.01138	15.16324	15.45929	11.61285	13.11802
Btla	ENSRNOG00000030246	0.034962	0.097293	0.032408	0.018673	0.051886	0
LOC100364335	ENSRNOG00000061058	109.656	91.56196	109.9561	100.7998	80.32025	106.0268
Laptm4a	ENSRNOG00000006865	373.0644	501.8666	411.0591	355.8393	453.3089	375.4377
Gemin2	ENSRNOG00000004360	12.26801	10.73809	12.06264	11.08613	8.662332	11.21977
Gucy1a3	ENSRNOG00000012302	15.63525	37.83031	10.87372	14.29656	35.81426	10.13171
Pde12	ENSRNOG00000059442	13.30107	10.44092	11.72142	11.84447	9.62174	11.12625
Tcta	ENSRNOG00000048237	22.91097	31.64203	23.72518	19.90054	26.22267	21.49105
Rps4x	ENSRNOG00000003201	900.1246	1024.404	1023.053	819.8449	837.144	923.9594
Vps28	ENSRNOG00000014633	55.99704	56.43535	50.19692	52.58925	48.90039	46.45549
Tmem9b	ENSRNOG00000013591	15.17502	14.12794	14.06662	14.10559	11.61042	12.7255
Osbpl9	ENSRNOG00000033593	31.76236	30.88198	33.44208	31.10687	29.31214	32.58836
Dbf4	ENSRNOG00000050482	2.699612	1.103242	3.079913	2.359356	0.691087	2.937777
Pex2	ENSRNOG00000008748	23.78344	19.12159	22.83977	22.56289	15.61104	19.6759
Atg4b	ENSRNOG00000018403	30.12694	26.72681	34.40041	28.62966	22.82072	30.10176

Rad18	ENSRNOG00000005907	11.23278	4.969794	9.4106	10.16139	3.343103	8.813248
Dhrs7	ENSRNOG00000005589	28.18367	30.89187	31.35553	26.71866	29.63991	28.49885
Selenof	ENSRNOG00000055257	365.7565	355.7729	393.7142	321.2438	282.8065	365.7192
Pacrgl	ENSRNOG00000004140	4.959321	2.925543	3.664959	3.759461	2.238533	3.184259
Eif2b5	ENSRNOG00000038160	33.32879	31.56482	37.37733	31.87858	28.51315	33.24138
Yeats4	ENSRNOG00000005689	30.89922	29.5664	27.20754	28.59684	24.47403	24.68828
Lgals3	ENSRNOG00000010645	89.89667	163.731	118.5217	78.33454	133.8585	85.14977
Vill	ENSRNOG00000011446	0.121387	0.2252	0.187535	0.014407	0.106755	0.146432
Wfdc21	ENSRNOG00000029668	10.37542	1.374905	28.3032	9.393963	0.879883	26.90965
LOC10255265	ENSRNOG00000059949	933.7024	258.9007	630.263	861.5551	107.1615	559.1612
	ENSRNOG00000049238	0.30096	0.459814	0.37744	0.283657	0.43789	0.33636
Tymp	ENSRNOG00000032394	22.00881	14.7561	16.3299	20.25072	12.42041	15.51947
B4galt7	ENSRNOG00000021886	28.06441	28.98559	31.27368	25.02768	24.6171	29.72088
Arpc1b	ENSRNOG00000000991	844.2614	944.9609	853.5174	778.3432	776.9035	755.6376
Mdh1	ENSRNOG00000008103	183.599	151.9774	203.5903	160.3751	111.8762	187.818
LOC10036583	ENSRNOG00000048109	847.6437	1030.462	1044.276	771.9365	834.8974	928.4453
Stat5b	ENSRNOG00000019075	30.48586	27.05453	30.49801	28.76932	25.16071	26.66713
Trip6	ENSRNOG00000048580	55.49245	64.20775	54.87853	51.50072	55.16424	50.3348
Ebna1bp2	ENSRNOG00000045760	51.43481	39.25857	48.80421	47.30891	29.27972	43.39453
Sdha	ENSRNOG00000013331	89.61004	93.37194	98.41212	83.01887	80.3543	92.71146
Eif4a2	ENSRNOG00000001815	182.2405	153.0818	142.6032	160.0557	128.1768	134.0727
B9d2	ENSRNOG00000028753	12.1781	8.386805	14.82342	9.493462	7.302331	13.32739
Sbds	ENSRNOG00000000888	49.93983	48.19859	43.92014	46.95694	39.54914	37.43291
Prkx	ENSRNOG00000060168	6.100828	7.284512	7.750679	5.17849	6.961248	6.858531
LOC100360846	ENSRNOG00000011827	12.20834	9.519849	14.74318	11.22222	7.795853	12.05753
Homer2	ENSRNOG00000061450	7.055835	4.983365	6.224844	6.465943	3.930291	5.801822
Sgca	ENSRNOG00000003998	5.41169	6.66581	2.857712	5.022516	5.551745	1.785905
Tcn2	ENSRNOG00000004280	42.43311	74.33897	29.49115	41.62978	73.53825	27.78252
Ttll6	ENSRNOG00000004939	0.008959	0.016622	0.024915	0	0	0
Dctpp1	ENSRNOG00000017850	44.36039	15.49841	41.39008	31.21666	7.545317	36.39515
Kctd14	ENSRNOG00000012494	8.668239	6.403148	9.763478	6.589496	5.043691	9.009035
Nmral1	ENSRNOG00000003794	23.32552	13.99533	20.02019	20.73015	9.005283	17.89339
Nifk	ENSRNOG00000025701	23.02018	19.57572	23.04444	21.1499	15.58846	21.17417
Rplp0	ENSRNOG00000001148	2452.684	2484.212	2372.48	2251.134	1988.39	2099.601
Casp9	ENSRNOG00000012944	10.12942	9.126397	9.456951	9.577509	8.667276	8.386197
Katna1	ENSRNOG00000014996	11.52516	9.727847	11.75984	8.890151	8.234413	10.71386
Aunip	ENSRNOG00000022030	11.43919	3.183328	6.547762	10.78332	2.178672	6.183842
Mad2l2	ENSRNOG00000009134	14.67036	10.62285	12.28057	13.13871	7.899133	11.19619
Sirt6	ENSRNOG00000006393	20.08181	17.68725	16.44222	15.69694	14.98512	14.80426
Commd3	ENSRNOG00000016383	102.9639	102.4461	103.5863	97.32665	91.15846	98.62693
Jpt1	ENSRNOG00000003661	87.71416	68.15182	137.7423	80.76874	53.65901	117.6512
Caskin1	ENSRNOG00000003195	0.373403	0.41095	0.363729	0.310975	0.388225	0.297752
Pgls	ENSRNOG00000018326	36.72736	35.40264	30.00158	35.92259	34.75833	28.46754
Def6	ENSRNOG00000000502	8.985732	11.75087	12.34662	7.914983	8.695461	10.22734
Tmem69	ENSRNOG00000029152	14.86119	13.62403	14.06234	13.93392	10.87941	11.88944
Ptges3	ENSRNOG00000002642	153.7727	119.0424	158.9267	145.5676	109.3021	155.6501
Nab2	ENSRNOG00000008415	25.16019	27.44314	32.22753	22.1114	25.52353	31.10927
Fam104a	ENSRNOG00000002851	16.0199	16.27427	14.99854	14.06959	15.59849	13.10257
Bfsp2	ENSRNOG00000010899	0.145445	0.044972	0.089881	0.129468	0	0.043864
Cox6a1	ENSRNOG00000001170	349.0224	290.5114	368.0362	326.4979	223.445	309.7112
Endov	ENSRNOG00000003699	5.657331	4.064464	4.98705	4.295283	3.100457	4.516261
Qprt	ENSRNOG00000016980	0.249703	0.26058	0.347197	0.100023	0.18529	0.282397
Pold1	ENSRNOG00000019681	22.05843	5.470429	11.46222	20.80446	3.773319	10.88426
Mrpl18	ENSRNOG00000014582	39.53587	27.75416	39.18957	38.36887	25.94646	38.53791
Snx4	ENSRNOG00000001786	35.00764	35.46342	38.77128	30.73979	33.71613	36.48204

Col9a3	ENSRNOG00000009531	0.092032	0.204888	0.153558	0.019661	0	0.016653
Gli1	ENSRNOG00000025120	0.067344	0.044621	0.080261	0.041106	0	0.008704
Ibtk	ENSRNOG00000027728	13.7393	15.51192	15.41518	12.51862	14.4001	15.00381
Dohh	ENSRNOG00000004259	73.58241	76.69256	80.55487	63.91966	55.17271	70.19297
Rpl13	ENSRNOG00000015335	724.9465	835.2848	860.8594	683.3675	735.0096	736.1737
Anxa5	ENSRNOG00000014453	403.9149	574.2248	373.8035	373.0688	491.457	323.9149
Fam136a	ENSRNOG00000016273	30.14213	21.91292	32.2074	26.35667	18.60998	30.94927
Anapc5	ENSRNOG00000001316	240.9596	203.302	314.4461	221.9928	150.8623	281.3573
Odf2	ENSRNOG00000014584	23.29323	17.86275	26.69605	21.93204	14.96643	25.37369
Tctn3	ENSRNOG00000047112	4.059936	3.200151	3.431904	3.437919	2.226955	3.083202
Sphk2	ENSRNOG00000021032	14.58143	14.92962	12.12822	14.4305	14.7074	11.73771
Atf3	ENSRNOG00000003745	1.122989	0.251444	0.239302	0.937578	0.153251	0.163497
Suclg2	ENSRNOG00000005686	60.78258	56.82195	61.23689	58.52777	53.13927	55.14569
Bzw2	ENSRNOG00000005096	41.55548	27.72063	39.99813	36.04793	25.09739	37.52305
Anxa4	ENSRNOG00000018159	124.3827	153.6942	117.7457	113.1953	135.9061	111.3229
Psmd9	ENSRNOG00000001339	49.71138	35.45336	49.99388	42.76706	25.25577	46.46847
Dnase1l3	ENSRNOG00000009291	0.10713	0.173906	0.049653	0	0.026498	0
Kctd2	ENSRNOG00000023764	15.79084	16.77675	12.91416	13.87636	14.51054	12.16857
Snrpb	ENSRNOG00000006961	107.2484	78.6478	118.1362	100.4388	61.33148	108.5707
Ftsj3	ENSRNOG00000009857	41.62914	27.40813	34.44052	39.33583	21.75908	31.44791
Fh	ENSRNOG00000003653	46.87137	37.04763	59.23908	44.27918	29.51917	54.11596
Cd151	ENSRNOG00000046094	621.0473	586.674	687.079	531.4083	443.0018	635.2948
Ssr2	ENSRNOG00000019940	154.1791	123.1316	204.0688	142.122	88.19432	180.4604
Tapbpl	ENSRNOG00000027552	41.27221	31.56205	34.95416	38.21632	25.43024	32.33315
Slc39a14	ENSRNOG00000009832	109.3647	75.90692	155.8778	103.5075	70.64435	143.8358
Pmm2	ENSRNOG00000002615	76.77077	62.11293	69.55632	70.74096	48.04758	62.57476
Mocos	ENSRNOG00000015113	4.839393	5.0825	5.318062	4.651284	4.910537	5.255954
Ilf2	ENSRNOG00000014154	44.83831	26.82786	42.13333	39.02712	19.54165	39.74892
LOC690468	ENSRNOG00000048701	657.9034	677.9764	749.7292	596.175	488.8881	598.2637
Srp9	ENSRNOG00000003211	30.25421	28.27389	34.7541	29.23612	25.23332	31.82347
Arl6ip6	ENSRNOG00000005074	28.49085	14.99438	22.34367	24.92759	11.06706	21.05411
Wrap53	ENSRNOG00000010520	11.90886	9.740361	9.656746	9.203181	7.29488	8.769468
AABR07037009.1	ENSRNOG00000003054	2.594432	2.894446	3.379905	2.396806	2.393457	3.108578
Nbn	ENSRNOG00000008580	21.6689	25.70389	23.64609	19.55298	22.46509	22.5214
Emc3	ENSRNOG00000009934	66.48171	60.70988	60.06388	60.84877	49.82463	55.60997
Camk2g	ENSRNOG00000009783	7.00121	5.986028	6.826599	6.320892	5.696264	6.490284
Ribc1	ENSRNOG00000003081	2.139628	1.261901	1.065131	1.777609	1.00619	0.944007
Taf6	ENSRNOG00000001355	33.96108	25.99421	27.48934	32.40536	23.14591	26.37602
Hist1h3a	ENSRNOG00000046434	888.0236	280.9355	573.4184	825.6025	121.4852	486.5233
Rps25	ENSRNOG00000027503	881.3536	955.4817	1043.917	824.6422	780.1628	899.8588
Pcyox1	ENSRNOG00000016704	42.32691	57.677	47.90575	40.6841	52.59674	43.6526
Glipr1	ENSRNOG00000026644	22.05503	16.33236	16.25221	19.67838	11.2222	13.95112
Dnajc24	ENSRNOG00000004842	10.18041	9.267747	13.25545	9.05362	8.620065	11.43835
Cops5	ENSRNOG00000006499	67.59843	46.73398	62.12976	58.39367	36.28779	58.74709
Aco2	ENSRNOG00000024128	44.98762	44.0971	54.14164	41.86839	35.18571	48.44888
Ltbr	ENSRNOG00000019264	94.26193	126.5327	109.5189	89.94093	113.5287	100.2634
Ppan	ENSRNOG00000020608	27.67869	25.18412	27.36322	26.05122	20.14601	22.91883
Cdipt	ENSRNOG00000024144	65.34317	53.26531	50.50028	62.34881	45.84501	46.6276
Cmpk1	ENSRNOG00000007775	48.06674	57.25776	60.89365	42.9126	46.96898	56.59164
Grwd1	ENSRNOG00000021058	18.14004	10.40426	14.51425	14.6721	8.475213	13.18707
Tra2b	ENSRNOG00000001783	88.40326	64.57062	93.13902	81.02676	51.68129	88.32118
Swi5	ENSRNOG00000022860	239.1634	198.4091	296.2365	226.0745	157.5511	263.1491
Ankrd50	ENSRNOG00000010534	15.66863	27.75082	16.72608	15.3054	26.6147	15.76968
Smad2	ENSRNOG00000018140	33.79682	33.2226	34.51867	30.3513	29.52078	33.31675
Ddx56	ENSRNOG00000004670	19.76271	15.57468	20.04517	17.4812	11.32778	18.38259

AABR07009834.1	ENSRNOG00000061630	1.452794	0.829308	0.621545	0.119372	0.110567	0.101108
Elovl7	ENSRNOG00000010450	0.50233	0.310644	0.271623	0.134144	0.041417	0.151493
Sphk1	ENSRNOG00000010626	4.590555	6.461093	6.106569	4.149945	5.082526	5.007299
Ptges2	ENSRNOG00000014050	18.21038	14.00966	12.35849	15.46507	12.62399	11.22987
Ostf1	ENSRNOG00000012156	116.0697	100.2798	118.3244	104.0307	78.82638	110.2509
Prkcsh	ENSRNOG00000013360	152.1929	171.5445	154.5586	141.0823	148.6024	144.7823
Ssr3	ENSRNOG00000011148	146.3902	134.7841	159.1155	128.1292	112.9059	152.1641
Pecr	ENSRNOG00000055295	20.31497	25.74828	19.02907	19.61466	24.62624	17.11035
Uckl1	ENSRNOG00000050277	21.77442	22.65903	19.37059	18.46324	19.10026	18.22319
RGD1359634	ENSRNOG00000011170	0.980546	1.230585	1.176257	0.739337	0.57067	0.417479
Brms1l	ENSRNOG00000008249	7.750035	5.139504	6.490776	6.571882	4.730705	5.217837
Nme7	ENSRNOG00000002898	13.71056	14.11727	12.95881	11.73924	9.48753	10.72297
Rer1	ENSRNOG00000014270	98.37577	92.47539	105.7231	82.80682	62.87481	94.03971
Primpol	ENSRNOG00000022320	7.716101	2.692929	4.231312	7.038466	1.492827	3.784302
Snx6	ENSRNOG00000005249	42.92718	37.13617	43.40917	40.27497	29.14037	37.95976
Dync2li1	ENSRNOG00000005151	8.857181	6.986672	7.923602	7.998218	6.512283	6.554659
Arntl2	ENSRNOG00000001830	13.8695	14.3124	18.2743	11.52282	13.55495	16.56108
Slc25a5	ENSRNOG00000039980	146.0245	143.1709	188.3809	130.5324	105.028	169.0799
Frmd4b	ENSRNOG00000007764	3.722618	5.069325	4.301576	3.059473	4.860356	3.760177
Tmem132a	ENSRNOG00000021338	71.97012	82.0759	75.82084	70.35851	77.06926	71.05387
Slc7a7	ENSRNOG00000010296	7.155234	11.86483	7.923913	6.064832	8.561973	5.671601
Tmed4	ENSRNOG00000005016	23.43177	26.81137	24.4075	20.83326	21.50289	22.19297
Rars	ENSRNOG00000007739	92.41781	56.42641	80.84465	82.93098	40.44862	75.14122
Utp6	ENSRNOG00000014209	51.13656	37.79193	40.41294	49.26146	33.18206	38.09536
Kif23	ENSRNOG00000014080	34.07072	14.49909	55.59949	30.85587	7.8206	52.7741
Rgs3	ENSRNOG00000024501	11.10277	17.15674	11.61879	10.27314	14.88817	10.31878
Zfp78	ENSRNOG00000015322	1.390709	2.127424	1.78669	0.964285	1.979435	1.523125
3-Mar	ENSRNOG00000023013	43.76204	41.27842	52.06581	40.56531	39.91673	47.77906
Srgn	ENSRNOG00000000394	50.1133	48.12516	39.72195	47.32971	45.80018	34.08037
Smim15	ENSRNOG00000031995	25.55803	19.00248	21.5767	23.98207	16.24411	20.57077
Bik	ENSRNOG00000010359	0.353496	0.255038	0.436902	0.293686	0.116581	0.248751
Mogs	ENSRNOG00000008648	40.69612	35.82608	32.63194	36.21275	29.14023	30.42626
Hmgxb3	ENSRNOG00000018132	12.83967	16.43913	15.10973	11.86672	15.88742	13.52112
Psma1	ENSRNOG00000011745	181.1251	140.3621	178.4205	165.8342	104.4584	161.383
Mrps12	ENSRNOG00000019949	50.03071	41.17702	63.90071	46.18957	30.02017	51.69084
Tyw1	ENSRNOG00000024352	19.32962	16.73326	16.42569	18.3055	13.90745	14.80169
LOC108349244	ENSRNOG00000005753	1.500884	1.511388	1.648202	1.372114	1.25084	1.541416
Rasl12	ENSRNOG00000032339	83.10057	46.62204	78.21482	80.02085	41.70308	69.68502
Cwf19l1	ENSRNOG00000012763	15.08468	10.28341	13.93768	12.6057	6.738574	12.79477
Prdx1	ENSRNOG00000017194	668.2819	530.4564	809.8683	621.758	382.5802	699.5852
Vps11	ENSRNOG00000010733	27.95899	38.46749	24.78365	27.09585	35.80555	22.95968
Psmb6	ENSRNOG00000019551	174.9151	129.7051	172.0423	160.5481	90.25992	149.7246
Adora2a	ENSRNOG00000001302	0.498641	0.43451	0.29414	0.274388	0.194349	0.218735
Slc30a6	ENSRNOG00000005856	12.50991	12.84762	12.94666	11.72355	10.62819	10.40435
Ndufs3	ENSRNOG00000009155	55.51204	54.66882	57.90183	52.96356	47.38099	53.54472
Dhrs4	ENSRNOG00000018239	26.21045	25.45629	21.19872	22.61437	21.62377	19.99684
Serpinb6a	ENSRNOG00000017962	150.1132	182.4047	211.1321	136.3886	154.2396	169.1072
LOC100911372	ENSRNOG00000007663	1477.078	1652.727	1683.966	1371.421	1338.232	1483.981
Gadd45gip1	ENSRNOG00000003011	47.89685	40.63607	40.92351	45.76801	34.75973	37.58818
Vcp	ENSRNOG00000034242	257.0247	257.9058	286.8105	239.273	214.3313	265.3649
Tmem127	ENSRNOG00000022727	25.18793	32.05628	21.87793	23.73764	31.23176	21.35358
Sync	ENSRNOG00000008118	21.56115	25.2508	22.45634	19.84989	21.9354	21.16179
Ldhd	ENSRNOG00000019036	7.731268	6.640363	5.892512	6.790196	4.306222	4.73229
Cd81	ENSRNOG00000020451	500.4135	553.4724	473.869	484.7802	503.83	437.7808

Uap1	ENSRNOG00000002926	52.87601	20.86138	35.58034	46.58286	18.93046	30.52826
Tmigd1	ENSRNOG00000003865	0.082927	0.046154	0.023061	0.017716	0.008205	0
Elovl5	ENSRNOG00000006331	90.5397	121.0248	167.8532	83.5242	101.2556	156.4142
H3f3b	ENSRNOG00000006532	487.7078	226.3273	313.3008	473.82	180.8819	275.4118
AABR07071244.1	ENSRNOG00000060723	130.4159	123.1736	178.9801	119.7807	94.27684	163.0733
Relt	ENSRNOG00000025075	3.890471	1.219711	2.9529	3.70683	0.684	2.35476
AABR07005779.2	ENSRNOG00000020000	12.74558	11.66735	13.307	12.00226	9.258615	11.47078
AABR07071986.1	ENSRNOG00000031947	122.1225	107.6395	143.8886	102.3343	91.42985	103.7161
Mpi	ENSRNOG00000018898	22.68801	21.36725	23.37272	21.16573	18.35475	22.18509
Atp6v0d1	ENSRNOG00000017235	48.26424	69.447	44.91072	46.29363	66.08287	39.23859
Pwp1	ENSRNOG00000005350	17.89794	15.09453	19.0454	15.78221	13.11836	18.39066
Zfp111	ENSRNOG00000024376	12.04597	9.288244	10.3563	11.7725	8.809764	9.56089
Obscn	ENSRNOG00000058068	0.11188	0.33976	0.108647	0.062599	0.250377	0.075917
Rps8	ENSRNOG00000054626	701.6727	759.8244	744.3395	673.9589	676.1838	654.5529
Ms4a6e	ENSRNOG00000030689	0.049756	0.046154	0.122991	0	0	0.015005
Eef1g	ENSRNOG00000020075	1092.021	1093.766	1105.275	988.1779	840.4293	983.2977
Psmd6	ENSRNOG00000006751	86.7686	74.3452	93.44822	78.89195	71.13482	89.636
Atp5f1b	ENSRNOG00000002840	653.9227	608.2936	830.9787	599.9165	461.8158	751.3573
Arhgef40	ENSRNOG00000052354	24.83841	26.92877	23.3371	24.67644	26.60294	22.83886
Rabggtb	ENSRNOG00000009992	96.61967	87.70771	100.8759	89.3833	70.91115	93.23173
Armc5	ENSRNOG00000019935	14.98283	15.94771	11.18972	13.82859	13.59419	10.24479
Ppp1r2	ENSRNOG00000001733	99.27767	81.50082	85.5924	86.41678	62.63067	79.59474
Ndufa12	ENSRNOG00000007407	94.46661	59.97603	103.2429	88.48486	42.53886	92.84038
Immt	ENSRNOG00000009097	48.11645	44.39798	51.17146	46.20443	38.75373	44.87151
LOC288913	ENSRNOG00000049931	91.99541	96.6294	111.6973	83.80362	92.31363	98.67414
Tmem38b	ENSRNOG00000028063	5.562318	6.485309	5.753466	5.118394	5.544867	4.360626
Rpl18	ENSRNOG00000021035	1019.891	1046.859	1141.802	913.9368	808.8287	1037.347
Gpn1	ENSRNOG00000004941	20.86269	14.86142	19.33886	20.07427	12.24946	17.0196
Slc25a4	ENSRNOG00000010830	440.6058	546.7914	566.0512	408.2107	454.9654	513.6667
Tchp	ENSRNOG00000001191	9.441237	9.245706	9.076701	8.096727	7.085838	8.35885
Prss16	ENSRNOG00000057865	0.236616	0.270138	0.18559	0.038884	0.036016	0.115272
Cth	ENSRNOG00000010658	0.763781	0.363819	0.650589	0.396901	0.183813	0.504262
Mcph1	ENSRNOG00000028586	6.62632	3.970363	5.781638	6.100097	3.159104	5.518819
Rhpn1	ENSRNOG00000007597	1.514992	1.379052	1.679953	1.194802	1.064648	0.960756
Gorab	ENSRNOG00000003861	13.1274	11.52311	13.51481	12.47101	9.863117	12.69337
Lamp2	ENSRNOG00000000164	162.9422	215.4054	142.1748	148.0155	190.3464	133.6335
Appl1	ENSRNOG00000013574	22.70301	23.47398	22.93626	21.64207	22.57333	22.61914
AABR07048308.1	ENSRNOG00000027547	0.19109	0.53177	0.619963	0	0.472654	0.432218
Larp7	ENSRNOG00000048989	22.11971	15.49549	26.08551	21.59547	13.75565	24.74153
Nabp2	ENSRNOG00000023480	45.99964	50.62828	53.27578	42.89709	40.34663	43.8624
Ddx47	ENSRNOG00000007838	40.60014	39.14085	40.64766	36.95656	37.6833	35.8306
Gtpbp8	ENSRNOG00000002044	12.68341	8.889291	10.01522	10.87193	4.98853	8.386828
Brix1	ENSRNOG00000018021	16.86722	11.40785	13.12831	15.12832	10.22416	12.58186
Rnf19b	ENSRNOG00000000123	30.60373	20.70949	26.9587	28.25218	19.824	24.00049
Dram2	ENSRNOG00000017744	9.977345	10.15072	12.6298	8.967239	9.818375	11.52632
Nde1	ENSRNOG00000058007	25.96873	17.95742	28.00042	21.3679	13.09675	26.5246
Aimp1	ENSRNOG00000011384	40.99617	27.03897	41.35213	38.10718	25.62796	36.9396
Pskh1	ENSRNOG00000019290	7.559038	7.278102	6.525431	6.804084	7.032514	6.047493
Traf5	ENSRNOG00000047957	14.73306	18.25663	17.43439	13.9437	17.89964	16.28727
Entpd7	ENSRNOG00000045548	2.049466	1.968999	1.865847	1.759169	1.882877	1.62246
Dnajc5	ENSRNOG00000015202	49.78739	47.66675	51.08179	47.49952	40.38409	46.17719
Rpa2	ENSRNOG00000013005	39.63325	15.12179	27.91398	34.10968	9.506082	26.19032

Capza1	ENSRNOG00000013538	72.08323	48.87946	66.36673	65.6634	41.46458	64.1676
Bid	ENSRNOG00000012439	33.55295	38.71267	31.10221	31.53152	31.96594	24.83117
Armc6	ENSRNOG00000020280	10.23887	12.26935	12.77778	9.341048	9.420685	10.88556
Eef2	ENSRNOG00000020266	1506.69	2219.316	1621.707	1423.796	1954.272	1443.34
Hbp1	ENSRNOG00000008927	32.56713	47.92041	36.60392	31.56717	44.76837	33.28084
Grm1	ENSRNOG00000014290	0.397317	0.126217	0.267393	0.267432	0.053849	0.221591
Ngdn	ENSRNOG00000018069	42.79728	36.91729	38.04973	38.00683	27.63115	34.56585
Stmn1	ENSRNOG00000016810	104.0618	54.4437	179.1529	97.34457	32.29954	163.2917
Cpeb1	ENSRNOG00000019161	13.65635	17.27518	11.78548	13.14603	17.12519	11.35568
Rpp14	ENSRNOG00000039829	40.76139	36.34334	50.34877	32.7527	33.87615	44.82673
Gipc1	ENSRNOG00000003864	67.30246	65.1689	71.51586	61.56789	52.32576	66.02483
Zfp212	ENSRNOG00000006711	24.11879	16.50877	16.92082	21.80371	15.8248	15.11412
Hmbs	ENSRNOG00000010390	26.1056	22.01811	24.34385	24.54292	18.02247	22.38533
Dnase2	ENSRNOG00000023830	42.31186	57.5064	44.14843	37.07347	48.34526	40.99533
Psma5	ENSRNOG00000019868	181.531	125.6882	203.6647	167.7505	91.42216	187.4033
Bcl7c	ENSRNOG00000018916	31.68447	26.17618	34.38291	26.52242	22.60686	32.80086
Gle1	ENSRNOG00000015237	18.40513	11.76089	13.54279	16.1082	10.96574	12.24584
Fip1l1	ENSRNOG00000002275	42.95053	34.75036	38.21956	38.68056	33.3187	35.70079
Nipsnap3b	ENSRNOG00000010332	46.82255	28.28125	68.10496	42.54201	21.98775	56.28689
Polr2g	ENSRNOG00000019439	70.3074	59.59619	79.59572	65.12801	45.05068	71.68641
Tomm70	ENSRNOG00000001640	28.2009	27.93924	28.06444	26.00058	26.57714	27.35145
Ccer2	ENSRNOG00000046971	0.364113	0.422193	0.379708	0.145851	0.315217	0.041179
Atic	ENSRNOG00000015511	84.86349	66.22642	69.4449	83.02768	60.8259	66.33082
RGD1597339	ENSRNOG00000019701	39.0068	31.09903	43.67029	37.18184	25.03628	39.35303
Fbxo46	ENSRNOG00000008815	7.466138	8.320321	6.491976	7.01428	7.030899	5.778317
mrpl24	ENSRNOG00000022234	25.25263	20.69315	20.83151	19.17384	16.87393	18.88298
Atp6v0e1	ENSRNOG00000003269	32.0372	30.76756	31.98964	29.3005	23.65293	28.47357
Ccdc90b	ENSRNOG00000009462	17.0074	17.03338	13.76988	15.35669	16.48509	12.79071
Kank3	ENSRNOG00000007230	3.035118	3.629028	2.774713	2.156401	2.93403	2.519267
Ube2c	ENSRNOG00000015131	10.19117	4.113319	13.25677	9.223975	2.026863	12.41116
Tubgcp4	ENSRNOG00000012798	17.21329	14.03134	14.32696	16.2414	12.49738	13.84163
Tor3a	ENSRNOG00000004307	50.68823	26.89817	33.74828	45.27512	21.89196	32.16948
Cdk5rap1	ENSRNOG00000015696	8.924431	9.673764	8.178728	7.895118	8.615049	7.864932
Wdr1	ENSRNOG00000028498	192.5188	208.047	213.8768	173.6674	171.8937	200.7124
Hif1a	ENSRNOG00000008292	29.26666	36.63146	32.07266	28.12163	33.44953	30.3918
Hist1h2bd	ENSRNOG00000060769	176.0485	34.28383	85.74067	169.676	14.00442	63.85373
Ufsp2	ENSRNOG00000012087	27.62405	31.83763	30.45571	22.79636	23.8238	27.8458
Mcrip2	ENSRNOG00000020029	8.288287	5.182161	3.565546	7.239195	3.760366	3.148654
Clta	ENSRNOG00000014635	280.0955	340.0402	273.7261	259.0819	326.4241	267.1948
Dph7	ENSRNOG00000008102	16.40168	13.23091	16.00133	14.81807	11.89335	15.54705
Stub1	ENSRNOG00000019798	42.83898	43.14314	49.89203	39.2445	33.0323	44.48569
Yipf5	ENSRNOG00000014564	58.49334	54.84211	66.85915	53.06615	41.76089	60.98511
Wdr5b	ENSRNOG00000002253	3.402512	3.306495	4.148997	2.639323	2.985675	3.81135
Plgrkt	ENSRNOG00000015932	18.1512	16.73517	18.52496	15.74594	12.84308	17.28474
Ppp1ca	ENSRNOG00000018708	192.7321	193.0768	245.4665	181.1498	152.614	210.0314
Kdelr2	ENSRNOG00000001083	108.5801	122.3263	158.1744	102.6353	101.9101	138.8294
Anxa7	ENSRNOG00000007136	135.0477	143.5019	119.9224	131.9133	132.5551	110.2684
Necap1	ENSRNOG00000009236	33.50516	23.42438	30.23877	32.38162	19.63484	26.46507
Pfdn1	ENSRNOG00000018653	101.3486	86.66565	109.9253	95.64187	68.52599	98.55061
AABR07049329.1	ENSRNOG00000030107	176.1292	191.2286	185.8623	166.3783	160.3385	166.605
Abcf3	ENSRNOG00000001710	32.23179	31.30776	30.88544	30.51534	26.12774	27.88129
Trmt12	ENSRNOG00000008978	6.93215	6.794708	6.575755	6.442131	5.441135	5.874598
Amdhd2	ENSRNOG00000006460	21.68259	35.75375	20.2171	20.54499	31.77487	16.6169
Akt1s1	ENSRNOG00000020289	21.73148	29.23506	20.95877	20.37165	28.73826	20.26306
Ap1s3	ENSRNOG00000049873	7.243208	4.534202	7.469329	6.771864	3.531872	7.075891

Ckap2	ENSRNOG00000024650	82.13968	35.58247	133.1906	74.20726	18.61174	126.4932
Rfc5	ENSRNOG00000001134	32.11062	12.27074	24.29136	28.96978	8.275422	23.15704
Ikbip	ENSRNOG00000008247	64.63378	78.36205	82.19625	62.94941	72.50619	77.85201
Ugt1a7c	ENSRNOG00000018740	8.703205	5.490202	10.42147	7.68274	4.814274	10.11354
Ufc1	ENSRNOG00000003706	77.08981	80.20851	91.93352	70.97408	62.87302	82.785
Vps72	ENSRNOG00000021081	34.75234	26.32868	33.47176	31.73821	25.452	30.48991
Scarb1	ENSRNOG00000000981	18.05992	15.74435	22.37168	16.71476	12.53575	20.97339
Slc43a2	ENSRNOG00000003835	6.971502	9.250508	7.516101	6.741393	8.427531	6.82169
Phyhd1	ENSRNOG00000016794	62.45298	46.50562	43.24614	59.81398	38.82857	39.08701
Elp2	ENSRNOG00000015301	30.94755	29.56227	33.52484	30.01663	26.23343	30.80811
Anxa1	ENSRNOG00000017469	1519.719	1298.172	1833.234	1444.521	1058.254	1686.227
Polr2b	ENSRNOG00000024779	42.61569	36.34609	43.60108	40.6001	29.80565	39.49426
Sharpin	ENSRNOG00000012812	22.36922	22.72505	19.88641	20.71486	18.93775	18.31191
Serpina9	ENSRNOG00000042391	0.089403	0.103663	0.082873	0.071623	0.044227	0.020222
Ergic3	ENSRNOG00000031085	90.54233	96.99339	81.71317	80.2468	90.19254	78.63693
St6galnac6	ENSRNOG00000046984	11.78104	15.13694	14.9668	11.23054	13.20395	13.10765
Col4a5	ENSRNOG00000018951	40.87577	80.63419	46.74356	40.04096	78.17167	45.3978
Stard3nl	ENSRNOG00000052429	25.52991	24.54983	28.08377	22.70972	23.60973	26.51111
Adipor2	ENSRNOG00000007990	61.81967	72.69899	83.87288	54.34846	62.01195	80.78124
Vopp1	ENSRNOG00000006646	40.14099	30.91652	41.10864	38.24481	24.98614	37.61347
Eef1a1	ENSRNOG00000009439	3641.024	4371.192	3957.118	3387.699	3559.611	3461.948
Oas1a	ENSRNOG00000001369	92.56086	11.50174	55.37868	87.97542	7.002305	54.06815
Nmt1	ENSRNOG00000002989	72.84943	68.25532	76.21428	70.42742	59.72078	67.97547
Med9	ENSRNOG00000053961	13.33484	10.09189	10.62145	12.41014	7.426172	9.210996
Ndufa6	ENSRNOG00000008569	132.9109	111.2299	133.7055	118.9307	105.9906	115.0591
Rab10	ENSRNOG00000047088	73.68657	62.92161	69.43343	70.65285	56.23942	66.78009
Nhej1	ENSRNOG00000018162	3.805477	5.208592	4.292229	3.496395	4.344331	3.852283
Fam126a	ENSRNOG00000010517	5.308071	4.815893	6.821108	4.396151	4.517928	6.302378
Ncbp2	ENSRNOG00000001746	28.38136	33.39147	34.10623	26.34679	32.05982	30.25919
Esrra	ENSRNOG00000021139	27.56971	27.72257	29.52743	25.3007	22.7625	27.57493
Tubb5	ENSRNOG00000061216	962.9022	542.1086	1158.221	907.272	381.1728	1073.043
Tmem97	ENSRNOG00000022657	69.31917	61.24179	124.922	60.89078	36.04298	111.0328
Fut10	ENSRNOG00000050857	4.950365	4.870577	7.92037	4.760105	4.198134	7.266009
Polb	ENSRNOG00000019150	16.59148	16.32043	17.92791	15.37341	13.15151	16.44483
Apool	ENSRNOG00000004512	20.42567	13.75945	20.71111	19.74162	11.40866	18.28723
Kif2c	ENSRNOG00000019100	27.23316	12.07856	40.99755	24.45809	4.94317	37.70799
Coq8b	ENSRNOG00000020848	12.48652	12.9785	17.38665	12.01741	11.57412	15.6733
Mars	ENSRNOG00000025459	61.70041	60.23758	69.36291	54.65093	43.57154	62.32938
Fen1	ENSRNOG00000020531	24.35854	8.96431	15.56037	21.60571	5.028673	14.43667
Slc20a2	ENSRNOG00000019490	23.894	39.0282	19.32762	22.47657	36.79167	18.65469
Rps9l1	ENSRNOG00000058909	267.2381	333.947	340.8077	245.0407	262.4256	297.612
LOC500124	ENSRNOG00000010594	0.32158	0.456224	0.245487	0.202061	0.411745	0.085573
RT1-M3-1	ENSRNOG00000000763	25.01925	17.24619	17.19653	24.14773	14.72384	14.0145
Pigu	ENSRNOG00000025181	10.32191	7.89997	9.331326	8.649063	7.379601	7.424742
Sqor	ENSRNOG00000000172	213.2724	127.9555	178.251	193.4233	100.0386	170.3595
Snrpb2	ENSRNOG00000004967	25.08137	24.82125	31.69759	22.3131	21.82147	30.87193
Dhrs7b	ENSRNOG00000005360	23.55203	16.48249	20.25201	21.06919	12.32825	18.95777
LOC103692173	ENSRNOG00000008720	35.99895	32.40766	47.10057	33.91484	28.74344	40.64731
Ift20	ENSRNOG00000008891	34.21441	42.37865	45.99654	31.45319	32.99344	39.8858
Gbe1	ENSRNOG00000051232	7.647935	8.230345	11.40089	7.278638	6.960905	10.06675
Prr30	ENSRNOG00000025726	0.056692	0.105176	0.052551	0	0.037394	0.034195
Abt1	ENSRNOG00000017585	20.9996	16.61955	18.92655	18.36131	14.2732	18.20979
AC096024.2	ENSRNOG00000034206	0.26917	0.749052	0.249509	0	0.133156	0
Lman2	ENSRNOG00000016161	152.2651	141.0662	159.163	142.0388	117.132	149.2567
Tcirg1	ENSRNOG00000017220	48.60246	36.65563	38.62705	43.75403	31.53697	37.217

Psmd1	ENSRNOG00000017730	95.71495	84.36143	108.0156	89.24851	67.57838	100.2945
Pygb	ENSRNOG00000007583	38.98882	39.00525	37.24894	36.75293	33.84495	35.14378
Rpl26	ENSRNOG00000004214	583.8246	616.516	600.6828	547.2815	495.6846	525.9577
Shmt2	ENSRNOG00000008106	61.87014	43.97928	81.85823	58.1243	30.72311	72.62709
Spr	ENSRNOG00000015455	30.38243	15.40055	34.98607	27.36992	11.47097	26.81235
Brox	ENSRNOG00000052963	28.12813	32.89123	29.55428	27.52394	30.95513	27.31008
Kazald1	ENSRNOG00000016058	51.78634	70.49932	70.36411	45.96963	58.77217	66.16434
Egln1	ENSRNOG00000019773	66.17406	60.75886	97.18772	58.4549	54.42092	80.53826
Krt80	ENSRNOG00000025994	0.330991	0.318402	0.204544	0.170231	0.254705	0.133094
Ppp4c	ENSRNOG00000019813	89.13841	65.39631	84.99854	86.32166	54.97213	76.80698
Ccs	ENSRNOG00000047816	32.093	27.75785	28.42951	31.27638	24.97943	25.41792
Pqbp1	ENSRNOG00000007766	36.60332	28.95265	37.78743	34.08091	20.71201	32.83135
Tcf3	ENSRNOG00000051499	34.47762	28.02264	31.82213	32.17684	27.21612	30.65767
Ebag9	ENSRNOG00000004220	14.75005	12.12377	14.30653	13.20969	11.61681	13.46873
Rack1	ENSRNOG00000052620	613.4509	692.3256	671.0484	584.2728	594.2468	609.8329
Pcyox1l	ENSRNOG00000019643	10.28503	10.60858	10.23934	8.651381	8.51529	9.676107
Fopnl	ENSRNOG00000053230	44.62026	27.60141	50.78642	41.59764	21.06478	48.3084
Hgh1	ENSRNOG00000029238	11.54079	10.1963	12.91757	11.20851	9.098444	11.67516
Slc16a1	ENSRNOG00000019996	20.609	15.25166	24.10005	19.93357	12.77192	21.733
Gfpt2	ENSRNOG00000002810	2.632828	1.279266	2.951872	2.303425	1.153592	2.495462
Uqcc3	ENSRNOG00000045561	31.50863	36.8501	37.93406	28.65763	32.52024	29.59038
Ndufaf6	ENSRNOG00000040040	10.24693	5.773256	8.574134	9.806884	5.579395	8.399749
Psmd13	ENSRNOG00000014109	92.17262	75.23744	105.1521	84.82247	56.86318	97.15043
Atp6v1d	ENSRNOG00000009080	113.0827	106.0001	76.76802	107.1848	96.46526	73.93049
Arcn1	ENSRNOG00000061108	90.99478	91.73566	114.2564	88.2216	81.25834	105.5149
Mrto4	ENSRNOG00000017979	36.85636	23.10922	35.62852	35.87614	19.65829	31.99164
LOC100362684	ENSRNOG00000030596	1354.794	1400.3	1548.407	1266.443	1074.788	1309.633
Zfp788	ENSRNOG00000014380	1.821718	1.551123	1.184581	1.379566	1.398864	0.959392
Ppp2r3c	ENSRNOG00000023591	16.20405	16.19809	15.86502	15.33922	14.91604	15.50441
Hmgcl	ENSRNOG00000009422	15.2575	21.45246	16.5589	14.78413	19.90211	14.7656
LOC10091141	ENSRNOG00000046299	45.15726	36.35044	51.13842	41.95606	31.60413	41.82729
Malsu1	ENSRNOG00000009035	36.29248	30.68105	41.42302	32.59196	29.44198	36.7465
Rrp36	ENSRNOG00000017836	43.70474	31.92834	30.95498	37.64333	23.09225	28.49591
Spsb2	ENSRNOG00000047113	3.794376	3.773115	3.995575	3.145172	3.123414	3.817423
Rpp30	ENSRNOG00000018718	11.9071	8.259626	11.01319	10.68273	7.931319	9.8515
Rom1	ENSRNOG00000019858	16.61295	16.42415	19.85728	14.60634	13.52897	14.08555
Cenpn	ENSRNOG00000011296	14.18159	6.773733	14.38568	12.52406	2.984473	12.89244
Atp5c1	ENSRNOG00000019223	22.66521	19.65658	25.64019	21.70871	16.15463	22.13117
Degs1	ENSRNOG00000003223	89.86188	94.48219	100.5441	84.62815	78.67036	92.17681
Rrad	ENSRNOG00000011901	6.766282	6.828239	5.720676	4.765432	3.604712	4.776306
Dnajb11	ENSRNOG00000001803	92.08992	76.90999	97.33905	77.99099	50.85797	88.88865
Gtf2h3	ENSRNOG00000001035	12.49262	9.687193	11.7949	11.2313	7.634653	11.18974
Pmpcb	ENSRNOG00000012693	68.40589	60.23908	71.23041	65.69102	49.94404	61.78161
Zfp180	ENSRNOG00000029336	10.7215	11.40034	10.16889	9.776965	11.0903	9.665583
AABR07033324.1	ENSRNOG00000061099	176.1565	171.1035	134.0696	141.6904	148.7553	124.2656
LOC100911252	ENSRNOG00000031045	1011.475	1012.77	1518.689	926.9648	722.2979	1337.411
Rbm42	ENSRNOG00000024278	60.47483	56.98478	54.79912	56.65672	46.53185	49.88111
Ttc39c	ENSRNOG00000050949	29.84122	15.83679	25.17421	28.77849	14.73552	24.88045
Rps11	ENSRNOG00000020595	516.1833	587.5891	608.8452	487.5656	485.4729	541.1823
Man2c1	ENSRNOG00000030654	35.13635	30.84962	25.91403	31.17528	26.75257	24.82068
Ltv1	ENSRNOG00000015217	16.41906	15.47016	17.02553	14.99047	14.69111	14.51332
Usp16	ENSRNOG00000001598	14.09557	16.95064	17.49375	12.85992	14.42242	16.60346
Slc25a32	ENSRNOG00000004403	6.133461	7.802246	7.721377	5.248239	6.808799	7.462723
Tmem52b	ENSRNOG00000058653	0.059249	0.09618	0.054922	0	0	0.026803

Tmem39a	ENSRNOG00000003075	23.65144	23.78574	25.64779	22.26777	21.59211	25.01672
Phka1	ENSRNOG00000003063	4.287878	4.655512	4.342813	4.162144	4.361951	3.895048
AABR07073045.1	ENSRNOG00000060344	82.18912	93.35582	108.9011	76.34492	76.53344	86.68044
Tnip2	ENSRNOG00000013805	18.78633	15.74698	16.97826	17.37975	14.72532	14.08245
Comt	ENSRNOG00000001889	126.1431	170.4926	150.5659	117.3607	148.9203	141.5583
Dus2	ENSRNOG00000019819	6.730141	5.955079	4.746344	5.365829	4.931666	4.386921
Map3k8	ENSRNOG00000016378	6.15858	6.411781	6.961304	5.940026	6.067504	6.300748
Bcl10	ENSRNOG00000042389	23.27953	25.66373	29.7998	22.12045	22.23903	28.06131
Copg1	ENSRNOG00000010474	94.82661	94.92489	105.2709	86.28352	75.90886	98.08189
Melk	ENSRNOG00000013598	23.02891	8.088812	18.48101	19.74404	3.93467	17.40454
Sgpp1	ENSRNOG00000005175	17.02138	16.99799	17.69591	14.49379	15.93688	16.67622
Egln2	ENSRNOG00000020947	47.54587	52.18502	49.15304	45.63995	46.12509	45.80364
Acy3	ENSRNOG00000017901	11.32973	11.24339	9.376023	9.68774	10.45472	8.793437
LOC102548541	ENSRNOG00000049135	0.12035	0.502369	0.167339	0.064277	0.416754	0
Abcb6	ENSRNOG00000018697	9.481452	9.662537	14.57997	8.171648	8.956753	12.27986
Aup1	ENSRNOG00000007842	58.8701	54.09439	50.2163	56.09067	45.13942	45.19297
Utp11	ENSRNOG00000007174	26.40976	22.09305	24.92971	23.58461	19.78735	24.20334
Ikbke	ENSRNOG00000025100	18.57145	18.61597	18.23711	17.8538	18.40879	17.43247
Abraxas2	ENSRNOG00000017222	18.96971	14.83194	15.89589	18.19119	12.23922	14.38072
Copa	ENSRNOG00000006247	100.5321	120.7058	122.3726	98.39931	112.4577	114.7792
Lrrc56	ENSRNOG00000016697	0.75839	0.523527	0.555858	0.32027	0.401345	0.271268
Mrpl2	ENSRNOG00000018057	25.08013	24.68151	20.97612	22.27438	19.26067	19.18777
Tyms	ENSRNOG00000037225	69.08945	40.67778	50.40239	65.39007	31.47246	46.55384
Atp5h	ENSRNOG00000003626	188.8807	186.1743	210.1542	179.7835	157.6327	194.806
Ppdpf	ENSRNOG00000012722	33.05463	35.99195	37.49612	30.99336	28.93244	29.3912
Pgam1	ENSRNOG00000050585	399.4177	203.087	511.4681	375.3719	167.9455	440.8016
Il1rn	ENSRNOG00000005871	318.5419	146.563	350.5392	301.3291	137.3844	320.3057
Rsrp1	ENSRNOG00000017309	27.77519	64.11589	43.39671	25.08725	62.89499	42.40366
Alkbh3	ENSRNOG00000021678	35.22468	28.74933	37.67944	34.18819	24.82604	34.88903
Iars2	ENSRNOG00000002368	30.89608	33.56555	31.71187	30.24156	31.60998	29.16553
Cd2bp2	ENSRNOG00000017201	62.15741	60.53348	56.75884	55.9658	49.45778	53.35016
Taok2	ENSRNOG00000019964	37.26985	39.01834	38.22034	35.745	36.50798	33.47869
Bloc1s1	ENSRNOG00000007784	133.0665	133.0312	143.037	121.0591	100.5371	128.3261
Etf1	ENSRNOG00000019450	81.05768	62.88659	84.10807	78.1435	59.76616	83.30878
Kpna6	ENSRNOG00000000127	36.61765	29.44538	33.80907	31.60861	25.51125	32.5338
Cenpt	ENSRNOG00000024178	18.25098	6.07498	18.59738	15.53582	3.683814	17.91104
Gadd45g	ENSRNOG00000013090	36.27454	12.78199	45.44007	32.45522	5.794132	43.26016
Naa10	ENSRNOG00000060063	26.06023	19.49492	28.4983	24.62987	14.81272	22.81723
Afm	ENSRNOG00000002878	0.037233	0.086343	0.05177	0.019886	0.018419	0
AC096601.1	ENSRNOG00000024177	0.403754	1.217209	0.46783	0.32346	0.898805	0.182647
Eif2s3	ENSRNOG00000060793	52.2158	51.40282	49.22411	47.71743	48.04493	48.06868
Abhd5	ENSRNOG00000000221	13.27157	18.9256	17.11062	12.35142	16.8176	16.30851
LOC100910678	ENSRNOG00000043400	13.8643	8.752384	12.67314	13.36969	6.858574	11.32411
Haus4	ENSRNOG00000039284	33.35547	18.95575	42.60372	30.08826	11.10441	39.47719
Nptn	ENSRNOG00000009029	106.5355	112.3678	106.8751	100.3153	96.61053	100.271
Prdx2	ENSRNOG00000003520	226.7951	256.8581	302.4909	213.4571	207.5515	269.9558
Tbc1d10b	ENSRNOG00000017349	32.04973	30.89749	29.6621	31.15768	29.54709	26.97373
LOC691807	ENSRNOG00000014654	50.48274	42.28086	50.59837	47.8709	34.50191	46.74339
Nars	ENSRNOG00000017852	108.6161	109.3007	114.3891	98.19644	85.05609	105.1084
Akr1a1	ENSRNOG00000016727	520.9832	438.9585	592.8498	482.1729	314.7869	526.2049
Arsk	ENSRNOG00000026937	7.13664	9.205073	5.817934	5.487654	8.127819	5.371551
Glod4	ENSRNOG00000007788	42.34265	35.83496	43.88441	41.43042	33.27491	40.33776
Cav2	ENSRNOG00000057713	27.40318	27.21209	25.59756	22.86877	19.74793	23.48147
Ing4	ENSRNOG00000023363	26.06464	26.39171	25.16847	25.16597	25.3204	22.74798

Dennd2c	ENSRNOG00000018716	0.345134	0.568428	0.600675	0.308473	0.466909	0.554416
Gmpr2	ENSRNOG00000020216	11.77426	10.68629	12.23007	11.23628	8.618705	10.12545
Rps6kb1	ENSRNOG00000003919	27.37581	22.70652	28.73331	24.4769	19.69096	27.97019
Dph1	ENSRNOG00000003116	7.432805	6.630377	7.11838	6.178851	4.961035	6.696902
Tmbim6	ENSRNOG00000055579	307.2785	370.7937	333.0702	278.1387	297.2808	302.7172
LOC102556347	ENSRNOG00000049911	15.27627	17.61072	16.50197	14.13346	13.58685	14.07502
Gnl3	ENSRNOG00000028461	44.39384	47.06299	43.53504	35.66856	43.6357	39.90261
Polr3k	ENSRNOG00000017843	27.8603	24.90501	28.06163	25.56173	23.21432	23.19975
C17h6orf52	ENSRNOG00000039379	1.591267	2.401372	2.575815	1.29384	1.268901	1.396711
AABR07068955.1	ENSRNOG00000033199	0.427911	0.2977	0.793311	0.228542	0	0.193574
Stk38l	ENSRNOG00000001828	11.05392	15.38812	14.69827	10.48591	14.02107	12.59325
Pdha1	ENSRNOG00000025383	70.04283	93.57947	100.0177	63.78978	74.94789	90.87444
Scnm1	ENSRNOG00000021092	19.13632	11.57675	11.90396	15.76541	9.878196	10.80045
LOC100912604	ENSRNOG00000046379	45.712	30.95758	40.94714	44.0663	25.22265	34.25637
Ppp2ca	ENSRNOG00000056485	85.31328	68.61316	84.1719	72.92948	53.3291	80.40115
Zw10	ENSRNOG00000054479	26.50779	16.97331	25.02007	23.77058	12.48677	23.76389
RGD1561849	ENSRNOG00000021663	5.230386	3.252157	8.41581	4.76966	2.530942	6.994909
RGD1305014	ENSRNOG00000020597	3.998457	4.113622	4.599313	3.649808	3.506471	3.469583
Birc3	ENSRNOG00000005731	8.711137	5.510149	5.521671	6.114726	4.465296	4.473597
D2hgdh	ENSRNOG00000019012	6.351752	7.637344	7.747631	5.407838	7.405437	6.903566
Spata13	ENSRNOG00000013707	0.745587	1.119321	0.554722	0.717824	1.028861	0.505923
Emp3	ENSRNOG00000021104	413.4012	324.9452	401.0628	389.8666	228.7642	323.5992
Cwc27	ENSRNOG00000013252	8.83031	6.184788	8.381302	8.233734	6.033555	7.783177
Fkbp4	ENSRNOG00000006444	80.98473	57.55365	76.17062	75.25558	51.46241	74.66746
Api5	ENSRNOG00000009689	67.79301	54.2309	71.63488	61.52359	50.69747	69.78583
Abcd4	ENSRNOG00000011964	8.200388	12.78172	15.52884	7.359303	9.34696	12.81374
LOC100360522	ENSRNOG00000013874	2837.307	3388.781	3138.271	2752.484	3074.213	2791.073
Plau	ENSRNOG00000010516	1.521992	0.613024	0.408397	1.048183	0.376459	0.25366
Ryk	ENSRNOG00000008593	120.1809	127.9268	150.03	103.0744	101.013	142.7765
Ano2	ENSRNOG00000023561	0.024908	0.034658	0.069267	0.013303	0	0.022536
RGD1564937	ENSRNOG00000030962	0.097026	0.112503	0.04497	0.05182	0	0
Wdr83os	ENSRNOG00000033611	143.2954	94.31063	126.8348	130.7172	61.18291	112.8314
Arap2	ENSRNOG00000056826	3.353154	4.892573	2.272689	2.73996	3.801695	1.952593
Gprasp2	ENSRNOG00000037658	5.526491	3.82086	3.818184	5.379137	3.525986	3.306109
Pid1	ENSRNOG00000029735	37.09659	30.3691	30.13209	34.62583	29.33414	29.18765
Puf60	ENSRNOG00000009960	90.60772	77.68448	98.65451	87.02752	64.95588	91.12037
Slc36a4	ENSRNOG00000011455	7.611142	11.17174	10.82348	7.399546	10.81315	10.14014
Vamp3	ENSRNOG00000030055	77.52011	74.43818	78.35007	71.78146	58.86677	71.59651
Golga5	ENSRNOG00000007699	21.19804	23.60431	23.03293	19.41515	21.85415	18.61081
	ENSRNOG00000000916	1.657518	1.123578	1.75313	1.112249	0.98816	1.211237
Gtpbp3	ENSRNOG00000023403	3.57117	3.821229	3.310328	3.339783	2.858867	2.533847
Tmem2	ENSRNOG00000012782	17.85273	7.81533	16.81702	15.96691	7.179835	15.91937
Thg1l	ENSRNOG00000005539	6.454516	5.852552	7.404052	6.153388	4.645822	6.190057
Ufd1	ENSRNOG00000047394	42.44508	36.10406	41.6425	40.24035	27.96936	36.60411
Naprt	ENSRNOG00000007939	27.67962	24.61616	20.53672	25.66579	20.30776	19.06177
Inpp5k	ENSRNOG00000056954	28.52107	41.96148	27.68095	27.34476	39.23559	23.31264
Rab32	ENSRNOG00000014258	97.71378	77.11097	94.00239	86.12709	66.30998	91.20269
Ccdc17	ENSRNOG00000023216	6.380789	6.236241	5.391443	5.335692	5.432959	5.13841
Pygo2	ENSRNOG00000020663	21.59424	21.26531	20.73355	18.81593	16.71339	19.49137
Cct7	ENSRNOG00000015630	115.91	94.13875	127.0592	108.6623	73.4793	117.6869
AABR07052523.1	ENSRNOG00000028543	180.5109	114.9671	279.8937	172.0163	79.45235	247.6757
Spryd4	ENSRNOG00000003127	13.18539	11.2411	11.89262	11.99472	7.716669	10.22847

LOC100365810	ENSRNOG00000045885	291.8342	322.2713	382.8717	268.4751	250.8577	287.0161
Mrpl54	ENSRNOG00000020464	69.56732	55.46724	77.36049	66.62961	47.66952	65.83179
Ptpn11	ENSRNOG00000030124	65.13807	62.64911	71.68082	62.177	56.08145	69.36782
Bop1	ENSRNOG00000021773	27.09779	19.23498	22.74569	24.83558	14.98613	21.46053
Pomgnt1	ENSRNOG00000023455	41.07976	28.73037	34.44716	37.12158	25.42461	33.50883
Tars2	ENSRNOG00000057194	16.51142	11.69408	14.03489	15.91764	9.240347	12.18447
Selenot	ENSRNOG00000013507	107.1955	84.76235	116.6979	99.82151	77.07422	114.8389
Smim8	ENSRNOG00000023035	4.40249	3.917588	4.484276	4.10113	2.785663	3.774702
Usf1	ENSRNOG00000004255	41.67911	45.1021	44.40832	39.27444	37.6807	40.7587
Dph2	ENSRNOG00000019735	7.091649	6.448972	6.525213	6.793413	5.395889	5.265315
Ndufs7	ENSRNOG00000024568	72.68688	58.86815	63.04912	64.76325	41.99799	57.34777
Rsl1d1l1	ENSRNOG00000048608	11.47409	9.094876	9.628904	9.623909	8.442128	7.000647
Tpm3	ENSRNOG00000017441	146.5551	83.53233	146.9045	142.3266	66.21123	129.7948
Myg1	ENSRNOG00000013343	49.0207	39.92666	40.34801	40.07454	34.81671	37.80224
Vps4a	ENSRNOG00000020351	35.54127	30.74033	35.72843	31.24938	29.27862	33.73463
Pole	ENSRNOG00000037449	13.41683	5.163586	8.39904	12.41162	3.028326	7.681197
Gpx8	ENSRNOG00000010461	222.0774	187.7327	200.1539	213.7261	161.3008	186.8478
Rps15a	ENSRNOG00000018320	420.5087	447.0856	448.6846	383.6021	339.5225	399.2338
Med23	ENSRNOG00000013422	16.6179	16.78545	16.22881	15.18832	16.41008	15.3224
Fuk	ENSRNOG00000059453	9.060899	10.20345	8.290708	8.304885	8.646626	7.783487
Maea	ENSRNOG00000005397	85.89149	95.35571	91.9216	83.91188	86.9497	85.08881
Il17rc	ENSRNOG00000027376	14.75661	15.6821	14.85336	14.22064	14.98085	14.68603
Prim2	ENSRNOG00000012486	21.50807	8.171196	14.02746	18.21475	6.138074	13.14857
C2cd4a	ENSRNOG00000034236	0.513869	0.680956	0.34024	0.235244	0.363154	0.265668
Atp6ap1	ENSRNOG00000054352	114.8803	146.6124	101.623	109.4761	131.9243	95.36306
Caprin1	ENSRNOG00000009152	178.1855	151.4288	188.9333	164.1416	133.3316	184.6387
Zfp523	ENSRNOG00000000501	5.023447	4.300284	4.269637	4.920087	4.232721	4.059416
Smug1	ENSRNOG00000036842	8.415426	8.482352	7.872416	7.160646	8.096647	7.224114
Slc35b1	ENSRNOG00000004510	69.6701	69.40776	73.53686	59.73802	49.19746	67.04352
AABR07027870.1	ENSRNOG00000059510	21.37132	28.38493	20.25107	19.33829	27.70615	17.44942
H2afz	ENSRNOG00000010306	526.0792	329.0796	813.8744	493.216	199.2975	727.4854
Orc1	ENSRNOG00000008841	13.09957	3.532188	8.544906	10.30209	2.574651	7.262681
Napa	ENSRNOG00000001494	48.1274	46.16173	41.93993	41.30946	37.12644	39.78541
AC109542.1	ENSRNOG00000048562	54.40557	50.79059	70.00816	51.65748	38.99089	59.88103
Trmt10c	ENSRNOG00000039567	16.05975	12.81076	13.99698	12.60964	10.60231	13.10416
Dcst2	ENSRNOG00000058618	3.560695	4.403909	3.564304	2.881398	4.056676	3.354633
Atcay	ENSRNOG00000020407	0.100078	0.102116	0.166982	0.03207	0.069311	0.144871
Coq5	ENSRNOG00000001171	13.89728	12.06459	14.1548	13.35253	10.05616	11.81077
Ptgir	ENSRNOG00000016756	0.316272	0.252911	0.62678	0.279587	0.140272	0.473619
Utp23	ENSRNOG00000004387	8.362765	8.220962	9.079962	7.509326	5.472051	7.686727
Wdyhv1	ENSRNOG00000006700	5.868459	5.714773	4.77287	5.307845	5.561468	4.109227
Ap2a2	ENSRNOG00000019534	81.74966	78.4683	86.97897	78.62014	68.51743	82.03123
Leng1	ENSRNOG00000061893	3.844414	4.547265	4.487515	3.302587	3.159613	3.938286
Gpx7	ENSRNOG00000009751	116.5796	110.9507	149.683	108.1395	87.0249	139.1844
Prrt3	ENSRNOG00000009863	0.185032	0.22885	0.125779	0.144941	0.170864	0.111604
Mydgf	ENSRNOG00000046883	132.3268	130.7208	167.4627	123.2266	91.16073	134.2521
Shq1	ENSRNOG00000005433	4.844641	3.562119	4.125392	3.952501	3.346448	3.49732
Eef1b2	ENSRNOG00000024186	216.1014	252.7553	272.4629	206.9738	234.4105	239.9441
Evc2	ENSRNOG00000007025	14.33527	13.82084	12.7481	13.57385	13.58834	12.3589
Fuca1	ENSRNOG00000009325	144.2464	172.7956	118.501	139.852	164.2119	115.8852
Rab8a	ENSRNOG00000014621	69.28173	46.18144	51.96017	65.58279	37.98186	49.15559
Apoc3	ENSRNOG00000047503	0.865188	0.321022	0.588129	0.800949	0.057067	0.313109
Adipor1	ENSRNOG00000004143	125.0897	139.0829	122.8455	115.5141	115.4512	113.8786
Poc5	ENSRNOG00000018193	3.698145	2.527686	2.931867	3.023339	2.286808	2.641476

Nucks1	ENSRNOG00000047287	87.98408	57.10416	125.8916	84.76752	44.26684	117.5073
Tmem161a	ENSRNOG00000020390	22.26648	22.86345	20.98604	18.08989	16.21054	19.27724
S100a4	ENSRNOG00000011821	3070.405	3490.933	3918.052	2742.587	2628.647	3570.943
Pi4kb	ENSRNOG00000021024	34.54433	36.4705	32.98376	33.59532	32.93168	30.89392
LOC100911807	ENSRNOG00000050492	3.517377	3.093257	3.916792	3.025257	2.847309	3.037671
Wdr61	ENSRNOG00000012803	41.16204	39.3379	41.41214	35.47802	30.17408	39.0445
Prpf31	ENSRNOG00000061039	25.2254	16.61858	25.1493	23.82518	11.88387	22.66705
Pgm2	ENSRNOG00000002185	22.14117	17.79549	19.74791	21.34837	14.55712	17.56633
Ido2	ENSRNOG00000025365	0.776451	0.605004	0.374264	0.099526	0.338012	0.112398
Rbpjl	ENSRNOG00000026295	1.21687	0.913774	1.181703	0.943925	0.802637	0.747076
Pola2	ENSRNOG00000020906	19.88566	7.029608	11.35569	18.09168	3.988476	10.54224
Tmem30a	ENSRNOG00000010895	70.07425	75.10826	71.85742	64.29087	64.2999	68.7296
Rps5	ENSRNOG00000019453	896.9092	884.1134	1001.563	861.2174	725.8695	868.4923
Tmem101	ENSRNOG00000054581	24.36818	26.67288	24.39537	23.35554	22.29036	21.10493
Cul1	ENSRNOG00000005310	64.27753	60.32806	71.12277	59.2818	52.43688	69.12549
	ENSRNOG00000031367	26.89538	22.32824	34.37962	25.94134	18.28673	31.50009
Gcsh	ENSRNOG00000011535	37.85026	31.59083	41.28215	35.73469	25.79986	38.8761
Eif4a1	ENSRNOG00000030628	380.8162	292.1861	426.6847	357.6277	213.8784	386.0561
AABR07050701.1	ENSRNOG00000055406	4.405767	3.250885	2.1348	2.246107	1.684161	1.630671
Zfp105	ENSRNOG00000032919	11.96703	12.21254	11.90886	9.742199	9.505368	11.30151
AC118165.1	ENSRNOG00000032625	4.586155	4.667756	4.384028	3.606073	4.442948	3.428928
Vcam1	ENSRNOG00000014333	813.9459	1023.561	909.0956	721.0821	778.6793	811.5798
Pin1	ENSRNOG00000020474	102.2149	59.26904	84.02483	83.25339	49.88222	78.19142
Tuba1b	ENSRNOG00000053468	575.38	306.1135	849.9242	544.8895	178.5447	762.6025
Atp5f1a	ENSRNOG00000017032	336.0375	346.8242	424.4217	310.2012	256.686	376.2806
Kin	ENSRNOG00000026690	9.36809	8.499978	8.327941	8.80376	7.419992	8.012558
Il1rl2	ENSRNOG00000014683	9.456758	6.387816	4.837817	8.190633	4.682489	4.446885
Ssx2ip	ENSRNOG00000015425	9.506606	6.234327	11.1417	8.073833	4.51975	10.7601
Oxnad1	ENSRNOG00000019760	9.516252	6.119966	7.575389	8.671444	3.989057	6.767443
	ENSRNOG00000006796	45.45198	33.95915	43.12207	36.73241	27.51427	41.12235
Pla2g15	ENSRNOG00000019859	15.46303	13.56452	9.100495	14.54634	13.32354	8.558483
Tmem184c	ENSRNOG00000012860	11.44892	22.51773	14.99618	10.31528	21.70265	12.52007
Pdk3	ENSRNOG00000012513	16.47684	9.08363	16.12532	16.01149	7.919511	14.2723
Zfp708	ENSRNOG00000049662	2.6031	3.82499	3.053578	2.313038	3.715061	2.93871
Chac2	ENSRNOG00000001531	5.666621	5.660746	8.94695	4.656109	5.113599	7.042355
LOC680121	ENSRNOG00000009920	708.8921	773.2795	613.1417	631.6412	631.525	573.3719
Emilin1	ENSRNOG00000008246	260.5633	231.622	368.862	252.0827	194.2078	340.6265
Pqlc3	ENSRNOG00000005126	135.3309	168.0564	105.6457	119.0197	141.6489	98.95197
Rps18	ENSRNOG00000033152	330.1138	387.51	367.1382	304.888	313.3811	334.4581
Crot	ENSRNOG00000006779	23.45967	19.21093	16.92776	20.83625	18.58207	15.17306
LOC100360117	ENSRNOG00000048456	102.3141	124.8783	110.9402	99.85807	115.3877	99.76625
Asl	ENSRNOG00000000903	45.46196	51.01006	40.81838	39.27952	49.63105	36.02319
Tldc1	ENSRNOG00000016224	7.542985	6.663751	5.583386	5.961758	6.232773	4.699629
Ndufs8	ENSRNOG00000017446	61.09266	54.38838	71.19516	56.57093	38.83927	63.13038
Fam103a1	ENSRNOG00000019426	21.59907	16.6889	23.14568	18.7108	11.50683	21.73198
Rpn1	ENSRNOG00000046345	253.2239	280.4698	309.3023	227.1218	207.0514	278.5975
Abcg3l1	ENSRNOG00000030216	5.774791	7.262976	5.30477	5.084833	5.932259	4.918749
Sin3b	ENSRNOG00000048622	97.12395	86.08257	100.7906	86.65447	65.37564	94.64817
Ctsl	ENSRNOG00000018566	440.0083	572.2828	318.343	409.0977	497.384	291.401
Psmb4	ENSRNOG00000020979	416.2629	348.4752	408.5929	386.5576	266.0763	374.6673
Smcr8	ENSRNOG00000005165	16.16897	20.47089	15.3728	14.62224	20.10478	14.33223
Cnpy2	ENSRNOG00000003549	101.2472	117.1617	102.4316	98.30336	105.1236	88.91352
Nuak2	ENSRNOG00000000034	15.80329	15.66483	22.99046	14.73223	13.82649	22.51035
Sdf4	ENSRNOG00000019981	200.908	201.2696	187.1754	173.6124	165.5295	179.2473

9-Sep	ENSRNOG00000002807	91.72058	92.9234	105.0744	89.31987	85.94218	94.80694
Kdelr3	ENSRNOG00000013577	144.8414	152.4437	214.65	123.8266	112.4803	203.2924
Cfl1	ENSRNOG00000020660	692.3315	695.2944	813.2441	637.5343	504.9793	714.6177
Hnrnpf	ENSRNOG00000014562	260.8593	204.9917	253.7938	239.4576	161.9361	240.9196
Dctd	ENSRNOG00000013215	13.34142	5.683823	9.457077	11.62324	5.241103	7.414377
Cct3	ENSRNOG00000019090	124.6006	96.05909	124.1388	114.1037	71.04977	115.3577
N4bp2l2	ENSRNOG00000001108	29.6909	25.67968	27.78554	29.18677	23.86781	26.81123
Mtmr6	ENSRNOG00000012918	18.57362	25.78588	24.49939	18.10223	24.45464	22.50103
Tapt1	ENSRNOG00000003174	11.87871	10.44277	12.55226	9.767823	9.919381	11.24557
Hdhd2	ENSRNOG00000043171	18.83573	16.96163	23.72399	16.51069	12.52472	22.46378
Ppp1r15a	ENSRNOG00000020938	24.1742	23.99303	14.25341	22.97155	21.16971	13.28009
Nppa	ENSRNOG00000008176	0.126998	0.157073	0.078481	0	0.12565	0
Zbtb49	ENSRNOG00000005633	3.028157	4.044881	3.518079	2.889577	3.488695	3.178056
Adgrg6	ENSRNOG00000011411	15.64411	8.261863	18.28929	14.03671	7.755291	17.53874
Rps15	ENSRNOG00000024603	720.4402	766.1539	738.5036	685.487	613.9273	633.473
Lhx3	ENSRNOG00000018427	0.173705	0.05371	0.107345	0.154622	0	0.026193
Tipinl1	ENSRNOG00000037221	11.15637	5.174372	8.095914	10.28263	3.153702	7.411618
Supv3l1	ENSRNOG00000000392	9.801111	10.29034	9.623613	9.399106	8.533434	8.407689
Rab13	ENSRNOG00000016733	49.73677	50.71068	58.77274	47.25795	45.75868	49.68372
RGD1559786	ENSRNOG00000012724	25.56251	19.38938	22.32212	23.47847	14.2846	20.4955
Haus7	ENSRNOG00000055648	32.77739	21.28849	33.05128	27.71024	14.8565	31.65953
Sec11c	ENSRNOG00000017036	32.87862	31.22772	34.16354	29.24207	24.79757	32.46616
LOC100360316	ENSRNOG00000051615	120.8249	57.67272	163.1023	116.5322	41.90807	154.5103
Mob1a	ENSRNOG00000059474	61.70676	56.64669	65.9309	58.9207	51.04941	64.28133
Ppia	ENSRNOG00000027864	554.4163	524.6546	713.1986	510.9657	354.1469	613.0281
Eci2	ENSRNOG00000029549	49.3674	49.23762	44.76565	45.33878	39.39768	41.2688
Chchd3	ENSRNOG00000013211	61.84125	64.45109	65.70194	54.08322	56.04703	63.90139
Eif3m	ENSRNOG00000012738	150.3411	175.0931	175.6135	139.687	139.279	157.9883
Adcy10	ENSRNOG00000053410	0.045708	0.035333	0.070616	0.024412	0.022611	0.027569
Thoc3	ENSRNOG00000000104	46.31949	31.09256	34.60904	41.11415	20.93784	31.7075
Lbr	ENSRNOG00000052574	41.72127	32.68086	47.84729	37.28872	21.99523	44.11113
Fam32a	ENSRNOG00000039528	47.40674	31.51535	31.85948	46.31832	27.06262	29.12156
Fbxo3	ENSRNOG00000009549	19.54131	17.7134	18.97839	16.51289	16.99661	17.03303
AABR07028349.1	ENSRNOG00000021584	33.03743	22.35059	34.14697	28.82946	19.43927	33.1969
Gorasp2	ENSRNOG00000055853	34.21028	33.74448	40.04046	33.23749	30.56105	35.63298
Nkapd1	ENSRNOG00000009958	7.030247	7.380717	7.567514	6.764401	6.837006	7.405867
Smu1	ENSRNOG00000007671	57.10858	54.18142	61.24646	53.96411	44.67064	57.0877

Supplementary Table 5. The 1551 down-regulated genes under siPde4d.

Symbol	Gene id	NC1A	NC2A	NC3A	Si1A	Si2A	Si3A
Plscr3	ENSRNOG00000027914	18.899	17.44156	20.12305	15.32208	13.88181	16.51773
Vom1r90	ENSRNOG00000058136	0.029176	0.029414	0.054675	0	0	0.02616
RGD1302996	ENSRNOG00000000812	0.299389	0.408365	0.363034	0.113568	0.225523	0.1737
B4galt4	ENSRNOG00000003114	9.657043	9.550296	10.85066	7.746674	7.635503	8.842369
Akr1d1	ENSRNOG00000013004	0.026459	0.026675	0.024792	0	0	0
AABR07072272.1	ENSRNOG00000029560	0.138932	0.140067	0.130179	0	0	0
LOC100911857	ENSRNOG00000057016	0.072576	0.073169	0.068004	0	0	0
Olr1734	ENSRNOG00000058776	0.044385	0.044748	0.041589	0	0	0
Fyco1	ENSRNOG00000006336	7.741594	10.84845	10.11378	5.184005	8.274068	7.354447
Snx13	ENSRNOG00000004186	12.29417	15.92272	15.20389	10.33574	13.82296	13.06835
Tyk2	ENSRNOG00000032948	7.122293	8.733488	7.703378	5.700295	7.19547	6.161123
Utp15	ENSRNOG00000016591	11.70693	11.48358	10.73529	10.64678	10.51022	9.750349
Ddx5	ENSRNOG00000030680	294.2642	324.4571	288.164	273.1875	305.1718	266.9
Mif4gd	ENSRNOG00000003837	13.64854	15.40303	13.83843	9.508344	11.05568	9.954678
RGD1308106	ENSRNOG00000025061	7.883753	7.806861	7.879513	7.117548	7.002889	7.021784
Irak1bp1	ENSRNOG00000008984	5.474893	5.053679	6.928765	4.290397	3.967254	5.865326
Parl	ENSRNOG00000023271	21.77136	21.09034	19.18752	20.49259	19.91411	18.04997
Gltp	ENSRNOG00000001192	17.70183	12.70924	11.56333	16.79656	11.8707	10.61321
Glrx	ENSRNOG00000012183	18.4681	35.31067	23.38191	9.654607	25.63559	14.81459
AABR07040855.1	ENSRNOG00000051669	8.590066	12.34982	9.572899	7.398406	11.01878	8.385849
Uros	ENSRNOG00000017810	5.140485	4.258039	4.113648	4.7868	3.863236	3.762086
Ciita	ENSRNOG00000002659	1.103017	1.504038	0.914556	0.752967	1.103281	0.53364
Rtn4	ENSRNOG00000004621	53.72901	52.61662	45.12581	39.01718	39.2846	32.17738
Med4	ENSRNOG00000017170	14.86373	13.14956	11.29069	13.20507	11.65783	9.824917
Gdpd2	ENSRNOG00000002800	0.132662	0.074303	0.082869	0.067896	0.013483	0.026433
Ankdd1a	ENSRNOG00000015554	0.910625	1.350096	1.079113	0.271409	0.710451	0.360223
Heatr3	ENSRNOG00000015459	9.380681	8.558483	7.675817	8.094275	7.363107	6.302547
Tuba4a	ENSRNOG00000003597	24.69258	19.13541	17.6429	21.66184	16.0895	14.95901
Prdm15	ENSRNOG00000001620	2.371884	2.411357	2.533712	2.129981	2.187777	2.323321
Cxcl12	ENSRNOG00000013589	96.73504	93.96181	106.391	67.25882	67.18704	80.68032
Snapc5	ENSRNOG00000010156	23.91433	25.40196	22.71646	19.23053	20.96982	17.60067
Ube2i	ENSRNOG00000017907	117.8956	110.9965	99.89375	102.6088	96.92858	83.58102
Dbndd2	ENSRNOG00000014571	32.81328	27.4287	23.69409	28.53017	23.74967	19.73824
Cyp2s1	ENSRNOG00000020743	0.515453	0.637007	0.545297	0.229768	0.380226	0.298179
Shkbp1	ENSRNOG00000020882	5.888255	6.04112	6.766769	3.254686	3.786003	4.301371
Bst2	ENSRNOG00000059900	78.48166	201.2071	171.472	59.15033	178.9644	151.7573
Zbtb17	ENSRNOG00000010436	11.72719	14.06968	13.28373	9.784318	12.12906	11.06778
Faap20	ENSRNOG00000036876	9.422287	10.99549	10.60731	5.023681	6.345488	5.47755
Tmem222	ENSRNOG00000008564	21.19079	20.8066	18.58952	18.5598	18.17514	16.30194
Ypel4	ENSRNOG00000060130	4.784611	5.793684	7.35823	3.737014	4.602408	6.132026
Arhgdia	ENSRNOG00000036688	142.6972	128.9135	113.1163	129.0561	115.0657	97.32848
RGD1311946	ENSRNOG00000025245	16.79731	12.92919	11.99714	15.66394	11.5986	10.79754
Anp32a	ENSRNOG00000058484	35.33628	30.83011	28.6145	28.28698	23.04887	20.27478
Cnot2	ENSRNOG00000004909	39.20535	40.74809	37.3454	36.65524	37.77097	34.73039
Pip5k1a	ENSRNOG00000021068	21.33365	20.21213	20.82785	16.27524	15.94064	16.32659
Agpat1	ENSRNOG00000000437	13.30501	12.83171	12.33795	10.39571	9.466952	9.452674
Arrb2	ENSRNOG00000019308	3.922571	4.187242	2.95476	2.999538	3.353442	2.183848
Kxd1	ENSRNOG00000019971	25.53795	29.00659	24.30768	18.61809	20.63692	16.60547
Adamts16	ENSRNOG00000016812	0.011947	0.012045	0.022389	0.011006	0.010928	0.021425
Csnk1a1	ENSRNOG00000017106	139.177	161.1197	159.4848	117.8513	142.7218	137.2474
Tulp3	ENSRNOG00000005767	25.18702	27.8611	33.83736	12.0386	15.03234	18.52252
Rmdn3	ENSRNOG00000011690	20.98582	33.22132	25.27913	15.55437	27.045	20.19144
Atxn7l3b	ENSRNOG00000059744	30.98935	35.07096	36.2232	20.92106	24.30286	23.9684

Atp5md	ENSRNOG00000042869	53.99404	50.99715	46.86434	38.74585	33.40186	32.36051
Cluap1	ENSRNOG00000007117	19.24184	21.69307	19.16801	17.2582	20.04543	17.19363
Rundc3b	ENSRNOG00000008463	0.023334	0.0941	0.05466	0	0.074703	0.031384
Ddx52	ENSRNOG00000002612	19.2205	19.34913	18.43172	17.27856	17.41341	16.12287
Slc25a22	ENSRNOG00000049944	6.422027	6.529054	6.642817	4.952331	5.049157	5.418591
Ttc1	ENSRNOG00000003980	31.77816	36.74199	29.8903	26.52188	30.43415	24.5833
Zc3h4	ENSRNOG00000015229	5.008859	4.973958	4.911736	3.595864	3.226359	3.344766
Mk1	ENSRNOG00000019657	22.91876	25.19689	31.33935	20.67644	22.90888	29.46963
Smurf1	ENSRNOG00000000999	13.74728	11.31725	10.62892	12.3848	10.19849	9.475448
Eloc	ENSRNOG00000031730	17.69977	14.79028	12.35366	14.6207	11.51937	9.720389
Zmynd15	ENSRNOG00000019477	4.970717	8.367536	5.298486	2.688709	6.534983	3.271161
Zhx3	ENSRNOG00000027988	5.878872	4.616615	5.645052	5.124901	3.710825	4.715807
RT1-A1	ENSRNOG00000038999	85.56713	123.7141	123.2296	71.66414	106.2918	107.3202
Tm4sf19	ENSRNOG00000001757	1.885549	2.205106	2.137769	1.094333	1.224536	1.183465
Camk2b	ENSRNOG00000052080	0.17655	0.198932	0.107041	0.10524	0.123492	0.018624
Cuta	ENSRNOG00000000481	24.08779	21.74285	22.44177	20.90305	19.15035	19.26387
Naa50	ENSRNOG00000039017	24.20009	21.25685	17.03444	20.8674	18.43939	13.49912
Chchd6	ENSRNOG00000060248	16.52299	20.13692	17.42197	12.97762	15.819	13.03157
Bap1	ENSRNOG00000019097	20.61591	20.2315	20.90217	15.02603	15.58095	16.33008
Rab24	ENSRNOG00000016539	13.54396	9.730107	9.002051	12.16712	8.493292	7.446538
AABR07048308.1	ENSRNOG00000027547	0.890862	0.561338	0.417367	0.615521	0.305574	0.199696
Tomm20	ENSRNOG00000019980	173.0895	181.752	164.611	131.1936	128.6278	117.0402
Prpf31	ENSRNOG00000061039	23.35636	20.64737	17.65454	20.2254	18.00029	14.29888
Tgm1	ENSRNOG00000020136	0.764599	1.097476	1.347853	0.238772	0.663812	0.801771
Iqcd	ENSRNOG00000001378	0.700727	0.578006	0.626734	0.264083	0.233073	0.257033
RGD1359508	ENSRNOG00000038330	5.076269	3.954618	4.215937	4.570163	3.552772	3.792999
Klhl7	ENSRNOG00000010453	8.990139	10.63986	10.66191	6.988394	8.170249	8.658049
Klf16	ENSRNOG00000033694	6.973134	7.336499	6.343953	6.050605	6.285613	5.525737
AABR07068214.1	ENSRNOG00000061442	62.77202	145.9563	135.8592	35.83701	123.7299	107.4154
Gckr	ENSRNOG00000048874	0.415642	0.122219	0.129818	0.350996	0.047523	0.046585
Rfx1	ENSRNOG00000006049	2.843159	2.951317	2.890964	2.3476	2.330924	2.266043
Cacnb2	ENSRNOG00000018378	0.364697	0.464433	0.44963	0.229876	0.333585	0.344213
Med27	ENSRNOG00000013933	19.4699	22.80062	20.89618	14.61961	18.70423	17.11278
Hikeshi	ENSRNOG00000017383	17.69706	17.64277	12.83262	13.55137	13.12002	9.348946
Wdr4	ENSRNOG00000001181	6.390527	7.432986	6.374212	5.876018	6.765988	5.751765
Jsrp1	ENSRNOG00000032951	0.076199	0.076821	0.095197	0	0	0
Msantd4	ENSRNOG00000022245	19.58828	24.61384	22.03616	17.42603	21.79865	19.51967
Polrmt	ENSRNOG00000024879	5.246956	4.465129	5.35436	4.801393	4.008369	4.998031
Ccdc58	ENSRNOG00000031653	14.82779	16.32772	12.61213	9.813917	10.30384	7.906147
Tle4	ENSRNOG00000013239	0.866426	1.013998	1.021893	0.580497	0.775887	0.798605
Mt-nd2	ENSRNOG00000031033	8.6769	4.671149	7.143542	8.187497	4.12246	6.722593
Rrp12	ENSRNOG00000048495	11.0439	11.79265	10.31984	8.609522	9.145831	8.28957
Med10	ENSRNOG00000017150	33.76048	37.81811	31.50325	26.40846	31.30408	25.86953
Bcl2l12	ENSRNOG00000020486	17.22609	13.8726	13.59606	10.57954	7.901952	8.511342
Chchd3	ENSRNOG00000013211	60.20403	67.79516	62.19107	55.03327	61.13662	56.64275
Nop9	ENSRNOG00000020321	19.83191	16.98792	16.52383	10.96916	10.02999	9.339579
Pcsk6	ENSRNOG00000011526	0.474774	0.159551	0.563491	0.40822	0.096505	0.482463
Zfp746	ENSRNOG00000007064	14.15706	13.26861	13.06513	12.16817	11.13819	11.43933
Dohh	ENSRNOG00000004259	67.3687	80.38747	62.74425	60.72843	74.20564	54.74425
Phrf1	ENSRNOG00000017299	12.09321	11.46131	11.19394	10.61556	9.743126	9.886746
Elmod2	ENSRNOG00000028176	2.036796	2.488173	2.69077	1.614356	2.156766	2.352773
Xylb	ENSRNOG00000014168	4.293591	3.846257	5.852849	3.919672	3.536951	5.566001
Ets2	ENSRNOG00000001647	11.04383	15.83428	12.45847	9.002634	13.14773	9.913226
Bhlhe40	ENSRNOG00000007152	31.73597	44.69678	43.0428	28.06553	41.93404	39.80182
Lin37	ENSRNOG00000020929	23.98666	35.6329	28.96737	18.58282	29.20042	21.78269

Crocc	ENSRNOG00000008334	2.75235	2.216186	2.698759	2.266303	1.576257	2.16758
Peg12	ENSRNOG00000024429	4.285605	4.032576	4.047088	2.957172	2.951539	2.606979
Fam222b	ENSRNOG00000029570	1.551442	1.716184	1.726262	1.081859	1.320545	1.371768
Syf2	ENSRNOG00000060597	44.64706	59.74007	50.79223	37.99123	54.51848	43.9084
Camk2a	ENSRNOG00000018712	0.433589	0.466273	0.56878	0.026629	0.1322	0.259183
Mtfmt	ENSRNOG00000014602	9.763124	11.46774	9.844996	8.794279	10.74466	8.976358
Aatf	ENSRNOG00000002778	15.70499	18.34887	15.63236	13.43136	15.3274	12.74378
Prpf19	ENSRNOG00000020897	50.47097	55.18272	44.71122	29.15995	29.65344	25.5229
RGD1561157	ENSRNOG00000042258	21.04421	48.34325	36.10124	15.81801	42.04133	31.35469
Sap130	ENSRNOG00000016773	3.748442	3.779066	3.442822	2.916688	2.721631	2.335619
Plrg1	ENSRNOG00000006655	56.85337	56.50079	51.59154	52.23967	50.45339	45.58217
Rmt1	ENSRNOG00000013373	0.059461	0.07993	0.055715	0.00913	0.01813	0.008886
Pigz	ENSRNOG00000047384	0.208027	0.209726	0.19492	0.071866	0.09514	0.093263
Zfp770	ENSRNOG00000043037	4.731689	4.660683	3.788071	3.99158	4.112469	3.153529
Sdhaf3	ENSRNOG00000011283	6.607444	7.007473	5.306697	5.138394	5.023403	3.616263
Rars	ENSRNOG00000007739	84.93713	87.16462	73.94231	73.43724	78.68792	63.66182
Zbtb2	ENSRNOG00000019544	4.407041	4.218081	4.024837	3.340447	3.431528	3.05119
Nrbf2	ENSRNOG00000000641	6.746264	7.504105	7.230917	5.320511	5.578732	5.602569
Cwc15	ENSRNOG00000008490	35.22514	38.98239	30.51136	27.24663	31.06205	24.48152
Gabpb2	ENSRNOG00000021105	10.35332	11.39771	9.896149	7.893399	8.762569	7.949429
Ccl7	ENSRNOG00000000239	889.0187	1280.343	919.12	560.3537	1016.015	671.3324
Zcchc6	ENSRNOG00000016629	16.50322	24.33398	25.16722	15.50344	23.13962	23.79879
RGD1562747	ENSRNOG00000037352	4.538552	5.565344	4.359252	3.276709	4.606862	3.367828
Ppm1b	ENSRNOG00000030667	9.189285	10.23389	9.475644	9.042063	10.032	9.300239
Sfxn1	ENSRNOG00000018279	43.3554	56.17287	49.7758	33.11819	42.71049	39.29259
Ift74	ENSRNOG00000008075	10.25579	12.05587	9.415108	8.9125	10.21645	7.706587
Sf3b2	ENSRNOG00000020412	54.40585	53.29022	52.17364	47.64465	48.33677	45.78847
Dnaaf4	ENSRNOG00000056654	0.4609	0.50892	0.452427	0.323504	0.321206	0.275509
Nek7	ENSRNOG00000000657	33.26987	45.72408	46.77921	21.86106	31.0498	31.18553
Agrn	ENSRNOG00000020205	8.636298	16.33029	18.83279	4.944833	11.4065	13.86133
Slc41a1	ENSRNOG00000042320	3.416383	4.563927	5.238224	1.760059	2.350163	2.995765
Slc9a1	ENSRNOG00000007982	3.358564	3.457371	3.640746	2.673766	2.510884	2.856277
Nlgn3	ENSRNOG00000003812	0.033054	0.044432	0.123886	0	0	0.079034
Gja1	ENSRNOG00000000805	162.4165	143.8064	173.5326	120.9241	87.60057	117.5452
Spsb2	ENSRNOG00000047113	4.634624	5.63552	5.237668	3.585149	4.30398	3.774936
Wdr62	ENSRNOG00000049708	6.426941	5.522751	3.976956	4.665063	4.229492	2.575798
Nsmce4a	ENSRNOG00000020452	50.57971	33.2678	29.63089	46.83844	28.87208	24.42027
Psmd9	ENSRNOG00000001339	46.33792	42.37994	38.19723	39.0558	37.16881	31.58508
Ik	ENSRNOG00000017251	46.09747	49.87428	39.88128	41.65886	46.52097	36.51864
Crtc2	ENSRNOG00000056337	3.441701	4.065131	3.288092	2.238086	2.82403	2.390119
Ei24	ENSRNOG00000030391	28.06531	26.97755	27.39873	15.34439	17.14728	17.99978
Notch1	ENSRNOG00000019322	1.658477	1.773923	1.915579	0.960726	1.15981	1.421153
Gosr1	ENSRNOG00000003971	16.57416	17.4127	16.61586	14.5412	15.82635	15.12779
Timm9	ENSRNOG00000008222	16.68578	15.69079	15.26879	14.69737	13.96819	13.86753
AABR07066944.1	ENSRNOG00000056226	9.331942	5.731421	2.713652	7.954629	4.022647	1.490747
Gmfb	ENSRNOG00000047250	75.60899	95.64384	75.66957	65.34983	81.10703	63.34873
Evi5	ENSRNOG00000002039	10.81121	10.3917	10.88382	9.329411	9.070588	9.842096
Inip	ENSRNOG00000017047	24.79732	25.24743	18.40395	20.35622	22.06436	14.85958
Daxx	ENSRNOG00000000477	21.57226	38.03587	28.15557	15.37647	32.05904	20.03647
Larp4b	ENSRNOG00000015888	31.89071	30.95782	31.85148	25.91202	26.76225	26.59849
Tmem167a	ENSRNOG00000016686	187.0083	203.0389	180.3518	169.1059	185.8812	156.9612
Ndufb3	ENSRNOG00000011825	62.537	62.48942	53.00251	55.28652	57.14585	47.51885
Rab1a	ENSRNOG00000004992	110.3707	114.0733	116.1437	93.86797	97.58866	94.02343
Spryd4	ENSRNOG00000003127	10.77804	15.22448	13.01215	8.510683	12.07949	10.56674
Arhgef17	ENSRNOG00000053502	30.40261	28.67766	32.28806	24.93738	24.54618	28.27133
Hmgn1	ENSRNOG00000050978	86.32406	80.20645	71.72579	59.43586	56.4	52.99464

Cnksr1	ENSRNOG00000022838	2.219575	5.594271	3.707943	1.372138	4.59473	3.011435
Chmp6	ENSRNOG00000004014	28.6233	30.83948	24.10897	23.41884	25.07671	20.09869
Usp10	ENSRNOG00000016509	16.83112	16.22379	13.16531	14.23085	13.36799	11.1771
Dnttip2	ENSRNOG00000025025	14.19033	13.88805	12.03305	10.68423	10.70319	9.593107
Cited2	ENSRNOG00000056940	18.92904	18.47247	14.70672	17.12785	15.87654	12.362
Inpp5b	ENSRNOG00000048506	4.940494	6.359281	6.416317	3.82103	4.813298	4.831636
Rap2a	ENSRNOG00000051564	12.79304	12.60413	13.32581	8.750708	9.813945	9.857525
Sf3a1	ENSRNOG00000005218	11.23954	10.03912	9.477447	8.554934	7.385536	7.575982
Pwp1	ENSRNOG00000005350	16.13463	15.83725	14.06101	12.45188	12.88912	11.48962
AABR07021591.1	ENSRNOG00000029459	7.344234	10.04136	8.012725	4.634095	7.177837	4.239757
Tbc1d8	ENSRNOG00000013583	1.142652	0.92159	0.996538	0.793539	0.514548	0.717186
Kcnt1	ENSRNOG00000017283	2.31043	5.668732	3.573076	1.708273	5.170488	3.157728
Lsm1	ENSRNOG00000015375	30.51934	37.33605	38.91552	26.05993	33.61092	35.85196
Ppp1r37	ENSRNOG00000017692	23.53058	23.7529	25.37553	18.48819	19.39413	19.09169
Lmo2	ENSRNOG00000009401	3.199302	7.197064	7.091651	1.649617	6.092983	5.523185
Clca2	ENSRNOG00000013771	0.042141	0.031864	0.039486	0	0	0.009446
Tox4	ENSRNOG00000012844	19.52219	20.33232	18.87172	12.53469	15.01595	13.91192
Akna	ENSRNOG00000008005	1.896722	2.744692	3.102475	1.498788	2.378034	2.829583
Prm2	ENSRNOG00000002539	0.277864	1.540738	0.390536	0	1.143721	0
Pcbp3	ENSRNOG00000001245	2.260692	1.69862	1.67862	1.198496	0.975396	0.745795
Ube2z	ENSRNOG00000006868	37.11324	36.02608	33.5976	32.48199	29.49007	26.99832
Hmgb3	ENSRNOG00000050910	6.039159	4.99639	3.4764	4.440817	3.170713	2.233978
Hnrnpul1	ENSRNOG00000020683	41.84762	38.85627	37.21874	38.41165	36.31147	33.49986
Erlin2	ENSRNOG00000013763	16.90737	26.20444	20.8707	11.5744	21.73635	14.33408
Kat2a	ENSRNOG00000018364	9.499517	10.08194	10.24152	7.433338	7.99558	7.388825
Asrgl1	ENSRNOG00000020202	4.445995	4.563324	5.094418	2.96083	3.552256	3.722305
Fbxo28	ENSRNOG00000000066	24.70289	28.52989	24.37916	16.93999	22.94319	18.66339
Mt-nd4	ENSRNOG00000029707	7.971452	5.667228	7.409701	7.314338	4.706023	6.634972
Snx5	ENSRNOG00000006077	64.94558	53.47186	48.79651	63.65986	52.23995	47.9125
Mettl16	ENSRNOG00000002764	9.845646	11.0654	8.906214	9.084434	10.0111	8.134
Dusp16	ENSRNOG00000006628	4.043675	5.058827	4.477798	3.149472	4.303966	3.872941
Gnai3	ENSRNOG00000019465	59.26092	53.36346	47.07799	56.71726	50.29985	45.00158
LOC108348180	ENSRNOG00000002100	20.31614	20.35282	22.04026	18.52891	19.02292	20.07532
Tsr2	ENSRNOG00000002556	23.20935	24.52969	20.13855	15.6248	14.2367	12.76541
Cers2	ENSRNOG00000021138	45.33642	53.81503	46.00484	39.80875	49.96736	40.50518
Wwtr1	ENSRNOG00000016617	61.43377	73.74952	68.69239	57.20702	68.46703	62.42234
Dnaja1	ENSRNOG00000007029	119.1946	117.7842	108.654	98.93851	103.6808	93.20181
Hmgxb4	ENSRNOG00000013878	7.091869	5.36506	6.192665	6.098397	4.651713	5.146742
Arap3	ENSRNOG00000055527	0.066194	0.143003	0.088605	0.026134	0.103795	0.033916
Ensa	ENSRNOG00000048617	39.41941	41.46678	36.73024	35.72172	36.07003	32.49916
Pno1	ENSRNOG00000005524	19.72688	18.83823	15.69243	16.81414	16.16555	11.85249
Yae1d1	ENSRNOG00000013438	12.46746	19.36806	14.64425	8.948812	14.79708	11.49891
Hsd17b12	ENSRNOG00000009630	58.83229	54.54732	46.51678	56.16115	50.60619	43.37924
Rrp1b	ENSRNOG00000001194	5.585995	4.819955	4.246848	4.58747	3.436619	3.252944
Camlg	ENSRNOG00000021911	18.25836	17.30054	18.22502	13.90129	12.51125	15.02102
Scamp4	ENSRNOG00000018271	30.06368	30.48834	30.59426	26.27008	27.17416	25.72799
Mllt1	ENSRNOG00000048736	12.59629	10.76646	12.12226	8.657078	8.100719	9.005971
Pdcd2l	ENSRNOG00000021119	8.314765	9.982445	8.712723	7.513687	9.405221	7.853752
Ccer2	ENSRNOG00000046971	0.530468	0.213921	0.347932	0.390949	0.048522	0.23782
NEWGENE_1304700	ENSRNOG00000057755	0.834095	0.957998	1.177262	0.641954	0.811228	0.956157
Zfp7	ENSRNOG00000050695	3.862253	3.411964	3.449467	3.53425	3.001087	3.173507
Atp6v1b2	ENSRNOG00000011891	25.19429	37.34451	35.44817	17.58191	32.17164	29.57115
Exoc3l2	ENSRNOG00000017448	0.273238	0.382597	0.28447	0.167811	0.291583	0.14972
Hars2	ENSRNOG00000016087	11.77507	13.3909	11.64883	9.58959	10.27565	8.409462
Rrp9	ENSRNOG00000012927	27.47243	29.55257	21.82884	21.51233	20.53026	14.11469

Lgals12	ENSRNOG00000021207	0.031338	0.031594	0.044045	0	0	0
Fst	ENSRNOG00000011631	25.35573	17.99466	25.47928	22.65971	13.91562	22.09057
Slc29a1	ENSRNOG00000019752	40.45287	45.16418	38.48582	32.76798	38.72429	28.83188
Atf3	ENSRNOG00000003745	1.624774	3.564271	1.381443	0.443497	2.32558	0.55306
Eral1	ENSRNOG00000010673	12.20559	12.43552	11.1542	11.00626	11.36127	9.57363
Hspe1	ENSRNOG00000051624	317.4909	260.5956	246.8057	290.3703	219.4917	213.1028
Sirpa	ENSRNOG00000004763	6.053221	7.340739	11.07893	4.24491	5.218743	8.370355
Tra2b	ENSRNOG00000001783	80.15972	72.38623	56.44566	73.64288	64.78685	51.46334
Txnl4b	ENSRNOG00000021379	6.035471	7.172273	7.050965	4.755659	5.779014	5.181378
Prodh2	ENSRNOG00000057578	0.061294	0.092691	0.086148	0	0	0
LOC690000	ENSRNOG00000014966	35.22833	62.64652	46.03233	26.59395	55.23014	40.3777
Pdcd10	ENSRNOG00000010147	30.93755	35.41769	31.56362	29.83171	34.06761	29.88835
Fam220a	ENSRNOG00000024605	5.649719	5.016752	5.456647	5.384893	4.80999	5.2801
Abraxas2	ENSRNOG00000017222	19.98652	22.66506	18.55939	18.20915	21.37048	16.58598
Rab21	ENSRNOG00000003923	30.66107	35.61227	32.90458	24.46727	31.20362	28.52397
Ndufb11	ENSRNOG00000008329	45.72238	43.54948	42.02059	39.60432	36.35256	32.8158
Prkrip1	ENSRNOG00000001426	8.791361	11.1358	8.267642	7.73304	9.514179	6.881731
AABR07043288.1	ENSRNOG00000057732	5.096964	3.898252	4.281782	2.428713	1.768404	2.521465
Ssr3	ENSRNOG00000011148	153.5237	198.3292	158.0227	120.8079	176.7754	127.4888
Znhit6	ENSRNOG00000030049	4.485698	4.211434	3.625156	3.538359	3.282429	2.991413
Smap2	ENSRNOG00000011421	12.57376	10.80547	9.287751	11.05328	8.816655	7.958997
Bccip	ENSRNOG00000018066	10.59441	10.06521	6.999465	7.336199	6.875834	4.823419
Tgfbr3l	ENSRNOG00000050573	1.125909	0.78964	1.10084	0.58626	0.358212	0.43893
Kcnn4	ENSRNOG00000019440	40.78062	35.42204	16.86398	38.14034	31.35031	13.26559
RGD1564541	ENSRNOG00000012564	12.43428	11.71062	12.07088	11.05094	9.573031	10.25788
Ogfod2	ENSRNOG00000001081	8.007806	8.71694	9.198373	6.421251	7.665366	7.657226
Stard8	ENSRNOG00000033883	1.717603	1.763264	1.28604	1.314987	1.198038	0.893104
Parp2	ENSRNOG00000008892	13.29247	9.928367	9.434345	11.04537	7.392064	7.800545
Gmcl1	ENSRNOG00000017838	9.548214	9.956588	9.444259	7.286765	8.299584	6.869333
Prpf38a	ENSRNOG00000009451	46.11562	50.11585	36.17766	33.23201	39.23254	27.89
Pnrc2	ENSRNOG00000009248	80.05634	95.65066	83.52355	71.64476	89.80666	74.52729
Zfp422	ENSRNOG00000013379	8.778483	7.922507	8.570282	8.239648	7.103769	7.772154
Nup85	ENSRNOG00000003673	36.01004	16.41951	12.43296	30.43077	12.21014	8.761876
Btg2	ENSRNOG00000003300	15.88608	14.1904	12.66041	7.663622	8.218544	7.137385
Mdp1	ENSRNOG00000019840	10.92358	9.313167	8.482569	10.29723	8.36516	7.537451
AABR07001512.1	ENSRNOG00000017412	20.65248	9.832237	7.295128	15.93035	5.4723	4.262055
Lin54	ENSRNOG00000002203	10.98404	7.870366	5.440873	9.763526	6.713493	4.651309
Sft2d2	ENSRNOG00000003038	19.57291	24.08303	21.83903	14.31965	20.4652	18.15326
Prkx	ENSRNOG00000060168	6.094736	5.984515	5.393478	3.041293	3.300366	3.45345
LOC103693323	ENSRNOG00000003349	0.080065	0.129151	0.43512	0.029504	0.073235	0.358949
Yif1b	ENSRNOG00000055286	2.538288	2.007511	2.439875	1.935198	1.541164	1.706953
Med12	ENSRNOG00000003848	4.94907	5.65595	6.539301	4.117351	4.451967	5.701672
Abcf1	ENSRNOG00000000799	26.61219	26.09405	22.53721	23.01761	20.65513	18.53163
LOC100360117	ENSRNOG00000048456	91.37204	92.80258	99.17802	85.68586	85.54267	90.24256
Actn4	ENSRNOG00000020433	136.8243	88.72929	79.53329	121.9715	73.59299	69.66975
Carns1	ENSRNOG00000018603	7.976539	6.433365	5.835456	7.206969	5.261599	4.965212
Oasl	ENSRNOG00000001187	35.57782	122.6497	57.76396	16.98932	101.0516	44.10964
Eif1b	ENSRNOG00000018848	18.41678	18.13656	16.94512	14.77985	15.32611	12.51263
Lsm10	ENSRNOG00000025716	12.60739	15.53492	16.15811	8.187925	12.50393	13.0801
Chd3	ENSRNOG00000009722	19.20641	15.30643	21.9486	15.38754	12.82823	18.20328
Ipo13	ENSRNOG00000019758	16.41747	16.25515	14.93538	14.15371	13.24629	12.98489
Msmp	ENSRNOG00000043023	19.97719	19.28699	21.73254	16.53216	15.7953	16.698
RGD1563941	ENSRNOG00000005918	5.452513	6.885789	5.700548	3.806947	5.853603	4.322866
Tmem254	ENSRNOG00000011276	19.45753	19.77623	15.05445	13.54592	12.55112	10.51333
Tomm6	ENSRNOG00000046316	431.9952	404.9339	351.7429	392.236	367.0329	295.2615

Gfer	ENSRNOG00000013370	26.32235	29.95346	22.65874	19.09043	24.85986	17.81324
Mtx3	ENSRNOG00000023837	7.953557	9.514817	9.484935	6.590893	8.388947	8.632867
Slc30a3	ENSRNOG00000006204	0.111699	0.112611	0.083729	0	0.040868	0
Prr13	ENSRNOG00000042094	57.93825	84.33004	69.89251	42.30267	73.8982	53.448
Ccdc94	ENSRNOG00000047456	14.79959	17.20447	12.10152	11.76146	15.10575	10.04263
Zfp827	ENSRNOG00000011697	1.436891	1.048532	1.307893	0.995939	0.638381	1.030702
Mthfr	ENSRNOG00000008553	4.494483	5.068556	5.62858	2.72051	3.96613	4.068695
Serpinb5	ENSRNOG00000002640	0.849119	1.592264	1.066132	0.344185	1.165025	0.395905
LOC108348142	ENSRNOG00000032635	585.4432	570.711	566.2023	552.4638	530.9781	541.5351
Rnf213	ENSRNOG00000029658	58.89385	139.3003	128.8701	34.28392	120.4206	113.3414
Nfx1	ENSRNOG00000009015	26.05065	23.83219	24.60203	20.3047	19.26274	21.0198
Atad1	ENSRNOG00000010861	26.04846	39.61762	37.26078	17.69827	30.67953	31.68016
Ltv1	ENSRNOG00000015217	17.14402	19.7132	17.06244	14.72656	16.63512	15.14356
Hipk4	ENSRNOG00000020835	0.365001	0.367983	0.410405	0.201751	0.267091	0.278184
Ube2n	ENSRNOG00000058053	48.56081	41.5694	33.8364	43.92262	38.01124	30.9278
Anapc2	ENSRNOG00000011295	33.37625	32.71332	33.2566	28.27264	28.35476	26.39378
Pcgf3	ENSRNOG00000000062	19.15036	18.87846	17.17601	16.21612	15.01828	14.74913
Hdgf	ENSRNOG00000042261	138.5927	164.0469	104.2374	110.7278	137.312	86.73947
Tfpt	ENSRNOG00000056098	14.32055	16.28555	14.16972	10.80035	14.11188	11.36805
Kbtbd6	ENSRNOG00000011657	0.457687	0.483399	0.510537	0.30117	0.318966	0.273588
AABR07002848.1	ENSRNOG00000050453	7.883654	8.824226	6.456311	6.404912	6.870439	5.232041
Celf1	ENSRNOG00000010379	42.16462	45.02652	33.39201	35.39308	37.28543	28.65446
Lrtomt	ENSRNOG00000023434	0.698621	0.402473	0.56109	0.505682	0.091289	0.268463
Snx15	ENSRNOG00000021007	8.779241	9.278807	8.052146	7.892286	7.836224	6.730307
Rnf26	ENSRNOG00000007720	11.16479	9.249586	8.447409	8.873692	6.735421	6.905865
Rabggta	ENSRNOG00000030483	8.468829	8.650571	7.980088	7.111242	6.798139	5.748762
Pebp1	ENSRNOG00000001136	181.2835	182.1607	165.4469	156.0062	152.524	125.2038
Fgfr1op	ENSRNOG00000055093	13.13454	15.17094	11.44898	11.69711	13.98933	10.57601
Sacm1l	ENSRNOG00000005149	36.47827	51.67433	41.56305	35.4258	49.94328	40.15529
Ric8b	ENSRNOG00000007323	8.479712	8.486436	9.263237	7.564132	7.926594	8.419233
Ikbkb	ENSRNOG00000019073	13.93431	14.42111	15.08995	11.82576	12.48619	12.085
Ciao1	ENSRNOG00000012638	31.62634	37.92207	28.03057	24.30819	33.35446	20.83068
AABR07048397.1	ENSRNOG00000038905	6.617716	6.711495	7.086597	4.898961	4.071483	5.262651
AABR07030494.1	ENSRNOG00000049929	4.318007	2.353127	3.936605	3.332841	1.280968	2.406739
Gpam	ENSRNOG00000015124	10.17324	10.73373	13.32369	6.728251	8.229169	9.173225
Zfp672	ENSRNOG00000002713	9.903366	8.352956	9.110951	9.247567	7.674405	8.114051
RGD1559575	ENSRNOG00000032396	0.111398	0.16044	0.149114	0.014661	0.101895	0.071346
Hnrnpa3	ENSRNOG00000052968	17.73577	19.46748	17.69745	14.07546	13.5541	13.28663
Fbxo39	ENSRNOG00000014953	0.90206	2.204679	1.152582	0.528825	1.800236	0.906865
Cep250	ENSRNOG00000019340	7.067628	5.814956	5.263073	6.142112	4.840571	4.667005
Igf2bp2	ENSRNOG00000025946	3.595343	3.318575	4.028928	2.416984	2.599799	3.125719
Parp16	ENSRNOG00000029127	10.27076	11.91925	12.37327	9.097886	9.962395	10.7019
Rasgrp2	ENSRNOG00000021098	4.620145	3.607388	4.881702	3.296328	2.805354	3.649031
Smn1	ENSRNOG00000018067	12.19041	11.95203	10.16591	9.433418	8.697379	8.225161
Tuft1	ENSRNOG00000020923	13.86684	13.88926	12.1728	7.567523	9.139003	8.335245
Arl16	ENSRNOG00000049235	11.82284	9.804695	7.906443	10.62813	8.110703	6.839269
Eif2b2	ENSRNOG00000006467	53.9437	56.02799	45.75868	50.27898	53.84863	42.58612
Adgra2	ENSRNOG00000012991	33.90014	32.52709	40.73026	24.81719	25.35948	35.29769
Tmx1	ENSRNOG00000057934	80.71368	81.75739	77.50346	73.50049	70.77748	70.21387
Pfdn1	ENSRNOG00000018653	104.6348	118.1644	101.2856	100.2785	110.8474	94.67615
Nxph3	ENSRNOG00000005185	1.578977	0.926615	0.574132	1.324348	0.53891	0.316965
Sfxn2	ENSRNOG00000059312	0.646585	0.594686	0.68025	0.438906	0.45654	0.447531
Tmed10	ENSRNOG00000007901	220.0431	227.0557	236.5674	127.9097	172.1985	152.4757
Mrps18c	ENSRNOG00000002178	46.79296	50.9929	41.66524	39.76844	46.75968	34.97189

Smyd5	ENSRNOG00000015589	16.05197	16.16299	14.29234	14.34625	13.75127	11.40332
Clec2e	ENSRNOG00000052128	0.044794	0.09032	0.167887	0	0.020486	0.120492
Zfp687	ENSRNOG00000021026	7.146899	6.462242	6.68615	5.812426	5.495349	5.867522
LOC100911730	ENSRNOG00000019215	31.9105	33.25954	33.84486	23.84787	26.73949	23.05634
Fhl3	ENSRNOG00000007541	9.103245	10.6281	7.622808	7.374112	8.111329	6.027949
Bak1	ENSRNOG00000000485	17.04317	21.47257	17.62906	12.63626	18.61229	14.70059
Phb2	ENSRNOG00000012999	121.5728	128.5168	107.7408	111.8471	112.3353	95.64459
Mta2	ENSRNOG00000019913	49.82722	51.44512	46.24874	44.67482	43.51347	41.00008
Hdac3	ENSRNOG00000019618	34.03835	31.00735	35.35654	30.19268	28.28804	32.98366
Fam110c	ENSRNOG00000005660	3.812257	6.594768	3.321629	1.801354	4.503563	2.068598
Triobp	ENSRNOG00000059015	9.198142	13.07605	10.40989	7.400376	12.02262	8.95734
LOC100361898	ENSRNOG00000022466	7.891795	9.567249	6.600119	6.218735	7.367713	5.29247
Tbc1d5	ENSRNOG00000010637	11.06604	11.15645	12.94358	9.272138	10.05384	11.11602
Vta1	ENSRNOG00000011540	33.29838	35.23296	32.3296	29.65317	32.28526	30.19797
Katnb1	ENSRNOG00000014626	14.61145	13.99963	12.6135	11.92877	12.42653	10.48417
LOC103690128	ENSRNOG00000054319	1.553337	0.612793	0.632813	0.995472	0.185325	0.30278
Dennd2a	ENSRNOG00000026748	3.790947	4.682619	5.371006	2.640337	4.006743	4.319532
Gch1	ENSRNOG00000011039	2.657708	6.847409	4.676157	1.387414	4.861973	2.515404
Tulp2	ENSRNOG00000020927	0.832271	1.118761	1.104766	0.404657	0.401783	0.414583
Aldh1a2	ENSRNOG00000055049	1.570603	1.192463	0.926593	1.375446	0.904538	0.591126
Tnfrsf9	ENSRNOG00000036942	6.726957	13.92484	8.939421	5.278156	11.44056	6.926109
Prkab1	ENSRNOG00000001142	14.33231	14.70704	14.7463	10.31949	11.1812	12.41177
Nme4	ENSRNOG00000050424	4.213946	5.111324	5.583355	3.376568	4.025107	4.162149
Trim62	ENSRNOG00000049496	1.562985	1.792035	2.325987	0.903454	1.121295	1.923544
LOC100909712	ENSRNOG00000011586	27.49712	31.38846	32.20586	23.01178	28.78888	28.50745
Mdm2	ENSRNOG00000006304	90.94761	80.74317	95.47609	79.55999	73.65292	87.69471
Hdgfl3	ENSRNOG00000019740	19.48803	29.70936	23.85437	16.02288	24.60546	20.66555
Pcbd1	ENSRNOG00000000566	0.942166	0.712397	0.529683	0.867958	0.646344	0.422393
Slc30a4	ENSRNOG00000000170	19.31651	18.2995	17.16684	17.40742	17.06911	15.91961
Psmb10	ENSRNOG00000019494	93.63475	281.8259	143.9749	74.48052	253.0426	110.8263
Tada2a	ENSRNOG00000002757	13.16449	11.92803	11.37871	12.1468	10.47912	10.49645
LOC103689941	ENSRNOG00000049397	9.853038	14.84232	12.91438	8.105709	12.64205	9.951877
Parp10	ENSRNOG00000004361	20.73735	37.41373	32.81362	14.60564	33.70748	26.58435
Mrpl22	ENSRNOG00000027039	46.91205	53.16755	43.80889	39.01142	48.42988	38.15814
Ppwd1	ENSRNOG00000012505	6.458385	5.953564	6.335665	5.653861	5.483148	5.726877
Eif6	ENSRNOG00000049497	171.9689	217.6738	145.8482	146.9237	175.8865	116.0673
Eaf1	ENSRNOG00000019559	10.15409	12.44629	11.28877	8.266031	10.51691	10.14766
Trim25	ENSRNOG00000002341	29.30164	56.99659	45.79289	23.74588	47.28604	37.74037
Epc2	ENSRNOG00000029447	8.928437	10.22231	8.822028	7.536125	9.372247	7.867223
Otud4	ENSRNOG00000018477	15.29213	15.93519	13.44048	10.40531	9.621048	9.799836
Spr	ENSRNOG00000015455	27.25146	20.67873	16.95249	23.7978	17.28646	11.62461
Arnt	ENSRNOG00000031174	15.35219	17.50036	19.58835	12.32768	14.96014	15.32348
Tp53i11	ENSRNOG00000008738	3.941538	4.967175	4.543228	2.478363	3.619604	3.702445
Polr3c	ENSRNOG00000033560	10.73272	13.824	11.09684	8.983875	11.0189	9.241797
LOC100361920	ENSRNOG00000052174	15.56039	14.87045	12.75751	13.43888	12.75037	9.44674
Wdpcp	ENSRNOG00000054331	3.169087	2.65403	2.576641	2.687775	2.208565	2.300294
Ppp4c	ENSRNOG00000019813	82.26286	76.31676	80.25943	66.49323	65.38273	60.99308
Plekhg6	ENSRNOG00000019528	2.74222	2.463712	4.404768	2.423122	2.03052	3.847089
Ak2	ENSRNOG00000000122	62.98321	63.49777	45.28945	54.72055	49.91308	36.04985
Tmc1	ENSRNOG00000051262	0.05324	0.035784	0.033257	0.032698	0	0
Dnajc9	ENSRNOG00000006619	53.63708	26.661	20.92821	47.07704	20.84502	17.20352
Pcgf2	ENSRNOG00000012705	2.188179	3.211759	2.954866	1.274717	2.119248	2.337106
Lamtor2	ENSRNOG00000019908	23.62459	24.07025	22.58442	13.70472	16.42053	16.91357
AABR07062570.1	ENSRNOG00000056041	18.74072	19.03085	18.08352	11.37992	14.73853	12.74159
LOC103694876	ENSRNOG00000061740	12.56297	14.79839	12.22548	10.04922	11.29668	10.17302
Kank3	ENSRNOG00000007230	3.294034	2.977399	3.086495	2.780457	2.211534	2.61893

Simc1	ENSRNOG00000016932	6.548483	5.78107	4.749681	6.117225	5.035251	4.030975
Ppp1r12a	ENSRNOG00000004925	25.11001	23.61877	21.99416	23.40581	21.68653	20.90359
Clic1	ENSRNOG00000029682	197.1229	205.5096	140.1282	179.808	176.3107	110.355
Rab34	ENSRNOG00000023375	90.45245	84.43938	97.70102	56.20216	65.16797	67.88538
Cct7	ENSRNOG00000015630	111.8559	107.0017	84.73425	95.13168	91.07445	75.13429
6-Sep	ENSRNOG00000043182	0.842575	1.115707	0.954457	0.718285	0.931738	0.732937
Dstyk	ENSRNOG00000021298	6.263189	7.627745	8.296498	5.169813	5.931862	7.233206
1700092M07Rik	ENSRNOG00000048966	0.686488	0.692096	0.482427	0.474313	0.313963	0.153884
Mrps34	ENSRNOG00000015479	32.5434	34.47422	32.268	30.24866	30.70022	29.74598
Pisd	ENSRNOG00000018319	45.13406	52.44181	52.70608	37.37074	48.08087	46.50399
Pawr	ENSRNOG00000005917	31.15901	29.11451	30.08941	21.60024	23.76025	21.78571
Oxnad1	ENSRNOG00000019760	9.176747	6.04552	5.010892	6.282165	4.211425	3.177812
Sp2	ENSRNOG00000010492	1.174861	0.847966	1.038292	0.72565	0.573954	0.74219
Ckap4	ENSRNOG00000008016	51.32088	38.25101	28.79131	39.98435	24.09984	20.88299
Pdss1	ENSRNOG00000048849	6.117817	4.610582	4.199953	5.440653	4.210794	3.503114
Psmb5	ENSRNOG00000013386	55.19325	53.06665	48.61571	46.68971	45.07415	35.59945
Banf1	ENSRNOG00000020460	71.46715	71.70406	51.86249	63.93594	61.85539	38.62316
Nudt21	ENSRNOG00000042983	30.53274	29.10056	26.99316	21.66889	23.60957	21.26784
Slco3a1	ENSRNOG00000032798	5.068664	4.52844	3.359273	3.834256	2.764173	2.327815
Psma6	ENSRNOG00000007114	141.605	196.0387	138.1893	121.707	163.6542	117.0945
Rad9a	ENSRNOG00000018729	17.26045	11.90036	10.81676	14.70411	8.250509	8.686785
Rprd2	ENSRNOG00000054294	5.323096	5.505856	5.522734	4.343879	4.254056	4.822462
Npr1	ENSRNOG00000014684	1.290961	1.995646	2.278128	0.693747	1.348118	1.292576
Fam216a	ENSRNOG00000031731	28.52764	31.39799	26.79652	20.77707	24.5743	22.50454
Tnfaip3	ENSRNOG00000049517	28.65665	48.86708	36.24319	19.77081	33.46496	25.35774
LOC100910581	ENSRNOG00000050881	1.103284	0.593225	0.895936	0.914747	0.437305	0.626526
Egr2	ENSRNOG00000000640	1.651456	2.169892	1.52204	0.473873	0.730524	0.728246
Rrp1	ENSRNOG00000001203	36.13068	38.18685	35.15466	33.24083	35.21524	30.48686
Maea	ENSRNOG00000005397	79.20192	73.28212	74.80311	57.00584	59.5799	60.63083
Pja1	ENSRNOG00000047339	20.85748	22.56942	20.44751	16.9308	20.40891	17.18427
Acin1	ENSRNOG00000013533	19.66615	24.70968	23.3856	18.30274	22.29299	21.06498
Rbm19	ENSRNOG00000001397	6.195172	7.971594	5.833492	4.496313	5.359113	4.248368
Frmd8	ENSRNOG00000020881	32.58333	29.07747	29.57809	26.52946	25.61343	23.56267
Dnttip1	ENSRNOG00000014933	13.16079	16.35892	14.73354	11.80771	14.0686	12.32183
Cdkn2aip	ENSRNOG00000022736	21.55172	26.77804	21.59471	19.01357	23.69137	19.8987
Cnst	ENSRNOG00000002710	8.76939	9.025922	7.873209	5.928465	6.511587	6.308346
RGD1304622	ENSRNOG00000000040	9.218798	8.266233	8.373056	7.758662	5.963786	6.952325
Lsg1	ENSRNOG00000001727	13.14565	16.94652	13.5445	12.04917	16.34568	12.66392
LOC361646	ENSRNOG00000016635	2.218344	2.47722	3.073706	1.858363	2.213045	2.597613
Znf48	ENSRNOG00000017837	10.06825	11.04554	11.0787	7.93693	9.763024	8.75625
Rbm43	ENSRNOG00000004673	9.953618	13.29455	10.86288	8.595208	11.80839	10.04303
Cd151	ENSRNOG00000046094	612.477	577.0141	499.4177	420.3291	450.7425	384.8035
Dnajc5	ENSRNOG00000015202	48.30204	46.93998	44.46146	40.31663	39.73373	40.07598
Cish	ENSRNOG00000029543	2.87246	3.383223	2.575025	2.277273	2.374783	1.906913
Cnbp	ENSRNOG00000010239	326.3612	369.9511	287.9431	280.8169	304.7857	250.7604
Zbtb21	ENSRNOG00000001623	8.302036	9.078738	8.604496	6.313619	7.865021	7.35281
Tbc1d2b	ENSRNOG00000014543	20.8924	26.35978	37.11839	15.06398	20.05722	27.3568
Cib1	ENSRNOG00000033498	78.31641	75.29203	78.65372	62.42427	59.33949	52.94539
AABR07036855.1	ENSRNOG00000056398	110.0756	113.4656	104.3897	96.88997	101.4028	84.0713
Nup54	ENSRNOG00000002247	19.18708	17.57003	14.64635	15.93556	15.09393	12.86976
Rangrf	ENSRNOG00000004980	19.08448	31.52262	28.70931	15.31743	24.58249	23.3004
Wdhd1	ENSRNOG00000011167	18.99018	8.300843	6.576573	16.031	6.125625	4.94344
Stam2	ENSRNOG00000027447	11.87636	11.64535	10.80145	6.979937	8.418469	7.939753
Rfesd	ENSRNOG00000012810	8.003458	8.927232	8.137436	6.274962	7.476466	5.598487
Rab8b	ENSRNOG00000018009	14.41298	20.64841	16.2133	11.42654	16.47026	13.89077
Akap2	ENSRNOG00000011504	80.86527	64.07495	63.46746	74.82129	53.6549	56.55791

Sgca	ENSRNOG00000003998	3.82576	2.788518	5.183312	2.381372	2.009788	4.056145
Pip4p1	ENSRNOG00000009948	11.39677	16.16693	14.89996	7.855814	11.11134	8.343373
9-Sep	ENSRNOG00000002807	86.65242	78.98227	82.63938	83.07454	73.44762	75.94302
Tfe3	ENSRNOG00000009605	21.83097	25.03272	24.76571	20.34562	23.52532	22.33188
Cxcl1	ENSRNOG00000002802	554.0395	608.9287	595.9198	383.0951	517.5833	457.9165
Tymp	ENSRNOG00000032394	18.11228	15.22001	15.10483	17.26876	14.26854	13.65316
Cdk5	ENSRNOG00000008017	20.72359	24.41675	21.41397	16.97643	22.30419	18.84128
Xpo6	ENSRNOG00000016722	19.12472	18.16008	19.50761	14.46311	15.18869	16.74607
LOC100911295	ENSRNOG00000048226	102.9426	95.90088	72.60302	75.13709	66.90915	56.78381
Tars2	ENSRNOG00000057194	16.19773	16.40497	17.35283	11.89306	14.10234	13.49094
Inhbb	ENSRNOG00000060237	16.73933	11.13284	19.39221	12.73225	8.059953	13.80141
Ddx49	ENSRNOG00000022368	65.5027	53.66719	49.51383	62.27928	49.18951	43.54348
Ccl2	ENSRNOG00000007159	1984.351	2130.707	1329.783	1322.434	1715.835	933.4023
Hist1h1c	ENSRNOG00000054978	1722.395	1556.254	1068.795	1434.805	1403.627	823.985
Rad18	ENSRNOG00000005907	10.05835	5.830356	3.557093	7.419437	3.780724	2.147321
Usp37	ENSRNOG00000022720	8.968042	12.69594	11.76744	7.380655	9.83732	9.7767
Nono	ENSRNOG00000003689	99.02367	85.85811	78.99585	93.48156	82.77087	73.38397
Zmat2	ENSRNOG00000016016	30.43742	25.42497	23.50412	24.63565	22.04327	19.76073
Rab6b	ENSRNOG00000009198	1.473615	1.971529	2.561808	1.075793	1.220744	1.903013
LOC103694903	ENSRNOG00000005389	20.6583	20.58986	18.91581	17.60059	17.64774	17.25729
Fam110a	ENSRNOG00000050946	5.6414	5.323643	5.001202	4.199649	4.482554	3.423308
Plekhh1	ENSRNOG00000010650	0.361683	0.405153	0.517756	0.249896	0.220552	0.306283
Ssr1	ENSRNOG00000014165	71.23445	52.79618	45.7233	55.31128	41.66323	36.94732
Helz2	ENSRNOG00000013267	19.86836	35.82278	34.14911	12.78696	29.68154	30.41474
Polg	ENSRNOG00000032293	22.0764	18.38744	19.53067	17.51901	15.64423	16.73141
Mipol1	ENSRNOG00000009151	4.810335	5.534909	4.678735	3.781189	4.184763	3.961536
Mnat1	ENSRNOG00000007267	26.80982	26.85929	21.71663	24.91512	25.23044	20.72105
Gprasp2	ENSRNOG00000037658	5.357578	6.964098	8.813252	4.020572	4.68029	7.367677
Mical2	ENSRNOG00000016244	37.08967	31.57898	29.8408	30.86205	25.77391	26.52178
Znrd1	ENSRNOG00000000779	21.11038	26.10089	21.47277	18.71967	22.95802	19.80573
Mad1l1	ENSRNOG00000001265	13.11902	9.020169	8.304874	10.77374	7.785362	6.459928
Wac	ENSRNOG00000018698	44.55518	44.91919	39.33214	40.19162	41.64387	37.00959
Ccdc106	ENSRNOG00000015997	8.194947	12.9768	11.53805	6.758965	10.83171	8.809833
Nln	ENSRNOG00000011561	21.53571	27.2416	25.61211	15.2195	23.8079	21.14831
MGC94199	ENSRNOG00000049287	7.557115	7.459655	8.285808	7.232055	6.912439	7.666018
Samd4b	ENSRNOG00000019831	11.3897	10.62808	11.75614	7.120645	7.653914	9.425542
Anp32e	ENSRNOG00000021168	20.56461	18.47998	17.57597	15.36174	10.10533	12.63901
Rassf1	ENSRNOG00000021548	22.28453	26.18846	18.99371	17.82918	18.72581	14.42422
Fblim1	ENSRNOG00000011774	19.9256	12.21432	14.59546	14.06861	8.820224	10.88922
Pank1	ENSRNOG00000018944	3.710426	2.401261	2.626468	3.134577	2.074874	2.25185
Wbp11l1	ENSRNOG00000005505	18.51793	13.39751	14.15248	17.3202	12.76459	13.03531
Msl3	ENSRNOG00000004016	26.55472	26.22089	24.35149	22.75562	24.02172	20.15374
Mapk9	ENSRNOG00000002823	18.29318	21.98335	19.08843	17.05393	20.3754	18.24674
Tpm3	ENSRNOG00000017441	146.8218	135.5921	90.97134	117.2118	117.9435	72.91866
Uba1	ENSRNOG00000019164	47.43283	53.23443	47.98488	39.4324	42.36405	42.23616
Rap1b	ENSRNOG00000007048	154.9468	199.3003	223.7244	109.1818	125.5002	135.2553
Ash2l	ENSRNOG00000014875	63.43282	59.21664	50.60208	42.08038	47.13425	36.81107
Med20	ENSRNOG00000013852	29.5706	28.18975	32.14743	23.26751	24.30846	24.63041
Comp	ENSRNOG00000048472	1.075004	1.225151	1.021873	0.674575	0.983299	0.782292
Mnt	ENSRNOG00000002894	2.051119	2.782936	3.484547	1.916055	2.559972	3.351669
Gps1	ENSRNOG00000046698	73.65814	65.77672	61.46983	62.09151	58.98176	54.38668
B3gnt7	ENSRNOG00000018267	0.311362	0.924277	0.664529	0.207161	0.806934	0.48081
Vwa5b2	ENSRNOG00000001707	0.727937	1.056793	0.67298	0.59013	0.816763	0.52216
Rapgef3	ENSRNOG00000059961	4.313083	3.671336	7.901184	2.962184	2.964766	6.900921
Bicd2	ENSRNOG00000016031	34.12909	26.47407	24.52077	32.90113	24.78545	23.63231
Hist1h2ba	ENSRNOG00000016865	4.283245	0.750998	1.134217	3.774323	0.425854	0.50094
Nemp2	ENSRNOG00000012924	4.076281	2.690639	2.824159	3.547276	2.392584	2.250127

Atp10a	ENSRNOG00000056228	28.71503	23.41934	21.45229	26.54824	20.17217	19.67403
Oscp1	ENSRNOG00000026978	6.471098	6.720809	6.167932	4.548148	5.663935	4.151619
AABR07061825.1	ENSRNOG00000011606	3.120787	3.073113	2.85616	2.808124	2.887754	2.66808
Abcb8	ENSRNOG00000008557	11.79031	12.03676	11.4914	11.42284	11.41599	11.11788
Osbpl2	ENSRNOG00000057756	12.30599	11.26981	10.91187	11.03996	9.65028	10.08087
Stau1	ENSRNOG00000007781	16.07845	15.76472	15.93281	11.87327	12.93525	13.65047
Arid5a	ENSRNOG00000015382	1.934572	2.097884	1.78222	0.943517	1.442397	1.239014
Dhps	ENSRNOG00000004219	25.84441	26.2318	21.62249	23.91619	24.91896	20.58679
Ap4b1	ENSRNOG00000019455	10.32287	11.50986	10.02345	8.529845	10.37415	9.01412
Rhbdl3	ENSRNOG00000005515	0.688547	0.388266	0.677971	0.559057	0.245519	0.449955
AABR07027567.1	ENSRNOG00000018381	7.73821	8.584182	7.687165	6.69957	7.338484	7.054437
Nme1	ENSRNOG00000002693	144.9669	105.567	80.174	120.0734	88.54516	66.8578
Tas1r2	ENSRNOG00000061876	7.470554	7.913156	7.163553	6.147267	7.152823	6.348618
Gipc1	ENSRNOG00000003864	67.36582	61.22213	49.2932	55.72016	47.57895	42.37374
Dusp3	ENSRNOG00000036798	9.742493	9.23511	8.055783	7.929235	6.470521	6.542951
Gemin5	ENSRNOG00000002645	8.236823	8.524502	9.233586	6.822814	6.830336	6.648507
Dnajb12	ENSRNOG00000030408	15.35771	20.68873	17.33981	14.21585	18.62247	15.11576
LOC688583	ENSRNOG00000011096	2.276052	1.888001	1.187806	1.300536	1.264945	0.645825
Arih1	ENSRNOG00000009887	18.44885	19.39362	21.81067	17.09829	16.83588	20.03466
Mef2a	ENSRNOG00000047756	28.68202	33.65567	31.07452	21.57023	27.29549	27.46128
Rassf8	ENSRNOG00000015986	46.17681	36.96544	40.66203	41.65513	32.12502	38.17712
Depdc5	ENSRNOG00000018144	3.645647	3.483947	3.247877	2.575987	2.538386	2.705902
Plaur	ENSRNOG00000037931	50.25341	54.10757	31.20479	40.2773	44.32617	25.99431
Stk11ip	ENSRNOG00000020107	3.971647	5.116872	4.950103	2.49426	4.331792	3.941794
Psmc4	ENSRNOG00000018994	109.1465	104.3401	91.15726	94.23337	96.81274	78.25907
Zfp804a	ENSRNOG00000038004	0.036188	0.060806	0.022605	0.022225	0.033101	0
Gnl3l	ENSRNOG00000002509	26.24471	27.4753	28.28796	21.54229	25.03317	23.7301
Ap1b1	ENSRNOG00000008786	25.49743	22.03827	23.19684	17.98867	18.2351	17.33989
Map4k1	ENSRNOG00000020505	0.234448	0.12606	0.205031	0.100792	0.014297	0
RGD1305455	ENSRNOG00000039214	1.660207	2.331081	2.015361	1.492291	2.047316	1.681469
Anapc5	ENSRNOG00000001316	230.9775	191.6086	173.2613	200.7712	172.362	156.558
Hipk1	ENSRNOG00000019333	17.859	19.09193	21.74421	13.28593	15.01885	19.43367
AABR07066792.1	ENSRNOG00000054698	2.902297	2.117506	2.647876	2.392261	1.67666	1.84902
Actn3	ENSRNOG00000019745	0.091269	0.061343	0.171038	0	0.013914	0.081836
LOC497963	ENSRNOG00000057443	1.179082	4.002006	1.865876	0.609487	2.870003	0.875139
Polr1b	ENSRNOG00000018349	8.890024	9.384978	7.261418	7.857509	8.589314	6.740026
Atic	ENSRNOG00000015511	82.36505	79.27238	68.67399	70.42426	66.73319	62.24106
Lrrc45	ENSRNOG00000045926	15.61443	15.07749	13.56583	12.38498	13.19096	11.64676
Mamdc4	ENSRNOG00000016584	1.07643	1.186963	1.281775	0.929669	0.923065	1.115981
Rbm28	ENSRNOG00000005468	5.743628	4.639085	4.548252	4.562811	3.998002	3.781249
Clcf1	ENSRNOG00000018752	11.94434	12.3547	10.50139	10.59272	11.54616	8.884269
Rnh1	ENSRNOG00000016416	249.824	281.1927	213.6575	184.1047	247.448	166.1375
Nucks1	ENSRNOG00000047287	80.89173	64.77737	51.49518	77.0169	57.24232	44.23618
Tmem170a	ENSRNOG00000049019	1.769098	1.685673	1.526239	1.530382	1.381374	1.373459
Cyb5d1	ENSRNOG00000045742	8.02651	8.477422	5.551071	7.100891	7.633133	5.083533
Alkbh2	ENSRNOG00000028584	4.050845	4.521504	4.168409	3.498551	4.036107	3.8916
Fastk	ENSRNOG00000011667	48.27866	34.35909	37.92254	44.42597	32.41728	34.9724
Mum1	ENSRNOG00000024549	16.48591	11.77292	11.97499	15.70367	10.31759	11.02165
Lamb2	ENSRNOG00000047768	25.64864	24.0416	32.78003	23.45769	19.92695	28.50274
Cnnm4	ENSRNOG00000015886	1.750918	1.906818	1.965214	1.440496	1.370312	1.645507
RGD1562136	ENSRNOG00000032398	18.29094	17.72926	15.57638	16.51062	14.44315	12.05957
Pak4	ENSRNOG00000019883	7.717827	7.604042	6.872047	6.453489	6.969194	5.720906
Ccdc189	ENSRNOG00000053015	2.674605	2.281616	2.753949	2.518097	2.070066	2.450872
Noc4l	ENSRNOG00000037478	8.847423	8.26643	7.752899	5.579124	6.542529	5.564257

Ddx27	ENSRNOG00000008081	18.01071	17.35267	14.40184	14.70018	12.83651	12.11554
Bud13	ENSRNOG00000018665	9.149464	9.550735	8.345407	7.776149	6.832174	6.352501
Higd1b	ENSRNOG00000002814	0.063795	0.321583	0.239104	0	0.233413	0.114403
Pdgfrb	ENSRNOG00000018461	205.0545	184.3981	241.4463	184.057	164.6593	230.8828
Celsr2	ENSRNOG00000020058	0.178035	0.087658	0.096987	0.068657	0.011361	0.040837
Agk	ENSRNOG00000011509	12.53793	12.71246	9.603871	11.04535	10.16018	8.121632
Dmap1	ENSRNOG00000019407	14.91432	18.61889	16.67519	14.35829	18.04432	16.38358
Ccdc86	ENSRNOG00000020925	33.3344	36.39092	23.34954	18.76654	25.43867	16.08066
Plekhn1	ENSRNOG00000020295	2.578005	3.465422	2.44486	2.029506	2.944031	1.527024
AABR07005579.1	ENSRNOG00000046147	8.734615	6.182433	6.572881	6.04541	4.84337	4.544896
Tmx2	ENSRNOG00000005308	54.36471	53.05497	35.14825	48.8307	43.73491	29.83238
Bbox1	ENSRNOG00000059519	0.037357	0.018831	0.052505	0	0	0.016748
Atg2a	ENSRNOG00000060707	3.734401	4.26597	5.5559	3.440268	3.750468	4.957647
Nol6	ENSRNOG00000010409	4.193116	5.375754	4.756483	2.50173	2.144208	2.612566
Prom1	ENSRNOG00000003098	0.14757	0.092984	0.155556	0.110457	0.042182	0.082698
Lama5	ENSRNOG00000053691	24.61726	28.17843	29.25036	18.98468	18.66239	23.834
Uqcrb	ENSRNOG00000024967	22.54101	22.93956	20.24412	20.29545	18.36178	16.24526
Ciapin1	ENSRNOG00000016234	37.42721	38.75794	32.45501	30.68943	29.41174	27.77137
Hmgcl	ENSRNOG00000009422	15.27597	24.05256	21.77423	12.93175	19.31378	18.21871
Hadha	ENSRNOG00000024629	54.31851	65.598	62.84479	52.88481	63.26354	59.91201
Zbtb39	ENSRNOG00000004306	3.394218	3.141212	3.279093	2.398892	2.32678	2.793721
Slc38a7	ENSRNOG00000012007	5.69724	7.293326	7.114111	4.21584	6.553671	6.040955
Map1s	ENSRNOG00000018781	12.2352	11.79342	11.28036	9.681703	8.403043	9.608547
Naa20	ENSRNOG00000010523	43.62955	59.25609	39.75036	41.4897	55.17185	35.45449
Ftsj3	ENSRNOG00000009857	39.63726	38.58472	32.00877	34.39561	34.68446	29.41991
Sdad1	ENSRNOG00000022229	10.63999	12.8626	9.473402	8.175686	9.805722	7.9862
Agbl5	ENSRNOG00000008612	2.293521	2.366032	2.223986	1.805772	1.866127	1.972776
Myo10	ENSRNOG00000010161	8.605485	7.223427	6.558867	5.887304	5.073439	5.235075
Psmc3ip	ENSRNOG00000020022	23.89722	13.49758	13.35402	19.89307	10.3587	11.40206
Kif13a	ENSRNOG00000001455	11.25777	8.839953	10.09779	10.17946	6.944204	8.986863
Slc35a1	ENSRNOG00000008908	22.27108	20.6353	22.25014	19.95069	17.95364	17.9484
Anks3	ENSRNOG00000003186	5.345924	5.399543	5.341816	3.898092	4.37563	4.616506
Rpl7l1	ENSRNOG00000016269	97.92177	112.7143	86.21837	71.54706	91.35107	73.4498
Sympk	ENSRNOG00000014353	21.25176	21.2439	18.96033	17.5489	18.71242	17.0804
Wdr33	ENSRNOG00000011382	14.93281	16.56614	16.00378	12.8835	13.88895	14.69885
Coq5	ENSRNOG00000001171	16.47802	16.44283	12.88844	10.44767	12.6587	9.665347
Pear1	ENSRNOG00000014243	12.14028	11.44565	12.17321	10.10446	10.45141	10.631
Eftud2	ENSRNOG00000002818	8.184168	7.854585	6.210051	7.19239	5.957328	4.980331
Wrap53	ENSRNOG00000010520	10.18656	9.247672	9.001934	7.44957	7.838244	6.168498
Mgp	ENSRNOG00000005695	479.1336	545.0526	580.2148	387.3617	398.1707	391.184
Cycs	ENSRNOG00000010452	78.8474	70.55704	49.13921	69.74777	65.31602	43.91644
Nin	ENSRNOG00000005540	2.846783	3.165583	3.03366	2.478531	2.508594	2.646004
Mtmr2	ENSRNOG00000005923	45.88523	45.8712	38.85568	42.09348	39.92439	35.7296
Irf9	ENSRNOG00000019478	78.70873	110.7998	110.214	55.73181	99.02351	86.3876
Pkp1	ENSRNOG00000010076	44.50486	40.56653	23.81126	23.97986	22.68815	13.94927
Aaas	ENSRNOG00000013445	11.42754	9.396808	7.166982	10.3376	7.122074	5.256681
Pde4d	ENSRNOG00000042536	0.494833	0.572239	0.586389	0.294967	0.239622	0.404539
Elp1	ENSRNOG00000016725	44.88694	44.95863	44.88049	40.62101	42.88832	40.94162
Jrk	ENSRNOG00000027238	8.620657	8.802036	8.833712	7.722003	7.314831	8.025678
Prelp	ENSRNOG00000003120	124.4949	126.5688	142.5172	110.2269	119.5727	128.7735
Tsn	ENSRNOG00000002319	58.70118	45.66808	49.53443	45.20972	38.56675	41.07473
Nufip1	ENSRNOG00000001033	12.7358	10.9035	11.37636	12.1329	9.662473	10.47638
Pak3	ENSRNOG00000004676	1.938157	1.680433	1.61628	1.428401	1.223245	1.372889
Filip1l	ENSRNOG00000001645	5.48427	3.522556	3.988738	5.093056	3.104923	3.787215
Colca2	ENSRNOG00000042165	1.701917	2.757571	2.448987	1.063911	1.83472	1.144509
LOC100912393	ENSRNOG00000045895	1.901431	2.550801	2.356353	1.342004	1.977668	2.076134

Abr	ENSRNOG00000056837	20.1193	21.12694	17.34021	13.19578	14.79616	14.03544
Xrcc1	ENSRNOG00000019915	17.05895	12.90936	15.7079	15.38551	11.48124	14.91811
Naca	ENSRNOG00000002632	44.2644	48.81418	47.68426	41.65934	43.95927	42.18218
Babam1	ENSRNOG00000017071	46.39721	45.13729	40.36653	37.9204	40.62505	30.84416
Zc3h12a	ENSRNOG00000009131	29.60116	37.68744	38.71536	24.96061	31.81603	29.61636
Zwint	ENSRNOG00000048682	65.32271	59.68762	45.12708	50.22514	51.55179	36.11137
Pigh	ENSRNOG00000010914	7.127741	8.358813	8.218562	6.158068	7.786474	7.003649
RGD1311847	ENSRNOG00000023700	7.9501	11.27476	10.87086	7.376489	10.15978	10.16394
LOC103693015	ENSRNOG00000018291	15.98896	14.87322	12.89652	12.83147	13.11726	11.08483
Cox5a	ENSRNOG00000018816	144.1281	166.8514	127.1195	136.0014	155.4508	110.5269
Cndp2	ENSRNOG00000015591	54.64161	74.50723	66.71379	42.88469	66.73625	50.49746
Mylk	ENSRNOG00000002215	21.75623	19.19842	21.8025	16.9696	16.64931	18.92503
Mybbp1a	ENSRNOG00000015236	45.86997	41.98723	37.17393	37.94285	35.82146	33.44594
Coil	ENSRNOG00000000244	4.433456	5.739471	4.15413	3.527318	5.314837	3.43315
Prkd1	ENSRNOG00000004165	1.474741	1.669378	1.878799	0.965311	0.662635	1.229522
Cd274	ENSRNOG00000016112	6.200405	13.27498	8.661814	4.237297	11.23982	7.682285
Upp1	ENSRNOG00000004972	16.74956	38.08099	25.45288	13.68945	33.05736	18.94625
Stip1	ENSRNOG00000021164	100.5875	85.00825	70.26305	88.75032	79.43174	59.56084
Phf23	ENSRNOG00000017657	22.20645	27.17966	25.52855	19.23713	21.21484	19.32905
LOC500956	ENSRNOG00000020580	8.725366	30.11267	19.2217	6.400265	26.59509	14.34724
AABR07037173.1	ENSRNOG00000027850	0.237846	0.799296	1.040015	0	0.290074	0.639788
Lzts2	ENSRNOG00000014969	20.39437	23.80071	22.44838	18.62684	20.0806	18.94261
Sox6	ENSRNOG00000020514	0.028271	0.028502	0.02649	0	0	0.012675
Cttn	ENSRNOG00000047280	75.35178	79.02027	74.05386	70.40073	74.00366	71.63074
Mesp2	ENSRNOG00000014925	0.14209	0.0955	0.088758	0.043633	0.021661	0.042468
RGD1562218	ENSRNOG00000019169	10.81013	10.78858	10.22094	7.669797	8.821406	8.618015
Pih1d1	ENSRNOG00000020634	16.4753	17.43524	13.58354	12.69518	15.06984	11.65281
LOC100911361	ENSRNOG00000027006	8.82627	8.280436	8.117026	6.098314	4.522558	6.338536
AABR07014804.1	ENSRNOG00000024829	14.95485	16.92697	14.27054	14.53765	16.02888	13.57369
Vgll4	ENSRNOG00000007822	30.59699	34.83609	45.83245	21.80806	28.26766	32.48106
Gtf2h4	ENSRNOG00000000831	6.356086	5.006979	5.720816	5.708549	3.917588	5.147609
Srpk1	ENSRNOG00000000511	24.29199	20.88481	16.48023	22.93575	17.98437	14.36074
Dcaf5	ENSRNOG00000004556	14.94347	15.45885	15.00904	13.69313	13.93813	14.31268
Adgb	ENSRNOG00000042741	0.25549	0.835556	0.554691	0.17222	0.723885	0.502866
Asb5	ENSRNOG00000042630	0.300394	0.359632	0.387018	0.190254	0.188903	0.151507
AABR07035428.1	ENSRNOG00000001107	5.341402	6.606856	7.570393	3.762886	5.173139	6.841899
Popdc2	ENSRNOG00000058752	0.491947	0.813384	0.811276	0.326304	0.44998	0.511677
Pla2g16	ENSRNOG00000021206	28.36828	56.76593	43.93172	17.45566	33.97401	20.94747
Nfya	ENSRNOG00000012702	7.945659	7.141502	8.229354	6.307026	4.502403	6.900379
Hspa1l	ENSRNOG00000047966	0.655362	0.403771	0.494668	0.586973	0.266424	0.408073
Pqbp1	ENSRNOG00000007766	32.70327	30.52545	26.2702	29.48429	27.82959	24.80936
Dctn5	ENSRNOG00000018048	33.2805	37.48557	31.68103	21.58859	32.14072	22.70179
Max	ENSRNOG00000008049	12.18956	17.3024	14.82329	9.972741	12.45788	10.43646
LOC690468	ENSRNOG00000048701	618.6487	551.8248	494.0007	608.5351	547.08	486.9114
Cdipt	ENSRNOG00000024144	58.42192	61.97799	55.62153	43.62766	54.70585	39.83007
Clca4l	ENSRNOG00000036877	0.121355	0.061173	0.099495	0.027949	0	0.054406
Pml	ENSRNOG00000008400	38.62688	50.86511	45.15315	18.48737	39.49934	34.30924
Acp2	ENSRNOG00000013594	26.30106	28.15966	35.33778	18.02391	23.46871	24.94837
Hnrnpa2b1	ENSRNOG00000011175	98.49889	103.2807	82.24362	78.21048	63.22148	57.95774
Greb1	ENSRNOG00000024651	0.417064	0.289785	0.29045	0.228452	0.159812	0.202139
Dcp1a	ENSRNOG00000016015	5.500798	6.708431	6.462331	4.606217	5.253623	5.735017
Rpp25	ENSRNOG00000018812	6.940062	7.156505	7.690537	4.262294	5.565393	4.176919
RGD1304624	ENSRNOG00000006986	4.805723	4.408142	3.949301	4.173189	3.567052	3.5673
AC128303.1	ENSRNOG00000054663	0.612078	0.514232	0.286757	0	0.093311	0

Neurl4	ENSRNOG00000016109	7.479442	8.339658	7.016106	6.020306	6.124088	5.957873
Trnau1ap	ENSRNOG00000055344	21.4288	16.63974	16.90449	13.77152	11.99826	13.05067
Plk3	ENSRNOG00000018484	3.883583	3.34279	3.123962	2.261377	2.396118	2.283132
Scarb1	ENSRNOG00000000981	15.32246	14.76536	12.35717	11.19018	12.6027	9.973448
Cep44	ENSRNOG00000010566	13.1683	12.76745	11.26229	11.51166	11.58366	10.50093
Tyro3	ENSRNOG00000058586	8.15135	6.394366	6.988459	6.816822	5.339515	4.844482
Nyx	ENSRNOG00000009646	0.022204	0.04477	0.062414	0	0.02031	0.019909
Hnrnpm	ENSRNOG00000007921	56.43305	64.08644	51.99748	49.96317	54.17792	47.26754
Jun	ENSRNOG00000026293	16.13685	29.99494	20.60271	9.345001	15.93953	11.64887
Epb41l2	ENSRNOG00000012346	23.80996	18.46725	17.2867	17.50732	11.67772	14.22396
Ccdc112	ENSRNOG00000003559	4.060946	4.829475	3.287892	2.433526	3.263727	2.545317
Rgs3	ENSRNOG00000024501	15.85422	11.86145	9.424035	11.14174	9.608119	6.384363
Myadm	ENSRNOG00000053450	22.25033	13.14345	9.899064	17.56956	8.23078	7.66907
Dab2ip	ENSRNOG00000055226	10.03951	7.269999	7.629231	7.268732	4.751631	6.390438
Ppm1n	ENSRNOG00000058133	2.464435	5.3298	4.767308	1.977382	4.981057	4.027388
Ccl4	ENSRNOG00000011406	1.317763	1.608219	0.974793	0.19168	0.888151	0.435312
Gosr2	ENSRNOG00000003506	41.50578	38.46239	36.30066	37.07031	35.2243	34.30216
Sdhc	ENSRNOG00000003163	86.99348	92.12399	87.12052	54.82078	77.48014	64.32949
Hgs	ENSRNOG00000036696	33.98009	37.32632	33.36106	27.91061	34.57902	29.00021
Klhl23	ENSRNOG00000007981	2.937375	2.428991	2.69819	2.219543	2.045286	2.300855
Hnrnpf	ENSRNOG00000014562	234.581	202.8331	162.924	213.5269	190.1522	152.4451
Hsh2d	ENSRNOG00000023614	3.760645	10.18466	7.686522	1.409554	8.127455	3.652952
Zfp46	ENSRNOG00000049975	8.284065	8.292929	11.05102	6.93292	7.58627	10.28739
Dtx3l	ENSRNOG00000023400	25.33822	54.83972	48.92579	20.91645	52.87996	45.53439
Prkag3	ENSRNOG00000017248	0.271328	0.209181	0.478556	0.13233	0.145989	0.34346
Pdk4	ENSRNOG00000009565	1.140691	1.346354	0.860272	0.768912	0.534415	0.299354
Zfp511	ENSRNOG00000018272	12.23228	14.6106	12.43634	11.5332	13.45279	10.87149
Rbm38	ENSRNOG00000006420	2.642917	2.011443	1.820884	1.623168	1.113926	1.370739
Tmem59	ENSRNOG00000009778	90.60912	108.8676	125.4116	75.98238	84.94681	92.74502
Ror2	ENSRNOG00000053232	1.443244	1.186413	1.830824	0.961381	0.974861	1.433436
Epas1	ENSRNOG00000021318	4.181576	7.278455	12.41575	2.732773	4.895491	9.173162
Ythdf3	ENSRNOG00000010084	27.87161	32.00111	29.55418	26.86156	29.8153	27.34529
Gdpd3	ENSRNOG00000019685	0.530468	0.356534	0.289944	0.244343	0.161738	0.158547
Akr1a1	ENSRNOG00000016727	488.5944	437.4083	415.6592	236.9447	300.8498	279.8808
Ripk3	ENSRNOG00000020465	79.5483	124.2973	61.18172	47.04278	86.63889	44.68926
Xrn2	ENSRNOG00000011785	35.16786	40.00869	37.73881	33.84416	38.91208	35.52511
Clpp	ENSRNOG00000047052	27.67953	31.24573	27.83822	23.48111	28.73966	22.18347
Hdac1	ENSRNOG00000009568	50.05597	44.49429	45.96377	47.41717	39.37303	39.92359
Nrp1	ENSRNOG00000010744	36.0936	30.54357	40.58052	29.11516	24.15001	37.49816
Idh3a	ENSRNOG00000010277	20.74323	18.58109	16.06913	11.73443	12.80642	11.81995
Sf3b4	ENSRNOG00000021181	16.32927	13.98752	11.95683	12.07132	9.426561	9.931662
LOC100911456	ENSRNOG00000024139	16.63016	18.9966	14.87617	15.40991	18.41391	13.5597
Nsrp1	ENSRNOG00000022502	4.742467	5.700676	5.041858	3.600771	5.148278	3.785013
RGD1304587	ENSRNOG00000002944	15.11833	14.5094	15.23554	11.1548	12.75272	11.50842
Trappc3l	ENSRNOG00000031290	0.10026	0.151619	0.046972	0	0.045854	0
LOC100910554	ENSRNOG00000051758	11.5812	5.83791	3.860644	10.87421	4.278042	2.795747
Als2cl	ENSRNOG00000033921	8.480248	10.46314	9.624073	5.654764	8.778931	8.235111
Ccr1	ENSRNOG00000006715	1.137948	2.987617	0.821903	0.45864	2.168486	0.467653
AABR07005779.1	ENSRNOG00000018518	4.247099	3.803087	5.363693	3.548058	2.364656	4.494067
Mrpl20	ENSRNOG00000018647	54.89188	50.68989	42.30294	35.27041	39.23913	32.5193
Dchs1	ENSRNOG00000031643	0.049246	0.037237	0.030762	0.018903	0	0.014719
Dnajc28	ENSRNOG00000002026	5.148658	5.628493	4.446466	3.857367	4.936402	3.754388
Fgfr1	ENSRNOG00000016050	31.67663	30.33409	40.6554	27.55188	26.30492	33.04569
Lsm7	ENSRNOG00000019552	60.26461	59.49119	54.28293	54.27874	54.71347	44.38657
Qrsl1	ENSRNOG00000026049	4.153687	4.921535	4.313285	3.392593	3.662258	3.705175
LOC103694328	ENSRNOG00000038766	11.95216	11.36705	11.54213	9.341453	8.705867	10.35576

Dcps	ENSRNOG00000009993	22.84105	24.59689	22.14815	20.47511	21.48853	20.80483
Hoxb4	ENSRNOG00000008191	2.705747	3.528689	3.41913	2.14961	3.133394	2.559634
Nr1d1	ENSRNOG00000009329	4.63561	7.771827	7.287506	3.463845	5.873406	6.388672
Ppp1r36	ENSRNOG00000038480	19.21831	14.71693	16.90441	15.40003	13.05627	14.09168
Lbh	ENSRNOG00000039902	49.42267	34.80244	39.76904	35.01638	26.91689	21.39611
Mrps28	ENSRNOG00000032630	10.97574	13.38388	13.90819	9.533471	11.25054	10.7161
Capza1	ENSRNOG00000013538	63.64507	55.21273	53.97432	38.36181	41.90937	40.31887
RT1-N3	ENSRNOG00000000795	57.90817	94.38509	66.65222	47.02122	87.4694	51.01239
Med6	ENSRNOG00000006976	22.76835	20.59799	14.76378	19.08172	18.35641	13.00784
Rhbdd2	ENSRNOG00000001443	14.83063	13.31644	11.10458	13.51004	10.59802	8.014283
Aen	ENSRNOG00000018421	53.16916	59.21862	49.56008	46.61982	48.49851	44.48227
Tmem11	ENSRNOG00000005377	22.85497	22.86331	18.84521	19.96814	21.39083	17.24035
Clcn3	ENSRNOG00000010682	12.65358	16.10807	12.03583	10.06878	12.26467	10.31523
Ccdc97	ENSRNOG00000020675	19.70103	19.39834	18.988	16.86465	17.58617	14.88471
Lig1	ENSRNOG00000014193	9.675867	4.118743	3.505615	8.279898	3.527067	2.352105
Cxcl10	ENSRNOG00000022256	147.5767	561.6301	310.3678	59.58613	353.9535	132.3582
Ctsw	ENSRNOG00000027096	2.170095	4.436422	1.750958	1.41608	3.556406	1.378275
Traf2	ENSRNOG00000006238	30.42309	39.90085	33.59743	27.83211	33.88067	29.17431
Lin7b	ENSRNOG00000020746	0.140268	0.141414	0.328576	0	0	0.062885
Dtx2	ENSRNOG00000001432	6.667173	9.734793	10.12463	5.597431	8.591858	8.039477
Snx6	ENSRNOG00000005249	40.36931	43.41391	41.98163	26.34965	37.44298	31.3679
Cnih4	ENSRNOG00000003717	33.03954	24.35274	23.98807	24.65005	20.77245	17.69938
Rpap1	ENSRNOG00000005483	4.870698	4.846362	5.27905	3.398786	3.914918	4.603566
Cfap52	ENSRNOG00000037718	0.078254	0.138063	0.146647	0	0.089473	0.035083
Ift52	ENSRNOG00000007692	11.25678	10.94343	12.1261	9.329614	9.753651	9.389522
Gtf2a1	ENSRNOG00000004300	12.69922	13.619	11.76658	11.84378	11.58714	10.05493
Tmprss9	ENSRNOG00000032429	0.528754	0.446294	0.414787	0.328514	0.326181	0.319744
Arl10	ENSRNOG00000045948	14.19662	13.29832	12.20238	9.88607	11.45182	8.168799
Toe1	ENSRNOG00000017561	9.909154	9.077872	7.857989	8.071459	8.296738	6.173966
Vps39	ENSRNOG00000008316	14.53538	16.64836	17.97195	13.35426	16.13992	17.12737
Hexim2	ENSRNOG00000021287	9.879186	12.20695	11.2605	8.601621	9.477223	8.236953
Cenpa	ENSRNOG00000032178	7.606529	6.338035	2.766298	5.74352	4.288955	1.899729
Rnd1	ENSRNOG00000059857	35.63101	40.91282	29.89458	19.74488	33.43732	20.3396
Actr2	ENSRNOG00000004959	102.1238	94.34524	83.53498	90.31408	82.31369	78.31613
Ywhah	ENSRNOG00000055471	57.82428	48.58058	31.30464	38.76862	34.7637	23.17637
LOC100909830	ENSRNOG00000049822	3.540151	4.215198	4.375149	2.86771	2.875255	2.763787
Prrg2	ENSRNOG00000020517	2.884837	2.103953	2.588055	1.498444	1.515872	1.568511
Dnal4	ENSRNOG00000015583	21.24647	25.85897	19.5479	17.80356	23.43432	18.07015
Rabep2	ENSRNOG00000018462	11.828	11.38557	12.44915	9.49329	10.34473	10.00984
Dyrk2	ENSRNOG00000007821	17.58982	12.29669	13.31177	13.56137	10.31454	11.04644
Csgalnact2	ENSRNOG00000014713	10.68814	11.97677	9.665126	6.974467	9.699298	7.953824
Rrp7a	ENSRNOG00000022896	2.609446	2.980239	2.002948	1.978132	2.307574	1.718112
Nrip3	ENSRNOG00000013290	3.957889	3.144159	1.700183	2.726779	2.448079	1.0982
Fgf13	ENSRNOG00000042753	3.276984	2.359826	2.263978	3.088438	1.906202	1.909207
Cyp26c1	ENSRNOG00000030698	0.196753	0.283373	0.131684	0.025894	0	0
Tmem253	ENSRNOG00000039489	5.577712	3.460481	3.685206	3.952611	2.550947	2.885316
LOC103690054	ENSRNOG00000020799	16.34366	17.07274	12.13122	11.83649	11.75241	9.93146
LOC680875	ENSRNOG00000023828	0.227343	0.592102	0.550301	0.087265	0.259937	0.23782
Tmem9	ENSRNOG00000010204	64.11031	54.27912	60.64643	38.56821	43.47673	42.39354
Hypk	ENSRNOG00000015703	106.9403	82.35944	70.02614	92.935	75.6469	61.97956
AABR07062138.1	ENSRNOG00000030902	0.475691	0.319718	0.148574	0.292149	0.145037	0.071088
Unc45a	ENSRNOG00000012357	15.33449	16.18131	16.91417	13.14991	10.98347	12.0264
Timp1	ENSRNOG00000010208	398.5444	502.0865	382.0789	261.2409	443.0375	288.5753
Dbf4	ENSRNOG00000050482	3.168405	2.395717	0.948361	2.371567	1.630191	0.611588
Rap1gds1	ENSRNOG00000015987	17.05348	16.34623	13.12109	12.84353	13.09116	11.38235
LOC100911485	ENSRNOG00000046424	38.03572	35.72575	33.18252	36.12843	31.21927	29.99779

Phf5a	ENSRNOG00000024170	57.14833	46.59757	33.11505	39.06972	30.49268	25.66577
Sde2	ENSRNOG00000003247	17.38051	19.22	20.86575	14.58554	13.07845	14.11093
Gltpd2	ENSRNOG00000026488	1.916	1.787231	1.308712	0.742327	1.212045	0.658287
Tmem248	ENSRNOG00000000893	40.09144	35.49841	35.54024	29.58979	30.91024	24.65283
Alkbh1	ENSRNOG00000012264	10.33677	10.96857	9.027171	8.659593	9.941834	8.26614
Nsun2	ENSRNOG00000017254	24.66593	21.9472	17.84385	24.01595	20.41419	16.78501
Fam136a	ENSRNOG00000016273	27.36926	24.68257	20.59246	23.30688	20.30159	18.77772
Senp1	ENSRNOG00000022260	12.85837	9.711197	9.51177	10.43245	6.871171	8.353735
Nme6	ENSRNOG00000020721	17.31749	20.19286	16.95598	15.80511	18.51706	13.89059
Batf2	ENSRNOG00000021018	10.08614	20.96903	15.53754	4.987083	18.86842	10.88305
Klrd1	ENSRNOG00000060246	0.276032	0.126494	0.517281	0.023117	0.022953	0.292502
Pskh1	ENSRNOG00000019290	8.841934	9.235402	9.822404	7.382332	7.577622	6.828127
Casp6	ENSRNOG00000052613	0.142475	0.215459	0.400496	0.109378	0.152041	0.319373
Cnot9	ENSRNOG00000016034	20.84292	24.31998	19.49299	17.88908	23.1169	16.9311
Wdr45	ENSRNOG00000009749	12.50388	15.84889	15.45165	9.828744	14.75235	13.42538
Mitd1	ENSRNOG00000018467	50.5182	87.89385	66.26359	30.4473	78.76734	54.14204
Cdkn1a	ENSRNOG00000000521	119.7551	101.3202	131.83	94.52699	82.87287	90.7759
AABR07028615.1	ENSRNOG00000018500	5.383545	5.014173	3.557776	3.596473	2.44584	2.477494
Dscc1	ENSRNOG00000026502	9.857162	4.342521	3.440907	9.386017	3.182215	2.450972
Zfp473	ENSRNOG00000026572	1.158127	0.892862	0.459597	0.765665	0.324031	0.219902
Mns1	ENSRNOG00000057867	1.895369	1.273903	0.341529	1.678927	1.022427	0.239669
LOC100910795	ENSRNOG00000048193	9.060888	12.6977	9.808242	7.544903	8.992635	7.028114
Fxr1	ENSRNOG00000051480	45.20359	49.52844	44.40882	43.5658	47.79959	43.6939
Hirip3	ENSRNOG00000029061	26.36986	18.25979	11.4384	20.15832	12.86296	8.924755
Ptprn	ENSRNOG00000019587	22.62047	27.0538	9.837427	18.84195	25.25428	7.70908
Tbc1d21	ENSRNOG00000008737	2.057656	2.755635	2.244555	1.471203	2.500135	1.624686
Pym1	ENSRNOG00000058130	24.9648	25.88894	22.26459	21.23204	22.66317	15.54105
Apmap	ENSRNOG00000006795	12.34498	9.90887	10.57621	9.020846	7.972691	9.04162
Ier3	ENSRNOG00000000827	82.64559	71.79148	53.89673	58.43081	57.04778	43.06639
Tgif1	ENSRNOG00000015906	37.53873	56.6407	48.64502	27.6249	50.83986	44.08514
Mrpl19	ENSRNOG00000006968	16.88399	16.53127	17.81967	13.00203	14.62183	13.05772
Enkd1	ENSRNOG00000024364	0.769783	0.632355	0.801426	0.630358	0.31294	0.460147
Tsr1	ENSRNOG00000002980	17.07824	15.81087	14.9971	15.81182	13.06844	13.40639
Gnb2	ENSRNOG00000001409	102.9684	112.2318	93.40043	90.63794	95.02254	86.3335
LOC103690064	ENSRNOG00000045593	11.2266	14.67316	11.6531	7.524532	12.1751	10.09639
Cdt1	ENSRNOG00000013970	6.645654	3.92144	1.593367	4.663692	2.091799	0.788662
Anp32b	ENSRNOG00000009266	15.91698	18.33478	15.12677	11.52757	7.353704	5.929646
Ezr	ENSRNOG00000018524	33.57085	33.7555	20.55867	24.17087	26.12671	16.80399
Dennd4b	ENSRNOG00000022373	4.199676	4.812197	4.775834	3.621765	3.875259	4.362799
Pcif1	ENSRNOG00000016870	7.526853	7.377559	8.37242	7.3091	7.23705	8.04145
Gtf2h3	ENSRNOG00000001035	11.02917	11.05646	10.95707	8.935865	10.2213	9.256019
Pa2g4	ENSRNOG00000004904	33.59376	27.11699	22.59903	26.13542	15.50378	17.61454
Hist3h2ba	ENSRNOG00000043419	15.00466	24.16157	8.13617	5.183567	19.8245	2.117731
Pfdn6	ENSRNOG00000000473	25.56924	31.29884	28.01338	21.07368	23.26682	16.94738
Mbd1	ENSRNOG00000024104	20.56853	21.989	20.93549	15.17187	18.99245	18.37897
Alg8	ENSRNOG00000012292	11.81792	9.2619	7.827354	9.897369	7.921817	7.038091
LOC103689996	ENSRNOG00000046031	15.29339	32.13623	25.25988	12.60811	27.5117	18.69265
Med18	ENSRNOG00000047303	17.11269	9.828264	7.42582	12.79278	5.645291	5.659659
Ssrp1	ENSRNOG00000008825	43.01861	36.31599	27.90782	33.58822	23.4356	22.71118
Atp8b2	ENSRNOG00000020822	71.25714	60.9474	60.83609	46.07402	47.571	48.47843
Rasl11a	ENSRNOG00000000956	0.583515	0.588282	0.771883	0.284588	0.439548	0.615534
Svop	ENSRNOG00000000693	0.18057	0.231694	0.246099	0.105858	0.090091	0.058875
Ccp110	ENSRNOG00000027405	7.099706	5.747857	4.502125	5.045773	4.206374	3.68129
Pex5	ENSRNOG00000049203	8.189833	10.09905	10.23595	7.864262	9.614091	10.03432
Ube2a	ENSRNOG00000039985	8.961827	9.035043	8.187264	7.269836	7.741913	5.312396
Mlst8	ENSRNOG00000009667	5.055721	5.807669	4.851064	3.269219	5.024624	3.759485

Ccdc89	ENSRNOG00000048071	0.104034	0.244729	0.324931	0.031947	0.063439	0.186562
Mrps10	ENSRNOG00000022609	7.252455	5.878038	4.563658	5.141926	4.422513	3.697705
Impdh2	ENSRNOG00000031965	109.449	88.43069	99.25075	107.0272	83.45537	96.60441
Ptx3	ENSRNOG00000012280	5.413583	4.17362	6.543105	3.394644	2.642329	3.120461
Psmd13	ENSRNOG00000014109	89.70419	80.84184	65.11842	76.98214	69.74658	60.11174
Pycr2	ENSRNOG00000003267	31.90699	36.87246	33.88803	28.59406	31.07121	31.23655
AABR07011031 .1	ENSRNOG00000059120	9.205933	4.140818	1.990597	7.306574	3.368241	0.126991
Paics	ENSRNOG00000002101	54.036	49.98448	46.83153	43.62161	45.8595	37.3622
Kpna6	ENSRNOG00000000127	36.76291	33.47771	35.04302	23.96925	28.0109	27.00498
Kbtbd4	ENSRNOG00000009397	6.698309	5.886755	6.880128	4.047854	4.805059	5.060441
RGD1310127	ENSRNOG00000020144	8.536728	6.152417	8.593172	8.195978	5.864204	7.977171
Gpr151	ENSRNOG00000027103	1.098011	0.853957	0.79367	0.895925	0.774779	0.618844
Ddc	ENSRNOG00000004327	0.016839	0.016976	0.007889	0	0.007701	0
Shcbp1l	ENSRNOG00000002691	0.126851	0.063944	0.099049	0.11686	0.038677	0.075827
AABR07045405 .1	ENSRNOG00000059487	28.5679	25.94159	35.69067	16.05199	20.75642	27.43188
Rexo4	ENSRNOG00000027867	37.01926	34.8987	31.42245	32.40434	33.06031	28.08129
Ttc8	ENSRNOG00000004542	7.987412	7.621944	7.17088	7.478085	7.000196	6.928678
Abhd17b	ENSRNOG00000012514	10.83029	10.52627	9.169625	10.56416	9.92258	8.489107
S100a11	ENSRNOG00000010105	416.4172	378.2235	206.2741	363.3927	295.5769	171.6268
Mxd1	ENSRNOG00000017720	3.505336	5.928898	5.881309	3.060221	5.061194	4.744856
Tgfbr1	ENSRNOG00000007036	12.57103	13.37323	12.05163	10.08947	10.89811	11.04428
Prkd3	ENSRNOG00000005289	23.00802	22.88583	22.58795	14.6974	18.97492	18.06847
Fam241b	ENSRNOG00000000551	7.624936	10.33307	11.2651	4.933396	9.245235	9.412585
Sema4b	ENSRNOG00000025167	17.70781	18.82161	13.73592	13.90108	13.9296	11.8288
Cxcl16	ENSRNOG00000026647	169.3048	285.8946	146.4201	148.8287	274.6004	117.9511
Chd4	ENSRNOG00000018309	58.11438	59.20568	60.14548	53.94737	49.60005	54.36069
Dnajc16	ENSRNOG00000012503	4.706839	4.896739	5.473762	3.628804	4.457983	4.384977
Fopnl	ENSRNOG00000053230	45.23741	37.08601	31.00132	22.4166	25.86146	19.25607
Cdc123	ENSRNOG00000017770	55.63509	48.78681	48.7048	52.92137	42.14679	41.91646
Sike1	ENSRNOG00000017502	8.697437	10.71911	7.562424	8.0124	9.303872	6.823682
Snx11	ENSRNOG00000008642	10.12623	11.71344	13.41194	6.747495	10.34881	10.02171
Ccdc25	ENSRNOG00000015939	18.3999	17.95316	16.62831	14.54258	15.18646	15.10651
Pycr3	ENSRNOG00000054724	34.30684	32.9931	24.73795	27.1251	29.60656	20.89071
Pdlim3	ENSRNOG00000012658	0.143174	0.096229	0.089436	0.021983	0	0.042792
Loxl3	ENSRNOG00000061373	117.3324	209.6374	85.00023	82.28476	166.4923	68.43126
Gtf3c4	ENSRNOG00000054725	6.904018	6.697644	7.439986	6.243104	6.431356	6.994168
Rab27a	ENSRNOG00000052499	7.60466	6.298308	7.462656	5.46896	4.741998	3.871241
Ifi35	ENSRNOG00000020678	86.17872	163.3017	119.4198	67.15674	155.8601	101.4532
LOC100912534	ENSRNOG00000001748	12.85209	14.68843	13.44384	10.56398	11.19833	12.02045
MGC116202	ENSRNOG00000015625	3.075399	1.650639	1.762151	2.955466	1.396404	1.626747
Ttc4	ENSRNOG00000042467	12.54678	12.53036	12.00748	8.72325	10.99581	9.423058
LOC103690120	ENSRNOG00000015799	0.223907	0.102607	0.267018	0.056256	0.037237	0.109508
Tmem82	ENSRNOG00000011970	0.844615	0.883053	0.937957	0.634	0.801177	0.785367
Sf3a3	ENSRNOG00000007629	22.07459	19.22606	14.6322	16.59139	14.6807	12.5471
Bcas2	ENSRNOG00000018783	45.12122	41.4642	37.30226	36.74846	38.24057	31.04137
Fbxw7	ENSRNOG00000010889	3.498666	3.002434	3.539133	3.000048	2.712977	3.321586
Nrxn2	ENSRNOG00000021103	0.01723	0.01158	0.032288	0.010582	0	0.015449
RGD1563917	ENSRNOG00000017273	1.064931	2.096137	0.71274	0.280301	0.974085	0.272818
Diexf	ENSRNOG00000004789	9.642078	10.56421	9.295861	8.679838	8.309431	7.94807
Speg	ENSRNOG00000019850	0.624198	0.640402	0.946114	0.449879	0.557515	0.727587
Rhot2	ENSRNOG00000019930	11.46336	9.961336	10.10759	10.31981	9.47345	9.424286
Tap2	ENSRNOG00000000455	33.44505	69.43713	54.49477	26.78912	56.79477	37.23462
Zbtb6	ENSRNOG00000009340	3.942175	5.732633	5.174726	3.330432	4.453344	4.512065
Suox	ENSRNOG00000005987	1.557281	1.328465	2.431943	1.397839	1.077456	2.040782
Las1l	ENSRNOG00000021748	16.48411	10.51011	9.929276	13.34544	7.829374	8.742474

Rpf1	ENSRNOG00000015969	21.92885	24.06895	21.75244	18.23641	22.66946	18.90286
Plat	ENSRNOG00000019018	109.7189	105.547	70.67214	51.86286	75.91889	42.85251
Cbarp	ENSRNOG00000024349	1.970438	1.852987	2.482409	1.754223	1.332827	1.915248
Fbf1	ENSRNOG00000008577	9.860557	8.247958	8.346659	7.925801	7.339477	5.93536
Aldh9a1	ENSRNOG00000004027	112.4065	103.4278	92.91367	103.1215	98.99603	81.1683
Nrg4	ENSRNOG00000015149	0.370878	0.467385	0.695022	0.213541	0.381644	0.623522
Ncbp2	ENSRNOG00000001746	28.96155	28.28647	28.8984	26.15429	27.23102	26.54428
Lce1m	ENSRNOG00000009581	0.791862	0.159666	0.049465	0.729493	0.048287	0
Ddx24	ENSRNOG00000009166	41.51333	45.5449	46.60467	34.22625	42.69893	41.56411
Zfp518b	ENSRNOG00000028534	3.231031	2.576771	3.38895	2.210195	2.150385	2.76736
LOC103692716	ENSRNOG00000059714	129.6405	103.4079	78.74161	107.7644	81.72963	70.20002
Yipf1	ENSRNOG00000010512	38.36501	45.1839	46.66337	32.54759	41.34511	37.32125
RGD1310429	ENSRNOG00000000237	9.725242	11.90036	14.92087	9.164425	11.03463	13.54606
Adprh	ENSRNOG00000027260	21.86741	20.27657	20.43575	19.74746	19.26505	19.34926
Acsl4	ENSRNOG00000019180	20.95808	31.5291	22.8098	15.65924	26.03462	20.69409
Pde6g	ENSRNOG00000046962	0.497314	0.501376	0.621308	0	0.303259	0.297275
Tmed5	ENSRNOG00000000073	31.69415	40.09705	31.30014	30.90504	38.07437	29.27045
Rrn3	ENSRNOG00000003326	32.89212	31.87505	31.2149	21.56537	24.99656	26.53216
Cstf2t	ENSRNOG00000050289	14.39156	17.89831	16.49679	12.04865	11.57028	11.22094
Blmh	ENSRNOG00000003563	30.16959	27.74978	25.36456	28.58477	25.82398	21.82534
Hsp90ab1	ENSRNOG00000019834	423.2289	524.1477	463.6131	394.2879	446.0492	396.8565
Oser1	ENSRNOG00000008297	34.82729	39.73896	31.31758	30.71625	37.93119	26.43455
Bysl	ENSRNOG00000049121	21.74079	18.71336	13.69279	17.78466	13.03856	11.51693
Dnaaf5	ENSRNOG00000021312	4.141344	4.034298	3.963744	2.855518	3.555662	2.972923
Btg1	ENSRNOG00000004284	57.32372	105.1725	68.38123	48.31377	86.08419	58.61889
Vps37b	ENSRNOG00000001086	10.2636	8.701667	7.257464	7.967334	7.640694	6.057333
RGD1560854	ENSRNOG00000037715	4.184413	3.831572	3.597044	2.510948	2.774026	2.925812
Cdk9	ENSRNOG00000022586	23.26738	28.54431	24.4715	19.02637	22.69337	22.26899
Tspan31	ENSRNOG00000025592	75.14846	79.97521	77.9288	72.20982	73.58743	69.95974
Rprd1b	ENSRNOG00000012923	8.353419	7.396741	7.723828	7.108726	5.993894	7.204489
Cdh11	ENSRNOG00000013481	47.24065	45.77578	43.57464	34.39345	40.9511	31.49541
Trim26	ENSRNOG00000032930	16.1993	20.4079	20.87788	14.55901	18.36294	17.15729
Cyp1b1	ENSRNOG00000040287	277.9414	242.0444	320.4293	214.3915	218.2191	274.1612
LOC103690005	ENSRNOG00000020781	33.33791	35.44357	30.78312	26.8033	32.97043	24.45725
Srp68	ENSRNOG00000009351	15.94138	17.09504	16.06432	14.47797	13.24024	12.30473
Arhgef1	ENSRNOG00000020130	10.51962	10.01844	10.89012	9.040422	6.528149	8.876841
RGD1307554	ENSRNOG00000018408	2.837952	2.28891	1.983282	1.786525	0.822021	1.431354
Tspan1	ENSRNOG00000023320	0.202475	0.379098	0.162616	0.053294	0.158745	0.077806
Srpx2	ENSRNOG00000003715	115.8052	63.88822	42.3445	110.8566	57.25538	39.88059
Rnase10	ENSRNOG00000010261	0.136975	0.138094	0.320863	0	0.062645	0.122818
Prcc	ENSRNOG00000012933	25.04075	22.83295	22.77472	24.07396	20.96548	20.19417
Timm13	ENSRNOG00000019682	103.9997	102.5474	90.41761	80.78351	94.03378	69.90136
Tceanc	ENSRNOG00000004531	1.056503	1.843501	1.865653	0.748684	1.040712	1.05661
Plekhd1	ENSRNOG00000038297	0.104168	0.091892	0.073204	0	0	0.035026
Fignl1	ENSRNOG00000004440	5.629341	2.074853	0.978366	4.614386	1.093496	0.597017
Cdk2	ENSRNOG00000006469	15.36615	9.33525	6.095783	12.81381	5.25705	4.473359
Kbtbd6	ENSRNOG00000047604	3.143376	2.726863	2.990994	2.806002	1.872239	2.075629
Mon1b	ENSRNOG00000011552	9.828907	12.33729	11.18169	6.955968	8.911046	9.941396
Nhp2	ENSRNOG00000004247	123.7997	82.27994	50.24195	114.6542	72.60741	46.66415
Prelid3b	ENSRNOG00000046644	56.26747	44.87149	38.14701	43.74492	39.41754	31.29881
RGD1359127	ENSRNOG00000016399	24.33418	27.3469	22.01391	22.09301	22.65489	19.70519
Pcsk7	ENSRNOG00000017478	18.01137	17.18939	13.78333	15.10126	13.89489	12.58865
RGD1306502	ENSRNOG00000022745	2.072273	2.573142	2.084322	1.725698	1.734858	1.595646
Parvb	ENSRNOG00000055305	6.981963	6.460139	5.821151	5.215455	5.314958	5.137998
Klhl15	ENSRNOG00000006515	2.215638	2.672851	2.253483	1.709662	2.130558	2.054555
Utp6	ENSRNOG00000014209	53.4562	45.20688	43.13917	38.5804	38.75411	35.00463
Ppie	ENSRNOG00000014762	30.57841	42.07063	28.88184	26.07013	30.66419	21.78969

Efcab14	ENSRNOG00000010091	7.28236	7.5674	8.948668	4.797061	5.863764	8.016836
Nol12	ENSRNOG00000010209	4.467826	7.412633	4.680783	2.398256	2.63865	2.081881
Tnfaip2	ENSRNOG00000010165	54.89754	62.98038	47.64809	36.15437	51.66927	40.11874
Ptcd2	ENSRNOG00000028403	3.243654	4.308298	3.473475	3.035605	3.767553	2.908399
Apol3	ENSRNOG00000042771	7.556101	21.5225	18.21332	5.977626	18.52491	13.95323
Anks6	ENSRNOG00000023309	5.539342	6.731057	5.883256	4.550323	4.428661	4.616506
Gkap1	ENSRNOG00000019272	9.862559	13.85198	10.85327	8.572307	13.38775	9.842668
Mapkapk5	ENSRNOG00000001345	26.76922	22.58582	20.27069	25.68928	21.85325	19.86485
Col11a2	ENSRNOG00000000463	0.305457	0.405937	0.286211	0.262212	0.304799	0.230313
LOC103692531	ENSRNOG00000040303	7.564077	4.539211	5.062506	5.475098	3.788871	3.229655
LOC498265	ENSRNOG00000059276	7.033187	4.759666	3.295033	5.637441	3.6725	2.793125
LOC100911248	ENSRNOG00000001868	5.986163	5.42722	6.069029	5.054457	4.876741	4.633769
MGC116121	ENSRNOG00000015546	14.42441	17.01403	14.01336	10.69087	15.24962	9.123096
Rcor2	ENSRNOG00000021183	6.961594	4.937616	5.785461	5.78484	3.999825	5.363783
Dxo	ENSRNOG00000000422	4.430943	4.370031	4.603055	3.697417	3.113137	4.088131
Ints3	ENSRNOG00000015153	21.56397	20.84684	18.19293	16.0326	17.99775	15.69094
Leo1	ENSRNOG00000010116	9.86175	9.239326	8.540386	8.16733	7.760036	7.934401
Ncl	ENSRNOG00000018273	45.23819	47.02608	33.60547	35.42929	27.48785	24.58978
Cpsf2	ENSRNOG00000005733	16.99498	16.84107	12.21525	10.45402	9.534007	9.599383
RT1-T24-4	ENSRNOG00000042905	132.7335	294.0331	281.136	91.98001	279.4541	244.7608
Tspan2	ENSRNOG00000023338	3.038662	2.107587	1.445014	2.77828	1.379272	0.880884
Map3k6	ENSRNOG00000008936	5.40615	6.24346	3.893661	3.893217	4.502134	3.273793
U2af1	ENSRNOG00000045860	63.73562	44.26031	42.25675	47.05727	36.03143	34.45389
Xab2	ENSRNOG00000000988	19.4679	19.42151	18.75539	17.29918	17.31968	17.96171
AABR07043951.1	ENSRNOG00000011422	4.998585	6.168346	5.841045	4.402819	4.793915	5.102999
Ccdc82	ENSRNOG00000005713	9.839625	8.809744	7.51483	8.899213	7.99291	7.18046
Clcn1	ENSRNOG00000016917	0.164267	0.151809	0.089785	0.063054	0.037564	0.049096
Nkx6-1	ENSRNOG00000002149	0.013621	0.027464	0.012763	0	0	0
Hist1h2aa	ENSRNOG00000048122	0.111358	0.224535	0.104342	0	0	0
Ubqln4	ENSRNOG00000019933	18.62007	15.84632	14.22225	14.37345	12.66215	12.70013
Ifit3	ENSRNOG00000022839	149.4168	794.8491	676.9949	58.05989	672.8409	458.6798
Txndc11	ENSRNOG00000002452	9.271102	7.107048	7.792931	7.622506	5.588342	7.20193
Cir1	ENSRNOG00000018719	10.9409	17.11171	12.19663	9.291723	13.00089	9.766281
Mok	ENSRNOG00000007850	3.321105	5.562394	5.621426	2.6154	3.846235	3.626246
RGD1564855	ENSRNOG00000059655	3.719104	3.587872	2.944042	1.978912	2.404747	2.299798
Chchd7	ENSRNOG00000031769	26.80096	39.33279	31.31101	22.18977	35.06525	29.65837
Spef2	ENSRNOG00000058275	0.007529	0.02277	0.021163	0	0.006886	0.013501
Kpna3	ENSRNOG00000014945	20.03634	21.58214	17.3511	18.12791	19.9476	16.67949
Tarbp2	ENSRNOG00000042355	13.70405	13.78598	12.42192	12.59723	10.9	9.322577
Slc16a2	ENSRNOG00000002832	2.705777	5.048619	5.537301	1.512959	3.460012	4.972933
Cdkl4	ENSRNOG00000040266	0.754545	0.811423	0.848406	0.695114	0.7822	0.766765
LOC108348298	ENSRNOG00000061236	2.254582	2.522097	2.401921	1.849387	2.373	1.993862
Tbl3	ENSRNOG00000013429	29.17005	25.13359	21.63601	23.31148	22.80322	18.16963
Naa10	ENSRNOG00000060063	22.39501	20.6024	14.03307	18.86887	15.66215	12.25737
Oxld1	ENSRNOG00000047517	8.127524	9.804695	9.372868	6.975417	8.89561	8.969212
Foxj3	ENSRNOG00000061851	12.82679	12.08961	11.98462	11.9085	11.34232	11.66384
Cacybp	ENSRNOG00000002572	49.73855	41.72758	33.5169	47.26086	40.8627	31.52352
Tmem63a	ENSRNOG00000003310	17.04301	21.21948	18.18035	16.04101	18.43441	15.47385
Med21	ENSRNOG00000001820	18.24402	19.17102	14.77053	15.94386	18.0489	11.61394
Letm1	ENSRNOG00000016427	25.68415	27.0056	27.72749	23.48194	21.86165	24.99225
G3bp1	ENSRNOG00000013186	52.67134	37.18867	34.61335	37.75567	27.64037	29.0123
Ly6g6c	ENSRNOG00000000843	0.114865	0.231607	0.322884	0	0	0
Lrrc10b	ENSRNOG00000030180	0.035609	0.0718	0.100097	0	0	0
AABR07043407.1	ENSRNOG00000060719	0.138932	0.280134	0.390536	0	0	0
Smarca4	ENSRNOG00000009271	32.86199	26.41087	30.44541	21.99023	21.72619	24.74366

Wdr74	ENSRNOG00000042878	24.14082	22.55918	18.53675	16.82117	19.63609	14.31061
Cyb5b	ENSRNOG00000011142	32.14078	27.47241	22.71273	21.89749	21.97546	18.38523
Cenpn	ENSRNOG00000011296	12.40843	7.297388	3.115346	11.37248	6.184244	2.724427
Sem1	ENSRNOG00000010420	86.82975	91.93367	73.40835	82.50721	85.42975	71.0286
Spout1	ENSRNOG00000025711	5.942658	6.548012	5.588945	4.009276	5.112394	4.912466
Efnb1	ENSRNOG00000006877	5.096587	4.624403	5.84368	4.398627	4.342846	5.0268
Snrnp200	ENSRNOG00000012157	46.31748	51.06066	50.05987	39.724	47.62087	47.21757
LOC102553386	ENSRNOG00000053557	95.34856	82.95501	75.65831	84.75655	77.45522	60.31981
Atp6v1e2	ENSRNOG00000015566	1.14703	0.489246	0.496044	0.975402	0.322825	0.435124
Styxl1	ENSRNOG00000023366	4.381702	3.690229	5.207149	3.667386	3.152565	4.958939
Synj2	ENSRNOG00000017114	8.319618	8.948486	9.223008	7.262198	8.471098	7.843456
Ccdc65	ENSRNOG00000058916	1.946078	2.102118	1.866883	0.853714	0.911223	1.454114
Cldnd1	ENSRNOG00000001657	11.93354	14.82435	13.30763	10.89313	12.85119	10.4067
Ttc9c	ENSRNOG00000019464	41.90827	38.48771	29.26684	32.09244	30.26726	25.9123
Psmd4	ENSRNOG00000021042	116.0483	124.7585	100.0624	103.9503	120.1763	92.65072
Fgfbp3	ENSRNOG00000022796	1.143648	1.383589	1.452604	0.796031	1.208811	0.957081
Lzic	ENSRNOG00000016200	11.76641	12.05009	10.10995	8.811676	9.599885	9.10464
Coa7	ENSRNOG00000010636	6.249692	7.364102	5.900187	4.278204	5.975738	5.208515
Ngdn	ENSRNOG00000018069	40.37755	47.49812	39.84639	34.78232	45.0257	37.05303
LOC100910308	ENSRNOG00000046308	33.25709	33.44732	27.71617	24.79305	25.79973	24.81958
Rnf216	ENSRNOG00000001101	9.292756	11.37454	11.20775	8.866313	10.13214	10.0667
Pnn	ENSRNOG00000004061	12.50388	12.9814	10.78578	10.733	8.911377	8.721613
LOC103689986	ENSRNOG00000020616	10.07039	8.788868	8.356177	7.015601	6.965767	7.187692
Nol10	ENSRNOG00000005344	8.676342	8.285224	7.142109	7.021989	4.97409	5.684023
Nepro	ENSRNOG00000013721	4.746457	5.544127	5.211503	4.507449	4.991827	4.930817
Rinl	ENSRNOG00000025354	0.420804	0.729165	0.455899	0.302859	0.481133	0.341938
Tex101	ENSRNOG00000020057	0.780026	0.786399	1.192491	0.416025	0.638381	0.993891
Clic3	ENSRNOG00000015184	0.195498	0.337877	0.628048	0.102914	0.063864	0.388146
Bend3	ENSRNOG00000046505	1.082218	0.812728	1.510704	0.986806	0.565656	1.227807
Pola2	ENSRNOG00000020906	14.21154	6.193026	4.091296	12.54159	4.41912	3.483396
Itgb4	ENSRNOG00000005580	0.464541	0.546392	0.689181	0.392289	0.430813	0.497518
Mtrex	ENSRNOG00000010125	36.72733	32.59686	28.25385	27.5768	25.93336	25.11598
Cep63	ENSRNOG00000008410	12.42943	9.473862	9.722532	11.82873	8.82659	9.502073
Tonsl	ENSRNOG00000014703	2.112938	0.966384	0.53117	1.053968	0.37711	0.110901
Eif3d	ENSRNOG00000005804	126.9699	120.576	108.015	100.9193	111.7846	88.30104
Slc25a19	ENSRNOG00000003918	6.511023	5.904798	4.568636	5.696915	5.139753	4.291908
Ppp1r13l	ENSRNOG00000025350	1.200938	1.531242	1.489333	0.634523	0.920793	1.282673
Tardbp	ENSRNOG00000012455	68.09534	62.89968	46.50944	55.8373	46.84521	41.10602
Nob1	ENSRNOG00000021890	18.23244	24.70091	21.75023	15.50437	17.35778	17.27189
Wnk4	ENSRNOG00000020441	0.556317	0.540089	0.37647	0.265741	0.216736	0.267883
Wdr46	ENSRNOG00000031171	21.77486	21.54115	17.81615	19.71951	17.49878	16.09009
Prss42	ENSRNOG00000031971	0.019803	0.039929	0.092774	0	0	0.035512
Zfp263	ENSRNOG00000007678	8.985884	8.173258	8.100424	8.525976	7.53028	7.880225
Lrrc8e	ENSRNOG00000028460	7.009183	6.25675	7.456934	4.50653	5.382779	4.936134
Wdr12	ENSRNOG00000017340	29.0316	35.30936	23.44044	22.76168	28.4195	21.12939
Csnk2b	ENSRNOG00000000847	43.68954	42.30452	36.03373	34.9047	38.65301	25.05359
Cd68	ENSRNOG00000037563	2.609594	5.29738	4.26254	2.014207	3.548209	3.003891
Nup93	ENSRNOG00000018564	22.17464	14.28606	11.63115	16.66621	9.866191	9.802782
Tcof1	ENSRNOG00000026108	9.049491	7.279776	7.147707	6.315117	4.215219	6.127784
Gnl2	ENSRNOG00000009430	15.66242	14.67853	12.80764	14.4288	12.37669	11.87573
Fosl1	ENSRNOG00000020552	9.026405	10.05527	5.572714	4.945636	6.76398	4.223701
Gart	ENSRNOG00000028292	40.74719	32.65873	23.68582	33.63084	26.65932	21.33655
Map3k11	ENSRNOG00000020773	12.83349	11.40435	14.15179	8.640099	9.639657	8.823197
Tkfc	ENSRNOG00000020704	15.88716	14.28489	13.41489	13.3084	13.43354	11.32983
Cyp2j4	ENSRNOG00000031004	0.588646	1.506464	1.103118	0.437998	1.09757	0.649607
Btbd7	ENSRNOG00000008598	15.12133	13.72918	16.01354	10.95483	11.71098	14.17591
Sf3b5	ENSRNOG00000014908	82.17966	85.70157	83.45242	68.40237	75.33782	57.7582

Ppp2ca	ENSRNOG00000056485	75.99806	74.24685	74.07589	62.51245	69.73011	64.0925
Fam151a	ENSRNOG00000007799	1.750544	1.470704	1.718359	1.401483	1.162783	1.009036
Ppm1h	ENSRNOG00000004314	2.002848	2.896334	2.785269	1.703167	2.694104	2.68157
Setmar	ENSRNOG00000006806	2.096132	1.947872	2.20317	1.208995	1.517181	1.814108
Txlna	ENSRNOG00000048242	44.63681	32.59025	33.54623	43.84986	31.85505	33.28364
Pln	ENSRNOG00000000413	2.194004	2.290227	2.110351	1.52037	1.349733	1.793146
Dctn2	ENSRNOG00000025481	129.5283	161.2775	135.7154	124.5556	158.3623	127.6746
Chrdl2	ENSRNOG00000018394	0.243432	0.599916	0.278782	0	0.222665	0.145514
LOC100911313	ENSRNOG00000049007	0.975777	2.065872	1.188588	0.719137	1.96358	1.049908
Aimp1	ENSRNOG00000011384	40.05812	37.79002	27.30098	35.89689	34.71429	25.91539
Eml2	ENSRNOG00000030127	13.98712	21.1119	19.33752	11.66596	14.01934	13.27077
Cachd1	ENSRNOG00000010514	3.978508	4.530958	4.245601	3.438905	3.122449	3.446188
Dkk4	ENSRNOG00000019267	4.578887	4.586704	5.583017	4.191199	3.705013	5.15835
Epha10	ENSRNOG00000037340	0.243928	0.144659	0.389896	0.158623	0	0.154388
Sfi1	ENSRNOG00000018412	6.887264	5.374739	5.961572	6.378731	4.960617	5.795643
Tigar	ENSRNOG00000051816	5.038465	5.323841	3.661817	3.763877	4.18028	3.243479
Arfgap2	ENSRNOG00000014429	48.45708	45.83162	43.38347	37.17602	39.31956	39.12656
Slc26a4	ENSRNOG00000058692	0.054314	0.054758	0.050892	0.025018	0.01242	0.036525
Chchd4	ENSRNOG00000033038	6.251941	5.987867	5.56514	4.799599	3.717094	4.764894
Chaf1b	ENSRNOG00000001692	12.45778	4.750505	2.86278	11.27835	3.247291	2.372939
Cdk5rap2	ENSRNOG00000005788	12.37503	11.22488	9.086968	10.79542	8.111652	7.789831
Spata5	ENSRNOG00000017462	9.606966	8.357392	7.29361	7.367071	7.059149	6.454547
AABR07054578.1	ENSRNOG00000019099	5.033991	5.658216	5.479549	3.67053	3.879583	4.897835
LOC103689945	ENSRNOG00000009596	15.53342	12.4116	14.28368	13.75474	11.43721	13.58928
Prkar2a	ENSRNOG00000020284	24.86279	18.08692	15.04413	16.2769	11.719	12.21893
Tjap1	ENSRNOG00000018980	5.989152	6.871912	6.867786	5.622521	5.752147	6.022217
Lurap1	ENSRNOG00000023459	1.833844	2.193522	1.68918	1.460333	1.648973	1.504955
Foxp4	ENSRNOG00000022804	1.651851	1.433462	2.243297	1.55064	1.185798	1.93108
Slc11a2	ENSRNOG00000019550	22.70948	29.839	27.21022	20.45335	27.26257	21.94354
Pfdn2	ENSRNOG00000003983	3.25536	5.081738	4.058812	2.684551	3.409895	2.942434
Zyx	ENSRNOG00000017354	33.58402	27.30404	27.89326	26.27358	24.91327	22.44275
Gbp7	ENSRNOG00000029191	115.7941	330.6265	167.7153	80.97675	319.4107	137.9679
Lonp1	ENSRNOG00000046502	51.00569	58.55941	53.80082	47.07018	47.54862	47.03525
Rbm8a	ENSRNOG00000021215	10.27826	12.91567	9.796255	7.678066	4.956639	3.855373
Ifi44l	ENSRNOG00000049994	19.74618	98.14632	56.70691	9.140858	92.08159	39.62328
Fam117a	ENSRNOG00000004417	3.688761	4.27347	3.183483	3.293891	3.359285	2.741744
Hspd1	ENSRNOG00000014525	176.8033	145.1024	108.5645	118.5421	95.28529	89.86329
Nup88	ENSRNOG00000006126	33.18557	31.13357	25.41642	27.94752	26.67023	23.73615
RT1-CE7	ENSRNOG00000031090	80.08451	105.7324	117.7844	68.00554	101.6592	105.5583
Mus81	ENSRNOG00000020617	39.88202	31.56424	26.35834	31.41632	27.64272	22.56509
Rnf144a	ENSRNOG00000007370	8.779758	6.619447	8.598733	8.407407	5.456913	7.622272
Cep78	ENSRNOG00000014041	4.976402	4.274461	3.181523	4.501693	3.368719	2.818987
LOC108348065	ENSRNOG00000047281	14.29762	8.778425	10.96103	11.17194	7.572042	9.255643
Fcgr2b	ENSRNOG00000046452	0.425923	0.620248	1.008806	0.119893	0.476164	0.572851
Phka1	ENSRNOG00000003063	4.11206	4.537903	4.588381	3.164027	4.215325	3.963728
Rgp1	ENSRNOG00000016309	14.0058	11.99302	11.75991	12.39996	11.01414	11.18822
Ltb	ENSRNOG00000058566	0.133527	0.201927	0.062557	0.092258	0.091602	0
Arvcf	ENSRNOG00000001888	5.330778	4.095286	5.660178	4.106721	3.694281	4.477152
Etfb	ENSRNOG00000017851	37.44968	29.00916	34.45793	26.55214	25.52589	25.8865
Lyrm1	ENSRNOG00000024530	3.629348	3.730047	4.42419	2.012585	3.126356	2.527556
Drg2	ENSRNOG00000055309	31.41386	31.58992	30.48322	28.46096	29.68453	25.33598
Zmat3	ENSRNOG00000010119	18.41959	18.87958	16.52397	12.91515	15.16344	14.68071
Lmnb1	ENSRNOG00000013774	45.7195	22.32759	13.66877	39.67847	15.05793	11.36288
Spag7	ENSRNOG00000004246	9.510715	10.23726	9.64854	8.728682	7.816363	7.373586
Men1	ENSRNOG00000021054	12.38842	13.82304	14.19661	10.20777	12.44728	13.44027
Lrrd1	ENSRNOG00000026196	0.031851	0.128446	0.074611	0	0.029134	0

Tbrg4	ENSRNOG00000052477	22.08081	17.52234	16.96016	17.51303	15.94969	14.05445
Sec24c	ENSRNOG00000009042	32.65057	28.01931	32.6742	23.40562	24.99562	26.14571
Fdxr	ENSRNOG00000058497	4.801871	3.954365	3.853389	4.598855	3.848644	3.772696
Jpt2	ENSRNOG00000024661	48.92804	28.88334	19.68184	38.71619	20.21015	16.45515
Mob1a	ENSRNOG00000059474	61.3485	61.16861	58.90757	53.60602	58.72314	52.48669
AABR07072108.1	ENSRNOG00000006943	4.349177	4.430382	3.947811	3.881415	3.02506	2.518529
Cct4	ENSRNOG00000009642	182.6041	195.5549	154.5801	150.3562	180.1361	140.763
Dlst	ENSRNOG00000005061	50.25175	42.81925	44.71	46.52589	39.43966	36.86809
Iqce	ENSRNOG00000001241	3.869508	4.084272	4.08531	2.861828	2.907967	3.713902
11-Sep	ENSRNOG00000002182	40.12005	35.18691	25.05512	25.90429	18.12282	19.67552
RGD1563349	ENSRNOG00000005818	0.121819	0.061407	0.028536	0.042084	0.013928	0
Itfg2	ENSRNOG00000006264	6.556709	6.886527	6.308661	6.382866	6.337526	5.879026
Zfp64	ENSRNOG00000012762	6.579532	8.055974	6.826119	4.858796	6.921796	6.247449
Sap18	ENSRNOG00000010732	34.4804	25.28681	25.93284	26.61216	22.30935	21.6106
Dcaf7	ENSRNOG00000042245	10.24271	11.30986	11.66133	8.196676	8.116865	10.57365
Rdm1	ENSRNOG00000020751	9.832449	8.337851	7.577017	6.454894	6.955387	5.891195
Atg2b	ENSRNOG00000004519	7.157279	9.385045	9.961886	6.0886	7.856055	9.463814
Rassf4	ENSRNOG00000013526	37.1047	22.33495	23.54632	20.19175	12.54679	17.4076
Cxcl11	ENSRNOG00000022298	11.58779	79.84903	20.50315	7.902681	71.13344	8.902203
RGD1560917	ENSRNOG00000034128	21.82777	21.67927	11.74501	17.12212	17.89006	10.2703
Foxk1	ENSRNOG00000001104	4.685592	6.314322	7.743836	4.012864	4.163835	5.897305
Erh	ENSRNOG00000004883	125.4819	115.022	87.34975	112.6891	94.09171	80.0482
Acat2l1	ENSRNOG00000013975	6.230383	5.064149	3.070422	5.34245	4.022744	2.744888
Paip2b	ENSRNOG00000014092	12.13782	14.00455	9.414389	9.753022	10.30054	8.162467
RGD1563888	ENSRNOG00000001554	2.020968	3.6141	2.479765	1.75097	2.750842	1.790514
RGD1561590	ENSRNOG00000020771	128.2551	89.51743	72.68939	97.80084	77.36096	57.15929
Senp3	ENSRNOG00000013746	25.29332	23.85292	21.59269	22.13254	21.69404	20.55937
Dbr1	ENSRNOG00000014588	18.4042	14.13681	10.43066	17.86506	13.40595	10.1988
Zfp1	ENSRNOG00000019059	3.511223	4.452256	4.205774	3.034585	4.072556	4.057103
Sipa1	ENSRNOG00000020726	14.55827	8.706603	10.88431	12.19663	7.67894	9.790177
Fmo5	ENSRNOG00000018076	2.305403	2.737874	3.130391	1.529872	2.430408	2.732807
Stx4	ENSRNOG00000019302	68.23193	82.79849	70.0539	60.8402	78.92072	58.53881
Ybey	ENSRNOG00000021365	0.574403	0.688925	0.686686	0.41968	0.606931	0.443995
Gse1	ENSRNOG00000017489	1.409821	1.56049	1.542701	1.153461	1.082142	0.786751
Tsta3	ENSRNOG00000009020	34.37442	37.19643	32.65152	19.76647	31.12503	25.56694
Blm	ENSRNOG00000011213	4.955108	2.418813	1.293777	3.888223	1.630232	0.957081
Actr6	ENSRNOG00000007875	14.9771	17.24921	18.10081	14.10147	16.09104	15.86455
Dpf2	ENSRNOG00000020892	23.32416	27.38064	24.03022	20.25554	18.8406	19.11545
Imp4	ENSRNOG00000013285	29.78634	30.68746	27.20429	23.92644	28.87503	22.07267
Plin2	ENSRNOG00000007060	59.90435	108.2865	91.73505	49.98395	97.29932	68.39627
Ahdc1	ENSRNOG00000042855	4.44907	3.832202	5.099648	3.839995	2.950082	4.815474
Cdk19	ENSRNOG00000000583	9.607381	12.06915	10.05279	6.295984	8.808595	8.994834
Hpf1	ENSRNOG00000011293	21.03265	15.14606	12.21008	16.81865	11.23237	10.89358
AABR07043601.3	ENSRNOG00000049551	0.50303	0.338093	0.314224	0	0	0.150346
LOC100910418	ENSRNOG00000060898	148.5696	139.4075	94.44277	69.45594	105.6678	57.44239
Arl6ip4	ENSRNOG00000001080	25.25531	25.53599	27.22238	19.2922	23.60672	21.15737
Gpn1	ENSRNOG00000004941	22.24793	16.69742	16.70926	15.03937	14.20365	12.35915
Chmp5	ENSRNOG00000008672	81.89096	100.5761	89.1887	63.98738	94.9202	76.34023
Uba3	ENSRNOG00000006221	50.13104	54.08659	48.54464	47.18815	52.8806	44.65963
Wdr5	ENSRNOG00000008212	28.93542	27.39044	21.17864	20.18436	20.55817	18.47945
Cyp2d2	ENSRNOG00000008988	0.052286	0.105427	0.048992	0.024084	0.023913	0
LOC100911825	ENSRNOG00000046663	0.73981	1.003045	0.836619	0.293767	0.583361	0.697656
H2afv	ENSRNOG00000052275	35.43096	36.97704	33.15541	33.2995	36.08212	30.27878
Smtn	ENSRNOG00000019451	33.4546	13.19498	18.02938	22.83431	8.4975	13.27787
LOC108348069	ENSRNOG00000047594	4.994651	5.058494	4.245617	4.16519	3.3473	3.544682

Abt1	ENSRNOG00000017585	19.9665	20.84732	18.89911	16.27031	19.56552	14.71131
Cbfa2t2	ENSRNOG00000016352	5.910358	6.620716	8.472636	5.274203	5.467771	6.597927
Tceal1	ENSRNOG00000002387	3.656293	3.64855	5.733191	2.784013	3.378512	4.917583
Rpp30	ENSRNOG00000018718	10.43874	8.928545	7.414212	9.784773	7.599622	5.403046
Sdhb	ENSRNOG00000007967	85.96419	89.41282	75.86165	61.95597	81.49014	60.42206
Dclre1b	ENSRNOG00000019367	6.328854	3.445059	2.851806	5.668419	2.864328	2.650174
Crip1	ENSRNOG00000027990	66.3543	50.31524	38.0836	47.71817	43.6615	27.45974
Tsks	ENSRNOG00000020443	0.293715	0.546674	0.46574	0.187326	0.454654	0.243099
Nacc2	ENSRNOG00000018231	19.05099	10.62951	13.35243	10.00218	6.633107	9.317415
Gtf3c6	ENSRNOG00000000586	9.313789	13.57573	11.67102	8.425142	10.64907	9.231533
Ahcy	ENSRNOG00000017777	24.80079	20.98653	16.27076	22.25855	16.60456	14.72944
Luzp1	ENSRNOG00000022402	8.227747	6.453888	7.543895	7.539032	5.19101	7.078657
Rab20	ENSRNOG00000023991	12.22893	17.91115	15.09298	10.85658	17.36088	14.41643
Hsd17b11	ENSRNOG00000002210	29.70653	51.95443	52.29975	25.92506	49.22163	45.09927
Obsl1	ENSRNOG00000015346	13.59434	11.62068	13.90339	11.39998	10.84614	11.34912
Ddit3	ENSRNOG00000006789	220.947	490.6269	364.3576	159.218	460.2396	270.4824
Gpkow	ENSRNOG00000022939	12.82128	13.079	13.4352	10.92283	11.70771	9.814935
Apobec4	ENSRNOG00000028089	0.031148	0.094209	0.087558	0	0	0.027929
Cbx1	ENSRNOG00000008689	82.27241	66.57704	70.91591	77.95994	52.58453	57.66496
Mrps14	ENSRNOG00000002569	26.36558	25.56859	18.8839	22.91207	23.26197	17.77749
Pcmt1	ENSRNOG00000014330	76.57348	79.67605	50.69433	64.50661	72.42899	46.58352
Extl1	ENSRNOG00000016776	0.22285	0.588424	1.024163	0.146641	0.339733	0.789754
Ubiad1	ENSRNOG00000009575	8.498029	6.698886	5.942428	7.015628	5.777918	5.4491
Hint1	ENSRNOG00000000622	209.3235	206.5467	174.1725	201.1732	186.1746	164.2107
Med9	ENSRNOG00000053961	13.28496	12.50216	10.22432	12.23859	10.53428	9.49194
Sh2b1	ENSRNOG00000049181	8.627435	9.171568	10.49836	6.859336	6.7455	9.752632
Ube2d1	ENSRNOG00000000611	1.351523	1.362564	1.77895	0.444669	1.089058	1.067567
Nppb	ENSRNOG00000008141	7.684678	6.368675	2.623915	3.479709	3.216716	1.343046
AC098008.1	ENSRNOG00000018503	3.071129	5.07436	2.797701	1.728978	2.028823	1.453345
Prpsap2	ENSRNOG00000002724	21.6911	26.7226	23.69699	19.34583	25.88012	20.88114
RGD1559962	ENSRNOG00000026462	8.89165	5.112448	2.603574	5.055578	2.287443	1.432583
Gtf2f1	ENSRNOG00000047134	54.77442	55.32446	52.58623	45.04874	52.38124	43.54967
Hscb	ENSRNOG00000037508	16.46869	17.01947	15.56004	12.3401	13.8889	14.31407
Mcoln2	ENSRNOG00000015089	0.016989	0.051383	0.031837	0	0	0
Sdhd	ENSRNOG00000022980	83.18669	60.86546	59.79525	49.3383	48.13473	42.19941
Gtf2f2	ENSRNOG00000029316	18.95012	25.74748	17.01988	16.56657	19.32399	13.63113
Dpysl3	ENSRNOG00000018992	71.74197	64.47067	53.77445	57.30244	31.63535	39.38805
LOC102546764	ENSRNOG00000056435	37.54229	43.47229	30.55371	34.98061	35.12458	24.62137
Fam96a	ENSRNOG00000017119	40.0422	37.06775	32.11463	26.80919	31.55445	25.99336
Mrpl41	ENSRNOG00000029875	45.00511	43.8708	36.17861	42.31817	40.88195	29.72244
Fundc1	ENSRNOG00000003470	5.304677	6.9042	5.255651	4.846808	6.124846	4.990322
Apba3	ENSRNOG00000020466	12.94416	13.23634	13.57251	10.10755	12.34736	11.66156
Cabin1	ENSRNOG00000001237	7.928426	7.609393	7.878668	6.587286	7.146095	6.312485
Swap70	ENSRNOG00000009910	16.20246	18.69115	17.59377	13.65731	16.35911	16.83607
Wdr7	ENSRNOG00000018387	9.513015	10.15968	12.52793	7.009849	9.421884	10.37585
Sirt6	ENSRNOG00000006393	18.70329	22.6432	24.46594	15.02799	21.24054	19.73792
LOC102555083	ENSRNOG00000050661	0.109068	0.082469	0.025549	0.025119	0	0
Rmi1	ENSRNOG00000019108	25.15032	16.58905	12.67016	19.1171	12.79701	10.7185
Cd80	ENSRNOG00000001527	5.992443	4.249881	4.551116	4.838359	3.630083	4.13382
Fstl3	ENSRNOG00000009311	12.43467	6.791444	8.343928	11.73157	6.372764	8.108697
Bcl2l11	ENSRNOG00000016551	3.345476	5.252311	7.784895	2.925132	4.453861	6.495526
Zfp777	ENSRNOG00000033639	6.64782	7.90584	6.546644	4.820614	6.175093	6.00037
Psme3	ENSRNOG00000051344	7.224253	7.738478	6.596733	6.223889	4.634759	4.648259
Atp13a2	ENSRNOG00000008052	3.127948	4.827929	4.841562	2.694576	3.401263	3.416877
Zfp541	ENSRNOG00000021800	0.037915	0.009556	0.017763	0.008732	0	0
LOC103694404	ENSRNOG00000029267	1.122143	0.282828	0.525722	0.25844	0	0
Spry4	ENSRNOG00000013851	0.855764	0.584447	0.913933	0.59339	0.479757	0.561047

Dnajc7	ENSRNOG00000017781	23.7557	21.56858	26.50229	16.00437	19.26523	20.77531
Coprs	ENSRNOG00000000111	14.27074	16.14578	13.74305	12.92762	11.53045	9.881163
Lysmd2	ENSRNOG00000010642	9.419638	8.735885	7.936703	7.287489	8.10401	6.350906
Trim72	ENSRNOG00000022099	0.036607	0.055359	0.102902	0	0.016742	0.016412
Ndufaf8	ENSRNOG00000042653	39.3196	40.22522	34.94135	35.33268	37.29087	27.11306
Enpp4	ENSRNOG00000010174	3.870058	13.97785	12.86783	1.722045	8.798234	5.68517
mrpl11	ENSRNOG00000019970	21.13824	19.11559	15.77548	20.20724	16.12869	13.8103
Slc25a32	ENSRNOG00000004403	6.314244	5.907032	5.809818	4.821226	5.4634	4.369479
Cdc42ep1	ENSRNOG00000008517	5.098986	4.682242	5.295062	4.532816	3.11924	4.470046
Clca1	ENSRNOG00000060831	0.023967	0.036244	0.011228	0	0	0
Fbxo34	ENSRNOG00000011704	2.96412	3.293811	3.024245	2.609294	2.301558	2.492385
Slc35f6	ENSRNOG00000009459	23.03424	26.30099	25.48966	18.01468	24.84266	21.53544
AABR07071891.2	ENSRNOG00000037673	87.05559	89.28823	102.7809	77.0708	61.85708	68.33225
Olr1	ENSRNOG00000056219	5.702247	6.204534	3.420811	4.057288	4.060271	2.795456
Psmc5	ENSRNOG00000010038	84.63905	82.00775	66.18951	68.39428	73.78246	60.10956
Wdr60	ENSRNOG00000004520	3.179836	3.68732	3.839188	2.477817	3.379923	3.538614
Pkia	ENSRNOG00000012095	5.795178	2.932455	2.787846	4.397806	2.274677	2.229789
Dus4l	ENSRNOG00000008155	1.665798	1.467443	1.257769	1.191918	1.287003	0.638054
AABR07045462.1	ENSRNOG00000000320	0.174821	0.17625	0.109205	0.161052	0.159908	0.104501
Rcc1l	ENSRNOG00000001483	8.305372	8.338264	8.204509	6.22959	6.724574	7.60242
Ambra1	ENSRNOG00000017422	7.371145	7.898433	8.154749	6.638145	6.477484	7.632944
Nol11	ENSRNOG00000052934	21.31291	19.78691	15.85805	18.24917	15.22041	14.46462
AABR07018323.1	ENSRNOG00000038483	4.094557	4.249421	5.303511	3.57236	2.555591	3.520181
Stmn3	ENSRNOG00000013657	0.231962	0.272834	0.434696	0.142462	0.070725	0.138658
Cks1b	ENSRNOG00000042561	45.37234	28.45194	17.22009	28.25932	19.75324	10.87971
Cdc37	ENSRNOG00000033426	42.33111	47.87894	40.14344	36.49234	29.56141	28.79457
Nmral1	ENSRNOG00000003794	20.60521	14.5016	10.81596	15.50385	11.24926	9.221633
Mcm2	ENSRNOG00000016316	62.1494	30.75355	21.49027	53.81732	20.02126	18.39878
RGD1565616	ENSRNOG00000021237	12.61973	12.38294	11.28676	7.462312	7.375967	9.771269
Tcp11	ENSRNOG00000000499	0.091604	0.11544	0.021458	0.021097	0.041895	0
Tepsin	ENSRNOG00000028161	6.459512	5.50917	5.475399	4.393945	3.871565	4.885567
Snrpd1	ENSRNOG00000013714	59.96453	43.43174	27.99468	47.43988	33.48684	24.43086
Dusp4	ENSRNOG00000011921	0.990624	0.898846	0.701315	0.8112	0.59401	0.602027
Hgfac	ENSRNOG00000009572	0.128653	0.021617	0.040183	0.079013	0	0.019226
Ss18	ENSRNOG00000016800	36.96349	31.51509	30.23416	26.27269	26.37064	26.12921
Ets1	ENSRNOG00000008941	16.19714	15.95236	13.38349	11.84416	13.21611	12.02714
Ccdc34	ENSRNOG00000005902	6.175336	3.278566	1.977379	4.175082	1.835379	1.3959
Aaed1	ENSRNOG00000018886	8.389911	10.0587	10.62339	7.520201	7.881603	7.624411
Shc4	ENSRNOG00000037134	0.515141	0.448529	0.351044	0.301997	0.364106	0.251944
Elavl1	ENSRNOG00000001069	46.2577	41.57046	36.76948	43.83752	39.48863	36.08692
Cstf2	ENSRNOG00000048025	65.46521	42.52267	20.2668	56.15116	32.11154	17.31808
Nfe2l2	ENSRNOG00000001548	143.9058	192.5685	154.338	128.9888	184.9165	148.9329
Sdccag8	ENSRNOG00000004181	7.095511	7.601573	7.079796	5.103556	5.894915	6.518701
Vegfb	ENSRNOG00000021156	5.929893	10.62267	10.14825	5.372541	8.76362	8.217174
Cbx5	ENSRNOG00000036841	43.73384	34.823	26.31539	35.87659	28.70966	24.08837
Csrnp1	ENSRNOG00000033433	7.662312	7.724911	4.652903	5.117633	5.836059	3.92403
Pdlim1	ENSRNOG00000016166	309.5582	158.8781	136.6505	268.0467	144.5552	114.173
Bms1	ENSRNOG00000006576	15.338	12.59857	12.21443	13.45162	11.37352	11.64191
Hsf1	ENSRNOG00000021732	11.83338	15.52918	12.4632	10.36713	13.45944	11.86146
Alpk2	ENSRNOG00000017421	0.050056	0.100931	0.060303	0.019763	0.013082	0.012824
Fchsd1	ENSRNOG00000039415	3.333758	4.799112	4.340165	2.561778	4.420113	3.111344
Mrpl2	ENSRNOG00000018057	21.81969	27.80007	23.27699	20.67324	27.3054	22.79427
Zfp689	ENSRNOG00000018877	4.80954	4.729776	5.10504	4.628525	4.08011	4.548275
Dbi	ENSRNOG00000046889	51.37021	45.16967	29.46492	37.74283	35.72131	25.48974

Cyp19a1	ENSRNOG00000000196	0.764296	0.801362	0.315103	0.563278	0.517331	0.233002
Atp1b4	ENSRNOG00000007059	0.114589	0.259932	0.393689	0.061579	0.148487	0.351048
Isg15	ENSRNOG00000021802	278.2352	903.0393	827.7405	118.1885	808.1906	542.5256
Tmem129	ENSRNOG00000017329	25.862	22.98368	17.40404	22.87822	21.77563	16.06808
Dusp8	ENSRNOG00000029394	1.607642	1.058007	0.899628	1.41931	0.79652	0.380391
Deaf1	ENSRNOG00000017960	7.514126	6.326803	7.098732	7.321652	6.004537	6.99662
AC119762.3	ENSRNOG00000058654	2.481281	1.407123	2.615568	1.885824	0.964584	2.447305
Tcp1	ENSRNOG00000014160	102.4978	82.26401	55.69892	88.84662	68.50596	51.74628
Shmt1	ENSRNOG00000005275	15.72152	12.09773	11.96518	11.66542	10.97604	8.53467
Map3k1	ENSRNOG00000013177	2.345234	2.227823	2.332337	2.121517	1.85863	2.216709
Serpinb6b	ENSRNOG00000016420	8.048476	5.266448	6.634971	3.77857	4.034879	3.642999
Lamtor5	ENSRNOG00000018186	29.12594	42.11334	33.16892	26.28149	31.87847	25.08879
LOC103690024	ENSRNOG00000010407	0.490152	0.776532	0.734833	0.38704	0.691723	0.514833
Zkscan3	ENSRNOG00000055000	8.740765	10.69512	10.37408	8.052314	8.910796	9.538783
LOC100911727	ENSRNOG00000047206	32.08279	15.71341	10.81359	28.4104	12.09397	9.766894
Prmt5	ENSRNOG00000012046	24.12652	22.50972	17.8379	17.44318	19.99945	14.62677
Dph2	ENSRNOG00000019735	7.189007	7.923377	5.175737	5.799608	5.01539	4.078807
Cxxc1	ENSRNOG00000014614	13.42083	17.22825	17.10548	11.62655	14.19739	11.67492
Tmem167b	ENSRNOG00000042160	23.105	26.85634	26.04282	20.65914	25.36078	25.28684
LOC103689949	ENSRNOG00000021731	7.188875	6.006889	4.120317	6.830046	5.216562	3.808301
Sod1	ENSRNOG00000002115	478.3974	542.8378	438.7549	307.3529	474.993	361.5372
Dpf1	ENSRNOG00000020687	2.310211	1.59655	1.745692	1.887965	1.039525	0.601384
Tnip1	ENSRNOG00000010370	67.43321	68.67737	60.09198	56.23776	65.62221	50.33829
Isy1	ENSRNOG00000010021	34.62028	35.85774	30.77552	26.03271	32.37064	27.00922
H2afx	ENSRNOG00000010386	58.86696	22.82356	15.16925	57.74675	22.45306	13.80787
Ero1b	ENSRNOG00000002609	4.393935	4.743328	4.015754	3.00164	3.747039	3.624606
Pkd1	ENSRNOG00000010771	23.51822	20.21593	22.27693	20.95659	16.72733	21.30902
Med11	ENSRNOG00000019384	16.1336	13.68437	16.38441	13.03838	12.5923	12.38712
Ankrd34a	ENSRNOG00000033741	1.054728	0.5702	0.816402	0.661852	0.461403	0.438592
Abcb9	ENSRNOG00000001082	0.056376	0.097433	0.030185	0.022258	0.0442	0.014443
Synpo	ENSRNOG00000019181	23.64774	16.37447	16.51245	20.29905	11.62565	15.17672
Relt	ENSRNOG00000025075	3.823389	1.385754	1.199101	3.405809	1.271719	0.878303
Daw1	ENSRNOG00000016247	0.182893	0.237069	0.53859	0.048139	0.167291	0.491968
Zfand3	ENSRNOG00000059659	30.51623	29.24941	37.38064	27.73909	25.641	29.86202
Stoml2	ENSRNOG00000009535	60.40322	57.58932	50.89344	45.80019	53.27054	41.57531
Rgs2	ENSRNOG00000003687	213.1206	188.1248	102.7047	65.86288	86.28639	61.03043
Mdc1	ENSRNOG00000032813	7.671878	4.27459	2.767945	6.203174	2.73385	2.344914
Casp7	ENSRNOG00000056216	10.2073	10.63553	9.142498	6.384987	9.484748	6.779485
Grip2	ENSRNOG00000009726	0.033801	0.017038	0.055425	0	0.007729	0.02273
Isyna1	ENSRNOG00000019741	4.470592	3.623368	3.141694	4.098285	2.926598	2.908145
Mob1b	ENSRNOG00000010996	8.270412	8.109542	6.960758	6.664768	6.750663	6.530376
Hexim1	ENSRNOG00000003203	26.86765	28.8428	33.04202	23.83475	24.27225	24.2208
Ttc22	ENSRNOG00000007189	1.1828	0.949554	0.841471	1.140085	0.791389	0.697213
Eps8l3	ENSRNOG00000048209	0.048322	0.081195	0.015092	0.014839	0.029467	0
AABR07044408.1	ENSRNOG00000051805	1.013046	1.021322	3.037503	0	0.555976	1.453345
Cdc7	ENSRNOG00000002105	8.461661	3.94776	2.516327	7.574053	2.566217	2.112008
Armc7	ENSRNOG00000042691	4.524084	5.715269	4.463977	4.099708	4.763093	4.089601
Snx8	ENSRNOG00000001258	21.10418	26.80649	26.23024	19.59601	21.56769	20.67726
Epha2	ENSRNOG00000009222	30.59984	28.35794	18.53824	19.49376	19.0893	15.57378
Fbxw17	ENSRNOG00000030027	32.07753	58.28759	52.08908	25.49754	56.43183	45.32785
Cgnl1	ENSRNOG00000054080	0.137786	0.343973	0.319689	0.102756	0.23406	0.188259
Gng12	ENSRNOG00000050231	63.01462	88.86632	68.9625	58.33651	72.19033	51.95039
Actr8	ENSRNOG00000015280	14.93587	14.86457	15.79162	13.22951	14.12947	13.2202
Clcn6	ENSRNOG00000008345	3.531712	3.801256	4.296559	3.238375	2.876515	3.771971
Bcr	ENSRNOG00000001304	10.16913	9.485585	7.986444	7.078413	6.810125	7.164205
Atp5mc3	ENSRNOG00000001596	105.551	101.9162	85.8534	92.23956	85.96027	81.61551

Rab30	ENSRNOG00000010224	22.83793	23.24484	19.83763	18.57283	19.99014	18.68936
Elp5	ENSRNOG00000027628	21.14732	23.05252	19.86689	17.84758	22.15734	16.70012
Pomt2	ENSRNOG00000012146	6.498071	5.616539	6.441037	5.535077	5.231771	6.022493
Prr30	ENSRNOG00000025726	0.110125	0.08882	0.185736	0.04058	0.020146	0.019749
Cfdp1	ENSRNOG00000019326	26.85441	30.92838	28.23314	24.50865	29.58033	24.0176
Ube2j1	ENSRNOG00000007434	37.15399	34.36131	35.34486	35.45772	32.70205	31.30843
AABR07044362.3	ENSRNOG00000058490	1.23626	0.997088	1.15837	0.797222	0.565399	0.110848
Tbx15	ENSRNOG00000019565	0.655257	0.53357	0.436865	0.325041	0.437993	0.225974
Plekhg4	ENSRNOG00000016479	0.236134	0.102027	0.252865	0.082871	0.061712	0.120988
Timm10	ENSRNOG00000007883	10.85801	16.92146	13.51344	9.544657	14.17738	9.066888
Gclc	ENSRNOG00000006302	41.40654	25.21207	30.34062	40.60792	23.17679	29.44634
LOC100912489	ENSRNOG00000054017	137.4366	92.80416	56.79499	105.3645	75.48914	46.35564
LOC361635	ENSRNOG00000016063	20.58028	27.50053	27.84566	19.15914	22.08608	23.47368
LOC108348098	ENSRNOG00000047683	2.490611	2.869667	2.166999	2.185183	2.278144	1.967332
Ankfy1	ENSRNOG00000016212	18.85595	22.5736	21.55672	16.04581	19.11485	20.6502
Ska2	ENSRNOG00000025981	28.04698	19.25893	14.20908	22.88791	16.96825	12.21103
Pacsin3	ENSRNOG00000014204	21.44561	25.87169	25.27132	18.6403	24.40382	20.46753
LOC367195	ENSRNOG00000031912	0.72422	1.147358	0.58165	0.190622	0.189268	0.2783
Star	ENSRNOG00000015052	4.095726	3.288056	4.619427	1.676948	2.566931	3.162347
Hist1h2ah	ENSRNOG00000049649	68.37365	30.87356	15.12955	65.75817	20.88092	7.189063
Cep164	ENSRNOG00000029826	16.01132	12.63075	14.0566	13.52258	11.73666	10.69943
Ift27	ENSRNOG00000006440	11.87318	12.05661	11.04479	9.516462	11.09555	7.571342
Irak2	ENSRNOG00000021817	4.429874	5.350286	4.039343	3.519488	5.112559	3.225596
Tead2	ENSRNOG00000020695	5.625037	4.021981	5.476228	4.759347	3.667311	5.11519
Scap	ENSRNOG00000020853	24.09709	25.31523	28.54238	21.57699	23.47343	23.34968
Runx1	ENSRNOG00000001704	9.482755	11.60467	12.10645	7.614968	9.398098	11.53312
Zbtb48	ENSRNOG00000009595	6.277321	4.915615	5.308438	6.001122	3.863993	4.336434
Sart1	ENSRNOG00000020475	20.98254	21.48067	19.17473	16.22517	18.94063	17.62254
Sytl2	ENSRNOG00000049878	1.4317	2.028558	1.559037	0.784232	1.415747	1.387809
Hoxc9	ENSRNOG00000016199	0.095658	0.192879	0.179263	0.044062	0.131247	0.042886
Pusl1	ENSRNOG00000022354	10.4199	10.93823	9.64262	9.262731	10.43498	9.188819
Rcan3	ENSRNOG00000018576	7.401195	5.555694	7.990198	5.336025	4.635856	4.760772
Gmeb1	ENSRNOG00000010910	3.113839	3.320025	2.493268	2.851198	2.753266	2.284364
Mtdh	ENSRNOG00000006870	45.31326	46.13832	40.62031	44.38263	42.64989	37.16484
Fpgt	ENSRNOG00000009548	6.758014	8.379168	8.000398	6.342882	7.627183	6.662218
Eif4enif1	ENSRNOG00000018475	8.088719	6.901153	7.375477	6.906656	6.559439	6.051125
Sh3pxd2b	ENSRNOG00000004063	5.357185	7.548511	11.57352	4.642344	5.728993	10.79001
Pcca	ENSRNOG00000057042	4.506504	5.777067	5.769639	3.221036	3.820019	5.225074
Pole3	ENSRNOG00000055277	19.69024	14.02155	9.856675	15.46817	9.868782	8.736863
Pum1	ENSRNOG00000011709	20.06239	23.84548	22.17734	18.04628	20.10468	20.98083
Lysmd4	ENSRNOG00000050624	7.504227	9.406705	9.333755	7.127295	8.321918	7.889742
Ccdc71	ENSRNOG00000048718	7.770106	10.52554	8.58155	6.813732	6.789755	5.458688
Casp8ap2	ENSRNOG00000006487	9.297759	7.619888	6.264156	9.238202	7.478178	6.207325
Ppp2r2b	ENSRNOG00000018851	0.379776	0.2027	0.230255	0.308703	0.183906	0.160246
LOC100911166	ENSRNOG00000046797	0.62655	0.478537	0.320223	0.349819	0.347334	0.221312
Rpp25l	ENSRNOG00000059653	19.52086	23.80219	19.53195	17.24066	21.96396	18.94894
Cracr2b	ENSRNOG00000019199	9.178466	8.16645	7.797154	8.761163	7.409262	7.560513
Wipf2	ENSRNOG00000027849	1.850131	1.404691	2.033193	0.883767	0.689456	1.781788
Ddx55	ENSRNOG00000001043	4.482826	5.178536	3.727845	4.319024	4.630046	3.081612
Polr2g	ENSRNOG00000019439	72.25526	66.92747	54.5111	62.03573	59.32509	51.86396
Rhbdl1	ENSRNOG00000019921	2.08398	1.459032	2.41373	1.066578	1.191376	1.686918
Dis3l2	ENSRNOG00000018931	4.997027	5.444364	5.869608	4.192187	5.189838	4.867915
Napepld	ENSRNOG00000011363	2.358012	2.348976	2.814416	2.068846	1.694674	2.567358
Majin	ENSRNOG00000054336	0.168754	0.510399	0.606134	0.051821	0.077179	0.151312
Pttg1ip	ENSRNOG00000001223	45.04024	60.90466	72.50666	38.45056	48.67155	50.82466
Rps19bp1	ENSRNOG00000017847	27.05477	27.30105	22.51004	20.42371	23.83026	20.37261

Smarcb1	ENSRNOG00000028302	0.777915	0.78427	0.86143	0.564628	0.646866	0.443871
Chordc1	ENSRNOG00000026643	27.0541	30.60036	27.75045	22.16096	29.20924	24.74669
Tomm5	ENSRNOG00000062108	3.493324	3.060667	2.62378	3.026621	2.510608	2.485924
Ctu2	ENSRNOG00000051531	12.47477	11.29848	8.676449	9.969658	8.759852	8.018583
Cntrob	ENSRNOG00000008270	9.385508	5.45483	3.400583	7.250135	4.158671	2.789256
LOC100909715	ENSRNOG00000061540	13.10536	12.85372	9.390331	11.52684	11.33648	8.985923
Mrpl35	ENSRNOG00000008546	22.80751	23.72424	19.91232	16.24036	22.06449	14.77225
Mycbpap	ENSRNOG00000042912	0.77604	0.884988	1.478134	0.562558	0.605108	0.878344
Psmb4	ENSRNOG00000020979	369.0955	383.2596	321.4033	321.5239	368.1924	296.1029
Srgap2	ENSRNOG00000006733	10.15218	12.78371	11.52118	6.427559	9.554354	10.5898
Plin1	ENSRNOG00000015086	0.204708	0.368536	0.438423	0.175114	0.294242	0.327767
Ift122	ENSRNOG00000010952	8.509587	13.04949	10.30759	7.077441	11.41117	9.899421
Sp100	ENSRNOG00000022769	36.31905	100.9103	108.8663	27.86882	90.56991	85.85022
Zfp524	ENSRNOG00000016565	6.716814	6.93993	6.371808	6.264643	5.266133	5.274435
Paf1	ENSRNOG00000019746	17.9938	20.07504	17.05078	16.29536	13.37444	12.0158
Mphosph6	ENSRNOG00000050087	16.89555	17.28285	15.13603	15.83054	13.4565	12.74756
Pnpo	ENSRNOG00000046493	7.322396	8.824226	7.941219	3.795663	7.576456	6.29759
Nudt9	ENSRNOG00000002186	16.99648	23.81844	20.40012	15.19471	19.88581	18.98634
Ppm1f	ENSRNOG00000037909	15.37614	14.37094	15.25693	10.5829	12.58533	13.14994
Ebi3	ENSRNOG00000050509	0.617924	2.272018	1.05581	0.401827	1.396404	0.325916
Dhx37	ENSRNOG00000022171	6.430653	6.389381	5.889872	4.171671	4.142038	5.311763
Cotl1	ENSRNOG00000016257	2.099562	1.42947	1.737338	1.783477	1.247048	1.149093
MGC105567	ENSRNOG00000038955	13.27544	33.18956	21.36268	10.19785	32.43148	18.61281
Tnf	ENSRNOG00000000837	1.660267	1.909213	1.507048	1.147124	1.708463	0.744333
AABR07048271.1	ENSRNOG00000033271	0.090049	0.363137	0.3375	0	0	0
AABR07026557.1	ENSRNOG00000053136	0.986222	0.434997	0.462043	0.454272	0	0.331608
Ppm1g	ENSRNOG00000026905	43.41915	28.90342	25.07839	33.78384	24.62972	21.54583
Sart3	ENSRNOG00000000702	10.48382	10.05616	8.684834	8.741311	5.715582	6.930863
Ugdh	ENSRNOG00000002643	154.069	154.0149	152.7613	89.03039	132.4848	120.5806
Cry1	ENSRNOG00000006622	3.474727	2.597137	2.11908	2.455975	1.959044	1.839809
RGD1560795	ENSRNOG00000028235	5.221792	5.932955	3.805509	4.581435	3.487484	2.155244
AABR07066522.1	ENSRNOG00000049511	6.075199	7.167356	6.747872	4.763146	4.526627	5.877767
Cnih1	ENSRNOG00000009811	61.89465	77.25025	72.50801	57.29942	66.19853	55.38846
Map3k5	ENSRNOG00000031700	5.998055	5.460483	5.56068	5.779009	5.041276	4.818485
Mtif3	ENSRNOG00000047329	6.063461	7.669548	6.60204	5.353138	7.266555	6.393225
Exosc2	ENSRNOG00000009245	15.86872	10.40623	6.086166	11.92318	5.500115	4.887018
Scrn2	ENSRNOG00000010214	10.96288	15.54435	13.2934	9.316301	14.85382	10.72341
Itpa	ENSRNOG00000021233	68.29326	55.05872	51.4474	59.28916	50.18918	48.70414
AABR07029596.1	ENSRNOG00000046195	0.386068	0.500429	0.69765	0.127022	0.403582	0.321439
Nfkbie	ENSRNOG00000019907	22.61938	24.97994	22.98605	18.5225	23.23853	16.53384
Gmnn	ENSRNOG00000018782	25.11391	16.96678	6.579109	21.3904	12.64113	5.529618
Dhx34	ENSRNOG00000047575	1.977507	2.53739	1.93714	1.324911	1.3155	1.561553
Klrk1	ENSRNOG00000061739	10.16445	55.31746	41.71178	3.439196	50.50411	27.56667
Ndufb8	ENSRNOG00000014078	94.41762	89.181	85.26983	89.15408	84.94361	83.99095
AABR07035832.1	ENSRNOG00000055795	21.35133	21.79316	12.17763	16.12665	11.76647	9.037161
Tprn	ENSRNOG00000010896	2.403712	2.859553	2.702722	2.007712	2.330578	2.571957
Psmc3	ENSRNOG00000011414	99.31589	114.0203	77.90188	92.48526	91.36054	65.56944
Pak1ip1	ENSRNOG00000023799	33.65777	28.9099	20.23791	25.34784	24.31333	17.76728
LOC100365112	ENSRNOG00000036571	0.927283	0.723989	1.659328	0.597331	0.644106	1.362814
Serpinb9	ENSRNOG00000033772	386.1133	426.677	301.5155	219.3288	376.2351	211.3446
Tsen54	ENSRNOG00000004598	6.191918	6.666103	7.894593	5.194917	5.724386	5.472624
Lif	ENSRNOG00000007002	10.4199	12.68532	8.215997	8.711725	7.804639	5.975949

Slfn2	ENSRNOG00000037113	146.4055	367.1615	332.9563	114.7158	349.9258	275.1693
Ranbp1	ENSRNOG00000001884	106.6217	68.23676	50.28339	98.07059	59.7029	48.15516
Ftsj1	ENSRNOG00000004776	14.03188	18.76416	15.82699	12.58523	17.94459	13.12758
Bbs9	ENSRNOG00000015189	2.845151	2.907509	3.671663	2.239813	2.75622	3.0613
Susd6	ENSRNOG00000045941	21.45138	25.50403	25.79806	17.70248	16.57344	22.4088
Spry2	ENSRNOG00000010058	9.671511	9.18987	5.663853	7.150076	7.276216	5.051376
AABR07003173 .1	ENSRNOG00000037360	0.218599	0.308539	0.450619	0.120829	0.119971	0.392011
Rangap1	ENSRNOG00000031789	92.64765	51.3061	34.69332	84.97802	40.92042	32.15208
Arhgef39	ENSRNOG00000021433	6.038653	3.144661	1.844481	5.30897	2.531248	1.671252
Kbtbd2	ENSRNOG00000013809	15.44597	15.10962	12.80103	11.37648	12.99759	11.54127
AABR07018078 .1	ENSRNOG00000053891	34.08411	112.1542	80.50099	19.73143	105.2403	55.8905
Ano3	ENSRNOG00000004731	0.190563	0.162563	0.2541	0.094529	0.120674	0.098577
B4galt5	ENSRNOG00000008283	32.03581	47.88845	57.40882	29.57877	43.54029	49.26623
Nemp1	ENSRNOG00000046492	6.871361	5.927272	3.339724	4.569895	4.302159	2.767578
Tigd2	ENSRNOG00000038449	6.36662	8.902366	7.910766	4.615629	7.798429	7.445978
Mcam	ENSRNOG00000007726	1.778885	1.200678	0.988784	0.694396	0.785988	0.540686
Cfap70	ENSRNOG00000007046	0.436545	0.539136	0.971471	0.341837	0.479165	0.782849
Arhgap26	ENSRNOG00000013920	0.33352	0.467818	0.285332	0.160305	0.252013	0.234038
RGD1560925	ENSRNOG00000011042	0.05868	0.035496	0.054983	0.043247	0	0.042092
Trove2	ENSRNOG00000003434	14.2111	16.90301	13.42606	12.61454	15.59424	13.04843
AABR07008298 .1	ENSRNOG00000054825	1.167029	3.039456	2.004752	0.895925	2.757639	1.30801
Ppif	ENSRNOG00000010558	2.925453	4.081428	3.128772	2.341141	3.162416	2.888044
Dhrs9	ENSRNOG00000058568	1.74586	1.019583	0.628411	1.60835	0.62319	0.448624
Cd3g	ENSRNOG00000015945	0.567437	0.214527	0.066461	0.457401	0	0
Tubg1	ENSRNOG00000020213	39.01242	23.47095	21.17092	31.46545	21.2706	17.01675
Acad9	ENSRNOG00000014178	22.01411	20.20968	20.65812	19.74692	19.31921	19.75362
Fam156b	ENSRNOG00000048516	0.569282	0.43045	0.466738	0	0.06509	0.319027
Pdcl3	ENSRNOG00000013286	25.77417	23.36095	19.25178	21.80926	19.77498	18.32033
Lcmt2	ENSRNOG00000043002	5.418938	5.089302	5.038909	5.143977	4.202799	3.879691
Aasdhppt	ENSRNOG00000005795	39.80733	43.09314	37.22286	38.25007	40.44062	32.10903
Pold3	ENSRNOG00000018411	13.65196	7.948367	5.719149	9.680865	5.78773	4.560705
Synj2bp	ENSRNOG00000006399	8.269217	9.232712	8.834948	7.187752	6.909368	8.056046
Selenoh	ENSRNOG00000054563	71.26639	58.28846	36.30374	58.25568	45.90625	33.24026
Brsk1	ENSRNOG00000017673	1.263561	1.411601	1.279947	1.006736	1.015203	1.178893
Tmprss5	ENSRNOG00000008058	0.277453	0.111888	0.138652	0.2556	0.050757	0.049755
Snrnp70	ENSRNOG00000020763	33.64765	31.6865	40.567	31.99416	25.77968	33.40357
Dusp18	ENSRNOG00000024945	4.01973	4.991581	5.121481	3.736994	3.990694	4.560271
Mtfr1	ENSRNOG00000021359	17.11953	16.47299	11.07862	14.97691	10.89003	8.83459
Tbxas1	ENSRNOG00000007918	0.312438	0.697478	0.815526	0.226151	0.38785	0.440228
Nup107	ENSRNOG00000006541	28.21451	13.05393	10.64892	25.06767	10.99022	9.860774
Pes1	ENSRNOG00000004515	37.66487	51.37217	41.36099	32.64465	39.08768	36.75125
LOC102554838	ENSRNOG00000031595	0.524743	0.793545	0.147505	0.193365	0.623973	0.047051
Naxd	ENSRNOG00000015021	36.55273	35.1428	32.9109	27.94919	31.7628	29.55686
Hivep3	ENSRNOG00000009341	0.479347	0.366345	0.340482	0.238602	0.187404	0.284224
Cox16	ENSRNOG00000047115	30.91869	32.7757	27.42629	30.47309	30.82849	25.6336
LOC100911548	ENSRNOG00000015125	14.68407	22.1515	18.18746	13.62722	17.49054	14.13686
Egln2	ENSRNOG00000020947	44.09396	48.5177	50.04543	36.25859	46.67845	42.18283
Frem3	ENSRNOG00000039152	0.013272	0.01338	0.024871	0.006113	0	0
Pelp1	ENSRNOG00000019268	13.37857	11.8353	10.01724	9.937589	7.805402	9.110321
AABR07000747 .1	ENSRNOG00000054246	0.69466	1.120537	1.171608	0.511957	0.508321	0.373717
LOC108348151	ENSRNOG00000046980	1.347792	0.959155	1.708596	0.730374	0.50763	0.355438
Tbpl1	ENSRNOG00000011114	32.76157	35.03633	30.79563	23.8103	30.2543	28.21669
Supt16h	ENSRNOG00000011953	46.05361	34.66577	30.16977	41.716	29.11482	28.91174

Mrps6	ENSRNOG00000059381	35.31139	30.55025	19.1832	29.70396	28.86301	16.33609
C1rl	ENSRNOG00000011718	8.030534	9.366497	10.62604	6.54907	8.360421	10.26959
Ola1	ENSRNOG00000019047	42.93198	45.11674	37.25631	41.84885	44.66558	36.86352
Btf3	ENSRNOG00000016912	162.5249	185.417	134.8428	135.9553	146.0408	125.3443
Plin4	ENSRNOG00000047046	4.318844	6.652433	11.6111	2.100126	4.610413	6.132177
Xpr1	ENSRNOG00000000042	4.598927	5.491897	5.173315	3.670297	3.509254	4.531544
RT1-CE4	ENSRNOG00000039744	50.5927	81.20329	78.59391	48.13007	75.8408	69.37706
Fam53a	ENSRNOG00000017548	10.34224	10.38511	9.92275	9.470605	10.02644	8.514406
Zfp830	ENSRNOG00000007578	7.78616	8.601665	6.820407	7.255345	6.521628	5.536975
Arf2	ENSRNOG00000004807	20.76609	15.97035	12.5072	13.25986	13.36793	9.246796
Engase	ENSRNOG00000027498	3.006492	3.284561	3.861947	2.769691	2.710017	2.931014
Nfu1	ENSRNOG00000018410	28.01817	36.56205	31.76232	23.37565	34.72416	24.41462
Spa17	ENSRNOG00000010934	1.422155	2.54169	1.877689	1.191038	2.306026	1.275165
AABR07049128.1	ENSRNOG00000061730	0.537448	0.309622	0.143882	0	0	0
Spata9	ENSRNOG00000012770	0.633643	0.936935	0.989534	0.505904	0.463671	0.719658
Neil1	ENSRNOG00000018577	7.015344	8.604641	9.373546	5.926171	8.3628	8.37944
S1pr3	ENSRNOG00000014524	9.001442	3.73746	4.94961	4.953654	1.755047	3.706324
Abhd17c	ENSRNOG00000012683	10.07146	7.032628	8.984843	7.500338	6.417343	7.264053
Lpcat4	ENSRNOG00000005058	6.287872	4.451557	4.242031	4.91729	3.706504	3.85888
Slc39a11	ENSRNOG00000031213	10.23063	10.67225	11.44183	8.304924	9.882623	8.315907
Acvr1b	ENSRNOG00000006934	8.023971	10.09237	13.42057	6.204619	9.050239	9.8709
RGD1559896	ENSRNOG00000018836	32.40934	16.31493	19.13901	18.15664	12.27273	11.54512
Mapk14	ENSRNOG00000000513	34.12711	30.01395	34.23429	31.61164	29.37759	32.69279
Lrrc47	ENSRNOG00000024796	24.58204	22.08344	25.3862	20.15135	21.08749	21.06471
Kcnk5	ENSRNOG00000047005	0.189507	0.420323	0.355135	0.128026	0.150229	0.158592
Zfp41	ENSRNOG00000007489	5.410264	3.25735	3.098623	4.844065	2.654003	2.965176
LOC688981	ENSRNOG00000032000	0.999169	1.208798	1.497947	0.736377	0.913933	1.433436
Ppa1	ENSRNOG00000000557	127.9024	100.7039	103.0993	119.6371	96.93769	100.4007
Lyar	ENSRNOG00000005374	17.27445	11.5691	8.472946	11.81655	9.17032	6.631854
Slpi	ENSRNOG00000046699	6.339585	7.576876	14.03603	4.804085	6.827589	11.277
Gcgr	ENSRNOG00000036692	0.453979	0.320382	0.255226	0.146378	0.228388	0.101764
Rbmx2	ENSRNOG00000007371	6.491421	4.962279	3.475676	4.797252	3.360322	3.102127
Dlx1	ENSRNOG00000001520	0.111115	0.032007	0.104115	0.058493	0.014519	0.028466
Irx3	ENSRNOG00000011533	0.893704	1.773855	0.968241	0.617485	0.945194	0.601
RGD1561149	ENSRNOG00000008113	0.778772	1.083827	1.284917	0.709635	0.774279	1.05501
Hif1an	ENSRNOG00000014234	4.284776	6.06748	6.589203	3.856897	4.12868	4.721741
Eif4a3	ENSRNOG00000045791	113.4936	81.41819	81.02844	78.24114	69.17226	66.08067
Hsp90aa1	ENSRNOG00000007219	8.30753	7.073006	5.065265	7.213784	5.126489	4.544177
Cdc45	ENSRNOG00000050071	4.573542	1.663516	0.705678	4.087168	1.095947	0.583202
Helq	ENSRNOG00000002181	4.360503	4.407545	4.223733	3.568397	3.470531	4.021522
Ifit1bl	ENSRNOG00000036603	2.163812	12.53865	8.676165	1.454631	9.860457	5.401004
LOC100911224	ENSRNOG00000055843	11.58448	5.733387	4.802342	10.24086	4.302716	4.490602
Masp1	ENSRNOG00000001827	0.045714	0.107538	0.114224	0.028076	0.027876	0.054652
Rpl36al	ENSRNOG00000011494	144.8974	124.9109	145.3915	133.2438	120.9883	128.3962
Grm1	ENSRNOG00000014290	0.444043	1.541266	0.285303	0.134408	0.986395	0.136508
Pop1	ENSRNOG00000005243	6.307142	6.044507	4.50824	4.926185	5.187628	4.168793
Prnd	ENSRNOG00000021260	2.363397	1.725407	0.458171	0.90093	0.447265	0.146146
Brd9	ENSRNOG00000015676	12.77472	11.41433	10.87414	12.3235	10.43704	10.5647
Bin1	ENSRNOG00000012852	15.07836	23.73605	18.99461	14.03427	20.39516	17.43151
Ifit2	ENSRNOG00000036604	57.17895	238.1687	154.0653	29.83833	134.6749	95.79284
Ccdc73	ENSRNOG00000022660	0.174755	0.276858	0.280705	0.045997	0.228353	0.078346
Nans	ENSRNOG00000008945	102.4	79.75411	60.70854	80.06237	70.95205	52.48007
Tor1b	ENSRNOG00000006435	49.60473	62.72388	62.94088	44.26275	60.29196	53.58276
Rfc4	ENSRNOG00000001816	33.22037	20.47549	11.05749	25.18511	13.26446	9.353986
RGD1304567	ENSRNOG00000025034	30.49949	24.66299	24.70797	28.75578	23.90191	21.72104

AABR07051376.1	ENSRNOG00000027193	5.852186	5.777928	6.668408	5.230132	4.725366	4.547677
Anapc15	ENSRNOG00000019936	123.3936	95.87223	55.60002	113.1695	74.94384	49.37001
Mt-cyb	ENSRNOG00000031766	34.11492	30.76513	27.76804	27.08939	26.02141	26.1602
Zscan22	ENSRNOG00000031113	3.970487	3.587807	3.086495	3.30553	2.152164	2.637117

Supplementary Table 6. The significantly enriched metacore pathways for the 1848 genes up-regulated under AngII.

Maps	Total	p-value	FDR	In_Data	Network Objects from Active Data
Transcription_Negative regulation of HIF1A function	69	6.67E-12	9.37E-09	23	Ubiquitin, RACK1, MCM3, ING4, HIF-prolyl hydroxylase, EGLN2, MCM7, ARD1, Sirtuin3, VCP, MCM2, PSMA7, Sirtuin6, Elongin B, HSP90, FHL2, CHIP, PRDX2, VHL, HIF1A, COMMD1 (MURR1), LAMP2, EGLN1
Regulation of degradation of deltaF508-CFTR in CF	39	3.09E-10	2.17E-07	16	Ubiquitin, RNF4, BAG-2, UFD1, USP19, VCP, Hdj-2, HSP90, Csp, CHIP, Sti1, Aha1, Dynein 1, cytoplasmic, intermediate chains, E2I, SUMO-2, HSPBP1
Beta-catenin-dependent transcription regulation in colorectal cancer	36	8.95E-08	4.19E-05	13	COX-2 (PTGS2), Beta-catenin, PLAUR (uPAR), AKT1, MTS1 (S100A4), LAMC2, LAMC2 (80kDa), LAMC2 (100kDa), CD44, CD44 soluble, PLAU (UPA), Fascin, CD44 (EXT)
LRRK2 in neurons in Parkinson's disease	33	2.24E-06	7.85E-04	11	Ubiquitin, MEK1/2, PAK1, AP2A2, HSP90, AP-2 alpha subunits, CHIP, MSN (moesin), eEF1A, Tubulin (in microtubules), VIL2 (ezrin)
dATP/dITP metabolism	94	3.06E-06	8.58E-04	19	POLE1, POLA2, AK2, ADSSL1, POLE4, NDPK 7, DNA polymerase beta, POLD reg (p50), POLD cat (p125), Adenosine kinase, POLG cat, DGUOK, ADSS, DNA polymerase lambda, NDPK 6, POLG reg, ADSL, PNPH, PKM2
Regulation of degradation of wtCFTR	20	1.19E-05	1.91E-03	8	Ubiquitin, BAG-2, UFD1, VCP, HSP90, Csp, CHIP, HSPBP1
Immune response_BAFF-induced non-canonical NF-kB signaling	32	1.25E-05	1.91E-03	10	Cullin 1, Ubiquitin, UBE1C, c-IAP2, E2N(UBC13), SUMO-1, c-IAP1, E2I, UBC12, NEDD8
Cell cycle_Start of DNA replication in early S phase	32	1.25E-05	1.91E-03	10	CDC18L (CDC6), MCM3, ASK (Dbf4), PP2A catalytic, CDC7, MCM2, ORC1L, MCM4, MCM10, RPA2
Apoptosis and survival_IL-17-induced CIKS-dependent MAPK signaling pathways	32	1.25E-05	1.91E-03	10	IKK-epsilon, Ubiquitin, MEK1/2, TPL2(MAP3K8), IL-17RC, E2N(UBC13), ABIN-2, TAB2, TRAF5, MEK4(MAP2K4)
Regulation of GSK3 beta in bipolar disorder	46	1.36E-05	1.91E-03	12	IL-1RI, MEK1/2, Shc, PP2A regulatory, AKT1, PP2A catalytic, AKT(PKB), IPP-2, FRAT1, Dsh, PP1-cat, DVL-1
Neurophysiological process_Dynein-dynactin motor complex in axonal transport in neurons	54	1.55E-05	1.97E-03	13	Ubiquitin, Importin (karyopherin)-alpha, Importin (karyopherin)-beta, CDK5, Centractins, NudE, DYNLL, AKT(PKB), Snapin, Dynein 1, cytoplasmic, intermediate chains, DYI2, NUDEL, Tubulin (in microtubules)
Cell cycle_Spindle assembly and chromosome separation	33	1.70E-05	1.99E-03	10	Ubiquitin, Importin (karyopherin)-alpha, DCTN2, Importin (karyopherin)-beta, HEC, Kid, Tubulin alpha, ZW10, Dynein 1, cytoplasmic, intermediate chains, Tubulin (in microtubules)
Translation_Insulin regulation of translation	42	2.99E-05	3.01E-03	11	MEK1(MAP2K1), eIF4B, eIF4H, Shc, eEF2, p70 S6 kinase1, eIF2B5, AKT(PKB), RPS6, PP1-cat, eIF4A
dCTP/dUTP metabolism	74	3.20E-05	3.01E-03	15	POLE1, PD-ECGF (TdRPase), DCTD, POLA2, AK2, POLE4, NDPK 7, DNA polymerase beta, POLD reg (p50), POLD cat (p125), POLG cat, DNA polymerase lambda, KCY, NDPK 6, POLG reg
Chemotaxis_Lysophosphatidic acid signaling via GPCRs	129	3.22E-05	3.01E-03	21	SIVA1, RhoA, Beta-catenin, MEK1/2, G-protein beta/gamma, Rho GTPase, G-protein alpha-12 family, PAK, AKT1, p70 S6 kinase1, Caspase-9, PKC, AKT(PKB), PKC-delta, TRIP6, Caspase-3, PLC-delta 1, Cofilin, IP3 receptor, MEK4(MAP2K4), PRK1
Cytoskeleton remodeling_Regulation of actin cytoskeleton organization by the kinase effectors of Rho GTPases	58	3.51E-05	3.01E-03	13	ARPC1B, RhoA, RhoA-related, PAK, Cofilin, non-muscle, PAK1, Cdc42 subfamily, RhoJ, RhoC, MSN (moesin), ERM proteins, Cofilin, PRK1
Role of IFN-beta in activation of T cell apoptosis in multiple sclerosis	29	3.65E-05	3.01E-03	9	c-IAP2, PP2A catalytic, Survivin, Caspase-9, AKT(PKB), PP2A cat (alpha), c-IAP1, Adenosine A2a receptor, Caspase-3
TTP metabolism	68	4.87E-05	3.57E-03	14	POLE1, PD-ECGF (TdRPase), POLA2, Thymidylate kinase, POLE4, NDPK 7, DNA polymerase beta, POLD reg (p50), POLD cat (p125), TYSY, POLG cat, DNA polymerase lambda, NDPK 6, POLG reg
Signal transduction_mTORC1 downstream signaling	60	5.13E-05	3.57E-03	13	BAD, eIF4B, eEF2, p70 S6 kinases, HMGCS2, p70 S6 kinase1, G6PD, HIF1A, RPS6, PFKP, SIN1, eIF4A, PDIP46
Transport_RAN regulation pathway	18	5.32E-05	3.57E-03	7	NUP54, Importin (karyopherin)-alpha, RANBP3, Importin (karyopherin)-beta, NTF2, SUMO-1, E2I
CFTR folding and maturation (normal and CF)	24	5.60E-05	3.57E-03	8	DNAJB6 (Hdj-1), PARP-1, Hdj-2, Csp, Sti1, Aha1, HSPBP1, p23 co-chaperone
Prolactin/ JAK2 signaling in breast cancer	24	5.60E-05	3.57E-03	8	SPHK1, STAT5, PAK1, SK4/IK1, SHP-2, STAT5B, PKM2, Cyclophilin A
Mechanisms of drug resistance in SCLC	70	6.81E-05	4.16E-03	14	BAD, VCAM1, p70 S6 kinase1, Survivin, Caspase-9, AKT(PKB), Collagen IV, CD81, Rad51, c-IAP1, RPS6, tBid, Caspase-3, Bid
Signal transduction_Adenosine A2B receptor signaling pathway	71	8.02E-05	4.69E-03	14	Cullin 1, Ubiquitin, JAB1, MEK1/2, G-protein beta/gamma, PKA-reg (cAMP-dependent), PKC, AKT(PKB), LDHA, HIF1A, VASP, IP3 receptor, PKM2, VIL2 (ezrin)
Apoptosis and survival_Ubiquitination and phosphorylation in TNF-alpha-induced NF-kB signaling	39	8.36E-05	4.69E-03	10	SPHK1, Ubiquitin, Sharpin, c-IAP2, E2N(UBC13), TAB2, PKC-delta, c-IAP1, RBCK1, UBE2D3
GTP-XTP metabolism	90	9.43E-05	5.09E-03	16	POLR2C, POLR2B, POLR1B, RPB6, POLR2G, NDPK 7, RPA39, RRP4, RRP42, GMP2, HPRT, POLR3K, NDPK 6, POLR3A, GUAC, PNPH
HGF signaling in colorectal cancer	64	1.04E-04	5.10E-03	13	COX-2 (PTGS2), Beta-catenin, MEK1/2, Shc, PLAUR (uPAR), AKT1, p70 S6 kinase1, LAMC2, AKT(PKB), CD44, SHP-2, TIMP1, VIL2 (ezrin)
dGTP metabolism	64	1.04E-04	5.10E-03	13	POLE1, POLA2, POLE4, NDPK 7, DNA polymerase beta, POLD reg (p50), POLD cat (p125), POLG cat, DGUOK, DNA polymerase lambda, NDPK 6, POLG reg, PNPH
Immune response_IL-1 signaling pathway	82	1.11E-04	5.10E-03	15	CCL5, COX-2 (PTGS2), SPHK1, IL-1RI, MEK1/2, TPL2(MAP3K8), c-IAP2, AKT(PKB), IRF1, ECSIT, CD44, PLAU (UPA), MEK4/7, IRAK1, MEK4(MAP2K4)
Apoptosis and survival_Regulation of Apoptosis by Mitochondrial Proteins	33	1.13E-04	5.10E-03	9	BAD, Caspase-9, VDAC 2, Bik, Bak, ANT, tBid, MAP1, Bid
Development_Adiponectin signaling	48	1.13E-04	5.10E-03	11	MEK1(MAP2K1), AdipoR2, Shc, Calmodulin, AKT(PKB), LKB1, HSP90, AdipoR1, Caspase-3, APPL1, IP3 receptor
ATP/ITP metabolism	123	1.55E-04	6.81E-03	19	POLR2C, POLR2B, POLR1B, RPB6, AK2, POLR2G, ADSSL1, NDPK 7, RPA39, RRP4, Adenosine kinase, RRP42, ADSS, HPRT, POLR3K, NDPK 6, ADSL, POLR3A, PNPH

Apoptosis and survival_BAD phosphorylation	42	1.64E-04	6.97E-03	10	BAD, MEK1(MAP2K1), Shc, G-protein beta/gamma, PKA-reg (cAMP-dependent), p70 S6 kinase1, PP2C, PP2A catalytic, AKT(PKB), PP1-cat alpha
Immune response_BAFF-induced signaling	51	2.01E-04	8.30E-03	11	BAD, Ubiquitin, MEK1/2, AKT1, p70 S6 kinase1, Bcl-10, AKT(PKB), TRIM2, PKC-delta, RPS6, MEK4(MAP2K4)
Cytoskeleton remodeling_Substance P mediated membrane blebbing	16	2.36E-04	9.48E-03	6	RhoA, G-protein alpha-12 family, Tubulin alpha, PAK1, Substance P receptor, Tubulin (in microtubules)
Translation_Translation regulation by Alpha-1 adrenergic receptors	53	2.88E-04	1.12E-02	11	MEK1(MAP2K1), RhoA, Shc, G-protein beta/gamma, eEF2, p70 S6 kinase1, Calmodulin, PKC- delta, eIF4A, IP3 receptor, PRK1
Translation_Regulation of EIF4F activity	54	3.42E-04	1.27E-02	11	MEK1(MAP2K1), eIF4B, eIF4H, Shc, p70 S6 kinase1, PP2A catalytic, PAK1, AKT(PKB), eIF4G2, eIF4A, MEK4(MAP2K4)
CHDI_DEGs from Replication data_Causal network	81	3.45E-04	1.27E-02	14	MEK1(MAP2K1), RhoA, Beta-catenin, Shc, LTBR(TNFRSF3), CDK5, Calmodulin, PKC, AKT(PKB), PKC-delta, Dsh, PP1-cat, IP3 receptor, MEK4(MAP2K4)
Tau pathology in Alzheimer disease	55	4.04E-04	1.41E-02	11	Ubiquitin, CDK5, VCP, PP2C, PP2A catalytic, Syndecan-1, Caspase-9, PKC, Calpain 2(m), Caspase-3, Tubulin (in microtubules)
Apoptosis and survival_Role of IAP-proteins in apoptosis	31	4.11E-04	1.41E-02	8	Ubiquitin, c-IAP2, Survivin, Caspase-9, c-IAP1, tBid, Caspase-3, Bid
NETosis in SLE	31	4.11E-04	1.41E-02	8	Pin1, PKC, Histone H3, HMGB1, Histone H2, Histone H2A, Histone H1.2, Histone H4
Regulation of lipid metabolism_Insulin signaling: generic cascades	47	4.37E-04	1.46E-02	10	MEK1(MAP2K1), eIF4B, Shc, eEF2, PKA-reg (cAMP-dependent), p70 S6 kinase1, eIF2B5, AKT(PKB), RPS6, PP1-cat
Development_VEGF signaling via VEGFR2 - generic cascades	93	4.66E-04	1.52E-02	15	COX-2 (PTGS2), SPHK1, MEK1(MAP2K1), RhoA, Beta-catenin, Shc, PLAUR (uPAR), Calmodulin, PKC, PAK1, AKT(PKB), HSP90, PLA2G5, PLAU (UPA), IP3 receptor
HSP70 and HSP40-dependent folding in Huntington's disease	25	5.52E-04	1.74E-02	7	Ubiquitin, PSMD1, DNAJB6 (Hdj-1), Hdj-2, HSP90, CHIP, Sti1
Signal transduction_Angiotensin II signaling via Beta-arrestin	57	5.57E-04	1.74E-02	11	BAD, MEK1(MAP2K1), RhoA, p70 S6 kinases, PP2A catalytic, AKT(PKB), AP-2 alpha subunits, SET, Cofilin, p23 co-chaperone, MEK4(MAP2K4)
Signal transduction_Soluble CXCL16 signaling	49	6.21E-04	1.90E-02	10	RhoA, MEK1/2, G-protein beta/gamma, CXCL16, p70 S6 kinase1, AKT(PKB), CD44, HIF1A, P-selectin, Cofilin
IGF family, invasion and metastasis in colorectal cancer	33	6.48E-04	1.90E-02	8	RhoA, Beta-catenin, PLAUR (uPAR), Collagen IV, PKC-delta, IGF-2, PLAU (UPA), Alpha-1 catenin
Development_Prolactin receptor signaling	58	6.51E-04	1.90E-02	11	MEK1(MAP2K1), RhoA, OAS1, Shc, STAT5, AKT(PKB), IRF1, PKC-delta, SHP-2, NMI, VAV-1
Development_Thromboxane A2 signaling pathway	50	7.35E-04	1.95E-02	10	RhoA, Beta-catenin, MEK1/2, G-protein alpha-12 family, PKA-reg (cAMP-dependent), p70 S6 kinase1, PKC, AKT(PKB), IP3 receptor, G-protein alpha-13
Apoptosis and survival_HTR1A signaling	50	7.35E-04	1.95E-02	10	MEK1(MAP2K1), Shc, G-protein beta/gamma, PP2A regulatory, PKA-reg (cAMP-dependent), PARP-1, PP2A catalytic, Calmodulin, AKT(PKB), Caspase-3
The role of PTEN and PI3K signaling in melanoma	50	7.35E-04	1.95E-02	10	BAD, Epo receptor, Beta-catenin, AKT1, p70 S6 kinase1, Survivin, Caspase-9, AKT(PKB), RhoC, Caspase-3
Signal transduction_mTORC2 downstream signaling	68	7.41E-04	1.95E-02	12	BAD, PRAS40, RhoA, Beta-catenin, PKA-reg (cAMP-dependent), AKT1, PKC, AKT(PKB), STK4, PKC-delta, IGF-2, Rab-10
Inhibition of apoptosis in gastric cancer	42	7.95E-04	1.95E-02	9	BAD, c-IAP2, Caspase-9, PAK1, AKT(PKB), c-IAP1, tBid, Caspase-3, Bid
Chemoresistance pathways mediated by constitutive activation of PI3K pathway and BCL-2 in small cell lung cancer	42	7.95E-04	1.95E-02	9	p70 S6 kinase1, PARP-1, LAMC2, Survivin, Caspase-9, AKT(PKB), Collagen IV, RPS6, Caspase- 3
Apoptosis and survival_Role of CDK5 in neuronal death and survival	34	8.03E-04	1.95E-02	8	BAD, MEK1(MAP2K1), Shc, CDK5, Caspase-9, AKT(PKB), PKC-delta, Caspase-3
Role of Apo-2L(TNFSF10) in Prostate Cancer cell apoptosis	34	8.03E-04	1.95E-02	8	c-IAP2, Survivin, Caspase-9, Bak, c-IAP1, tBid, Caspase-3, Bid
Development_Role of CDK5 in neuronal development	34	8.03E-04	1.95E-02	8	Beta-catenin, DCTN2, CDK5, Pin1, AKT(PKB), Dynein 1, cytoplasmic, intermediate chains, NUDEL, Tubulin (in microtubules)
Oxidative stress_ROS-induced cellular signaling	108	8.04E-04	1.95E-02	16	GSTP1, COX-2 (PTGS2), Thioredoxin, PKA-reg (cAMP-dependent), HIF-prolyl hydroxylase, p70 S6 kinase1, Pin1, GPX1, PKC, AKT(PKB), LKB1, Bak, HIF1A, NRF2, E2I, DLC1 (Dynein LC8a)
Chemotaxis_SDF-1/ CXCR4-induced chemotaxis of immune cells	79	9.00E-04	2.14E-02	13	RhoA, MEK1/2, Shc, G-protein beta/gamma, PKA-reg (cAMP-dependent), VCAM1, PAK, p70 S6 kinase1, PAK1, AKT(PKB), VAV-1, Cofilin, G-protein alpha-13
Apoptosis and survival_TNF-alpha-induced Caspase-8 signaling	43	9.53E-04	2.23E-02	9	PP2A regulatory, AKT1, PP2A catalytic, Caspase-9, AKT(PKB), HSP90, tBid, Caspase-3, Bid
Inhibition of remyelination in multiple sclerosis: regulation of cytoskeleton proteins	44	1.13E-03	2.59E-02	9	Tubulin beta, RhoA, CDK5, Tubulin alpha, PAK1, VAV-1, Stathmin, Cofilin, Tubulin (in microtubules)
Immune response_M-CSF-receptor signaling pathway	81	1.15E-03	2.59E-02	13	MEK1(MAP2K1), RhoA, Beta-catenin, Shc, Calmodulin, PKC, AKT(PKB), Tyk2, STAT5B, PRIM2A, VAV-1, PLAU (UPA), IP3 receptor
Effect of H. pylori infection on apoptosis in gastric epithelial cells	53	1.18E-03	2.64E-02	10	COX-2 (PTGS2), c-IAP2, PARP-1, Caspase-9, Bak, PTPR-alpha, c-IAP1, tBid, Caspase-3, Bid
Possible regulation of HSF-1/ chaperone pathway in Huntington's disease	21	1.23E-03	2.70E-02	6	Calmodulin, HSP90, PLA2, E2I, SUMO-2, p23 co-chaperone
Apoptosis and survival_CXCR3-B signaling	63	1.33E-03	2.81E-02	11	G-protein beta/gamma, PKA-reg (cAMP-dependent), MCU, PARP-1, Survivin, Caspase-9, IDO2, NRF2, Calpain 2(m), Caspase-3, IP3 receptor
Transcription_Sirtuin6 regulation	63	1.33E-03	2.81E-02	11	Ubiquitin, c-IAP2, PARP-1, Histone H3, Sirtuin6, LKB1, CHIP, LDHA, HIF1A, LDLR, GPIAP1

and functions					
Cell adhesion_Histamine H1 receptor signaling in the interruption of cell barrier integrity	45	1.34E-03	2.81E-02	9	RhoA, Beta-catenin, G-protein beta/gamma, GEFT, Calmodulin, PKC-delta, Alpha-catenin, Cofilin, IP3 receptor
Development_FGFR signaling pathway	54	1.37E-03	2.84E-02	10	Ubiquitin, MEK1(MAP2K1), Shc, Syndecan-1, PAK1, AKT(PKB), PKC-delta, SHP-2, IP3 receptor, MEK4(MAP2K4)
Cell cycle_Role of SCF complex in cell cycle regulation	29	1.45E-03	2.95E-02	7	Cullin 1, Ubiquitin, CDC25A, RING-box protein 1, CKS1, NEDD8, CDC34
Signal transduction_CXCR4 signaling via PI3K cascade	46	1.58E-03	3.01E-02	9	BAD, Ubiquitin, Beta-catenin, G-protein beta/gamma, AKT1, p70 S6 kinase1, PARP-1, AKT(PKB), Caspase-3
Role of GIP in pathogenesis of type 2 diabetes	46	1.58E-03	3.01E-02	9	BAD, Ubiquitin, MEK1/2, PKA-reg (cAMP-dependent), PP2A catalytic, Caspase-9, AKT(PKB), Caspase-3, MEK4(MAP2K4)
Signal transduction_Calcium signaling	46	1.58E-03	3.01E-02	9	CaMK I, RhoA, G-protein beta/gamma, PP2A catalytic, Calmodulin, CD44, PP1-cat, IP3 receptor, VIL2 (ezrin)
Cell adhesion_ECM remodeling	55	1.59E-03	3.01E-02	10	PLAUR (uPAR), Collagen IV, CD44, Caveolin-2, MSN (moesin), MMP-16, IGF-2, PLAU (UPA), TIMP1, VIL2 (ezrin)
Immune response_IL-6 signaling pathway via MEK/ERK and PI3K/AKT cascades	74	1.60E-03	3.01E-02	12	BAD, MEK1/2, Shc, p70 S6 kinase1, Caspase-9, AKT(PKB), Proepithelin, PKC-delta, SHP-2, RPS6, VAV-1, IP3 receptor
Ubiquitin-proteasome system in Huntington's disease	22	1.61E-03	3.01E-02	6	PSMC2, Ubiquitin, PSMB9, CHIP, HIP-2, CDC34
TNF-alpha and IL-1 beta-mediated regulation of contraction and secretion of inflammatory factors in normal and asthmatic airway smooth muscle	65	1.74E-03	3.18E-02	11	CCL5, COX-2 (PTGS2), IL-1RI, GRO-2, RhoA, VCAM1, Calmodulin, Histone H3, AKT(PKB), PLA2, Histone H4
Signal transduction_Cyclic AMP signaling	38	1.74E-03	3.18E-02	8	KDELR, G-protein beta/gamma, PKA-reg (cAMP-dependent), Calmodulin, PKC, PHK alpha, PKC-delta, PHK gamma
Apoptosis and survival_Endoplasmic reticulum stress response pathway	56	1.83E-03	3.26E-02	10	GADD34, eIF2S1, Caspase-9, Bak, Calpain 2(m), PP1-cat, tBid, PP1-cat alpha, MEK4(MAP2K4), Bid
Role of FSH and Lutropin in ovarian cancer	56	1.83E-03	3.26E-02	10	COX-2 (PTGS2), MEK1/2, PKA-reg (cAMP-dependent), Survivin, AKT(PKB), CD44, HIF1A, PKC-delta, NRF2, APPL1
CTP/UTP metabolism	107	2.04E-03	3.47E-02	15	POLR2C, PD-ECGF (TdRPase), POLR2B, POLR1B, RPB6, AK2, POLR2G, NDPK 7, RPA39, RRP4, RRP42, POLR3K, KCY, NDPK 6, POLR3A
Role of alpha-V/ beta-6 integrin in colorectal cancer	23	2.07E-03	3.47E-02	6	MEK1/2, Caspase-9, PKC, Collagen IV, PLAU (UPA), Caspase-3
Proteolysis_Putative ubiquitin pathway	23	2.07E-03	3.47E-02	6	Cullin 1, Ubiquitin, E2N(UBC13), RING-box protein 1, UBCH8, CHIP
Signal transduction_Leptin signaling via JAK/STAT and MAPK cascade	39	2.08E-03	3.47E-02	8	MEK1/2, STAT5, Caspase-9, SHP-2, STAT5B, RPS6, Caspase-3, TIMP1
Regulation of lipid metabolism_Insulin regulation of glycogen metabolism	57	2.10E-03	3.47E-02	10	MEK1(MAP2K1), Shc, PKA-reg (cAMP-dependent), Calmodulin, AKT(PKB), PHK alpha, PP1- cat, PHK gamma (muscle), GYS1, PHK gamma
Lysophospholipid mediators-induced inflammatory signaling in normal and asthmatic airway epithelium	57	2.10E-03	3.47E-02	10	CCL5, COX-2 (PTGS2), SPHK1, RhoA, MEK1/2, G-protein beta/gamma, G-protein alpha-12 family, AKT(PKB), IRF1, PKC-delta
Signal transduction_Adenosine A3 receptor signaling pathway	48	2.16E-03	3.48E-02	9	BAD, MEK1/2, G-protein beta/gamma, Rho GTPase, PKC, AKT(PKB), HIF1A, PKC-delta, IP3 receptor
GLP-1 in beta cell apoptosis in type 2 diabetes	48	2.16E-03	3.48E-02	9	BAD, IL-1RI, Thioredoxin, c-IAP2, PKA-reg (cAMP-dependent), AKT1, Caspase-9, IGF-2, Caspase-3
Role of neuropeptides in pathogenesis of SCLC	67	2.23E-03	3.56E-02	11	MEK1/2, Shc, G-protein beta/gamma, G-protein alpha-12 family, p70 S6 kinase1, PKC, AKT(PKB), PKC-delta, V1a receptor, Substance P receptor, IP3 receptor
Apoptosis and survival_NGF/ TrkA PI3K-mediated signaling	77	2.27E-03	3.57E-02	12	BAD, RhoA, Shc, AKT1, p70 S6 kinase1, Cofilin, non-muscle, Calmodulin, PAK1, AKT(PKB), MSN (moesin), Cofilin, Tubulin (in microtubules)
Immune response_IL-33 signaling pathway	58	2.41E-03	3.68E-02	10	Ubiquitin, MEK1/2, VCAM1, TAB2, AKT(PKB), Histone H2A, IL-33, Histone H2B, IRAK1, MEK4(MAP2K4)
Apoptosis and survival_Ceramides signaling pathway	40	2.46E-03	3.68E-02	8	BAD, MEK1(MAP2K1), PP2A catalytic, AKT(PKB), tBid, Caspase-3, MEK4(MAP2K4), Bid
CHDI_Correlations from Replication data_Causal network (negative correlations)	40	2.46E-03	3.68E-02	8	RhoA, Beta-catenin, PLAUR (uPAR), PKC, AKT(PKB), Collagen IV, Dsh, PLAU (UPA)
Tissue Factor signaling in cancer via PAR1 and PAR2	49	2.50E-03	3.68E-02	9	MEK1(MAP2K1), Shc, G-protein alpha-12 family, p70 S6 kinase1, AKT(PKB), Tissue factor, SHP-2, STAT5B, IP3 receptor
Development_G-CSF signaling	49	2.50E-03	3.68E-02	9	MEK1(MAP2K1), Shc, STAT5, c-IAP2, G-CSF receptor, Survivin, AKT(PKB), Tyk2, SHP-2
Immune response_Sublytic effects of membrane attack complex	68	2.52E-03	3.68E-02	11	BAD, RhoA, MEK1/2, G-protein beta/gamma, MCU, AKT1, eIF2S1, PKC, AKT(PKB), IRF1, IP3 receptor
Eosinophil adhesion and transendothelial migration in asthma	68	2.52E-03	3.68E-02	11	CCL5, Galectin-3, PLAUR (uPAR), VCAM1, Calmodulin, PKC, Collagen IV, CD44, PKC-delta, P-selectin, PLAU (UPA)
Cell cycle_Role of Nek in cell cycle regulation	32	2.67E-03	3.82E-02	7	Tubulin beta, NEK6, HEC, p70 S6 kinase1, Tubulin alpha, Histone H3, Tubulin (in microtubules)

Epithelial cell apoptosis in COPD	32	2.67E-03	3.82E-02	7	BAD, PSMA1, Caspase-9, PSMB6, AKT(PKB), NRF2, NIP3
Development_Negative regulation of WNT/Beta-catenin signaling in the nucleus	89	2.74E-03	3.84E-02	13	Beta-catenin, RANBP3, AKT1, TAB2, NARF, HBP1, Oct-3/4, VHL, PKC-delta, Nitrilase 1, Dsh, PGAM5, Alpha-1 catenin
Oxidative stress_Activation of NADPH oxidase	59	2.74E-03	3.84E-02	10	MEK1/2, G-protein beta/gamma, AKT1, Pin1, PKC, PAK1, AKT(PKB), PKC-delta, VAV-1, IP3 receptor
LRRK2 in neuronal apoptosis in Parkinson's disease	17	2.77E-03	3.84E-02	5	Thioredoxin, AKT1, Caspase-9, ANT, Caspase-3
CHDI_Correlations from Replication data_Causal network (positive correlations)	79	2.83E-03	3.84E-02	12	PSMC2, IL-1RI, RhoA, G-protein beta/gamma, IRAK1/2, Calmodulin, AKT(PKB), CD44, Caspase-3, IP3 receptor, MEK4(MAP2K4), VIL2 (ezrin)
Development_IGF-1 receptor signaling	50	2.89E-03	3.84E-02	9	BAD, MEK1(MAP2K1), Shc, p70 S6 kinase1, Caspase-9, AKT(PKB), RPS6, IGF-2, GYS1
Development_GM-CSF signaling	50	2.89E-03	3.84E-02	9	BAD, MEK1(MAP2K1), RACK1, Shc, STAT5, Caspase-9, AKT(PKB), SHP-2, Caspase-3
IGF-1 signaling in multiple myeloma	50	2.89E-03	3.84E-02	9	BAD, RhoA, MEK1/2, Shc, c-IAP2, p70 S6 kinase1, Survivin, AKT(PKB), HIF1A
Cytoskeleton remodeling_Role of PKA in cytoskeleton reorganisation	41	2.90E-03	3.84E-02	8	RhoA, G-protein beta/gamma, PKA-reg (cAMP-dependent), Calmodulin, PAK1, VASP, Cofilin, IP3 receptor
IGF family signaling in colorectal cancer	60	3.12E-03	3.95E-02	10	COX-2 (PTGS2), GIPC, Beta-catenin, MEK1/2, Shc, p70 S6 kinase1, AKT(PKB), HIF1A, Rad51, IGF-2
Signal transduction_MIF signaling pathway	60	3.12E-03	3.95E-02	10	BAD, COX-2 (PTGS2), MEK1/2, G-protein beta/gamma, VCAM1, AKT(PKB), SPPL2a, CD44, PRDX1, NRF2
Main pathways of Schwann cells transformation in neurofibromatosis type 1	80	3.14E-03	3.95E-02	12	BAD, MEK1(MAP2K1), Beta-catenin, MEK1/2, p70 S6 kinase1, Calmodulin, PAK1, AKT(PKB), HIF1A, RPS6, VAV-1, IP3 receptor
Stellate cells activation and liver fibrosis	70	3.18E-03	3.95E-02	11	IL-1RI, GRO-2, MEK1(MAP2K1), Beta-catenin, Shc, IRAK1/2, GLI-1, AKT(PKB), SMAD2, Dsh, TIMP1
Aminoglycoside- and cisplatin-induced hair cell death	112	3.20E-03	3.95E-02	15	COX-2 (PTGS2), Beta-catenin, Shc, NOL3, PARP-1, Caspase-9, PKC, Histone H3, Calpain 2(m), Histone H2A, Histone H2B, Histone H4, Caspase-3, MEK4(MAP2K4), ACON
Role of metalloproteases and heparanase in progression of pancreatic cancer	33	3.21E-03	3.95E-02	7	MEK1(MAP2K1), Shc, Collagen IV, CD44, CD44 soluble, TIMP1, CD44 (EXT)
Apoptosis and survival_Granzyme B signaling	33	3.21E-03	3.95E-02	7	PARP-1, Tubulin alpha, Caspase-9, tBid, Caspase-3, LAMP2, Bid
Resistance of pancreatic cancer cells to death receptor signaling	33	3.21E-03	3.95E-02	7	c-IAP2, Survivin, Caspase-9, c-IAP1, tBid, Caspase-3, Bid
Development_Glucocorticoid receptor signaling	25	3.26E-03	3.95E-02	6	STAT5, HSP90, SUMO-1, FKBP4, E2I, p23 co-chaperone
Defective macrophage-mediated bacterial phagocytosis in COPD	25	3.26E-03	3.95E-02	6	CR1, Tubulin alpha, AKT(PKB), SR-BI, NRF2, Tubulin (in microtubules)
HIF-1 in gastric cancer	51	3.33E-03	3.99E-02	9	COX-2 (PTGS2), Ubiquitin, PDF, PGES2, AKT(PKB), HSP90, LDHA, VHL, HIF1A
Development_c-Kit ligand signaling pathway during hemopoiesis	61	3.53E-03	4.20E-02	10	BAD, MEK1/2, Shc, DOK1, STAT5, p70 S6 kinase1, AKT(PKB), SHP-2, VAV-1, IP3 receptor
Regulation of lipid metabolism_Alpha-1 adrenergic receptors signaling via arachidonic acid	71	3.57E-03	4.21E-02	11	COX-2 (PTGS2), RhoA, MEK1/2, Shc, G-protein beta/gamma, Calmodulin, PGES2, LPP2, LPP1, IP3 receptor, PRK1
Development_The role of GDNF ligand family/ RET receptor in cell survival, growth and proliferation	92	3.68E-03	4.31E-02	13	RhoA, MEK1/2, Shc, DOK1, Calmodulin, CREM (activators), AKT(PKB), HIF1A, ARTN, SHP- 2, Cofilin, IP3 receptor, MEK4(MAP2K4)
Signal transduction_AKT signaling	43	3.95E-03	4.39E-02	8	BAD, p70 S6 kinase1, PP2A catalytic, Caspase-9, AKT(PKB), HSP90, RPS6, GYS1
Immune response_Substance P-stimulated expression of proinflammatory cytokines via MAPKs	43	3.95E-03	4.39E-02	8	COX-2 (PTGS2), GRO-2, MEK1/2, G-protein beta/gamma, AKT(PKB), PKC-delta, Substance P receptor, IP3 receptor
Apoptosis and survival_TNFR1 signaling pathway	43	3.95E-03	4.39E-02	8	c-IAP2, jBid, Caspase-9, c-IAP1, tBid, Caspase-3, MEK4(MAP2K4), Bid
Muscle contraction_Oxytocin signaling in uterus and mammary gland	62	3.98E-03	4.39E-02	10	COX-2 (PTGS2), MEK1(MAP2K1), RhoA, G-protein beta/gamma, eEF2, PP2A catalytic, Calmodulin, PGES2, PKC, IP3 receptor
Regulation of CFTR activity (normal and CF)	62	3.98E-03	4.39E-02	10	RACK1, PP2A regulatory, PKA-reg (cAMP-dependent), Annexin V, PP2C, PP2A catalytic, Syntaxin 1A, COMMD1 (MURR1), Tubulin (in microtubules), VIL2 (ezrin)
Regulation of lipid metabolism_Stimulation of Arachidonic acid production by ACM receptors	72	3.98E-03	4.39E-02	11	MEK1(MAP2K1), RhoA, G-protein beta/gamma, Calmodulin, PKC, PAK1, ARF1, LPP2, LPP1, IP3 receptor, G-protein alpha-13
Nicotine / nAChR alpha-3/nAChR beta-2 signaling in NSCLC	26	4.03E-03	4.39E-02	6	CDC18L (CDC6), AKT1, CDC25A, Survivin, AKT(PKB), TYSY
Neurophysiological process_Dopamine D2 receptor transactivation of PDGFR in CNS	26	4.03E-03	4.39E-02	6	G-protein beta/gamma, PP2A regulatory, PP2A catalytic, Calmodulin, PKC, IP3 receptor
Cell cycle_Transition and termination of DNA replication	26	4.03E-03	4.39E-02	6	Ribonuclease H1, Ubiquitin, MCM2, POLD reg (p50), FEN1, POLD cat (p125)

Apoptosis and survival_Role of PKR in stress-induced apoptosis	53	4.35E-03	4.56E-02	9	PP2A regulatory, IRF3, PP2A catalytic, eIF2S1, TAB2, IRF1, Tyk2, ATF-3, Caspase-3
Regulation of VEGF expression in lung cancer	53	4.35E-03	4.56E-02	9	COX-2 (PTGS2), IL-1RI, RhoA, p70 S6 kinase1, AKT(PKB), HSP90, RhoC, VHL, HIF1A
TLRs-mediated IFN-alpha production by plasmacytoid dendritic cells in SLE	53	4.35E-03	4.56E-02	9	La protein, SNRPB (Sm-B), SSH3, p70 S6 kinase1, TAB2, AKT(PKB), HMGB1, SNRPD1 (SMD1), IRAK1
Immune response_Induction of the antigen presentation machinery by IFN-gamma	53	4.35E-03	4.56E-02	9	PSMB9, IFNGR1, RFXANK, PSMB8(LMP7), NLRC5, IRF1, Nucleophosmin, USF1, PSMB10
CHDI_Correlations from Discovery data_Causal network	73	4.44E-03	4.56E-02	11	MEK1(MAP2K1), Beta-catenin, Shc, G-protein beta/gamma, CDK5, Calmodulin, AKT(PKB), Dsh, Substance P receptor, IP3 receptor, TSC-22
Neuroprotective action of lithium	63	4.48E-03	4.56E-02	10	Thioredoxin, Beta-catenin, CDK5, IPP-2, FRAT1, P2X2, Dsh, PP1-cat, Caspase-3, MEK4(MAP2K4)
Neurogenesis_NGF/ TrkA MAPK-mediated signaling	105	4.51E-03	4.56E-02	14	SPHK1, MEK1/2, Shc, PLAUR (uPAR), PP2A regulatory, PKA-reg (cAMP-dependent), CDK5, PP2A catalytic, Calmodulin, PKC-delta, SHP-2, Ephrin-A receptor 2, SAC, IP3 receptor
Interleukins-induced inflammatory response in asthmatic airway fibroblasts	35	4.54E-03	4.56E-02	7	CCL5, COX-2 (PTGS2), IL-1RI, GRO-2, MEK1/2, VCAM1, IL-33
Anti-apoptotic action of Gastrin in gastric cancer	35	4.54E-03	4.56E-02	7	BAD, MEK1(MAP2K1), RhoA, Caspase-9, PAK1, Caspase-3, IP3 receptor
IGF signaling in lung cancer	44	4.58E-03	4.56E-02	8	BAD, MEK1(MAP2K1), Shc, p70 S6 kinase1, Survivin, AKT(PKB), IGF-2, PLAU (UPA)
Development_Flt3 signaling	44	4.58E-03	4.56E-02	8	BAD, MEK1(MAP2K1), Shc, PAK1, AKT(PKB), SHP-2, VAV-1, MEK4(MAP2K4)
Apoptosis and survival_FAS signaling cascades	44	4.58E-03	4.56E-02	8	PARP-1, Caspase-9, SUMO-1, c-IAP1, tBid, Caspase-3, MEK4(MAP2K4), Bid
Translation_Regulation of translation initiation	27	4.92E-03	4.82E-02	6	RACK1, eIF3S7, p70 S6 kinase1, eIF3S4, RPS6, eIF4A
Ubiquinone metabolism	74	4.94E-03	4.82E-02	11	COQ3, NDUFS8, NDUFA11, NDUFS1, NDUFS3, DAP13, NDUFS2, NDUFS7, NDUFB6, NDUFA6, NDUFV3
NRF2 regulation of oxidative stress response	54	4.94E-03	4.82E-02	9	GSTP1, Ubiquitin, MEK1(MAP2K1), Thioredoxin, PKC, AKT(PKB), PRDX1, NRF2, MEK4(MAP2K4)

Items with FDR < 0.05 were considered as significant ones.

Supplementary Table 7. The significantly enriched metacore pathways for the 1551 down-regulated genes under siPde4d.

Maps	Total	p-value	FDR	In_Data	Network Objects from Active Data
Oxidative stress_ROS-induced cellular signaling	108	1.12E-10	1.50E-07	24	NOTCH1 (NICD), ELAVL1 (HuR), p38alpha (MAPK14), SENP1, HSF1, PLK3 (CNK), PRKD1, PKA-reg (cAMP-dependent), JNK(MAPK8-10), HIF-prolyl hydroxylase, HDAC1, FIH-1, SAE2, Glutaredoxin 1, p21, Bak, Cytochrome c, MEKK1(MAP3K1), NRF2, E2I, p38 MAPK, IKK-beta, MDM2, TNF-alpha
Transcription_Negative regulation of HIF1A function	69	1.14E-08	7.62E-06	17	PLK3 (CNK), SART1, FHL3, HIF-prolyl hydroxylase, EGLN2, MCM7, ARD1, FIH-1, MCM2, Sirtuin6, HSP90, CITED2, FBXW7, HSP90 beta, Elongin C, MDM2, AML1 (RUNX1)
Oxidative stress_Role of ASK1 under oxidative stress	55	1.87E-08	8.36E-06	15	MAPK8/9, MEKK6(MAP3K6), PRKD1, JNK(MAPK8-10), JNK2(MAPK9), HIPK1, PP2A catalytic, SOD1, Gemin5, TRAF2, p38 MAPK, ASK1 (MAP3K5), Glutaredoxin, DAXX, 14-3-3
Immune response_Substance P-stimulated expression of proinflammatory cytokines via MAPKs	43	3.82E-07	8.97E-05	12	GRO-2, MIP-1-beta, I-kB, G-protein beta/gamma, JNK(MAPK8-10), CCL13, CCL2, p38 MAPK, Substance P receptor, IKK-beta, c-Jun, TNF-alpha
Immune response_IFN-alpha/beta signaling via MAPKs	77	3.91E-07	8.97E-05	16	GCH1, TAP1 (PSF1), IRF9, JNK(MAPK8-10), HDAC1, PML, IP10, ISG15, p21, MEKK1(MAP3K1), Tyk2, p38 MAPK, ISG54, Oasl2, c-Jun, RIG-G
Proteolysis_Putative SUMO-1 pathway	29	4.02E-07	8.97E-05	10	SENP1, HSF1, RanGAP1, SAE2, PML, SP100, E2I, c-Jun, MDM2, DAXX
Attenuation of IFN type I signaling in melanoma cells	37	5.80E-07	1.11E-04	11	p38alpha (MAPK14), TAP1 (PSF1), IRF9, IP10, p21, MEKK1(MAP3K1), Tyk2, I-TAC, TAP, TAP2 (PSF2), c-Jun
Apoptosis and survival_Lymphotoxin-beta receptor signaling	42	2.35E-06	3.93E-04	11	LTB, I-kB, JNK(MAPK8-10), Caspase-7, Cytochrome c, MEKK1(MAP3K1), TRAF2, ASK1 (MAP3K5), SDF-1, IKK-beta, c-Jun
Apoptosis and survival_Role of PKR in stress-induced apoptosis	53	4.33E-06	6.45E-04	12	I-kB, PP2A regulatory, Caspase-7, C/EBP zeta, PP2A catalytic, p21, Tyk2, ATF-3, TRAF2, TARBP2, IKK-beta, TNF-alpha
Immune response_HSP60 and HSP70/ TLR signaling pathway	54	5.34E-06	7.15E-04	12	I-kB, CD80, IRAK1/2, JNK(MAPK8-10), E2N(UBC13), UBE1, HSP60, UEV1A, p38 MAPK, IKK-beta, c-Jun, TNF-alpha
Immune response_IFN-alpha/beta signaling via JAK/STAT	64	6.29E-06	7.66E-04	13	CrkL, TAP1 (PSF1), IRF9, Caspase-7, PML, IP10, ISG15, p21, CCL2, Tyk2, I-TAC, ISG54, RIG-G
Development_TNF-alpha, IL-1 alpha and WNT5A-dependent regulation of osteogenesis and adipogenesis in mesenchymal stem cells	47	7.73E-06	8.62E-04	11	G-protein beta/gamma, TAZ, IRAK1/2, JNK(MAPK8-10), ROR2, HDAC3, TRAF2, IKK-beta, c-Jun, TNF-alpha, 14-3- 3
Cell cycle_Start of DNA replication in early S phase	32	1.05E-05	1.08E-03	9	ASK (Dbf4), PP2A catalytic, CDC7, MCM2, Cdt1, HP1 alpha, Geminin, CDK2, CDC45L
Apoptosis and survival_Granzyme B signaling	33	1.38E-05	1.32E-03	9	Rab-27A, Caspase-7, Tubulin alpha, MUNC13-4, Cytochrome c, Caspase-6, NOTCH1 (NEXT), FGFR1, Lamin B1
Oxidative stress_ROS-mediated activation of MAPK via inhibition of phosphatases	34	1.80E-05	1.50E-03	9	MKP-7, PP2A regulatory, JNK(MAPK8-10), PP2A catalytic, SOD1, p38 MAPK, ASK1 (MAP3K5), VHR, PDGF-R-beta
Apoptosis and survival_Cytoplasmic/mitochondrial transport of proapoptotic proteins Bid, Bmf and Bim	34	1.80E-05	1.50E-03	9	GCKR(MAP4K5), Cytochrome c, MEKK1(MAP3K1), FLASH, TRAF2, Bim, ASK1 (MAP3K5), TNF-alpha, DAXX
Development_Notch Signaling Pathway	43	2.13E-05	1.59E-03	10	NOTCH1 (NICD), HDAC1, NOTCH1 receptor, TLE, NOTCH1 (NEXT), CIR, FBXW7, Histone H4, NOTCH1 precursor, GCN5
Apoptosis and survival_TNFR1 signaling pathway	43	2.13E-05	1.59E-03	10	I-kB, JNK(MAPK8-10), Caspase-7, Cytochrome c, Caspase-6, MEKK1(MAP3K1), FLASH, TRAF2, c-Jun, TNF-alpha
Apoptosis and survival_FAS signaling cascades	44	2.64E-05	1.86E-03	10	Lamin B, JNK(MAPK8-10), Caspase-7, Cytochrome c, Caspase-6, MEKK1(MAP3K1), FLASH, Bim, ASK1 (MAP3K5), DAXX
Mast cell migration in asthma	73	2.81E-05	1.88E-03	13	G-protein beta/gamma, CXCL16, CCR1, IP10, PDGF receptor, LTBR2, CCL2, I-TAC, p38 MAPK, G-protein alpha-i family, SDF-1, FGFR1, TGF-beta receptor type I
Immune response_HMGB1/TLR signaling pathway	36	2.97E-05	1.89E-03	9	p38alpha (MAPK14), MIP-1-beta, I-kB, IRAK1/2, E2N(UBC13), UBE1, UEV1A, IKK-beta, TNF-alpha
Immune response_TNF-R2 signaling pathways	45	3.26E-05	1.98E-03	10	I-kB, AIP1 (DAB2ip), JNK(MAPK8-10), TRAF2, p38 MAPK, Bim, ASK1 (MAP3K5), IKK-beta, c-Jun, TNF-alpha
Noise-induced hair cell death and spiral ganglion neuron degeneration	96	3.50E-05	2.04E-03	15	p38alpha (MAPK14), AMPK gamma subunit, MIP-1-beta, JNK(MAPK8-10), TMC1, HDAC1, HDAC3, CCL13, Bak, Cytochrome c, CCL2, ERM proteins, AMPK beta subunit, RIPK3, TNF-alpha
Apoptosis and survival_Endoplasmic reticulum stress response pathway	56	4.51E-05	2.51E-03	11	p38alpha (MAPK14), I-kB, JNK(MAPK8-10), Caspase-7, C/EBP zeta, Bak, Cytochrome c, TRAF2, Bim, ASK1 (MAP3K5), c-Jun
TNF-alpha-induced inflammatory signaling in normal and asthmatic airway epithelium	38	4.73E-05	2.51E-03	9	I-kB, PP2A catalytic, IP10, MEKK1(MAP3K1), CCL2, p38 MAPK, IKK-beta, c-Jun, TNF-alpha
Signal transduction_JNK pathway	47	4.86E-05	2.51E-03	10	HPK1(MAP4K1), MLK3(MAP3K11), JNK(MAPK8-10), GCKR(MAP4K5), MEKK1(MAP3K1), TRAF2, ASK1 (MAP3K5), c-Jun, TNF-alpha, DAXX
Transcription_ChREBP regulation pathway	23	5.88E-05	2.82E-03	7	AMPK gamma subunit, G-protein beta/gamma, PKA-reg (cAMP-dependent), PP2A catalytic, AMPK beta subunit, G-protein alpha-i family, Acyl-CoA synthetase
Regulation of degradation of deltaF508-CFTR in CF	39	5.89E-05	2.82E-03	9	UBE2J1, UBE2D1, UBE1, Hdj-2, HSP90, Csp, Sti1, E2I, DNAJB12
Putative glucocorticoid- and LABA-mediated inhibition of pro-fibrotic signaling in airway fibroblasts/myofibroblasts	31	6.46E-05	2.98E-03	8	PKA-reg (cAMP-dependent), JNK(MAPK8-10), cAMP-GEFI, p21, p38 MAPK, c-Jun, TIMP1, TGF-beta receptor type I

Transcription_Role of heterochromatin protein 1 (HP1) family in transcriptional silencing	40	7.29E-05	3.26E-03	9	Mi-2, HDAC1, MEF2, HP1 beta, HP1 alpha, E2I, HP1, Histone H4, Mi-2 alpha
Oxidative stress_ROS-mediated MAPK activation via canonical pathways	60	8.77E-05	3.64E-03	11	MLK3(MAP3K11), JNK(MAPK8-10), JNK2(MAPK9), MEKK1(MAP3K1), TRAF2, TSAD, p38 MAPK, ASK1 (MAP3K5), CaMK II alpha, FGFR1, PDGF-R-beta
Maturation and migration of dendritic cells in skin sensitization	41	8.96E-05	3.64E-03	9	I-kB, CD80, JNK(MAPK8-10), MEKK1(MAP3K1), TRAF2, p38 MAPK, ASK1 (MAP3K5), c-Jun, TNF-alpha
Transcription_Sin3 and NuRD in transcription regulation	41	8.96E-05	3.64E-03	9	Mi-2, HDAC1, NRB54, SAP18, Mi-2 beta, MTA2, SAP130, Histone H4, Mi-2 alpha
Development_Positive regulation of STK3/4 (Hippo) pathway and negative regulation of YAP/TAZ function	71	9.66E-05	3.81E-03	12	GCGR, MOBKL1A, Mol1b, AMPK gamma subunit, TAZ, PKA-reg (cAMP-dependent), PP2A cat (alpha), RASSF1, AMPK beta subunit, RhoGDI alpha, LIF, 14-3-3
Development_Beta-adrenergic receptors signaling via Cyclic AMP	51	1.02E-04	3.88E-03	10	BETA-PIX, Phospholamban, PRKAR2A, PKA-reg type II (cAMP-dependent), PKA-reg (cAMP-dependent), PP2A catalytic, PHK alpha (muscle), PDE4D, p38 MAPK, Perilipin
Signal transduction_Activin A signaling regulation	33	1.05E-04	3.90E-03	8	ALK-4, Follistatin, FSRP, HDAC1, SMURF1, Histone H2, DLX1, Histone H4
Regulation of CFTR activity (normal and CF)	62	1.20E-04	4.33E-03	11	Casein kinase II, beta chain (Phosvitin), PRKAR2A, PP2A regulatory, PKA-reg type II (cAMP-dependent), PKA-reg (cAMP-dependent), PP2C, PP2A catalytic, PDE4D, AMPK beta subunit, G-protein alpha-i family, VIL2 (ezrin)
Signal transduction_Erk Interactions: Inhibition of Erk	34	1.31E-04	4.63E-03	8	MKP-2, MKP-7, PKA-reg (cAMP-dependent), JNK(MAPK8-10), PP2A catalytic, MKP-4, GMF, VHR
Signal transduction_CXCR4 signaling via MAPKs cascades	53	1.42E-04	4.89E-03	10	Cortactin, G-protein beta/gamma, JNK(MAPK8-10), PAK, MEKK1(MAP3K1), p38 MAPK, G-protein alpha-i family, SDF-1, c-Jun, Beta-arrestin2
Immune response_Antigen presentation by MHC class I, classical pathway	54	1.68E-04	5.61E-03	10	PSMB5, MHC Class I alpha chain, TAP1 (PSF1), HSP90 alpha, Bleomycin hydrolase, Sec24, PC7, PSMB10, TAP, TAP2 (PSF2)
Immune response_Antigen presentation by MHC class I: cross-presentation	99	1.91E-04	6.24E-03	14	Rab-6, Rab-27A, HSP90 alpha, Adipophilin, HSP60, Fc gamma RII alpha, HSP90, Syntaxin 4, Rab8B, TAP, OLR1, Rab-34, IKK-beta, Connexin 43
Development_Keratinocyte differentiation	56	2.29E-04	7.31E-03	10	NOTCH1 (NICD), MAD, p38alpha (MAPK14), p21, Fra-1, MEKK1(MAP3K1), TGM1, ASK1 (MAP3K5), c-Jun, TGF-beta receptor type I
Immune response_Role of PKR in stress-induced antiviral cell response	57	2.67E-04	8.30E-03	10	p38alpha (MAPK14), I-kB, JNK(MAPK8-10), Caspase-7, Tyk2, TRAF2, TARBP2, IKK-beta, c-Jun, TNF-alpha
CHDI_Correlations from Replication data_Causal network (positive correlations)	79	2.75E-04	8.38E-03	12	I-kB, G-protein beta/gamma, CD80, IRAK1/2, JNK(MAPK8- 10), MEF2, p38 MAPK, SDF-1, IKK-beta, c-Jun, RhoGDI alpha, VIL2 (ezrin)
Immune response_CD137 signaling in immune cell	29	2.93E-04	8.48E-03	7	CD137(TNFRSF9), MEKK1(MAP3K1), TRAF2, p38 MAPK, Bim, ASK1 (MAP3K5), c-Jun
Putative pathways of MHC class I-dependent postsynaptic long-term depression in major depressive disorder	21	2.98E-04	8.48E-03	6	MHC Class I alpha chain, TAP1 (PSF1), HLA-B, TAP, TAP2 (PSF2), TNF-alpha
Possible regulation of HSF-1/ chaperone pathway in Huntington's disease	21	2.98E-04	8.48E-03	6	HSF1, HSP90 alpha, JNK(MAPK8-10), HSP90, E2I, HSP90 beta
Cytoskeleton remodeling_Regulation of actin cytoskeleton organization by the kinase effectors of Rho GTPases	58	3.09E-04	8.62E-03	10	BETA-PIX, MLCP (reg), Cortactin, Alpha-actinin, PAK, ERM proteins, PIP5KI, MLCK, SLC9A1, RhoGDI alpha
Common mechanisms of Th17 cell migration	48	3.21E-04	8.76E-03	9	MIP-1-beta, G-protein beta/gamma, MCAM, CXCL16, IP10, CCL2, I-TAC, G-protein alpha-i family, CCL7
Neurogenesis_NGF/ TrkA MAPK-mediated signaling	105	3.57E-04	9.52E-03	14	CrkL, SHPS-1, PLAUR (uPAR), PP2A regulatory, PKA-reg (cAMP-dependent), CDK5, PP2A catalytic, p21, RGS2, Fra- 1, p38 MAPK, Ephrin-A receptor 2, SH2B, c-Jun
Cooperative action of IFN-gamma and TNF-alpha on astrocytes in multiple sclerosis	39	3.63E-04	9.52E-03	8	CIITA, I-kB, CD80, PKA-reg (cAMP-dependent), IP10, CCL2, IKK-beta, TNF-alpha
Cigarette smoke-induced oxidative stress and apoptosis in airway epithelial cells	60	4.11E-04	1.06E-02	10	JNK(MAPK8-10), JNK2(MAPK9), C/EBP zeta, SOD1, p21, Cytochrome c, p38 MAPK, ASK1 (MAP3K5), Glutaredoxin, c-Jun
The role of UV radiation in melanoma development	40	4.35E-04	1.10E-02	8	JNK(MAPK8-10), p21, MEKK1(MAP3K1), TRAF2, p38 MAPK, ASK1 (MAP3K5), c-Jun, TNF-alpha
NETosis in SLE	31	4.56E-04	1.13E-02	7	HP1 beta, Fc gamma RII alpha, Histone H2, Histone H2A, p38 MAPK, Histone H1.2, Histone H4
Glucocorticoid-induced elevation of intraocular pressure as glaucoma risk factor	62	5.39E-04	1.27E-02	10	SENP1, Alpha-actinin, ROR2, C/EBP zeta, CLIM1, PLAT (TPA), Antileukoproteinase 1, MLCK, FGFR1, RhoGDI alpha
Development_WNT/Beta-catenin signaling in the nucleus	62	5.39E-04	1.27E-02	10	MED12, JNK(MAPK8-10), HDAC1, MEF2, TLE, RUNX, p38 MAPK, c-Jun, BRG1, GCN5
Cytoskeleton remodeling_Reverse signaling by Ephrin-B	32	5.60E-04	1.27E-02	7	G-protein beta/gamma, Tubulin alpha, RGS3, Ephrin-B, G-protein alpha-i family, SDF-1, WIRE
DNA damage_ATM/ATR regulation of G1/S checkpoint	32	5.60E-04	1.27E-02	7	I-kB, NFBD1, p21, BLM, RAD9, MDM2, CDK2
Apoptosis and survival_IL-17-induced CIKS-dependent MAPK signaling pathways	32	5.60E-04	1.27E-02	7	JNK(MAPK8-10), E2N(UBC13), UBE1, UEV1A, TRAF2, p38 MAPK, IKK-beta
Normal and pathological TGF-beta-mediated regulation of cell proliferation	33	6.83E-04	1.48E-02	7	MLK3(MAP3K11), JNK(MAPK8-10), PDGF receptor, DDX5, p38 MAPK, TGF-beta receptor type I, PDGF-R-beta
TLRs-mediated IFN-alpha production by plasmacytoid dendritic cells in SLE	53	6.89E-04	1.48E-02	9	IRAK2, CDC37, U1-70K, Fc gamma RII alpha, SNRPD1 (SMD1), Ro60, A20, Sec24C, IKK-beta
Immune response_Induction of the antigen presentation machinery by IFN-gamma	53	6.89E-04	1.48E-02	9	CIITA, TAP1 (PSF1), HLA-F, PRMT5, HLA-B, WDR5, ASH2L, PSMB10, TAP2 (PSF2)
MAPK-independent proliferation of normal and asthmatic smooth muscle	64	6.98E-04	1.48E-02	10	I-kB, G-protein beta/gamma, p21, PDGF receptor, PLAT (TPA), G-protein alpha-i family, IKK-beta, FGFR1, TGF-beta receptor type I, PDGF-R-beta

cells

Apoptosis and survival_TNF-alpha-induced Caspase-8 signaling	43	7.25E-04	1.52E-02	8	HSP90 alpha, PP2A regulatory, Caspase-7, PP2A catalytic, HSP90, Cytochrome c, Caspase-6, TNF-alpha
Immune response_CD40 signaling	65	7.91E-04	1.61E-02	10	I-kB, CD80, JNK2(MAPK9), MEKK1(MAP3K1), CCL2, TRAF2, A20, p38 MAPK, IKK-beta, c-Jun
TNF-alpha and IL-1 beta-mediated regulation of contraction and secretion of inflammatory factors in normal and asthmatic airway smooth muscle	65	7.91E-04	1.61E-02	10	GRO-2, p38alpha (MAPK14), JNK(MAPK8-10), CCL2, p38 MAPK, Histone H4, c-Jun, PDGF-R-beta, CCL7, TNF-alpha
DNA damage_Role of SUMO in p53 regulation	17	8.39E-04	1.68E-02	5	SAE2, PML, E2I, MDM2, DAXX
Role of CD8+ Tc1 cells in COPD	44	8.51E-04	1.68E-02	8	CXCL16, IP10, CCL2, KLRK1 (NKG2D), Tyk2, Antileukoproteinase 1, c-Jun, TNF-alpha
Tau pathology in Alzheimer disease	55	9.10E-04	1.77E-02	9	p38alpha (MAPK14), JNK(MAPK8-10), Caspase-7, CDK5, PP2C, PP2A catalytic, Caspase-6, Agrin, p38 MAPK
Development_Transcriptional regulation of megakaryopoiesis	35	9.92E-04	1.90E-02	7	NOTCH1 receptor, Fc gamma RII alpha, ETS, ETS1, THAS, AML1 (RUNX1), LMO2
B-regulatory cells and tumor cells intercellular interaction	67	1.01E-03	1.90E-02	10	LTB, CD80, MCAM, JNK(MAPK8-10), EBI3, CCL2, p38 MAPK, TGF-beta receptor type I, PD-L1, TNF-alpha
Neurophysiological process_Dopamine D2 receptor transactivation of PDGFR in CNS	26	1.04E-03	1.90E-02	6	G-protein beta/gamma, PP2A regulatory, PP2A catalytic, PDGF receptor, G-protein alpha-i family, PDGF-R-beta
Development_NOTCH1-mediated pathway for NF-KB activity modulation	26	1.04E-03	1.90E-02	6	NOTCH1 (NICD), HDAC1, NOTCH1 receptor, NOTCH1 (NEXT), CIR, Histone H4
Transport_RAN regulation pathway	18	1.12E-03	2.02E-02	5	NUP54, Importin (karyopherin)-alpha, RanGAP1, E2I, RanBP1
TTP metabolism	68	1.13E-03	2.02E-02	10	XRN2, PD-ECGF (TdRPase), POLA2, UDP, NDPK A, POLE3 (YBL1), POLG cat, NDPK 6, POLD reg (p68), NDPK D (mitochondrial)
Role of GIP in pathogenesis of type 2 diabetes	46	1.15E-03	2.03E-02	8	p38alpha (MAPK14), PKA-reg (cAMP-dependent), JNK(MAPK8-10), PP2A catalytic, Cytochrome c, TORC2, p38 MAPK, ASK1 (MAP3K5)
Cigarette smoke components TCDD and Benzo[a]pyrene and receptor AHR signaling in lung epithelial cells	36	1.18E-03	2.03E-02	7	ARNT, TGIF, HDAC1, CYP1B1, CCL2, TGF-beta receptor type I, TNF-alpha
Signal transduction_Angiotensin II signaling via Beta-arrestin	57	1.19E-03	2.03E-02	9	Casein kinase II, beta chain (Phosvitin), MLCP (reg), STAR, PP2A catalytic, MYLK1, ASK1 (MAP3K5), MLCK, Beta-arrestin2, 14-3-3
HCV-dependent regulation of membrane receptors signaling in HCC	27	1.28E-03	2.17E-02	6	LTB, I-kB, TRAF2, IKK-beta, c-Jun, TNF-alpha
Development_Hedgehog signaling	47	1.34E-03	2.20E-02	8	Casein kinase I, SPOP, CDC37, HSP90, SAP18, CDK11, DYRK2, Beta-arrestin2
Immune response_TLR5, TLR7, TLR8 and TLR9 signaling pathways	58	1.35E-03	2.20E-02	9	IRAK2, I-kB, JNK(MAPK8-10), E2N(UBC13), UEV1A, p38 MAPK, IKK-beta, c-Jun, TNF-alpha
Immune response_IL-33 signaling pathway	58	1.35E-03	2.20E-02	9	p38alpha (MAPK14), I-kB, JNK(MAPK8-10), CCL2, Histone H2A, Histone H2B, IKK-beta, c-Jun, TNF-alpha
G-protein signaling_Regulation of p38 and JNK signaling mediated by G-proteins	37	1.40E-03	2.23E-02	7	G-protein beta/gamma, JNK(MAPK8-10), MEKK1(MAP3K1), p38 MAPK, G-protein alpha-i family, c-Jun, ARHGEF1 (p115RhoGEF)
Immune response_Oncostatin M signaling via MAPK	37	1.40E-03	2.23E-02	7	JNK(MAPK8-10), MEKK1(MAP3K1), CCL2, p38 MAPK, c-Jun, TIMP1, LIFR
Stellate cells activation and liver fibrosis	70	1.42E-03	2.24E-02	10	GRO-2, I-kB, IRAK1/2, PDGF receptor, CCL2, TRAF2, TIMP1, TGF-beta receptor type I, PDGF-R-beta, TNF-alpha
K-RAS signaling in lung cancer	48	1.54E-03	2.38E-02	8	GRO-2, MIP-1-beta, JNK(MAPK8-10), BTG2, SPRY2, MEKK1(MAP3K1), CCL2, c-Jun
Apoptosis and survival_IL-17-induced CIKS- dependent NF-kB signaling and mRNA stabilization	28	1.56E-03	2.38E-02	6	I-kB, E2N(UBC13), UBE1, UEV1A, TRAF2, IKK-beta
DNA damage_ATM / ATR regulation of G2 / M checkpoint	28	1.56E-03	2.38E-02	6	p38alpha (MAPK14), PLK3 (CNK), NFBD1, p21, BLM, RAD9
Neutrophil chemotaxis in asthma	38	1.65E-03	2.48E-02	7	GRO-2, G-protein beta/gamma, CCR1, CCL2, G-protein alpha-i family, Substance P receptor, CCL7
Transcription_N-CoR/ SMRT complex-mediated epigenetic gene silencing	49	1.77E-03	2.57E-02	8	REV-ERBalpha, UBE2D1, HDAC3, BAF47, Histone H2B, Histone H4, c-Jun, BRG1
PDE4 regulation of cyto/chemokine expression in arthritis	49	1.77E-03	2.57E-02	8	MIP-1-beta, I-kB, PKA-reg (cAMP-dependent), cAMP-GEFI, IP10, CCL2, PDE4, TNF-alpha
Signal transduction_Soluble CXCL16 signaling	49	1.77E-03	2.57E-02	8	I-kB, G-protein beta/gamma, JNK(MAPK8-10), CXCL16, p38 MAPK, G-protein alpha-i family, IKK-beta, TNF-alpha
Regulation of degradation of wtCFTR	20	1.87E-03	2.61E-02	5	UBE2J1, UBE2D1, UBE1, HSP90, Csp
Abnormalities in cell cycle in SCLC	29	1.90E-03	2.61E-02	6	Max, CKS1, Miz-1, p21, MDM2, CDK2
Histone deacetylases in Prostate Cancer	29	1.90E-03	2.61E-02	6	HDAC1, HDAC3, Tubulin alpha, Sirtuin6, p21, HSP90
Apoptosis and survival_p53-dependent apoptosis	29	1.90E-03	2.61E-02	6	p38alpha (MAPK14), JNK(MAPK8-10), Bim, RAD9, c-Jun, MDM2
Transcription_p53 signaling pathway	39	1.93E-03	2.61E-02	7	MKP-7, JNK(MAPK8-10), p21, MEKK1(MAP3K1), MTA2, MDM2, CDK2
Apoptosis and survival_APRIL and BAFF signaling	39	1.93E-03	2.61E-02	7	MIP-1-beta, I-kB, JNK(MAPK8-10), MEKK1(MAP3K1), TRAF2, IKK-beta, CAML
Apoptosis and survival_Ubiquitination and phosphorylation in TNF-alpha-induced NF-kB signaling	39	1.93E-03	2.61E-02	7	UBE2D1, E2N(UBC13), UEV1A, TRAF2, Casein kinase I alpha, IKK-beta, TNF-alpha
Transport_The role of AVP in regulation of Aquaporin 2 and renal water reabsorption	50	2.02E-03	2.65E-02	8	p38alpha (MAPK14), PRKAR2A, PKA-reg type II (cAMP- dependent), PKA-reg (cAMP-dependent), SPA1, cAMP-GEFI, Syntaxin 4, MLCK

Apoptosis and survival_HTR1A signaling	50	2.02E-03	2.65E-02	8	I-kB, G-protein beta/gamma, PP2A regulatory, PKA-reg (cAMP-dependent), PP2A catalytic, Cytochrome c, G-protein alpha-i family, SLC9A1
PDE4 regulation of cyto/chemokine expression in inflammatory skin diseases	50	2.02E-03	2.65E-02	8	PKA-reg (cAMP-dependent), IP10, CCL2, PDE4, p38 MAPK, G-protein alpha-i family, TNF-alpha, 14-3-3
dCTP/dUTP metabolism	74	2.18E-03	2.80E-02	10	XRN2, PD-ECGF (TdRPase), POLA2, AK2, NDPK A, POLE3 (YBL1), POLG cat, NDPK 6, POLD reg (p68), NDPK D (mitochondrial)
G-protein signaling_Rap1A regulation pathway	40	2.25E-03	2.80E-02	7	CrkL, MAGI-1(BAIAP1), SPA1, CALDAG-GEFI, cAMP-GEFI, Rap1GDS1, G-protein alpha-i family
Stem cells_Pancreatic cancer stem cells in tumor metastasis	40	2.25E-03	2.80E-02	7	MLCP (reg), G-protein beta/gamma, CD133, G-protein alpha-i family, SDF-1, MLCK, ARHGEF1 (p115RhoGEF)
Recurrent gene fusions in Prostate Cancer	40	2.25E-03	2.80E-02	7	C19orf25, MgDP1, USP10, MIPOL1, MRPS10, hnRNP A2, DDX5
Stem cells_Neovascularization of glioblastoma in response to hypoxia	30	2.28E-03	2.80E-02	6	ARNT, I-kB, HIF-prolyl hydroxylase, CD133, SDF-1, IKK-beta
Apoptosis and survival_Granzyme A signaling	30	2.28E-03	2.80E-02	6	PHAP1 (pp32), Rab-27A, MUNC13-4, NDPK A, Histone H2B, Lamin B1
Immune response_IFN-gamma signaling via MAPK	51	2.30E-03	2.80E-02	8	MLK3(MAP3K11), IRF9, IP10, SP100, MEKK1(MAP3K1), p38 MAPK, c-Jun, TNF-alpha
Epithelial cell anoikis in COPD	51	2.30E-03	2.80E-02	8	Alpha-actinin, JNK(MAPK8-10), Beta-parvin, Bak, Laminin 11, p38 MAPK, Bim, ASK1 (MAP3K5)
Apoptosis and survival_CXCR3-B signaling	63	2.43E-03	2.88E-02	9	G-protein beta/gamma, PKA-reg (cAMP-dependent), IP10, p21, Cytochrome c, I-TAC, NRF2, p38 MAPK, Beta- arrestin2
Neuroprotective action of lithium	63	2.43E-03	2.88E-02	9	p38alpha (MAPK14), HSF1, CDK5, JNK2(MAPK9), FRAT1, p38 MAPK, Bim, ASK1 (MAP3K5), c-Jun
Vascular endothelial cell damage in SLE	63	2.43E-03	2.88E-02	9	JNK(MAPK8-10), Caspase-7, CCL2, TRAF2, p38 MAPK, PD-L1, AML1 (RUNX1), TNF-alpha, VEGF-B
Signaling Transduction_Role of MIF as an intracellular mediator	41	2.60E-03	3.01E-02	7	JNK(MAPK8-10), HDAC1, HDAC3, p21, Bim, c-Jun, MDM2
Development_Muscle progenitor cell migration in hypaxial myogenesis	41	2.60E-03	3.01E-02	7	NOTCH1 (NICD), G-protein beta/gamma, NOTCH1 receptor, NOTCH1 (NEXT), G-protein alpha-i family, SDF- 1, FGFR1
Immune response_Lysophosphatidic acid signaling via NF-kB	52	2.61E-03	3.01E-02	8	PKD, JNK(MAPK8-10), Caspase-7, CCL2, A20, p38 MAPK, IKK-beta, Beta-arrestin2
dGTP metabolism	64	2.72E-03	3.10E-02	9	XRN2, POLA2, NDPK A, POLE3 (YBL1), POLG cat, NDPK 6, POLD reg (p68), PPAL, NDPK D (mitochondrial)
Chemotaxis_Lysophosphatidic acid signaling via GPCRs	129	2.73E-03	3.10E-02	14	MLCP (reg), G-protein beta/gamma, Rho GTPase, PRKD1, TAZ, JNK(MAPK8-10), Caspase-7, PAK, p21, p38 MAPK, G-protein alpha-i family, c-Jun, G-protein gamma 12, ARHGEF1 (p115RhoGEF)
Inhibition of RUNX3 signaling in gastric cancer	22	2.94E-03	3.26E-02	5	Caspase-7, HDAC1, p21, Bim, TIMP1
Immune response_HMGB1/RAGE signaling pathway	53	2.95E-03	3.26E-02	8	MIP-1-beta, I-kB, MEF2A, JNK(MAPK8-10), PLAT (TPA), p38 MAPK, c-Jun, TNF-alpha
Chemotaxis_CCR1 signaling	53	2.95E-03	3.26E-02	8	I-kB, G-protein beta/gamma, CCR1, CCL13, p38 MAPK, G-protein alpha-i family, IKK-beta, CCL7
IL-1 signaling in melanoma	42	3.00E-03	3.27E-02	7	GRO-2, I-kB, JNK(MAPK8-10), p21, CCL2, c-Jun, PD-L1
TNF-alpha, IL-1 beta induce dyslipidemia and inflammation in obesity and type 2 diabetes in adipocytes	42	3.00E-03	3.27E-02	7	PTX3, PKA-reg (cAMP-dependent), CCL2, TRAF2, IKK-beta, Perilipin, TNF-alpha
Immune response_BAFF-induced non-canonical NF-kB signaling	32	3.21E-03	3.47E-02	6	UBE1C, E2N(UBC13), UBE1, UEV1A, TRAF2, E2I
GTP metabolism	54	3.32E-03	3.56E-02	8	ITPA, Guanylate cyclase A (NPR1), NDPK A, PDE6G, Guanylate cyclase C (GC-C), NDPK 6, PDE6H, NDPK D (mitochondrial)
Signal transduction_PDGF signaling via JAK-STAT and reactive oxygen species (ROS)	66	3.37E-03	3.58E-02	9	PKA-reg (cAMP-dependent), IP10, CCL13, PDGF receptor, CCL2, Tyk2, I-TAC, PDGF-R-beta, CCL7
Proteolysis_Putative ubiquitin pathway	23	3.62E-03	3.78E-02	5	UBE2D1, E2N(UBC13), UBE1, UEV1A, FBXW7
Development_Thrombopoietin signaling via JAK-STAT pathway	23	3.62E-03	3.78E-02	5	CrkL, TAP1 (PSF1), CISH, p21, Tyk2
Immune response_Platelet activating factor/ PTAFR pathway signaling	55	3.73E-03	3.84E-02	8	G-protein beta/gamma, PKA-reg (cAMP-dependent), CCL2, Tyk2, p38 MAPK, ASK1 (MAP3K5), G-protein alpha-i family, Beta-arrestin2
Immune response_CD28 signaling	55	3.73E-03	3.84E-02	8	I-kB, CD80, JNK(MAPK8-10), MEKK1(MAP3K1), PIP5KI, p38 MAPK, IKK-beta, c-Jun
Immune response_Gastrin in inflammatory response	68	4.13E-03	4.22E-02	9	ELAVL1 (HuR), GRO-2, p38alpha (MAPK14), I-kB, JNK(MAPK8-10), MEF2, MEKK1(MAP3K1), IKK-beta, c-Jun
MAPK-mediated proliferation of normal and asthmatic smooth muscle cells	56	4.18E-03	4.24E-02	8	p38alpha (MAPK14), G-protein beta/gamma, PDGF receptor, PLAT (TPA), G-protein alpha-i family, FGFR1, TGF-beta receptor type I, PDGF-R-beta
dATP/dITP metabolism	94	4.24E-03	4.27E-02	11	XRN2, ITPA, POLA2, AK2, NDPK A, POLE3 (YBL1), POLG cat, NDPK 6, POLD reg (p68), PPAL, NDPK D (mitochondrial)
CFTR folding and maturation (normal and CF)	24	4.40E-03	4.37E-02	5	HSP90 alpha, Hdj-2, Csp, Sti1, HSP90 beta
G-protein signaling_RhoA regulation pathway	34	4.41E-03	4.37E-02	6	Ephrin-A receptors, Rap1GDS1, GRAF, RhoGDI alpha, ARHGEF1 (p115RhoGEF), VIL2 (ezrin)
Signal transduction_Angiotensin II/ AGTR1 signaling via JAK/STAT	45	4.48E-03	4.41E-02	7	I-kB, p21, RGS2, Tyk2, BNP, c-Jun, TIMP1
Immune response_TLR2 and TLR4 signaling pathways	69	4.56E-03	4.45E-02	9	IRAK2, I-kB, JNK(MAPK8-10), E2N(UBC13), UEV1A, p38 MAPK, IKK-beta, c-Jun, TNF-alpha
Immune response_IL-1 signaling pathway	82	4.67E-03	4.47E-02	10	p38alpha (MAPK14), I-kB, JNK(MAPK8-10), MCPIP, IP10, MEKK1(MAP3K1), CCL2, MYLK1, CCL7, TNF-alpha

Skeletal muscle atrophy in COPD	57	4.67E-03	4.47E-02	8	ALK-4, ELAVL1 (HuR), p38alpha (MAPK14), I-kB, p38 MAPK, TGF-beta receptor type I, TNF-alpha, 14-3-3
Transcription_Epigenetic regulation of gene expression	57	4.67E-03	4.47E-02	8	PRMT5, HDAC1, HDAC3, Sirtuin6, Histone H2A, Histone H2B, Histone H4, GCN5
Apoptosis and survival_IL-17-induced CIKS-independent signaling pathways	46	5.07E-03	4.82E-02	7	p38alpha (MAPK14), JNK(MAPK8-10), c-Jun/Fra-1, Fra-1, Tyk2, p38 MAPK, c-Jun
Altered Ca2+ handling in heart failure	35	5.11E-03	4.82E-02	6	BIN1 (Amphiphysin II), Phospholamban, G-protein beta/gamma, PP2A catalytic, PDE4D, G-protein alpha-i family
TGF-beta signaling via kinase cascades in breast cancer	58	5.20E-03	4.82E-02	8	p38alpha (MAPK14), MLK3(MAP3K11), JNK(MAPK8-10), p38 MAPK, IKK-beta, c-Jun, TGF-beta receptor type I, TNF- alpha
Apoptosis and survival_Role of nuclear PI3K in NGF/ TrkA signaling	25	5.29E-03	4.82E-02	5	Nucleolin, Bim, Zyxin, Ebp1, Acinus
Cytoskeleton remodeling_Neurofilaments	25	5.29E-03	4.82E-02	5	Tubulin gamma, DCTN2, CDK5, Tubulin alpha, Tubulin gamma 1
HSP70 and HSP40-dependent folding in Huntington's disease	25	5.29E-03	4.82E-02	5	HSP90 alpha, Hdj-2, HSP90, Sti1, HSP90 beta
Inhibition of TGF-beta signaling in gastric cancer	25	5.29E-03	4.82E-02	5	Praja-1, HDAC1, p21, Bim, TGF-beta receptor type I
Cytoskeleton remodeling_Substance P mediated membrane blebbing	16	5.43E-03	4.91E-02	4	MLCP (reg), Tubulin alpha, Substance P receptor, MLCK
Blood coagulation_GPCRs in platelet aggregation	71	5.52E-03	4.95E-02	9	MLCP (reg), G-protein beta/gamma, PKA-reg (cAMP- dependent), CALDAG-GEFI, Syntaxin 4, RAP-1B, G-protein alpha-i family, Calmyrin, ARHGEF1 (p115RhoGEF)
SLE genetic marker-specific pathways in antigen-presenting cells (APC)	84	5.54E-03	4.95E-02	10	I-kB, TNIP1, IRF9, Fc gamma RII alpha, KLRK1 (NKG2D), Tyk2, TRAF2, A20, IKK-beta, TNF-alpha

Items with FDR < 0.05 were considered as significant ones.
